# Bis(pinacolato)diboron‐Enabled Ni‐Catalyzed Reductive Arylation/Vinylation of Alkyl Electrophiles

**DOI:** 10.1002/advs.202404301

**Published:** 2024-06-17

**Authors:** Deli Sun, Yuxin Gong, Yu Wu, Yunrong Chen, Hegui Gong

**Affiliations:** ^1^ School of Resources and Environmental Engineering Shanghai Polytechnic University No. 2360 Jinhai Road Shanghai 201209 China; ^2^ Center for Supramolecular Chemistry and Catalysis Department of Chemistry Shanghai University Shanghai 200444 China

**Keywords:** cross‐coupling, diboron, electrophile, nickel, reductant

## Abstract

Herein, the use of economically and environmentally friendly bis(pinacolato)diboron (B_2_Pin_2_) is described as a non‐metallic reductant in mediating Ni‐catalyzed C(sp^3^)–C(sp^2^) reductive cross‐coupling of alkyl electrophiles with aryl/vinyl halides. This method exhibits excellent suitability for heteroaryl halides and alkyl halides/Katritzky salts. The present study is compatible with an in situ halogenation of alcohol method, allowing for selective mono‐functionalization of diols and bio‐relevant alcohols (e.g., carbohydrates). The use of B_2_Pin_2_ shows potential for easy scalability without introducing additional metal impurities into the products. It is observed for the first time in the realm of cross‐electrophile coupling chemistry that B_2_Pin_2_ can sever as a reductant to reduce Ni^II^ to Ni^0^. This mechanistic insight may inspire the development of new reductive bond‐forming methodologies that can otherwise be difficult to achieve with a metal reductant.

## Graphical abstract

The use of economic bis(pinacolato)diboron as the non‐metal reductant to direct the Ni‐catalyzed reductive arylation and vinylation of alkyl halides/ Katritzky salts with the C(sp^2^)–halides are achieved, which is characteristic of easy removal of boron residue and homogeneous nature of the reaction that are critical concerns for large‐scale and eco‐friendly process chemistry.

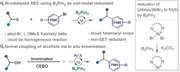

## 1 Introduction

**Figure 1:**
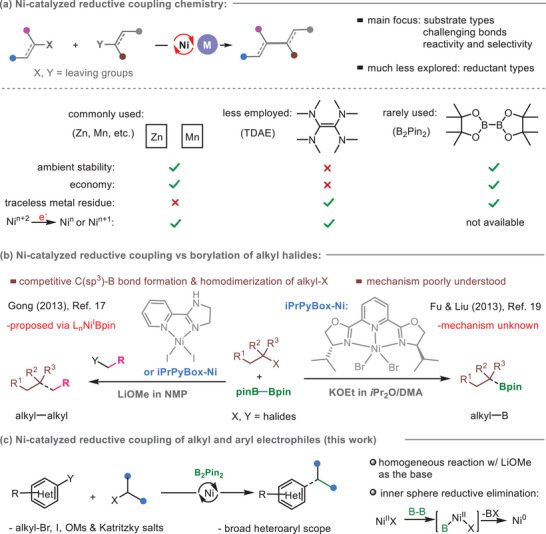
Brief summary of the current focuses of Ni‐catalyzed cross‐electrophile coupling with emphasis on the reductants.

Transition metal‐catalyzed cross‐electrophile coupling (XEC) has evolved into a privileged synthetic platform that significantly advances the coupling chemistry (For selected reviews on transition metal catalyzed traditional couplings, see ref. [1]) (For seminal work and applications on Ni‐catalyzed coupling chemistry, see ref. [2]) (For selected reviews on cross‐electrophile coupling chemistry, see ref. [3]).^[^ ^1^, ^2^, ^3^, ^4^, ^5^ ^]^ In this vein, metal reductants, notably Zn and Mn, have been predominantly used in developing methodologies for transforming unusual electrophiles,^[^ ^6^, ^7^, ^8^, ^9^ ^]^ (For carbonylation, see ref. [9]) forging challenging bonds, (For the examples of preparation of C‐heteroatom products, see ref. [10]) and addressing issues of chemo‐ and stereoselectivity (**Figure** **1**a).^[^ ^5^, ^11^, ^12^ ^]^ However, the drawbacks associated with metal reductants, including scalability issues and the introduction of metallic impurities in pharmaceuticals, are well recognized.^[^ ^13^ ^]^ To date, only meager efforts have been dedicated to the development of non‐metal reductants that can outcompete Zn and Mn. A noteworthy example is the use of tetra(dimethylamino)ethylene (TDAE) as the 1e SET reductant in Ni‐catalyzed aryl–alkyl XEC coupling.^[^ ^14^ ^]^ Unfortunately, the laborious and costly preparation of moisture‐ and air‐sensitive TDAE hampers its application (Figure 1a).^[^ ^15^ ^]^ Recently, photoredox/electrochemical protocols have emerged as important alternatives to (thermal) XEC chemistry (For leading reviews, see ref. [16]). However, the requirements for high operational efficiency, easy scalability, and convenience for mechanistic studies continue to fuel demand for the development of new reductants in the realm of XEC chemistry.

We initially discovered that using B_2_Pin_2_ as a cost‐effective and environmentally friendly reductant significantly enhances the chemoselectivity of Ni‐catalyzed C(sp^3^)–C(sp^3^) coupling of primary with secondary alkyl halides compared to that using Zn (Figure 1b),^[^ ^5a^ ^,17,18]^ (For other examples of B_2_Pin_2_‐mediated C(sp^3^)–C(sp^3^) bond formation, see ref. [18], For a recent Fe‐catalyzed alkyl‐aryl coupling, see ref. [18d]). However, achieving an equivalent version of C(sp^3^)–C(sp^2^) XEC chemistry has proven elusive. In a broader context, the XEC chemistry mediated by the Ni/B_2_Pin_2_ platform has received surprisingly less attention, and the reaction mechanisms of the disclosed methods remain unexplored. This challenge is attributed to the distinctive nature of B_2_Pin_2_ in mediating Ni‐catalyzed XEC chemistry. First, the 1e SET process associated with Zn/Mn is not applicable to B_2_Pin_2_, and thus far, the mechanism of reducing Ni intermediates with B_2_Pin_2_ remains poorly understood. Second, the competitive formation of alkyl–Bpin could become problematic, as observed as a primary pathway by simply varying the solvents and bases in Ni‐catalyzed C(sp^3^)–C(sp^3^) XEC process (Figure 1b),^[^ ^19^ ^]^ The profound impact of bases on Ni/B_2_Pin_2_‐mediated reactions has also been demonstrated in fine‐tuning the regioselectivity for borylation and arylation of cyclohexenes with B_2_Pin_2_ and aryl halides.^[^ ^20^ ^]^

Herein, we present an unprecedented Ni/B_2_Pin_2_‐reductive coupling protocol for the arylation/vinylation of alkyl halides/Katritzky salts with aryl/vinyl halides, effectively furnishing C(sp^3^)–C(sp^2^) bonds. The present studies encompass three noteworthy aspects. First, it showcases that B_2_Pin_2_ is a comparably versatile reductant to Zn/Mn for reductive C─C coupling. Second, it provides new mechanistic insight, revealing that B_2_Pin_2_ is capable of reducing L_n_Ni^II^X_2_ to L_n_Ni^0^ in the XEC framework. This contradicts our initial proposal of L_n_Ni^I^Bpin involved in the C(sp^3^)–C(sp^3^) XEC.^[^ ^17^ ^]^ It should be noted that the reduction of NiX_2_ (X = Br, Cl) to Ni(0) via in situ formation of Ni(OMe)_2_ with B_2_(OR)_4_ in the presence of 2 equiv of (*i*Pr)_2_NEt in methanol has been disclosed. However, given the sophisticated effect of bases and solvents on the mechanism of Ni/B_2_Pin_2_ reactions,^[^ ^21^ ^]^ our finding could be critical for future development of B_2_Pin_2_‐mediated XEC chemistry (Figure 1c). Finally, a homogeneous reaction could be attained based on a combination of B_2_Pin_2_/LiOMe/TBAI (tetrabutylammonium iodide), which holds promise for practical large‐scale Ni‐catalyzed XEC process (The use of metal reductant (e.g., Mn, Zn and Mg), can lead to handling and waste disposal issues when employed on a large scale, see ref. [22]). Notably, the boron residue can be easily removed during work‐up, avoiding the introduction of extra metal impurities into the products.^[^ ^23^ ^]^

## 2 Results and Discussion

**Figure 2:**
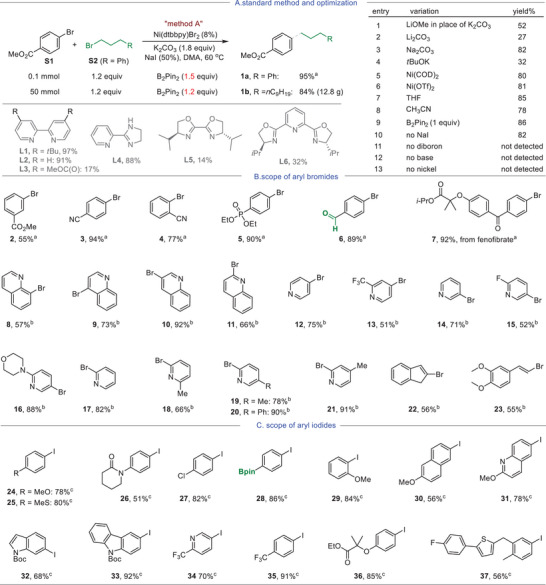
Optimization of the reaction conditions and scope of aryl halides. Note: a) standard Method A was used for electron‐deficient aryl bromides; b) for heteroaryl bromide, a similar procedure to the standard method A was used, except that dtbbpy (2.7 mg, 10 mol %) and NiCl_2_(Py)_4_ (3.6 mg, 8 mol %) displaced (dtbbpy)NiBr_2_, and NaI (30 mg, 2 equiv) and TBAI (18 mg, 0.5 equiv) were employed; c) for electron rich aryl iodides, a similar procedure to the standard method A was used except that NaI (30 mg, 2 equiv), TBAI (18 mg, 0.5 equiv) and alkyl bromide (1.5 equiv) were employed.

We commenced our investigation into the coupling of methyl 4‐bromobenzoate (**S1)** with (3‐bromopropyl)benzene (**S2**) following the (**L4**)Ni/B_2_Pin_2_‐meidated conditions for alkyl–alkyl bond formation, using LiOMe as base and *N*‐Methyl‐2‐pyrrolidone (NMP) as the solvent (Figure 1b; Equation S1, Supporting Information).^[^ ^17^ ^]^ Trace amounts of coupling product **1a** were detected alongside dimerization of alkyl halides and the recovery of aryl halides as the mass balance. After extensive screening of reaction parameters,^[^ ^24^ ^]^ we identified that the ligand, base and solvent are key factors in obtaining the optimized reaction conditions, which entailed the use of (**L1**)NiBr_2_ (**L1**, dtbbpy, 4,4'‐di‐*tert*‐butyl‐2,2'‐bipyridine) and 1.2 equiv of B_2_Pin_2_ with K_2_CO_3_ and NaI serving as the additives in *N,N*‐dimethyl acetamide (DMA) at 60 °C (Method A, **Figure** **2**A). Exploration with other bases, including Li_2_CO_3_, Na_2_CO_3_ and KO*t*Bu (entries 1–4, Figure 2A), alternative nickel sources (entries 5–6, Figure 2A), solvents (entries 7–8, Figure 2A), and 1 equiv of B_2_Pin_2_ (entry 9, Figure 2A), did not lead to improved results. Control experiments underscored the indispensability of diboron, base, and Ni catalyst for the coupling reaction (entries 10–13, Figure 2A). Other bipyridine ligands generally exhibited effectiveness (e.g., **L2**, Figure 2A), except for those bearing electron‐withdrawing groups (e.g., **L3**). Engagement of 2‐(4,5‐dihydro‐1*H*‐imidazol‐2‐yl)pyridine **L4**, biox and pybox ligands (**L5** and **L6)** proved less effective than **L1**. Upon scaling the reaction to 10 gram‐scale using 50 mmol of ArBr, the yield of **1b** was marginally reduced to 84%, indicating the robustness of the method (Figure 2A).

More importantly, we noticed that the reaction mixture with method A remained heterogeneous throughout the reaction suggesting likely due to the use of K_2_CO_3_. Thus, slight modifications to method A were implemented using LiOMe as the base, TBAI as the additive and DMA/THF (1/1, v/v, THF: tetrahydrofuran) as the solvent. The resulting reaction mixture remained homogeneous throughout the reaction course. Utilizing 1.2 equiv and 1.5 equiv of **S2** gave **1a** in 80% and 91% yields, respectively (Method B, Equation (1); Equation S2, Supporting Information). To the best of our knowledge, a homogeneous Ni‐catalyzed cross‐electrophile coupling reaction using an economical reductant has not been disclosed in the literature.


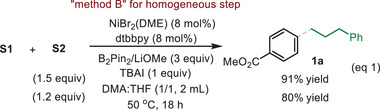


Next, the compatibility of aryl halides was assessed using Method A (Figure 2A), with (3‐bromopropyl)benzene (**S2**) as the coupling partner. A brief screening of electron‐deficient arenes suggested that aryl bromides decorated with ester, cyano, phosphoryl, aldehyde, and ketone functionalities were all competent substrates, affording products **2–7** in moderate to excellent yields. Notably, *meta*‐ester **2** exhibited a lower yield compared to its *para*‐counterpart **1a**. Efficient alkylation of a bromo‐analog of fenofibrate gave **7** in 82% yield. The method proved highly effective for heteroarenes. Coupling of the 2‐, 3‐, 4‐, and 8‐bromoquinolines was satisfactory, giving products **8–11** in good results. Similarly, 4‐, 3‐, and 2‐bromopyridines and their derivatives performed well, giving products **12–21** in good results. Noteworthy is the otherwise easy dimerization of 2‐bromopyridine in Zn/Mn conditions to form bipyridine was not problematic as evidenced in **17–21**.^[^ ^25^ ^]^ The present method was also suitable for vinyl bromides as manifested by the examples of **22** and **23**. For electron‐rich arenes, the use of aryl iodides was necessary, with the assistance of 2 equiv of NaI and 50 mol% of tetrabutylammonium iodide (TBAI) (Figure 2C). Good to excellent yields were obtained for **24**–**30**. Comparable results were observed for 4‐ and 2‐methoxy arenes **25** and **29**, indicating that *ortho*‐substitution pattern for MeO has only a minor impact on the coupling efficiency. The chloro‐ and Bpin‐ groups were well‐tolerated as exemplified by **27**–**28**. Heteroarenes such as 6‐iodo‐2‐methoxyquinoline, iodoindole, 9*H*‐carbazole, 2‐CF_3_‐5‐iodopyridine and 1‐iodo‐4‐(trifluoromethyl)benzene were all compatible with the method, as shown by examples **32**–**35**. Finally, alkylation of the iodo analog of bioactive deschloroclofibrate and iodo‐(fluorophenyl)methylbenzyl)thiophene gave **36** and **37** in 85% and 56% yields, respectively.

**Figure 3:**
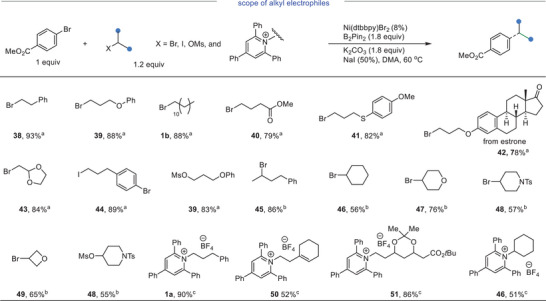
Scope of alkyl electrophiles. Note: a) unless otherwise noted, standard method A was used; b) for the coupling of aryl halides with secondary alkyl bromides, a similar procedure to the standard method A was used, except that Ni(OTf)_2_ (8 mol %) and dtbbpy (10 mol %) displaced (dtbbpy)NiBr_2_, and NaI (2 equiv), TBAI (0.5 equiv) and alkyl bromide (1.5 equiv) were employed; c) for the coupling of alkyl‐pyridinium salts, a similar procedure to the standard method A was used, except that NaI (1 equiv) and alkyl‐pyridinium salt (1.5 equiv) were employed.

Next, we studied the coupling efficiencies for the reactions of methyl 4‐bromobenzoate with a wide range of alkyl electrophiles (**Figure** **3**). Primary and secondary alkyl bromides, iodides and mesylates were generally effective as evident in the examples of **38**–**49**. The method was also suitable for a range of alkyl–Katritzky pyridinium salts, generating **1a, 46, 50 and 51** in moderate to good yields (Figure 3).

**Figure 4:**
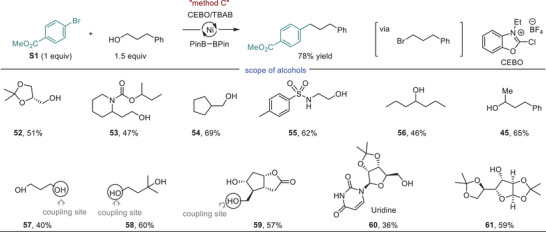
Formal coupling of alcohols and diols with aryl halides. Method C was used: alcohol (1.5 equiv) was treated with CEBO (1.5 equiv) and TBAB (tetrabutylammonium bromid1.5 equiv) in CH_3_CN (0.2 mL) for 5 min, before addition of the rest of ingredients as described in method A including 8 mol% NiBr_2_(dtbbpy), 180 mol% B_2_Pin_2_, 180 mol% K_2_CO_3_, 200 mol% NaI, DMA (0.4 mL). The reactions were run at 60 °C.

Additionally, we integrated our previously reported in situ halogenation of alcohols method with the diboron‐mediated coupling process to develop a protocol for in situ arylation of alcohols.^[^ ^26^ ^]^ The use of CEBO/TBAB (CEBO = 2‐chloro‐3‐ethylbenzoxazolium tetrafluoroborate) has proven to be a powerful handle to convert primary and secondary alcohols into their bromide counterparts within 1–5 min, exhibiting high mono‐selectivity for halogenation of polyols at the less‐hindered site. This technique facilitated a quick screening of various primary and secondary alcohols to couple with **S1**, giving **52–56** and **45** in synthetically useful yields (Method C, **Figure** **4**). The unprotected diols were also compatible with method C, offering **57–59** in mono‐ and stereoselective manners. The utility of the method was also showcased by facile functionalization of uridine and diacetone‐D‐glucose to yield **60** and **61**, respectively.

Lastly, we demonstrated that B_2_Pin_2_ serves as an effective reductant akin to TDAE in a three‐component reaction involving α‐bromoester, but‐3‐en‐1‐ylbenzene and **S1**, yielding **1c** in 80% yield (Equation (2)).^[^ ^14c^ ^]^ This was previously unsuccessful when Zn was employed as the reductant. This outcome suggests that B_2_Pin_2_ may function as a complementary reductant to enable reactions that have failed with reductants Zn and Mn.


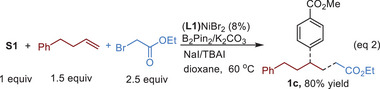


## 3 Mechanistic Studies

**Figure 5:**
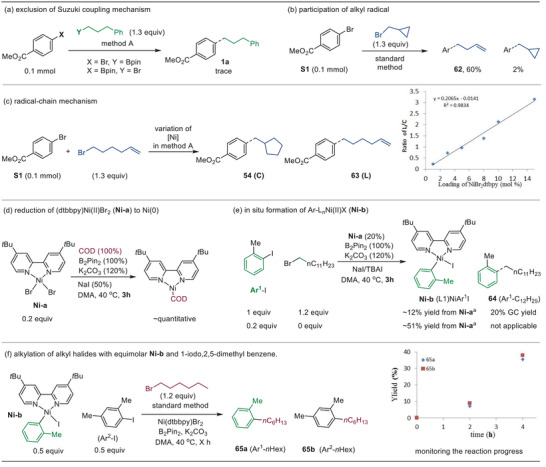
Mechanistic considerations. a) ^1^H NMR yields estimated using triazine as an internal standard (see Figures S1–S4, Supporting Information, for details).

To understand the details of the reaction process, we first evaluated whether the in situ formation of organoboron followed by Suzuki coupling was a viable process. The reactions of (4‐methoxycarbonyl)phenyl–Bpin with the alkyl bromide **S2**, as well as phenylpropyl–Bpin with the aryl bromide **S1** did not yield the coupling product **1a** (**Figure** **5**a), thus a mechanism of in situ Suzuki coupling was excluded.

Next, we established that the reaction accommodated a radical process, as suggested by the coupling of bromocyclopropyl methane with **S1**, which furnished the ring‐opening product **62** (Figure 5b). The coupling of **S1** with 6‐bromohex‐1‐ene as a radical clock was examined to give a mixture of the cyclized and linear products **54** and **63**. A linear dependence of their ratios on the concentration of Ni catalyst was observed, indicative of a radical‐chain process featuring diffusion of alkyl radical into the bulk solution, followed by trapping the radical with a Ni species (Figure 5c).^[^ ^27^ ^]^

**Scheme 1:**
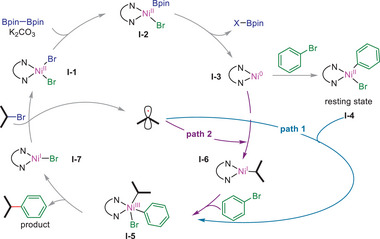
Possible reaction paths for the reductive coupling event.

Perhaps a more instructive result came from the detection of reduction of L_n_Ni^II^Br_2_ (**Ni‐a**) to L_n_Ni^0^ by B_2_Pin_2_ under the conditions similar to method A. First, a near‐quantitative amount of of (dtbbpy)Ni^0^(COD) (COD: 1,5‐cyclooctadiene) was observed upon exposure of (dtbbpy)NiBr_2_ (**Ni‐a**, 0.2 equiv) to B_2_Pin_2_ in the presence of COD after 3 h (Figure 5d; Figure S6, Supporting Information). Next, the formation of Ni^0^ was also evidenced by observation of (dtbbpy)Ni^II^(Ar^1^) (**Ni‐b**, dtbbpy = **L1**) in the reaction mixture of 2‐methyl iodobenzene (Ar^1^–I, 1.0 or 0.2 equiv) with or without 1‐bromododecane in the presence of (dtbbpy)Ni^II^Br_2_ (**Ni‐a**, 0.2 equiv) and stoichiometric amount of B_2_Pin_2_, K_2_CO_3_ and NaI in DMA after 3–5 h (Figure 5e; Figures S1–S4, Supporting Information).^[^ ^28^ ^]^ We reasoned that reduction of L_n_Ni^II^X_2_ (**I‐1**) to L_n_Ni^0^ (**I‐3**) under the present reductive coupling conditions,^[^ ^21^ ^]^ likely through reductive omission of Bpin–X from a putative L_n_Ni^II^(Bpin)X species (**I‐2**) (**Scheme** **1**).

As illustrated above, a radical‐chain mechanism (Figure 5c) is proposed in the present method, which likely involves the trapping of an alkyl radical with L_n_Ni^II^(Ar)X (**I‐4**, path 1, Scheme 1) or L_n_Ni^0^ (**I‐3**, path 2, Scheme 1) to yield L_n_Ni^III^(Ar)(alkyl)X (**I‐5**) or L_n_Ni^I^(alkyl) (**I‐6**), respectively.^[^ ^27^, ^29^ ^]^ Whereas path 1 represents the most wildly accepted mechanism in the literature, path 2 has recently emerged as an important alternative pathway in certain methods.^[^ ^29^ ^]^ In path 2, the oxidative addition of ArX to **I‐6** also leads to **I‐5**. In both pathways, reductive elimination of **I‐5** affords the product along with L_n_Ni^I^ (**I‐7**). Subsequent halide abstraction of alkyl halide with **I‐7** produces an alkyl radical and L_n_Ni^II^ (**I‐1**).

To discern the more advantageous process between the two mechanisms, we conducted a control experiment wherein an equimolar mixture of **Ni‐b** and 1‐iodo‐2,4‐dimethylbenzene (Ar^2^–I) (0.5 equiv) was introduced to the catalytic reaction containing 1.2 equiv of hexyl bromide (Figure 5f). We observed a slight preference for the formation of product **65b** arising from Ar^2^–I over **65a** derived from **Ni‐b**. Further, a control study indicated that **Ni‐b** remained reasonably stable under the same reaction conditions without Ar^2^–I and *n*‐hexyl bromide within 4 h (Figure S8, Supporting Information). In Path 1, the oxidative addition of ArX to L_n_Ni^0^ (**I‐3**) giving L_n_Ni^II^(Ar)X (**I‐4**) is crucial. Therefore, if Path 1 is the sole reaction pathway, the formation of **65a** would significantly exceed that of **65b**. This is because the concentration of preformed **Ni‐b** should be higher than that of in situ formed (dttppy)Ni^II^(Ar2)I (**Ni‐b’**) via the oxidative addition of Ar^2^–I to (dtbbpy)Ni^0^. However, the contrary observations suggest that Path 1 alone should not determine the predominance of the catalytic process (Scheme 1). This inference aligns with the significant observation of **Ni‐b** in the coupling reaction (Figure 5e), potentially representing a catalyst resting state. Based on the collective mechanistic studies, we propose that paths 1 and 2 may concurrently operate in our B_2_Pin_2_‐mediated reductive coupling event. The preference between the two pathways may vary depending on the stereoelectronic nature of the coupling electrophiles.

The kinetic profiles of the reaction, built upon initial rate models, were collected using Ni(COD)_2_/dtbbpy to substitute NiBr_2_/dtbbpy. This substitution was made in order to avoid errors induced by long induction periods (Figures S10–S28, Supporting Information).^[^ ^30^, ^31^ ^]^ The results revealed a first‐order dependence on the concentration of B_2_Pin_2_, suggesting that B_2_Pin_2_ may merely participate in the reduction of Ni(II) to Ni(0), which likely accounts for a rate‐determining step. In addition, a positive rate dependence on Ni(COD)_2_/dtbbpy was observed (k_obs_ = 0.2), indicating that Ni may engage in multiple transformations (e.g., paths 1 and 2), including formation of Ar–L_n_Ni^II^X (**I‐4**) species observed in the reaction. Finally, inverse reaction rate orders in both alkyl bromide **S1** and aryl bromide **S2** were noted, likely due to off‐cycle mechanism for the two electrophiles. The off‐cycle consumption of alkyl halides at elevated concentration could come from side reactions including formation of alkyl–Bpin and alkyl–alkyl dimers. Moreover, the undesired reaction of L_n_Ni^I^X with ArX (Figure S9, Supporting Information), and decomposition of Ar–L_n_Ni^II^X may account for the negative rate dependence on the concentration of aryl halides.^[^ ^20^ ^]^

## 4 Conclusion

In summary, we have demonstrated that B_2_Pin_2_ serves as an effective terminal reductant to mediate the Ni‐catalyzed C(sp^3^)–C(sp^2^) cross‐electrophile coupling of alkyl electrophiles with aryl/vinyl halides. The method circumvents the need for metal reductants, rendering it more environmentally benign and easily scalable, as the reaction can be adjusted to homogeneous. The versatility of the reaction was illustrated by a broad array of examples, encompassing electron‐rich aryl iodides, electron‐deficient aryl bromides, and various heteroarenes. Alkyl electrophiles ranging from halides, mesylates, Katritzky salts, to alcohols/diols derived from sugars were successfully employed.

Our mechanistic investigations revealed that B_2_Pin_2_ effectively reduces Ni(II) salt to Ni(0), a departure from the electron‐transfer reduction by metallic reductants. Furthermore, the reaction mechanism suggests the involvement of a radical chain process, emphasizing the trapping of the alkyl radical with L_n_Ni^0^. However, the well‐established mechanism involving the interception of an alkyl radical with L_n_Ni^II^(Ar)X may operate concurrently. The utilization of diboron ester as the reductant opens avenues for novel reductive coupling approaches, addressing challenges posed by traditional metal reductants, and holds promise for industrial applications.

## Conflict of Interest

The authors declare no conflict of interest.

## Supporting information

Supporting Information

## Data Availability

The data that support the findings of this study are available in the supplementary material of this article.
