# Supplementary material for: Bis(pinacolato)diboron‐Enabled Ni‐Catalyzed Reductive Arylation/Vinylation of Alkyl Electrophiles
Source: Adv Sci (Weinh). 2024 Jun 17;11(31):2404301. doi: 10.1002/advs.202404301 (PMC11336967; doi:10.1002/advs.202404301)

ADVANCED SCIENCE
Open Access

Supporting Information

for *Adv. Sci.*, DOI 10.1002/advs.202404301

Bis(pinacolato)diboron-Enabled Ni-Catalyzed Reductive Arylation/Vinylation of Alkyl Electrophiles

Deli Sun, Yuxin Gong, Yu Wu, Yunrong Chen and Hegui Gong**

Supporting Information

Bis(pinacolato)diboron-Enabled Ni-Catalyzed Reductive Arylation/Vinylation of Alkyl Electrophiles

Deli Sun,[a],[†] Yuxin Gong,[b],[†] Yu Wu,[b] Yunrong Chen[b]* and Hegui Gong[b]*

[a]School of Resources and Environmental Engineering, Shanghai Polytechnic University, No. 2360 Jinhai Road, Shanghai 20[1]209, China

[b]Center for Supramolecular Chemistry and Catalysis, College of Sciences, Shanghai University, No. 99 Shang-Da Road, Shanghai 200444, China

[†]These contributed equally.

Supporting Information Placeholder

Table of Contents

I. Experimental Section S2

Part 1. General Information S2

Part 2. Optimization Experiments S2

Part 3. Cross-Coupling Reactions and Product Characterization S5

Part 4. Homogeneous Reaction Conditions S32

Part 5. Initial Test of a Three-Component Reaction of Alkyl Halide/Alkene/Aryl halide S33

Part 6. Mechanistic Consideration S34

Part 7. Kinetic Studies S44

II. Reference S55

III. NMR Data S57

I. Experimental Section

Part 1. General Information

Commercial reagents were purchased from Adamas, Bide, Aldrich, Leyan, TCI and Energy Chemical. $NiBr_2$(dtbbpy),[1] $NiCl_2(Py)_4$,[2] Katritzky pyridinium salts[3] and CEBO (2-chloro 3-ethylbenzoxazolium tetrafluoroborate)[4] was prepared according to literature procedures. THF was distilled under a nitrogen atmosphere from sodium-benzophenone. Column chromatography was performed using silica gel 200-300 mesh (purchased from Qingdao-Haiyang Co., China) as the solid support. All NMR spectra were recorded on JEOL (400 MHz) spectrometers and Bruker Avance (600 MHz) spectrometer at STP. Data for ^{1}H NMR spectra are reported as follows: chemical shift (δ ppm), multiplicity, coupling constant (Hz) and integration. NMR spectra are internally referenced to residual proton solvent signals (note: $CDCl_3$ referenced at 7.26 ppm for ^{1}H NMR and 77.0 ppm for ^{13}C NMR). Coupling constants were reported in Hz, and multiplicity was indicated as follows: s (singlet); d (doublet); t (triplet); q (quartet); quint (quintet); m (multiplet); dd (doublet of doublets); ddd (doublet of doublet of doublets); dt (doublet of triplets); td (triplet of doublets). GC chromatograms were recorded on a GCMS-QP2010 SE (SHIMADZU) using an Agilent column CP7502 and Rxi-5 ms (Restek).

Part 2. Optimization experiments

1. ***General procedure of optimization experiments***: To a flame-dried tube equipped with a stir bar was added the appropriate ligand, B_2Pin_2 and methyl 4-bromobenzoate (21.5 mg, 0.1 mmol, 1.0 equiv). The tube was transferred into a N_2-filled glovebox, nickel catalyst, base and additives were added. The tube was sealed, removed from the glovebox and charged with the solvent. The mixture was stirred at 800 rpm, ensuring that the solid reagents were uniformly suspended. Then (3-bromopropyl)benzene (23.9 mg, 0.12 mmol, 1.2 equiv) was added to the tube with a syringe, the mixture was stirred vigorously (800 rpm) at 60 °C for 24 h. After that, the mixture was quenched with water (10 mL) and extracted with EtOAc (3 x 10 mL) and the combined organic layers were concentrated under reduced pressure. The residue was purified by column chromatography on silica

gel (eluted with 0-3% EtOAc/Petroleum ether) to give the target molecule, the yield was determined by ^{1}H NMR (using 2,5-dimethylfuran as the internal standard).

Table S1. Optimization of the reaction conditions

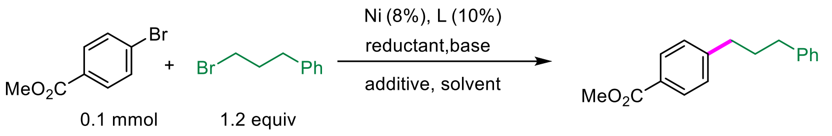

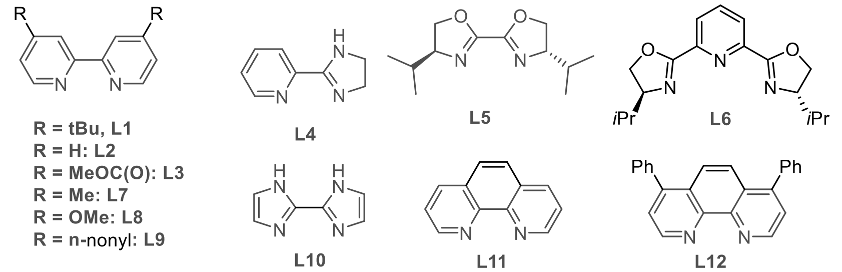

Entry	Ligand	Nickel catalyst	Solvent	Additive (equiv.)	Base	Reductant	T(°C)	Yield%[a]
1	L1	$NiBr_2$(DME)	DMA	NaI	K_2CO_3	B_2Pin_2	60	96
2	L2	$NiBr_2$(DME)	DMA	NaI	K_2CO_3	B_2Pin_2	60	91
3	L3	$NiBr_2$(DME)	DMA	NaI	K_2CO_3	B_2Pin_2	60	19
4	L4	$NiBr_2$(DME)	DMA	NaI	K_2CO_3	B_2Pin_2	60	88
5	L5	$NiBr_2$(DME)	DMA	NaI	K_2CO_3	B_2Pin_2	60	14
6	L6	$NiBr_2$(DME)	DMA	NaI	K_2CO_3	B_2Pin_2	60	32
7	L7	$NiBr_2$(DME)	DMA	NaI	K_2CO_3	B_2Pin_2	60	94
8	L8	$NiBr_2$(DME)	DMA	NaI	K_2CO_3	B_2Pin_2	60	84
9	L9	$NiBr_2$(DME)	DMA	NaI	K_2CO_3	B_2Pin_2	60	95
10	L10	$NiBr_2$(DME)	DMA	NaI	K_2CO_3	B_2Pin_2	60	19
11	L11	$NiBr_2$(DME)	DMA	NaI	K_2CO_3	B_2Pin_2	60	89
12	L12	$NiBr_2$(DME)	DMA	NaI	K_2CO_3	B_2Pin_2	60	75
13	-	$NiBr_2$(DME)	DMA	NaI	K_2CO_3	B_2Pin_2	60	ND
14	L2	$NiCl_2$(DME)	DMA	NaI	K_2CO_3	B_2Pin_2	60	94
15	L2	$Ni(COD)_2$	DMA	NaI	K_2CO_3	B_2Pin_2	60	80
16	L2	$Ni(ClO4)_2$	DMA	NaI	K_2CO_3	B_2Pin_2	60	78
17	L2	$NI(acac)_2$	DMA	NaI	K_2CO_3	B_2Pin_2	60	Trace
18	L2	$Ni(OTf)_2$	DMA	NaI	K_2CO_3	B_2Pin_2	60	81
19	L2	$NICl_2(Py)_4$	DMA	NaI	K_2CO_3	B_2Pin_2	60	86
20	L2	$NiBr_2$	DMA	NaI	K_2CO_3	B_2Pin_2	60	90
21	L2	NiI_2	DMA	NaI	K_2CO_3	B_2Pin_2	60	89
22	-	$NiBr_2$(dtbbpy)	DMA	NaI	K_2CO_3	B_2Pin_2	60	97(95)[b]

23	-	$NiBr_2$(dtbbpy)	NMP	NaI	K_2CO_3	B_2Pin_2	60	91
24	-	$NiBr_2$(dtbbpy)	DMF	NaI	K_2CO_3	B_2Pin_2	60	88
25	-	$NiBr_2$(dtbbpy)	Dioxane	NaI	K_2CO_3	B_2Pin_2	60	35
26	-	$NiBr_2$(dtbbpy)	DME	NaI	K_2CO_3	B_2Pin_2	60	55
27	-	$NiBr_2$(dtbbpy)	MeCN	NaI	K_2CO_3	B_2Pin_2	60	78
28	-	$NiBr_2$(dtbbpy)	DMSO	NaI	K_2CO_3	B_2Pin_2	60	79
29	-	$NiBr_2$(dtbbpy)	EtOAc	NaI	K_2CO_3	B_2Pin_2	60	26
30	-	$NiBr_2$(dtbbpy)	$PhCF_3$	NaI	K_2CO_3	B_2Pin_2	60	12
31	-	$NiBr_2$(dtbbpy)	DMA (1 mL)	NaI	K_2CO_3	B_2Pin_2	60	96
32	-	$NiBr_2$(dtbbpy)	DMA (0.2 mL)	NaI	K_2CO_3	B_2Pin_2	60	93
33	-	$NiBr_2$(dtbbpy)	DMA	KI	K_2CO_3	B_2Pin_2	60	95
34	-	$NiBr_2$(dtbbpy)	DMA	LiI	K_2CO_3	B_2Pin_2	60	96
35	-	$NiBr_2$(dtbbpy)	DMA	NaBr	K_2CO_3	B_2Pin_2	60	35
36	-	$NiBr_2$(dtbbpy)	DMA	TBAI	K_2CO_3	B_2Pin_2	60	95
37	-	$NiBr_2$(dtbbpy)	DMA	TBAB	K_2CO_3	B_2Pin_2	60	27
38	-	$NiBr_2$(dtbbpy)	DMA	NaI (1 eq)	K_2CO_3	B_2Pin_2	60	97
39	-	$NiBr_2$(dtbbpy)	DMA	-	K_2CO_{33}	B_2Pin_2	60	82
40	-	$NiBr_2$(dtbbpy)	DMA	NaI	Li_2CO_3	B_2Pin_2	60	27
41	-	$NiBr_2$(dtbbpy)	DMA	NaI	K_3PO_4	B_2Pin_2	60	54
42	-	$NiBr_2$(dtbbpy)	DMA	NaI	AcOK	B_2Pin_2	60	8
43	-	$NiBr_2$(dtbbpy)	DMA	NaI	LiOMe	B_2Pin_2	60	54
44	-	$NiBr_2$(dtbbpy)	DMA	NaI	*t*BuOK	B_2Pin_2	60	32
45	-	$NiBr_2$(dtbbpy)	DMA	NaI	K_2HPO_4	B_2Pin_2	60	39
46	-	$NiBr_2$(dtbbpy)	DMA	NaI	DIPEA	B_2Pin_2	60	18
47	-	$NiBr_2$(dtbbpy)	DMA	NaI	NaOH	B_2Pin_2	60	31
48	-	$NiBr_2$(dtbbpy)	DMA	NaI	-	B_2Pin_2	60	ND
49	-	$NiBr_2$(dtbbpy)	DMA	NaI	K_2CO_3	B2	60	97
50	-	$NiBr_2$(dtbbpy)	DMA	NaI	K_2CO_3	B3	60	69
51	-	$NiBr_2$(dtbbpy)	DMA	NaI	K_2CO_3	B4	60	26
52	-	$NiBr_2$(dtbbpy)	DMA	NaI	K_2CO_3	B5	60	41
53	-	$NiBr_2$(dtbbpy)	DMA	NaI	K_2CO_3	B6	60	34
54	-	$NiBr_2$(dtbbpy)	DMA	NaI	K_2CO_3	B_2Pin_2 (1 eq)	60	86
55	-	$NiBr_2$(dtbbpy)	DMA	NaI	K_2CO_3	B_2Pin_2 (2 eq)	60	96
56	-	$NiBr_2$(dtbpy)	DMA	NaI	K_2CO_3	-	60	ND
57	-	$NiBr_2$(dtbbpy)	DMA	NaI	K_2CO_3	B_2Pin_2	80	90
58	-	$NiBr_2$(dtbbpy)	DMA	NaI	K_2CO_3	B_2Pin_2	40	92

59	-	$NiBr_2$(dtbbpy)	DMA	NaI	K_2CO_3	B_2Pin_2	25	85

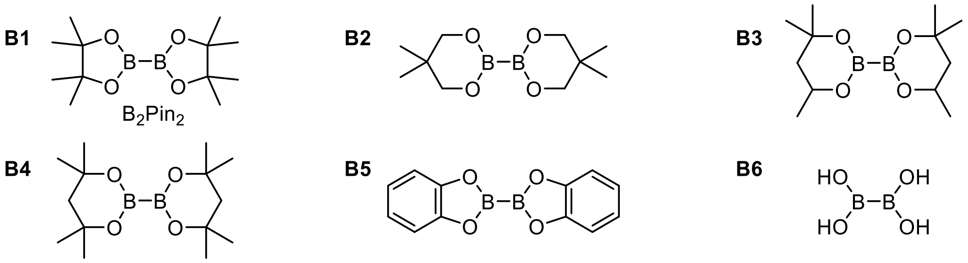

[a] NMR yield using 2,5-dimethylfuran as the internal standard. [b] Isolated yield. [c] Not detected.

2. **Initial trial for the reaction of S1 and S2 using the method developed for alkyl-alkyl coupling:**

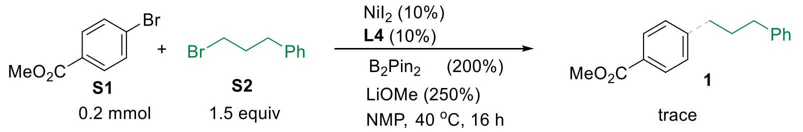

(eq S1)

Part 3. Cross-Coupling Reactions and Product Characterization

Standard method A for the coupling of electron deficient aryl bromides with primary alkyl bromides*:* To a flame-dried tube with a stir bar was added aryl halide (0.1 mmol, 1.0 equiv), $NiBr_2$(dtbbpy) (4 mg, 8 mol %). The tube was transferred into an N_2-filled glovebox, B_2pin_2 (38 mg, 0.15 mmol, 1.5 equiv), NaI (7.5 mg, 0.05 mmol, 0.5 equiv) and K_2CO_3 (25 mg, 0.18 mmol, 1.8 equiv) were added. The tube was sealed, removed from the glovebox and charged with DMA (0.5 mL). The mixture was stirred vigorously, alkyl bromide (0.12 mmol, 1.2 equiv) was added to the tube. The mixture was stirred vigorously at 60 °C for 24 h. After that, the reaction mixture was quenched with water (10 mL), extracted with EtOAc (3 x 15 mL). The combined organic layers were washed with brine (2 x 10 mL), dried with $MgSO_4$, filtered, and concentrated under reduced pressure. The residue was purified by chromatography on silica gel, eluting with ethyl acetate/petroleum ether to give the target molecular.

Note:

(1) for the coupling of heteroaryl bromides with primary alkyl halides**,** a similar procedure to the standard method A was used, except that dtbbpy (2.7 mg, 10 mol %) and $NiCl_2(Py)_4$ (3.6 mg, 8 mol %) displaced $NiBr_2$(dtbbpy**)**, and NaI (30 mg, 2 equiv), TBAI (18 mg, 0.5 equiv) were employed.

(2) for the coupling of electron rich aryl iodides with primary alkyl bromides, a similar procedure to the standard method A was used except that NaI (30 mg, 2 equiv), TBAI (18 mg, 0.5 equiv) and alkyl bromide (1.5 equiv) were employed.

(3) for the coupling of aryl halides with secondary alkyl bromides, a similar procedure to the standard method A was used, except that $Ni(OTf)_2$ (4 mg, 8 mol %), dtbbpy (2.7 mg, 10 mol %) displaced $NiBr_2$(dtbbpy**)**, and NaI (30 mg, 2 equiv), TBAI (18 mg, 0.5 equiv) and alkyl bromide (1.5 equiv) were employed.

(4) for the coupling of alkyl-pyridinium salts, a similar procedure to the standard method A was used, except NaI (18 mg, 1 equiv), and alkyl-pyridinium salt (1.5 equiv) were employed.

Method C for the coupling of alcohols with aryl halides: In a N_2 filled glove box, a mixture of a secondary alcohol (0.15 mmol, 150 mol%), 2-chloro-3-ethylbenzoxazolium tetrafluoroborate (CEBO) (40.4 mg, 0.15 mmol, 150 mol%) and Bu_4NBr (48.4 mg, 0.15 mmol, 150 mol%) in MeCN (0.2 mL) was stirred in a Schlenk tube for 5 minutes (unless otherwise noted) at room temperature. To the reaction mixture, pyridine (12.1 μL, 0.15 mmol, 1.5 equiv), aryl halide (0.1 mmol, 1.0 equiv), $NiBr_2$(dtbbpy) (4 mg, 8 mol %), B_2pin_2 (38 mg, 0.15 mmol, 1.5 equiv), NaI (30 mg, 2mmol, 2 equiv), K_2CO_3 (25 mg, 0.18 mmol, 1.8 equiv) were added. The tube was sealed, removed from the glovebox and charged with DMA (0.4 mL). The mixture was stirred vigorously at 60 °C for 24 h. After that, the reaction mixture was quenched with water (10 mL), extracted with EtOAc (3 x 15 mL). The combined organic layers were washed with brine (2 x 10 mL), dried with $MgSO_4$, filtered, and concentrated under reduced pressure. The residue was purified by chromatography on silica gel, eluting with ethyl acetate/petroleum ether to give the target molecular.

Methyl 4-(3-phenylpropyl)benzoate (1a)[4]

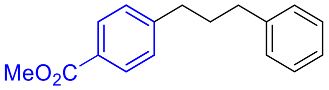

This compound was prepared according to method A using methyl 4-bromobenzoate and (3-bromopropyl)benzene. Purification by flash column chromatograph to afford **1a** as a colorless oil (24.1 mg, 95% yield).

^{1}H NMR (600 MHz, $CDCl_3$) δ 7.95 (d, *J* = 8.0 Hz, 2H), 7.28 (t, *J* = 7.5 Hz, 2H), 7.24 (s, 2H), 7.20 – 7.15 (m, 3H), 3.89 (s, 3H), 2.72 – 2.66 (m, 2H), 2.64 (t, *J* = 7.7 Hz, 2H), 1.96 (tt, *J* = 9.2, 6.7 Hz, 2H).

Gram-scale synthesis: To a dry 100 mL flask equipped with a teflon-coated magnetic stir bar was added 4-bromobenzoate (1.08 g, 5.0 mmol, 1.0 equiv), $NiBr_2$(dtbbpy) (98 mg, 4 mol %). The flask was transferred into an N_2-filled glovebox, B_2pin_2 (1.91 g, 7.5 mmol, 1.5 equiv), NaI (374 mg, 0.5 equiv) and K_2CO_3 (1.24 g, 1.8 equiv) were added. The flask was sealed, removed from the glovebox and charged with DMA (25 mL). The mixture was stirred vigorously, (3-bromopropyl)benzene (1.19 g, 1.2 equiv) was added to the flask. The mixture was stirred vigorously at 60 °C for 48 h (detected by GCMS). After that, the reaction mixture was quenched with water (100 mL), extracted with EtOAc (3 x 100 mL). The combined organic layers were washed with brine (3 x 50 mL), dried with $MgSO_4$, filtered, and concentrated under reduced pressure. The residue was purified by chromatography on silica gel, eluting with ethyl acetate/petroleum ether to give the target molecular **1a** (1.1g, 86%).

Methyl 4-dodecylbenzoate (1b)

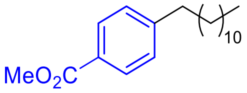

This compound was prepared according to method A using methyl 4-bromobenzoate and 1-bromododecane. Purification by flash column chromatograph to afford **1b** as a colorless oil (26.8 mg, 88% yield).

Ten Gram-scale synthesis: To a dry 250 mL flask equipped with a teflon-coated magnetic stir bar was added 4-bromobenzoate (10.75 g, 50.0 mmol, 1.0 equiv), $NiBr_2$(dtbbpy) (974 mg, 2.0 mmol, 4 mol %). The flask was transferred into an N_2-filled glovebox, B_2pin_2 (14.95 g, 60.0 mmol, 1.2 equiv), NaI (3.75 g, 25.0 mmol, 0.5 equiv) and K_2CO_3 (10.37 g, 75 mmol, 1.5 equiv) were added. The flask was sealed, removed from the glovebox and charged with DMA (125 mL). The mixture was stirred vigorously, 1-bromododecane (14.95 g,

60.0 mmol, 1.2 equiv) was added to the flask. The mixture was stirred vigorously at 60 °C for 48 h (detected by GCMS). After that, the reaction mixture was quenched with water (700 mL), extracted with EtOAc (4 x 300 mL). The combined organic layers were washed with brine (3 x 300 mL), dried with $MgSO_4$, filtered, and concentrated under reduced pressure. The residue was purified by chromatography on silica gel, eluting with ethyl acetate/petroleum ether to give the target molecular **1b** (12.8 g, 84%).

^{1}H NMR (600 MHz, $CDCl_3$) δ 7.94 (d, J = 8.0 Hz, 2H), 7.23 (d, J = 8.0 Hz, 2H), 3.90 (s, 3H), 2.65 (t, J = 7.8 Hz, 2H), 1.62 (t, J = 7.5 Hz, 2H), 1.31 – 1.24 (m, 18H), 0.88 (t, J = 7.0 Hz, 3H).

^{13}C NMR (151 MHz, $CDCl_3$) δ 167.3, 148.7, 129.8, 128.6, 127.7, 52.1, 36.2, 32.1, 31.3, 29.8, 29.8, 29.7, 29.6, 29.5, 29.4, 22.8, 14.3.

HRMS calcd for $C_{20}H_{33}O_2$ $[M+H]^+$ 305.2475, found 305.2463.

Methyl 3-(3-phenylpropyl)benzoate (2)[7]

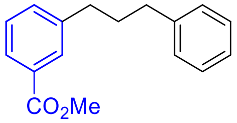

This compound was prepared according to method A using methyl 3-bromobenzoate and (3-bromopropyl)benzene. Purification by flash column chromatograph to afford **2** as a colorless oil (14 mg, 55% yield).

^{1}H NMR (600 MHz, $CDCl_3$) δ 7.91 – 7.83 (m, 2H), 7.40 – 7.33 (m, 2H), 7.31 – 7.26 (m, 2H), 7.19 (dd, J = 8.5, 6.9 Hz, 3H), 3.91 (s, 3H), 2.68 (dt, J = 26.3, 7.7 Hz, 4H), 1.98 (tt, J = 9.3, 6.8 Hz, 2H).

4-(3-phenylpropyl)benzonitrile (3)[7]

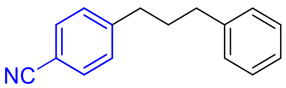

This compound was prepared according to method A using methyl 4-bromobenzonitrile and (3-bromopropyl)benzene. Purification by flash column chromatograph to afford **3** as a colorless oil (19.9 mg, 94% yield).

^{1}H NMR (600 MHz, $CDCl_3$) δ 7.58 (d, J = 8.0 Hz, 2H), 7.33 – 7.26 (m, 4H), 7.24 – 7.15 (m, 3H), 2.68 (dt, J = 28.1, 7.7 Hz, 4H), 1.97 (tt, J = 9.4, 6.8 Hz, 2H).

^{13}C NMR (151 MHz, $CDCl_3$) δ 148.1, 141.7, 132.3, 129.3, 128.6, 128.5, 126.1, 119.3, 109.8, 35.6, 35.4, 32.6.

2-(3-Phenylpropyl)benzonitrile (4)[8]

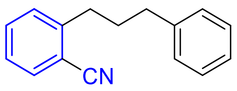

This compound was prepared according to method A using 2-bromobenzonitrile and (3-bromopropyl)benzene. Purification by flash column chromatograph to afford **4** as a colorless oil (17.0 mg, 77% yield).

^{1}H NMR (600 MHz, $CDCl_3$) δ 7.62 (dd, *J* = 7.7, 1.3 Hz, 1H), 7.50 (td, *J* = 7.7, 1.4 Hz, 1H), 7.36 – 7.27 (m, 4H), 7.25 – 7.17 (m, 3H), 2.95 – 2.86 (m, 2H), 2.71 (t, *J* = 7.8 Hz, 2H), 2.06 – 1.95 (m, 2H).

^{13}C NMR (151 MHz, $CDCl_3$) δ 146.4, 141.7, 133.0, 132.9, 129.6, 128.5, 126.6, 126.1, 118.2, 112.5, 35.6, 34.3, 32.6.

Diethyl (4-(3-phenylpropyl)phenyl)phosphonate (5)

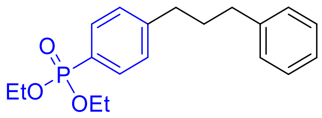

This compound was prepared according to method A using diethyl (4-bromophenyl)phosphonate and (3-bromopropyl)benzene. Purification by flash column chromatograph to afford **5** as a colorless oil (29.9 mg, 90% yield).

^{1}H NMR (600 MHz, $CDCl_3$) δ 7.72 (dd, *J* = 13.1, 7.8 Hz, 2H), 7.33 – 7.25 (m, 5H), 7.21 – 7.15 (m, 3H), 4.18 – 4.03 (m, 4H), 2.67 (dt, *J* = 22.4, 7.7 Hz, 4H), 2.02 – 1.94 (m, 2H), 1.32 (t, *J* = 7.1 Hz, 6H).

^{13}C NMR (151 MHz, $CDCl_3$) δ 147.3, 142.0, 132.1, 132.0, 128.8, 128.7, 128.5, 128.5, 62.1, 35.6, 35.5, 32.7, 16.5.

HRMS calcd for $C_{19}H_{26}O_3P$ $[M+H]^+$ 333.1614, found 333.1616.

4-(3-Phenylpropyl)benzaldehyde (6)[4]

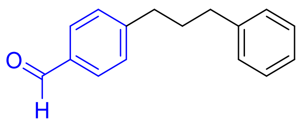

This compound was prepared according to method A using diethyl 4-bromobenzaldehyde and (3-bromopropyl)benzene. Purification by flash column chromatograph to afford **6** as a colorless oil (19.9 mg, 89% yield).

^{1}H NMR (600 MHz, $CDCl_3$) δ 9.97 (s, 1H), 7.80 (d, *J* = 7.9 Hz, 2H), 7.34 (d, *J* = 7.8 Hz, 2H), 7.28 (d, *J* = 7.4 Hz, 2H), 7.21 – 7.16 (m, 3H), 2.75 – 2.70 (m, 2H), 2.66 (t, *J* = 7.7 Hz, 2H), 1.99 (tt, *J* = 9.4, 6.7 Hz, 2H).

Isopropyl 2-methyl-2-(4-(4-(3-phenylpropyl)benzoyl)phenoxy)propanoate (7)

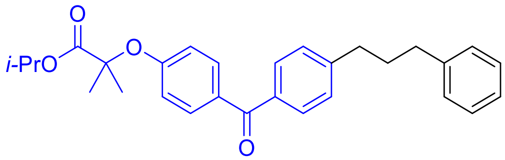

This compound was prepared according to method A using diethyl isopropyl 2-(4-(4-bromobenzoyl)phenoxy)-2-methylpropanoate and (3-bromopropyl)benzene. Purification by flash column chromatograph to afford **7** as a colorless oil (40.9 mg, 92% yield).

^{1}H NMR (600 MHz, CDCl$_3$) δ 7.78 – 7.73 (m, 2H), 7.71 – 7.66 (m, 2H), 7.32 – 7.25 (m, 4H), 7.22 – 7.17 (m, 3H), 6.88 – 6.83 (m, 2H), 5.09 (hept, J = 6.3 Hz, 1H), 2.70 (dt, J = 30.0, 7.7 Hz, 4H), 2.10 – 1.93 (m, 2H), 1.66 (s, 6H), 1.20 (d, J = 6.3 Hz, 6H).

^{13}C NMR (151 MHz, CDCl$_3$) δ 195.5, 173.3, 159.5, 147.1, 142.1, 135.9, 132.1, 131.0, 130.2, 128.5, 128.5, 128.4, 126.0, 117.3, 79.5, 69.4, 35.5, 35.5, 32.8, 25.5, 21.6.

HRMS calcd for $C_{29}H_{32}NaO_4$ $[M+Na]^+$ 467.2193, found 467.2182.

8-(3-Phenylpropyl)quinoline (8)[9]

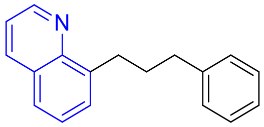

This compound was prepared according to method A (for heteroaryl halides) using 8-bromoquinoline and (3-bromopropyl)benzene. Purification by flash column chromatograph to afford **8** as a pale yellow oil (14.1 mg, 57% yield).

^{1}H NMR (600 MHz, CDCl$_3$) δ 8.95 (dd, J = 4.3, 1.7 Hz, 1H), 8.14 (dd, J = 8.3, 1.8 Hz, 1H), 7.68 (d, J = 8.0 Hz, 1H), 7.56 (d, J = 7.0 Hz, 1H), 7.47 (t, J = 7.6 Hz, 1H), 7.40 (dd, J = 8.2, 4.1 Hz, 1H), 7.28 (t, J = 7.5 Hz, 2H), 7.23 (s, 2H), 7.17 (t, J = 7.3 Hz, 1H), 3.36 (t, J = 7.8 Hz, 2H), 2.81 – 2.74 (m, 2H), 2.15 (tt, J = 9.8, 6.7 Hz, 2H).

4-(3-Phenylpropyl)quinoline (9)[10]

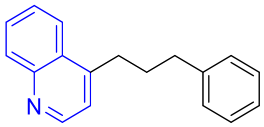

This compound was prepared according to method A (for heteroaryl halides) using 4-bromoquinoline and (3-bromopropyl)benzene. Purification by flash column chromatograph to afford **9** as a pale yellow oil (18.1 mg, 73% yield).

^{1}H NMR (600 MHz, $CDCl_3$) δ 8.80 (d, *J* = 4.4 Hz, 1H), 8.16 – 8.07 (m, 1H), 7.95 (dd, *J* = 8.5, 1.4 Hz, 1H), 7.70 (ddd, *J* = 8.4, 6.8, 1.4 Hz, 1H), 7.54 (ddd, *J* = 8.3, 6.8, 1.3 Hz, 1H), 7.31 (t, *J* = 7.6 Hz, 2H), 7.22 (ddd, *J* = 11.1, 7.1, 3.6 Hz, 4H), 3.17 – 3.05 (m, 2H), 2.77 (t, *J* = 7.6 Hz, 2H), 2.12 (tt, *J* = 9.6, 6.9 Hz, 2H).

^{13}C NMR (151 MHz, $CDCl_3$) δ 150.3, 148.4, 148.4, 141.7, 130.3, 129.2, 128.6, 127.7, 126.4, 126.2, 123.6, 120.9, 35.8, 31.7, 31.6.

3-(3-Phenylpropyl)quinoline (10)[11]

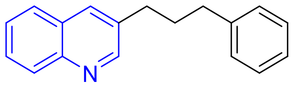

This compound was prepared according to method A (for heteroaryl halides) using 3-bromoquinoline and (3-bromopropyl)benzene. Purification by flash column chromatograph to afford **10** as a pale yellow oil (22.7 mg, 92% yield).

^{1}H NMR (600 MHz, $CDCl_3$) δ 8.79 (d, *J* = 2.2 Hz, 1H), 8.09 (d, *J* = 8.4 Hz, 1H), 7.92 (d, *J* = 2.1 Hz, 1H), 7.77 (dd, *J* = 8.2, 1.4 Hz, 1H), 7.66 (ddd, *J* = 8.4, 6.8, 1.4 Hz, 1H), 7.53 (td, *J* = 7.5, 6.8, 1.3 Hz, 1H), 7.31 (dd, *J* = 8.7, 6.7 Hz, 2H), 7.24 – 7.17 (m, 3H), 2.84 (t, *J* = 7.8 Hz, 2H), 2.72 (t, *J* = 7.7 Hz, 2H), 2.11 – 2.04 (m, 2H).

^{13}C NMR (151 MHz, $CDCl_3$) δ 152.1, 146.9, 141.8, 134.9, 134.4, 129.3, 128.7, 128.6, 128.6, 127.4, 128.3, 126.7, 126.1, 35.4, 32.7, 32.7.

2-(3-Phenylpropyl)quinoline (11)[10]

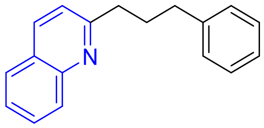

This compound was prepared according to method A (for heteroaryl halides) using 2-bromoquinoline and (3-bromopropyl)benzene. Purification by flash column chromatograph to afford **11** as a pale yellow oil (16.3 mg, 66% yield).

^{1}H NMR (600 MHz, $CDCl_3$) δ^{1}H NMR (600 MHz, Chloroform-*d*) δ 8.06 (dd, *J* = 10.9, 8.5 Hz, 2H), 7.78 (d, *J* = 8.1 Hz, 1H), 7.69 (ddd, *J* = 8.4, 6.8, 1.4 Hz, 1H), 7.49 (t, *J* = 7.5 Hz, 1H), 7.28 (dt, *J* = 7.5, 3.4 Hz, 3H), 7.24 – 7.15 (m, 3H), 3.06 – 2.98 (m, 2H), 2.74 (t, *J* = 7.8 Hz, 2H), 2.21 – 2.11 (m, 2H).

4-(3-Phenylpropyl)pyridine (12)[11]

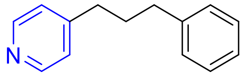

This compound was prepared according to method A (for heteroaryl halides) using 4-bromopyridine and (3-bromopropyl)benzene. Purification by flash column chromatograph to afford **12** as a pale yellow oil (14.8 mg, 75% yield)

^{1}H NMR (600 MHz, $CDCl_3$) δ^{1}H NMR (600 MHz, Chloroform-*d*) δ 8.54 – 8.42 (m, 2H), 7.29 (t, *J* = 7.5 Hz, 2H), 7.23 – 7.14 (m, 3H), 7.14 – 7.06 (m, 2H), 2.65 (dt, *J* = 10.8, 7.7 Hz, 4H), 1.97 (tt, *J* = 9.3, 6.8 Hz, 2H).

^{13}C NMR (151 MHz, $CDCl_3$) δ 151.4, 149.7, 141.7, 128.5, 128.5, 126.1, 124.1, 35.4, 34.7, 31.9.

.

4-(3-Phenylpropyl)-2-(trifluoromethyl)pyridine (13)

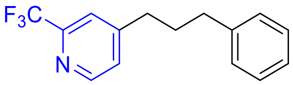

This compound was prepared according to t method A (for heteroaryl halides) using 4-bromo-2-(trifluoromethyl)pyridine and (3-bromopropyl)benzene. Purification by flash column chromatograph to afford **13** as a pale yellow oil (13.5 mg, 51% yield).

^{1}H NMR (600 MHz, $CDCl_3$) δ 8.61 (d, *J* = 5.0 Hz, 1H), 7.50 (d, *J* = 1.6 Hz, 1H), 7.30 (dd, *J* = 8.9, 6.3 Hz, 3H), 7.24 – 7.20 (m, 1H), 7.19 – 7.15 (m, 2H), 2.71 (dt, *J* = 26.0, 7.7 Hz, 4H), 2.01 (tt, *J* = 9.4, 6.9 Hz, 2H).

^{13}C NMR (151 MHz, $CDCl_3$) δ 153.2, 149.8, 148.2 (q, *J* = 34.2 Hz), 141.1, 128.5, 128.4, 126.4, 126.1, 121.6 (q, *J* = 274.2 Hz), 120.5 (q, *J* = 2.6 Hz), 35.2, 34.6, 31.6.

HRMS calcd for $C_{15}H_{15}F_3N$ $[M+H]^+$ 266.1151, found 266.1148.

3-(3-Phenylpropyl)pyridine (14)[11]

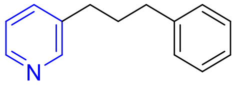

This compound was prepared according to method A (for heteroaryl halides) using 3-bromopyridine and (3-bromopropyl)benzene. Purification by flash column chromatograph to afford **14** as a pale yellow oil (14.0 mg, 71% yield).

^{1}H NMR (600 MHz, $CDCl_3$) δ 8.45 (s, 2H), 7.50 (d, *J* = 7.6 Hz, 1H), 7.29 (t, *J* = 7.5 Hz, 2H), 7.19 (dd, *J* = 14.1, 7.4 Hz, 4H), 2.66 (q, *J* = 7.8 Hz, 4H), 1.97 (p, *J* = 7.7 Hz, 2H).

^{13}C NMR (151 MHz, $CDCl_3$) δ 150.0, 147.4, 141.8, 137.5, 135.9, 128.5, 126.0, 123.4, 35.4, 32.7, 32.6.

2-fluoro-5-(3-Phenylpropyl)pyridine (15)[12]

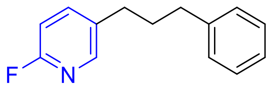

This compound was prepared according to method A (for heteroaryl halides) using 5-bromo-2-fluoropyridine and (3-bromopropyl)benzene. Purification by flash column chromatograph to afford **15** a colorless oil (11.2 mg, 52% yield).

^{1}H NMR (600 MHz, CDCl$_3$) δ 8.05 – 7.97 (m, 1H), 7.59 (td, *J* = 8.1, 2.5 Hz, 1H), 7.30 (t, *J* = 7.5 Hz, 2H), 7.23 – 7.14 (m, 3H), 6.85 (dd, *J* = 8.4, 2.9 Hz, 1H), 2.65 (dt, *J* = 14.8, 7.7 Hz, 4H), 1.95 (tt, *J* = 9.1, 6.9 Hz, 2H).

4-(5-(3-Phenylpropyl)pyridin-2-yl)morpholine (16)

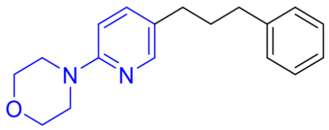

This compound was prepared according to method A (for heteroaryl halides) using 4-(5-bromopyridin-2-yl)morpholine and (3-bromopropyl)benzene. Purification by flash column chromatograph to afford **16** as a pale yellow oil (24.8 mg, 88% yield).

^{1}H NMR (600 MHz, CDCl$_3$) δ 8.05 (d, *J* = 2.3 Hz, 1H), 7.35 (dd, *J* = 8.6, 2.4 Hz, 1H), 7.28 (t, *J* = 7.5 Hz, 2H), 7.22 – 7.12 (m, 3H), 6.60 (d, *J* = 8.6 Hz, 1H), 3.89 – 3.77 (m, 4H), 3.49 – 3.38 (m, 4H), 2.63 (t, *J* = 7.7 Hz, 2H), 2.53 (t, *J* = 7.6 Hz, 2H), 1.90 (tt, *J* = 9.1, 6.8 Hz, 2H).

^{13}C NMR (151 MHz, CDCl$_3$) δ 158.3, 147.4, 142.2, 138.1, 128.5, 128.5, 127.5, 125.9, 107.1, 66.9, 46.2, 35.3, 33.0, 31.6.

HRMS calcd for $C_{18}H_{23}N_2O$ $[M+H]^+$ 283.1805, found 283.1804

2-(3-Phenylpropyl)pyridine (17)[11]

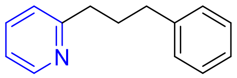

This compound was prepared according to method A (for heteroaryl halides) using 2-bromopyridine and (3-bromopropyl)benzene. Purification by flash column chromatograph to afford **17** as a colorless oil (16.2 mg, 82% yield).

^{1}H NMR (600 MHz, CDCl$_3$) δ 8.53 (dd, *J* = 5.0, 1.7 Hz, 1H), 7.57 (td, *J* = 7.7, 1.8 Hz, 1H), 7.27 (t, *J* = 7.6 Hz, 2H), 7.18 (dd, *J* = 15.6, 7.5 Hz, 3H), 7.13 (d, *J* = 7.8 Hz, 1H), 7.09 (dd, *J* = 7.5, 4.9 Hz, 1H), 2.86 – 2.79 (m, 2H), 2.68 (t, *J* = 7.8 Hz, 2H), 2.07 (tt, *J* = 9.7, 6.7 Hz, 2H).

^{13}C NMR (151 MHz, CDCl$_3$) δ 162.1, 149.3, 142.2, 136.4, 128.6, 128.4, 125.9, 122.9, 121.1, 38.0, 35.7, 31.6.

2-Methyl-6-(3-phenylpropyl)pyridine (18)[11]

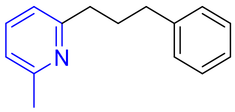

This compound was prepared according to method A (for heteroaryl halides) using 2-bromo-6-methylpyridine and (3-bromopropyl)benzene. Purification by flash column chromatograph to afford **18** as a colorless oil (13.9 mg, 66% yield).

^{1}H NMR (600 MHz, $CDCl_3$) δ 7.47 (t, *J* = 7.6 Hz, 1H), 7.31 – 7.26 (m, 2H), 7.22 – 7.15 (m, 3H), 6.95 (dd, *J* = 13.4, 7.6 Hz, 2H), 2.83 – 2.77 (m, 2H), 2.71 – 2.66 (m, 2H), 2.53 (s, 3H), 2.07 – 2.02 (m, 2H).

^{13}C NMR (151 MHz, $CDCl_3$) δ 161.5, 157.9, 142.4, 136.7, 128.6, 128.4, 125.9, 120.6, 119.7, 38.2, 35.8, 32.0, 24.7.

5-Methyl-2-(3-phenylpropyl)pyridine (19)

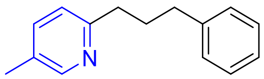

This compound was prepared according to method A (for heteroaryl halides) using 2-bromo-5-methylpyridine and (3-bromopropyl)benzene. Purification by flash column chromatograph to afford **19** as a pale yellow oil (16.5 mg, 78% yield).

^{1}H NMR (600 MHz, $CDCl_3$) δ 8.35 (d, *J* = 2.2 Hz, 1H), 7.39 (dd, *J* = 7.9, 2.3 Hz, 1H), 7.28 (d, *J* = 7.6 Hz, 2H), 7.22 – 7.15 (m, 3H), 7.03 (d, *J* = 7.9 Hz, 1H), 2.83 – 2.76 (m, 2H), 2.71 – 2.64 (m, 2H), 2.29 (s, 3H), 2.08 – 2.01 (m, 2H).

^{13}C NMR (151 MHz, $CDCl_3$) δ 159.0, 149.7, 142.2, 137.1, 130.3, 128.6, 128.4, 125.9, 122.4, 37.5, 35.7, 31.7, 18.2.

HRMS calcd for $C_{15}H_{18}N$ $[M+H]^+$ 212.1434, found 212.1430

5-Phenyl-2-(3-phenylpropyl)pyridine (20)

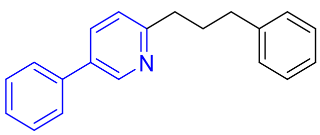

This compound was prepared according to method A (for heteroaryl halides) using 2-bromo-5-phenylpyridine and (3-bromopropyl)benzene. Purification by flash column chromatograph to afford **20** as a pale yellow solid (24.6 mg, 90% yield).

^{1}H NMR (600 MHz, CDCl$_3$) δ 8.77 (d, *J* = 2.3 Hz, 1H), 7.79 (dd, *J* = 8.0, 2.4 Hz, 1H), 7.60 – 7.55 (m, 2H), 7.47 (t, *J* = 7.6 Hz, 2H), 7.39 (t, *J* = 7.4 Hz, 1H), 7.29 (t, *J* = 7.6 Hz, 2H), 7.25 – 7.17 (m, 4H), 2.92 – 2.85 (m, 2H), 2.73 (t, *J* = 7.7 Hz, 2H), 2.12 (tt, *J* = 9.4, 6.9 Hz, 2H).

^{13}C NMR (151 MHz, CDCl$_3$) δ 160.9, 147.8, 142.2, 138.1, 134.9, 134.1, 129.1, 128.6, 128.5, 127.9, 127.1, 125.9, 122.8, 37.6, 35.7, 31.6.

HRMS calcd for $C_{20}H_{20}N$ $[M+H]^+$ 274.1588, found 274.1590.

4-Methyl-2-(3-phenylpropyl)pyridine (21)[12]

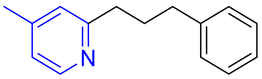

This compound was prepared according to method A (for heteroaryl halides) using 2-bromo-4-methylpyridine and (3-bromopropyl)benzene. Purification by flash column chromatograph to afford **21** as a colorless oil (19.0 mg, 91% yield).

^{1}H NMR (600 MHz, CDCl$_3$) δ 8.38 (d, *J* = 5.0 Hz, 1H), 7.27 (t, *J* = 7.5 Hz, 2H), 7.22 – 7.14 (m, 3H), 6.95 (s, 1H), 6.92 (d, *J* = 5.1 Hz, 1H), 2.82 – 2.74 (m, 2H), 2.68 (t, *J* = 7.8 Hz, 2H), 2.09 – 2.02 (m, 2H).

^{13}C NMR (151 MHz, CDCl$_3$) δ 161.8, 149.1, 147.4, 142.3, 128.6, 128.4, 125.9, 123.8, 122.2, 37.9, 35.7, 31.6, 21.1.

2-(3-Phenylpropyl)-1*H*-indene (22)[13]

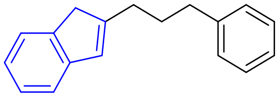

This compound was prepared according to method A (for heteroaryl halides) using 2-bromo-1*H*-indene and (3-bromopropyl)benzene. Purification by flash column chromatograph to afford **22** as a colorless oil (13.1 mg, 56% yield).

^{1}H NMR (600 MHz, CDCl$_3$) δ 7.38 (d, *J* = 7.4 Hz, 1H), 7.31 – 7.26 (m, 3H), 7.21 (t, *J* = 7.0 Hz, 4H), 7.10 (td, *J* = 7.4, 1.2 Hz, 1H), 6.53 (t, *J* = 1.7 Hz, 1H), 3.31 (s, 2H), 2.68 (t, *J* = 7.7 Hz, 2H), 2.52 (t, *J* = 7.6 Hz, 2H), 1.95 (p, *J* = 7.7 Hz, 2H).

(*E*)-1,2-Dimethoxy-4-(5-phenylpent-1-en-1-yl)benzene (23)

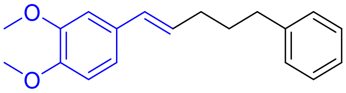

This compound was prepared according to method A (for heteroaryl halides) using (*E*)-4-(2-bromovinyl)-1,2-dimethoxybenzene and (3-

bromopropyl)benzene. Purification by flash column chromatograph to afford **23** as colorless oil (15.5 mg, 55% yield).

^{1}H NMR (600 MHz, CDCl$_3$) δ 7.32 – 7.27 (m, 2H), 7.23 – 7.17 (m, 3H), 6.91 (d, *J* = 2.0 Hz, 1H), 6.88 (dd, *J* = 8.2, 2.0 Hz, 1H), 6.81 (d, *J* = 8.2 Hz, 1H), 6.34 (dt, *J* = 15.8, 1.5 Hz, 1H), 6.11 (dt, *J* = 15.7, 6.9 Hz, 1H), 3.90 (s, 3H), 3.88 (s, 3H), 2.72 – 2.64 (m, 2H), 2.29 – 2.21 (m, 2H), 1.85 – 1.77 (m, 2H).

^{13}C NMR (151 MHz, CDCl$_3$) δ 149.1, 148.4, 142.5, 131.1, 129.9, 128.8, 128.6, 128.4, 125.8, 118.9, 111.3, 108.6, 56.1, 55.9, 35.6, 32.6, 31.3.

HRMS calcd for $C_{19}H_{23}O_2$ $[M+H]^+$ 283.1693, found 283.1700.

1-Methoxy-4-(3-phenylpropyl)benzene (24)[4]

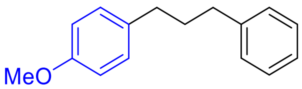

This compound was prepared according to method A (for electron-rich aryl iodides) using 1-iodo-4-methoxybenzene and (3-bromopropyl)benzene. Purification by flash column chromatograph to afford **24** as a colorless oil (17.7 mg, 78% yield).

^{1}H NMR (600 MHz, CDCl$_3$) δ 7.29 (t, *J* = 7.5 Hz, 2H), 7.22 – 7.16 (m, 3H), 7.14 – 7.08 (m, 2H), 6.91 – 6.78 (m, 2H), 3.80 (s, 3H), 2.63 (dt, *J* = 25.5, 7.7 Hz, 4H), 1.98 – 1.89 (m, 2H).

Methyl(4-(3-phenylpropyl)phenyl)sulfane (25)

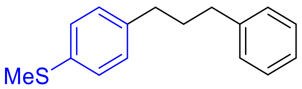

This compound was prepared according to method A (for electron-rich aryl iodides) using (4-iodophenyl)(methyl)sulfane and (3-bromopropyl)benzene. Purification by flash column chromatograph to afford **25** as a colorless oil (11.4 mg, 80% yield).

^{1}H NMR (600 MHz, CDCl$_3$) δ 7.32 – 7.27 (m, 2H), 7.23 – 7.16 (m, 5H), 7.12 (d, *J* = 8.2 Hz, 2H), 2.64 (dt, *J* = 17.4, 7.7 Hz, 4H), 2.48 (s, 3H), 1.99 – 1.90 (m, 2H).

^{13}C NMR (151 MHz, CDCl$_3$) δ 142.3, 139.6, 135.3, 129.1, 128.6, 128.4, 127.3, 125.9, 35.5, 35.0, 33.0, 16.5.

HRMS calcd for $C_{16}H_{19}S$ $[M+H]^+$ 243.1202, found 243.1188.

1-(4-(3-Phenylpropyl)phenyl)piperidin-2-one (26)

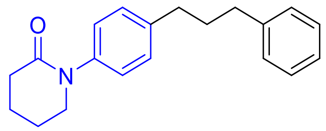

This compound was prepared according to method A (for electron-rich aryl iodides) using 1-(4-iodophenyl)piperidin-2-one and (3-bromopropyl)benzene. Purification by flash column chromatograph to afford **26** as a colorless oil (15.0 mg, 51% yield).

^{1}H NMR (600 MHz, CDCl$_3$) δ 7.28 (dd, *J* = 8.3, 6.9 Hz, 2H), 7.23 – 7.17 (m, 5H), 7.17 – 7.13 (m, 2H), 3.62 (td, *J* = 5.7, 5.1, 2.5 Hz, 2H), 2.71 – 2.61 (m, 4H), 2.60 – 2.52 (m, 2H), 1.99 – 1.90 (m, 6H).

^{13}C NMR (151 MHz, CDCl$_3$) δ 170.2, 142.3, 141.2, 141.0, 129.3, 128.6, 128.4, 126.2, 125.9, 51.9, 35.6, 35.1, 33.0, 32.9, 23.7, 21.6.

HRMS calcd for $C_{20}H_{24}NO$ $[M+H]^+$ 294.1852, found 294.1851.

1-Chloro-4-(3-phenylpropyl)benzene (27)[4]

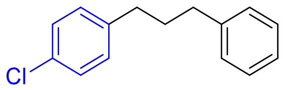

This compound was prepared according to method A (for electron-rich aryl iodides) using 1-chloro-4-iodobenzene and (3-bromopropyl)benzene. Purification by flash column chromatograph to afford **27** as a colorless oil (18.9 mg, 82% yield).

^{1}H NMR (600 MHz, CDCl$_3$) δ 7.29 (t, *J* = 7.5 Hz, 2H), 7.26 – 7.23 (m, 2H), 7.21 – 7.16 (m, 3H), 7.11 (d, *J* = 8.2 Hz, 2H), 2.63 (dt, *J* = 12.5, 7.7 Hz, 4H), 1.97 – 1.91 (m, 2H).

^{13}C NMR (151 MHz, CDCl$_3$) δ 142.2, 140.8, 131.6, 129.9, 128.6, 128.5, 128.5, 126.0, 35.4, 34.9, 33.0.

4,4,5,5-Tetramethyl-2-(4-(3-phenylpropyl)phenyl)-1,3,2-dioxaborolane (28)[7]

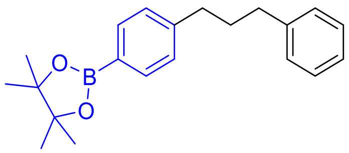

This compound was prepared according to method A (for electron-rich aryl iodides) using 2-(4-iodophenyl)-4,4,5,5-tetramethyl-1,3,2-dioxaborolane and (3-bromopropyl)benzene. Purification by flash column chromatograph to afford **28** as a colorless oil (27.7 mg, 86% yield).

^{1}H NMR (600 MHz, CDCl$_3$) δ 7.78 – 7.68 (m, 2H), 7.28 (dd, *J* = 8.6, 6.8 Hz, 2H), 7.22 – 7.15 (m, 5H), 2.65 (dt, *J* = 15.1, 7.7 Hz, 4H), 2.00 – 1.91 (m, 2H), 1.34 (s, 12H).

^{13}C NMR (151 MHz, CDCl$_3$) δ 145.9, 142.4, 135.0, 128.6, 128.4, 128.1, 125.9, 83.8, 35.8, 35.5, 32.9, 25.0.

1-Methoxy-2-(3-phenylpropyl)benzene (29)[4]

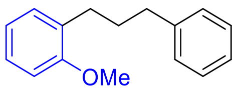

This compound was prepared according to method A (for electron-rich aryl iodides) using 1-iodo-2-methoxybenzene and (3-bromopropyl)benzene. Purification by flash column chromatograph to afford **29** a colorless oil (19.0 mg, 84% yield).

^{1}H NMR (600 MHz, CDCl$_3$) δ 7.29 (t, *J* = 7.6 Hz, 2H), 7.25 – 7.16 (m, 4H), 7.14 (dd, *J* = 7.4, 1.7 Hz, 1H), 6.89 (td, *J* = 7.4, 1.1 Hz, 1H), 6.85 (dd, *J* = 8.2, 1.1 Hz, 1H), 3.82 (s, 3H), 2.72 – 2.62 (m, 4H), 1.99 – 1.88 (m, 2H).

^{13}C NMR (151 MHz, CDCl$_3$) δ 157.6, 142.8, 130.8, 129.9, 128.6, 128.4, 127.1, 125.7, 120.4, 110.4, 55.4, 35.9, 31.5, 30.1.

2-Methoxy-6-(3-phenylpropyl)naphthalene (30)

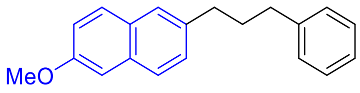

This compound was prepared according to method A (for electron-rich aryl iodides) using 2-iodo-6-methoxynaphthalene and (3-bromopropyl)benzene. Purification by flash column chromatograph to afford **30** a colorless oil (15.5 mg, 56% yield).

^{1}H NMR (600 MHz, CDCl$_3$) δ^{1}H NMR (600 MHz, Chloroform-*d*) δ 7.68 (d, *J* = 8.1 Hz, 2H), 7.56 (d, *J* = 1.3 Hz, 1H), 7.35 – 7.27 (m, 3H), 7.23 – 7.18 (m, 3H), 7.13 (d, *J* = 8.5 Hz, 2H), 3.92 (s, 3H), 2.79 (t, *J* = 7.7 Hz, 2H), 2.72 – 2.66 (m, 2H), 2.05 (tt, *J* = 9.2, 6.9 Hz, 2H).

^{13}C NMR (151 MHz, CDCl$_3$) δ 157.3, 142.5, 137.6, 133.1, 129.2, 129.0, 128.6, 128.4, 128.0, 126.8, 126.4, 125.9, 118.8, 105.8, 55.4, 35.6, 35.5, 33.1.

HRMS calcd for $C_{20}H_{21}O$ $[M+H]^+$ 277.1587, found 277.1572.

2-Methoxy-6-(3-phenylpropyl)quinoline (31)

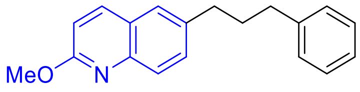

This compound was prepared according to method A (for electron-rich aryl iodides) using 6-iodo-2-methoxyquinoline and (3-bromopropyl)benzene. Purification by flash column chromatograph to afford **31** as a pale yellow oil (21.6 mg, 78% yield).

^{1}H NMR (600 MHz, $CDCl_3$) δ 7.91 (d, *J* = 8.8 Hz, 1H), 7.79 (d, *J* = 8.4 Hz, 1H), 7.51 – 7.46 (m, 2H), 7.30 (d, *J* = 15.2 Hz, 2H), 7.23 – 7.17 (m, 3H), 6.88 (d, *J* = 8.8 Hz, 1H), 4.07 (s, 3H), 2.80 (t, *J* = 7.7 Hz, 2H), 2.72 – 2.65 (m, 2H), 2.09 – 2.00 (m, 2H).

^{13}C NMR (151 MHz, $CDCl_3$) δ 162.2, 145.3, 142.3, 138.4, 138.1, 130.9, 128.6, 128.5, 127.2, 126.3, 125.9, 125.2, 113.1, 53.5, 35.6, 35.3, 33.1.

HRMS calcd for $C_{19}H_{20}NO$ $[M+H]^+$ 278.1539, found 278.1542.

tert-Butyl 6-(3-phenylpropyl)-1_H_-indole-1-carboxylate (32)

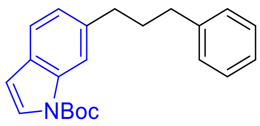

This compound was prepared according to method A (for electron-rich aryl iodides) using *tert*-butyl 6-iodo-1*H*-indole-1-carboxylate and (3-bromopropyl)benzene. Purification by flash column chromatograph to afford **32** as a pale yellow oil (22.8 mg, 68% yield).

^{1}H NMR (600 MHz, $CDCl_3$) δ 8.03 (s, 1H), 7.52 (d, *J* = 3.6 Hz, 1H), 7.46 (d, *J* = 7.9 Hz, 1H), 7.31 – 7.25 (m, 2H), 7.22 – 7.15 (m, 3H), 7.07 (dd, *J* = 8.0, 1.5 Hz, 1H), 6.58 – 6.44 (m, 1H), 2.86 – 2.74 (m, 2H), 2.70 – 2.64 (m, 2H), 2.08 – 1.98 (m, 2H), 1.67 (s, 9H).

^{13}C NMR (151 MHz, $CDCl_3$) δ 167.9, 150.0, 142.5, 138.9, 132.1, 128.6, 128.4, 125.8, 125.5, 123.7, 120.7, 115.0, 107.3, 83.6, 36.2, 35.7, 33.7, 28.4.

HRMS calcd for $C_{22}H_{26}NO_2$ $[M+H]^+$ 336.1958, found 336.1955.

tert-Butyl 3-(3-phenylpropyl)-9_H_-carbazole-9-carboxylate (33)

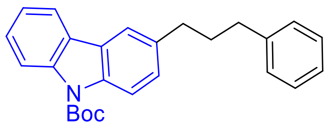

This compound was prepared according to method A (for electron-rich aryl iodides) using *tert*-butyl 3-iodo-9*H*-carbazole-9-carboxylate and (3-bromopropyl)benzene. Purification by flash column chromatograph to afford **33** as an off-white solid (35.5 mg, 92% yield).

^{1}H NMR (600 MHz, $CDCl_3$) δ^{1}H NMR (600 MHz, Chloroform-*d*) δ 8.31 (d, *J* = 8.4 Hz, 1H), 8.21 (d, *J* = 8.5 Hz, 1H), 7.97 (dt, *J* = 7.6, 0.9 Hz, 1H), 7.79 (d, *J* = 1.7 Hz, 1H), 7.46 (ddd, *J* = 8.5, 7.2, 1.3 Hz, 1H),

7.35 (td, J = 7.5, 1.0 Hz, 1H), 7.31 (ddd, J = 8.1, 4.3, 2.3 Hz, 3H), 7.24 – 7.19 (m, 3H), 2.83 (t, J = 7.7 Hz, 2H), 2.76 – 2.67 (m, 2H), 2.11 – 2.02 (m, 2H), 1.77 (s, 9H).

^{13}C NMR (151 MHz, CDCl$_3$) δ 151.3, 142.4, 138.9, 137.2, 137.0, 128.6, 128.5, 127.8, 127.0, 126.0, 125.9, 125.9, 123.0, 119.6, 119.2, 116.4, 116.2, 83.9, 35.6, 35.4, 33.6, 28.5.

HRMS calcd for $C_{26}H_{27}NNaO_2$ [M+Na]$^+$ 408.1934, found 408.1941.

5-(3-Phenylpropyl)-2-(trifluoromethyl)pyridine (34)[11]

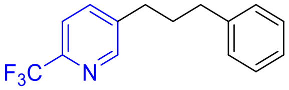

This compound was prepared according to method A (for electron-rich aryl iodides) using 5-iodo-2-(trifluoromethyl)pyridine and (3-bromopropyl)benzene. Purification by flash column chromatograph to afford **34** as a colorless oil (18.6 mg, 70% yield).

^{1}H NMR (600 MHz, CDCl$_3$) δ 8.56 (d, J = 2.0 Hz, 1H), 7.66 (dd, J = 8.1, 2.0 Hz, 1H), 7.60 (d, J = 8.0 Hz, 1H), 7.30 (d, J = 7.5 Hz, 2H), 7.20 (dd, J = 22.6, 7.4 Hz, 3H), 2.71 (dt, J = 25.8, 7.7 Hz, 4H), 2.00 (p, J = 7.7 Hz, 2H).

1-(3-Phenylpropyl)-4-(trifluoromethyl)benzene (35)[4]

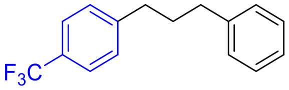

This compound was prepared according to method A (for electron-rich aryl iodides) using 1-iodo-4-(trifluoromethyl)benzene and (3-bromopropyl)benzene. Purification by flash column chromatograph to afford **35** as a colorless oil (24.0 mg, 91% yield).

^{1}H NMR (600 MHz, CDCl$_3$) δ 7.54 (d, J = 7.9 Hz, 2H), 7.30 (t, J = 7.7 Hz, 4H), 7.23 – 7.17 (m, 3H), 2.71 (t, J = 7.8 Hz, 2H), 2.66 (t, J = 7.7 Hz, 2H), 1.98 (tt, J = 9.3, 6.9 Hz, 2H).

Ethyl 2-methyl-2-(4-(3-phenylpropyl)phenoxy)propanoate (36)

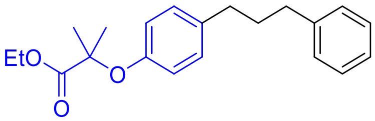

This compound was prepared according to method A (for electron-rich aryl iodides) using ethyl 2-(4-iodophenoxy)-2-methylpropanoate and (3-bromopropyl)benzene. Purification by flash column chromatograph to afford **36** as a colorless oil (22.7 mg, 85% yield).

^{1}H NMR (600 MHz, CDCl$_3$) δ 7.27 (dd, *J* = 8.6, 6.6 Hz, 2H), 7.17 (t, *J* = 7.2 Hz, 3H), 7.04 (d, *J* = 8.4 Hz, 2H), 6.81 – 6.71 (m, 2H), 4.23 (q, *J* = 7.1 Hz, 2H), 2.60 (dt, *J* = 27.1, 7.7 Hz, 4H), 1.91 (tt, *J* = 9.4, 6.7 Hz, 2H), 1.57 (s, 6H), 1.25 (t, *J* = 7.1 Hz, 3H).

^{13}C NMR (151 MHz, CDCl$_3$) δ 174.6, 153.5, 142.5, 136.2, 129.1, 128.6, 128.4, 125.8, 119.4, 61.5, 35.5, 34.7, 33.2, 25.5, 14.2.

HRMS calcd for $C_{21}H_{26}NaO_3$ $[M+Na]^+$ 349.1774, found 349.1774.

2-(4-Fluorophenyl)-5-(2-methyl-5-(3-phenylpropyl)benzyl)thiophene (37)

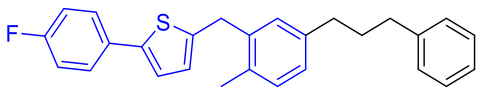

This compound was prepared according to method A (for electron-rich aryl iodides) using 2-(4-fluorophenyl)-5-(5-iodo-2-methylbenzyl)thiophene and (3-bromopropyl)benzene. Purification by flash column chromatograph to afford **37** as a yellow solid (22.4mg, 56% yield).

^{1}H NMR (600 MHz, CDCl$_3$) δ 7.51 – 7.42 (m, 2H), 7.26 (t, *J* = 7.5 Hz, 2H), 7.20 – 7.14 (m, 3H), 7.09 (d, *J* = 7.7 Hz, 1H), 7.06 – 6.94 (m, 5H), 6.66 (d, *J* = 3.6 Hz, 1H), 4.09 (s, 2H), 2.63 (dt, *J* = 13.1, 7.7 Hz, 4H), 2.29 (s, 3H), 1.94 (tt, *J* = 9.2, 6.8 Hz, 2H).

^{13}C NMR (151 MHz, CDCl$_3$) δ 163.0, 161.4, 143.9, 142.5, 140.3, 138.1, 133.7, 130.6, 129.8, 128.6, 128.4, 127.2, 127.2, 127.1, 126.0, 125.8, 122.8, 115.9, 115.7, 35.5, 35.1, 34.3, 33.2, 19.2.

HRMS calcd for $C_{27}H_{26}FS$ $[M+H]^+$ 401.1734, found 401.1738.

Methyl 4-phenethylbenzoate (38)[14]

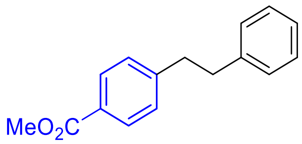

This compound was prepared according to the method A using methyl 4-bromobenzoate and (2-bromoethyl)benzene. Purification by flash column chromatograph to afford **38** as a pale yellow solid (22.3 mg, 93% yield).

^{1}H NMR (600 MHz, CDCl$_3$) δ 7.91 – 7.83 (m, 2H), 7.20 (t, *J* = 7.5 Hz, 2H), 7.16 – 7.10 (m, 3H), 7.09 – 7.05 (m, 2H), 3.82 (s, 3H), 2.94 – 2.82 (m, 4H).

^{13}C NMR (151 MHz, CDCl$_3$) δ 167.3, 147.3, 141.3, 129.8, 128.7, 128.6, 128.5, 128.1, 126.2, 52.1, 38.0, 37.6.

Methyl 4-(3-phenoxypropyl)benzoate (39)

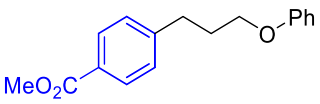

This compound was prepared according to method A using methyl 4-bromobenzoate and (3-bromopropoxy)benzene. Purification by flash column chromatograph to afford **39** as an off-white solid (22.8 mg, 88% yield).

^{1}H NMR (600 MHz, CDCl$_3$) δ 7.97 (d, *J* = 7.9 Hz, 2H), 7.29 (dt, *J* = 7.8, 3.8 Hz, 4H), 6.98 – 6.93 (m, 1H), 6.90 (d, *J* = 8.1 Hz, 2H), 3.96 (t, *J* = 6.2 Hz, 2H), 3.91 (s, 3H), 2.88 (t, *J* = 7.7 Hz, 2H), 2.19 – 2.07 (m, 2H).

^{13}C NMR (151 MHz, CDCl$_3$) δ 167.3, 159.0, 147.2, 129.9, 129.6, 128.7, 128.1, 120.8, 114.6, 66.6, 52.1, 32.4, 30.7.

HRMS calcd for $C_{17}H_{19}O_3$ $[M+H]^+$ 271.1329, found 271.1323.

Methyl 4-(4-methoxy-4-oxobutyl)benzoate (40)[15]

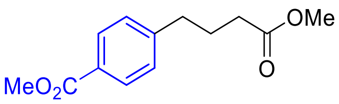

This compound was prepared according to method A using methyl 4-bromobenzoate and methyl 4-bromobutanoate. Purification by flash column chromatograph to afford **40** as a colorless oil (18.7 mg, 79% yield).

^{1}H NMR (600 MHz, CDCl$_3$) δ 7.95 (d, *J* = 8.1 Hz, 2H), 7.24 (d, *J* = 8.0 Hz, 2H), 3.90 (s, 3H), 3.66 (s, 3H), 2.70 (t, *J* = 7.7 Hz, 2H), 2.33 (t, *J* = 7.4 Hz, 2H), 1.97 (p, *J* = 7.5 Hz, 2H).

^{13}C NMR (151 MHz, CDCl$_3$) δ 173.8, 167.2, 147.0, 129.9, 128.6, 128.2, 52.1, 51.7, 35.2, 33.4, 26.2.

Methyl 4-(3-((4-methoxyphenyl)thio)propyl)benzoate (41)

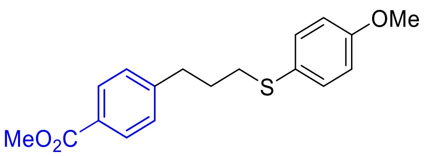

This compound was prepared according to method A using methyl 4-bromobenzoate and (3-bromopropyl)(4-methoxyphenyl)sulfane. Purification by flash column chromatograph to afford **41** as an off-white solid (25.9 mg, 82% yield).

^{1}H NMR (600 MHz, CDCl$_3$) δ 7.97 – 7.92 (m, 2H), 7.36 – 7.30 (m, 2H), 7.24 – 7.20 (m, 2H), 6.86 – 6.81 (m, 2H), 3.90 (s, 3H), 3.80 (s, 3H), 2.84 – 2.75 (m, 4H), 1.90 (dq, *J* = 8.8, 6.8 Hz, 2H).

^{13}C NMR (151 MHz, CDCl$_3$) δ 167.2, 159.1, 147.1, 133.5, 129.9, 128.7,128.1, 126.3, 114.7, 55.5, 52.1, 35.3, 34.7, 30.5.

HRMS calcd for $C_{18}H_{20}NaO_3S$ $[M+Na]^+$ 339.1025, found 339.1024.

Methyl 4-(3-(((8*R*,9*S*,13*S*,14*S*)-13-methyl-17-oxo-7,8,9,11,12,13,14,15,16,17-decahydro-6*H* -cyclopenta[a] -phenanthren-3-yl)oxy)propyl)benzoate (42)

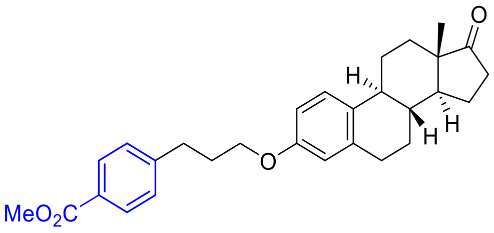

This compound was prepared according to method A using methyl 4-bromobenzoate and (8*R*,9*S*,13*S*,14*S*)-3-(3-bromopropoxy)-13-methyl-6,7,8,9,11,12,13,14,15,16-decahydro-17*H*-cyclopenta[a]phenanthren-17-one. Purification by flash column chromatograph to afford **42** as an off-white solid (34.8 mg, 78% yield).

^{1}H NMR (600 MHz, CDCl$_3$) δ 7.96 (d, *J* = 8.2 Hz, 2H), 7.28 (d, *J* = 8.2 Hz, 2H), 7.19 (d, *J* = 8.5 Hz, 1H), 6.70 (dd, *J* = 8.6, 2.8 Hz, 1H), 6.63 (d, *J* = 2.7 Hz, 1H), 3.93 (t, *J* = 6.2 Hz, 2H), 3.90 (s, 3H), 2.92 – 2.81 (m, 4H), 2.55 – 2.46 (m, 1H), 2.43 – 2.36 (m, 1H), 2.25 (td, *J* = 10.9, 4.4 Hz, 1H), 2.18 – 1.94 (m, 6H), 1.65 – 1.58 (m, 2H), 1.54 – 1.41 (m, 4H), 0.91 (s, 3H).

^{13}C NMR (151 MHz, CDCl$_3$) δ 221.1, 167.3, 157.1, 147.3, 137.9, 132.2, 129.9, 128.7, 128.1, 126.5, 114.7, 112.3, 66.6, 52.1, 50.6, 48.2, 44.1, 38.5, 36.0, 32.4, 31.7, 30.7, 29.8, 26.7, 26.1, 21.7, 14.0.

HRMS calcd for $C_{29}H_{35}O_4$ $[M+H]^+$ 447.2530, found 447.2532.

Methyl 4-((1,3-dioxolan-2-yl)methyl)benzoate (43)[16]

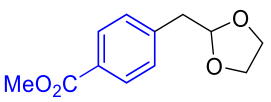

This compound was prepared according to method A using methyl 4-bromobenzoate and 2-(bromomethyl)-1,3-dioxolane. Purification by flash column chromatograph to afford **43** as a colorless oil (18.7 mg, 84% yield).

^{1}H NMR (600 MHz, CDCl$_3$) δ 7.97 (d, *J* = 8.1 Hz, 2H), 7.34 (d, *J* = 8.0 Hz, 2H), 5.09 (t, *J* = 4.6 Hz, 1H), 3.91 (d, *J* = 9.6 Hz, 5H), 3.87 – 3.82 (m, 2H), 3.02 (d, *J* = 4.6 Hz, 2H).

^{13}C NMR (151 MHz, CDCl$_3$) δ 167.2, 141.6, 130.0, 129.7, 128.7, 104.2, 65.2, 52.2, 40.9.

Methyl 4-(3-(4-bromophenyl)propyl)benzoate (44)

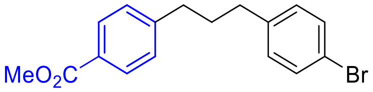

This compound was prepared according to method A using methyl 4-bromobenzoate and 1-bromo-4-(3-iodopropyl)benzene. Purification by flash column chromatograph to afford **44** as a colorless oi (29.5 mg, 89% yield).

<u>^{1}H NMR (600 MHz, $CDCl_3$)</u> δ 7.97 – 7.93 (m, 2H), 7.45 – 7.37 (m, 2H), 7.25 – 7.20 (m, 2H), 7.08 – 7.01 (m, 2H), 3.90 (s, 3H), 2.71 – 2.66 (m, 2H), 2.61 – 2.56 (m, 2H), 1.97 – 1.91 (m, 2H).

<u>^{13}C NMR (151 MHz, $CDCl_3$)</u> δ 167.3, 147.6, 141.0, 131.5, 130.3, 129.9, 128.6, 128.0, 119.7, 52.1, 35.4, 34.8, 32.5.

HRMS calcd for $C_{17}H_{18}BrO_2$ $[M+H]^+$ 333.0485, found 333.0477.

Methyl 4-(4-phenylbutan-2-yl)benzoate (45)[4]

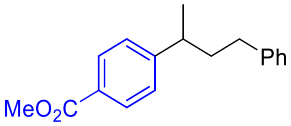

This compound was prepared according to method A (for secondary alkyl bromides) using methyl 4-bromobenzoate and (3-bromobutyl)benzene. Purification by flash column chromatograph to afford **45** as a colorless oil (23.1 mg, 86% yield).

<u>^{1}H NMR (600 MHz, $CDCl_3$)</u> δ 8.02 – 7.96 (m, 2H), 7.30 – 7.22 (m, 5H), 7.19 – 7.15 (m, 1H), 7.13 – 7.08 (m, 2H), 3.91 (s, 3H), 2.78 (h, *J* = 7.0 Hz, 1H), 2.55 – 2.43 (m, 2H), 1.99 – 1.88 (m, 2H), 1.28 (d, *J* = 6.9 Hz, 3H).

<u>^{13}C NMR (151 MHz, $CDCl_3$)</u> δ 167.3 , 152.9 , 142.3 , 130.0 , 128.5 , 128.1 , 127.3 , 125.9 , 52.1 , 39.8, 39.7, 33.9 , 22.4 .

Methyl 4-cyclohexylbenzoate (46)[17]

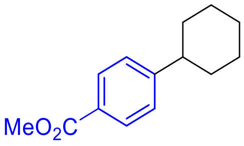

This compound was prepared according to method A (for secondary alkyl bromides) using methyl 4-bromobenzoate and bromocyclohexane. Purification by flash column chromatograph to afford **46** as a colorless oil (12.2 mg, 56% yield).

^{1}H NMR (600 MHz, CDCl$_3$) δ 8.04 – 7.87 (m, 2H), 7.27 (s, 2H), 3.89 (s, 3H), 2.56 (tt, *J* = 11.7, 3.4 Hz, 1H), 1.86 (ddt, *J* = 12.3, 6.4, 2.4 Hz, 4H), 1.76 (dtt, *J* = 12.9, 3.1, 1.5 Hz, 1H), 1.48 – 1.35 (m, 4H), 1.30 – 1.22 (m, 1H).

^{13}C NMR (151 MHz, CDCl$_3$) δ 166.6, 152.8, 129.0, 127.1, 126.2, 51.3, 44.0, 33.5, 26.1, 25.4.

Methyl 4-(tetrahydro-2*H*-pyran-4-yl)benzoate (47)[17]

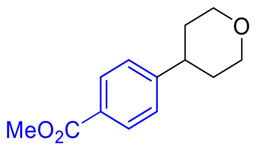

This compound was prepared according to t method A (for secondary alkyl bromides) using methyl 4-bromobenzoate and 4-bromotetrahydro-2*H*-pyran. Purification by flash column chromatograph to afford **47** as an off-white solid (16.7 mg, 76% yield).

^{1}H NMR (600 MHz, CDCl$_3$) δ 8.02 – 7.95 (m, 2H), 7.32 – 7.27 (m, 2H), 4.09 (ddt, *J* = 11.6, 4.4, 1.0 Hz, 2H), 3.90 (s, 3H), 3.53 (td, *J* = 11.7, 2.3 Hz, 2H), 2.82 (tt, *J* = 11.9, 4.1 Hz, 1H).

^{13}C NMR (151 MHz, CDCl$_3$) δ 167.2, 151.2, 130.0, 128.4, 126.9, 68.3, 52.2, 41.8, 33.7.

Methyl 4-(1-tosylpiperidin-4-yl)benzoate (48)[4]

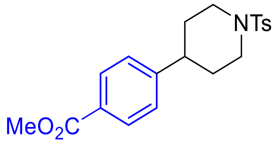

This compound was prepared according to method A (for secondary alkyl bromides) using methyl 4-bromobenzoate and 4-bromo-1-tosylpiperidine. Purification by flash column chromatograph to afford **48** as an off-white solid (18.7 mg, 50% yield).

^{1}H NMR (600 MHz, CDCl$_3$) δ 7.96 (d, *J* = 8.0 Hz, 2H), 7.68 (d, *J* = 8.0 Hz, 2H), 7.35 (d, *J* = 7.9 Hz, 2H), 7.21 (d, *J* = 8.0 Hz, 2H), 3.98 – 3.92 (m, 2H), 3.90 (s, 3H), 2.46 (s, 4H), 2.35 (td, *J* = 11.7, 3.3 Hz, 2H), 1.87 (qd, *J* = 12.3, 10.8, 4.9 Hz, 4H).

^{13}C NMR (151 MHz, CDCl$_3$) δ 167.04, 150.3, 143.72, 133.2, 130.09, 129.80, 128.7, 127.90, 126.89, 52.21, 46.82, 42.05, 32.40, 21.69.

Methyl 4-(oxetan-3-yl)benzoate (49)[17]

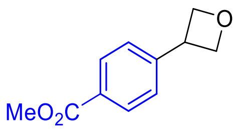

This compound was prepared according to method A (for secondary alkyl bromides) using methyl 4-bromobenzoate and 3-bromooxetane. Purification by flash column chromatograph to afford **49** as an off-white solid (12.5 mg, 65% yield).

^{1}H NMR (600 MHz, $CDCl_3$) δ 8.04 (d, J = 8.0 Hz, 2H), 7.47 (d, J = 8.0 Hz, 2H), 5.10 (dd, J = 8.3, 6.1 Hz, 2H), 4.77 (t, J = 6.3 Hz, 2H), 4.34 – 4.22 (m, 1H), 3.92 (s, 3H).

^{13}C NMR (151 MHz, $CDCl_3$) δ 167.0 , 146.9 , 130.3 , 129.1 , 127.0 , 78.56, 52.3 , 40.4 .

Methyl 4-(2-(cyclohex-1-en-1-yl)ethyl)benzoate (50)

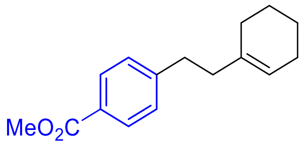

This compound was prepared according to method A (for pyridinium salts) using methyl 4-bromobenzoate and 1-(2-(cyclohex-1-en-1-yl)ethyl)-2,4,6 -triphenylpyridin-1-ium tetrafluoroborate. Purification by flash column chromatograph to afford **50** as a colorless oil (12.7 mg, 52% yield).

^{1}H NMR (600 MHz, $CDCl_3$) δ 8.01 – 7.88 (m, 2H), 7.33 – 7.19 (m, 3H), 5.39 (tt, J = 3.7, 1.5 Hz, 1H), 3.90 (s, 3H), 2.82 – 2.71 (m, 2H), 2.23 (t, J = 8.1 Hz, 2H), 1.96 (dq, J = 6.5, 4.0, 3.0 Hz, 4H), 1.66 – 1.60 (m, 2H), 1.56 – 1.51 (m, 2H).

^{13}C NMR (151 MHz, $CDCl_3$) δ 167.4, 148.3, 136.8, 129.7, 128.6, 127.8, 121.9, 52.1, 39.7, 34.6, 28.6, 25.4, 23.1, 22.6.

HRMS calcd for $C_{16}H_{20}NaO_2$ $[M+Na]^+$ 267.135, found 267.1331.

Methyl 4-(2-(6-(2-(tert-butoxy)-2-oxoethyl)-2,2-dimethyl-1,3-dioxan-4-yl)ethyl)benzoate (51)

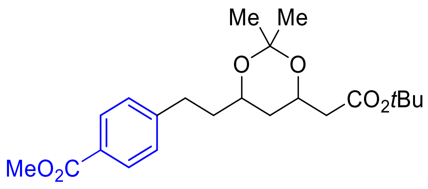

This compound was prepared according to method A (for pyridinium salts) using methyl 4-bromobenzoate and 1-(2-(6-(2-(tert-butoxy)-2-oxoethyl)-2,2-dimethyl-1,3-dioxan-4-yl)ethyl)-2,4,6-triphenylpyridin-1-ium tetrafluoroborate. Purification by flash column chromatograph to afford **51** as an off-white solid (33.7 mg, 86% yield).

^{1}H NMR (600 MHz, $CDCl_3$) δ 7.99 – 7.92 (m, 2H), 7.26 – 7.22 (m, 2H), 4.20 (dtd, J = 11.5, 6.6, 2.4 Hz, 1H), 3.90 (s, 3H), 3.78 (dddd, J = 11.0, 8.1, 4.3, 2.5 Hz, 1H), 2.79 (ddd, J = 14.2, 9.1, 5.2 Hz, 1H), 2.71 (dt, J = 13.9, 8.2 Hz, 1H), 2.42 (dd, J = 15.1, 7.0 Hz, 1H), 2.28 (dd, J = 15.1, 6.2 Hz, 1H), 1.83 (dtd, J = 13.8,

8.6, 5.3 Hz, 1H), 1.70 (dddd, J = 13.5, 9.1, 7.5, 4.2 Hz, 1H), 1.53 (dt, J = 12.7, 2.5 Hz, 1H), 1.43 (s, 9H), 1.41 (s, 3H), 1.39 (s, 3H), 1.21 (dt, J = 12.7, 11.5 Hz, 1H).

^{13}C NMR (151 MHz, CDCl$_3$) δ 170.4, 167.3, 147.7, 129.8, 128.7, 128.0, 98.9, 80.76, 67.7, 66.3, 52.1, 42.8, 37.6, 36.7, 31.3, 30.3, 28.2, 19.9.

HRMS calcd for $C_{22}H_{32}NaO_6$ $[M+Na]^+$ 415.2091, found 415.2091.

Methyl 4-((2,2-dimethyl-1,3-dioxolan-4-yl)methyl)benzoate (52)

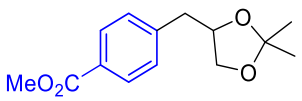

This compound was prepared according to method C using (2,2-dimethyl-1,3-dioxolan-4-yl)methanol (1.5 equiv), CEBO, TBAB (1.5 equiv) in CH_3CN (0.2 mL). After 5 min, to the crude mixture of alkyl bromide was added methyl 4-bromobenzoate. Purification by flash column chromatograph to afford **52** as a colorless oil (12.8 mg, 51% yield).

^{1}H NMR (600 MHz, CDCl$_3$.) δ 8.07 – 7.91 (m, 2H), 7.29 (d, J = 8.0 Hz, 2H), 4.33 (p, J = 6.5 Hz, 1H), 3.98 (ddd, J = 8.2, 5.9, 0.8 Hz, 1H), 3.90 (d, J = 0.8 Hz, 3H), 3.63 (ddd, J = 7.8, 6.8, 0.8 Hz, 1H), 3.02 (dd, J = 13.8, 6.6 Hz, 1H), 2.85 (dd, J = 13.8, 6.4 Hz, 1H), 1.42 (s, 3H), 1.35 (d, J = 0.9 Hz, 3H).

^{13}C NMR (151 MHz, CDCl$_3$) δ 167.2, 143.2, 129.9, 129.4, 128.7, 109.5, 69.0, 52.2, 40.2, 27.1, 25.8.

HRMS calcd for $C_{14}H_{19}O_4$ $[M+H]^+$ 251.1278, found 251.1281.

Sec-butyl 2-(4-(methoxycarbonyl)phenethyl)piperidine-1-carboxylate (53)[4]

This compound was prepared according to method C using methyl 4-bromobenzoate and sec-butyl 2-(2-hydroxyethyl)piperidine-1-carboxylate. Purification by flash column chromatograph to afford **53** as a colorless oil (16.3 mg, 47% yield).

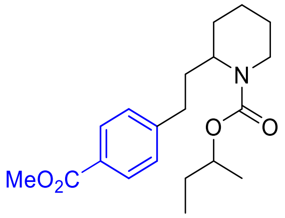

^{1}H NMR (600 MHz, CDCl$_3$) δ 7.95 (d, J = 8.1 Hz, 2H), 7.25 (d, J = 8.1 Hz, 2H), 4.76 (dtt, J = 12.5, 6.2, 3.2 Hz, 1H), 4.35 (s, 1H), 4.04 (d, J = 20.8 Hz, 1H), 3.90 (s, 3H), 2.82 (t, J = 13.3 Hz, 1H), 2.72 – 2.54 (m, 2H), 2.05 (tq, J = 15.1, 5.8 Hz, 1H), 1.81 – 1.68 (m, 1H), 1.62 – 1.48 (m, 7H), 1.41 (d, J = 12.0 Hz, 1H), 1.20 (d, J = 6.3 Hz, 3H), 0.90 (q, J = 7.4 Hz, 3H).

¹³C NMR (151 MHz, CDCl₃) δ 167.3, 155.9, 129.9, 128.5, 128.5, 128.0, 73.0, 52.1, 50.5, 39.0, 33.0, 31.6, 29.3, 28.6, 25.7, 20.0, 19.2, 9.8.

Methyl 4-(cyclopentylmethyl)benzoate (54)[17]

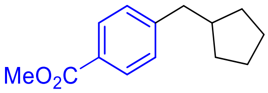

This compound was prepared according to method C using methyl 4-bromobenzoate and cyclopentylmethanol. Purification by flash column chromatograph to afford **54** as a colorless oil (15.1 mg, 69% yield).

¹H NMR (600 MHz, CDCl₃) δ 7.94 (d, *J* = 8.3 Hz, 2H), 7.23 (d, *J* = 8.2 Hz, 2H), 3.90 (s, 3H), 2.66 (d, *J* = 7.5 Hz, 2H), 2.14 – 2.05 (m, 1H), 1.75 – 1.61 (m, 4H), 1.56 – 1.47 (m, 2H), 1.23 – 1.13 (m, 2H).

¹³C NMR (151 MHz, CDCl₃) δ 167.4, 148.1, 129.7, 128.9, 127.7, 52.1, 42.3, 41.9, 32.6, 25.0.

Methyl 4-(2-((4-methylphenyl)sulfonamido)ethyl)benzoate (55)[18]

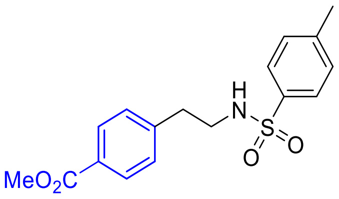

This compound was prepared according to method C using methyl 4-bromobenzoate and *N*-(2-hydroxyethyl)-4-methylbenzenesulfonamide. Purification by flash column chromatograph to afford **55** as an off-white solid (20.7 mg, 62% yield).

¹H NMR (600 MHz, CDCl₃) δ 8.00 – 7.89 (m, 2H), 7.76 – 7.63 (m, 2H), 7.31 – 7.27 (m, 2H), 7.18 – 7.10 (m, 2H), 4.42 (t, *J* = 6.3 Hz, 1H), 3.91 (s, 3H), 3.24 (q, *J* = 6.8 Hz, 2H), 2.83 (t, *J* = 6.9 Hz, 2H), 2.43 (s, 3H).

Methyl 4-(heptan-4-yl)benzoate (56)[4]

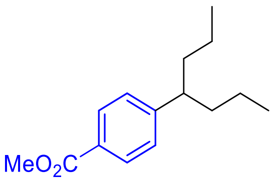

This compound was prepared according to method C using methyl 4-bromobenzoate and heptan-4-ol. Purification by flash column chromatograph to afford **56** as a colorless oil (10.8 mg, 46% yield).

^{1}H NMR (600 MHz, CDCl$_3$) δ 7.98 – 7.92 (m, 2H), 7.21 (d, *J* = 8.2 Hz, 2H), 3.90 (s, 3H), 2.59 (tt, *J* = 9.7, 5.3 Hz, 1H), 1.61 (dddd, *J* = 13.5, 10.0, 6.3, 5.3 Hz, 3H), 1.53 (dtd, *J* = 13.5, 9.5, 5.3 Hz, 2H), 1.21 – 1.05 (m, 3H), 0.83 (t, *J* = 7.3 Hz, 6H).

^{13}C NMR (151 MHz, CDCl$_3$) δ 167.4, 152.2, 129.7, 127.9, 127.9, 52.1, 45.8, 39.1, 20.8, 14.2.

Methyl 4-(3-hydroxypropyl)benzoate (57)[4]

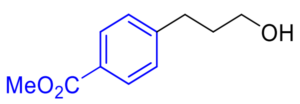

This compound was prepared according to method C using methyl 4-bromobenzoate and propane-1,3-diol. Purification by flash column chromatograph to afford **57** as a colorless oil (7.8 mg, 40% yield).

^{1}H NMR (600 MHz, CDCl$_3$) δ 8.02 – 7.88 (m, 2H), 7.34 – 7.17 (m, 2H), 3.90 (s, 3H), 3.67 (t, *J* = 6.4 Hz, 2H), 2.76 (dd, *J* = 8.7, 6.8 Hz, 2H), 1.98 – 1.85 (m, 2H), 1.72 (s, 1H).

^{13}C NMR (151 MHz, CDCl$_3$) δ 167.3, 147.6, 129.9, 128.6, 128.0, 62.1, 52.1, 33.9, 32.2.

Methyl 4-(3-hydroxy-3-methylbutyl)benzoate (58)[4]

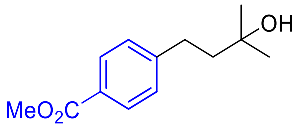

This compound was prepared according to method C using methyl 4-bromobenzoate and 3-methylbutane-1,3-diol. Purification by flash column chromatograph to afford **58** as an off-white solid (13.3 mg, 60% yield).

^{1}H NMR (600 MHz, CDCl$_3$) δ 7.95 (d, *J* = 8.3 Hz, 2H), 7.27 (d, *J* = 8.5 Hz, 2H), 3.90 (s, 3H), 2.86 – 2.68 (m, 2H), 1.89 – 1.74 (m, 2H), 1.30 (s, 6H).

^{13}C NMR (151 MHz, CDCl$_3$) δ 167.3, 148.3, 129.9, 128.5, 127.9, 71.0, 52.1, 45.4, 31.0, 29.5.

Methyl 4-(((3a*R*,4*R*,5*R*,6a*S*)-5-hydroxy-2-oxohexahydro-2*H*-cyclopenta[b]furan-4-yl)methyl) -benzoate (59)

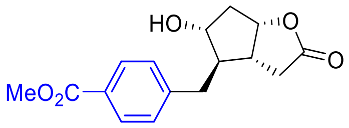

This compound was prepared according to method C using methyl 4-bromobenzoate and (3a*R*,4*S*,5*R*,6a*S*)-5-hydroxy-4-(hydroxymethyl)hexahydro-2*H*-cyclopenta[b]furan-2-one Purification by flash column chromatograph to afford **59** as an off-white solid (16.5 mg, 57% yield).

^{1}H NMR (600 MHz, CDCl$_3$) δ 8.02 – 7.93 (m, 2H), 7.25 – 7.21 (m, 2H), 4.94 (td, *J* = 7.0, 2.5 Hz, 1H), 4.01 (q, *J* = 5.5 Hz, 1H), 3.91 (s, 3H), 2.85 (dd, *J* = 13.8, 6.5 Hz, 1H), 2.65 – 2.50 (m, 3H), 2.37 (ddd, *J* = 15.0, 6.8, 5.8 Hz, 1H), 2.22 – 2.14 (m, 2H), 2.03 (ddd, *J* = 15.0, 5.5, 2.7 Hz, 2H).

^{13}C NMR (151 MHz, CDCl$_3$) δ 177.2, 170.0, 144.6, 130.2, 129.1, 128.8, 83.4, 55.1, 52.3, 42.4, 40.5, 38.8, 35.6.

Methyl 4-(((3a*R*,4*R*,6*R*,6a*R*)-6-(2,4-dioxo-3,4-dihydropyrimidin-1(2*H*)-yl)-2,2-dimethyltetrahydro -furo[3,4-d][1,3]dioxol-4-yl)methyl)benzoate (60)[4]

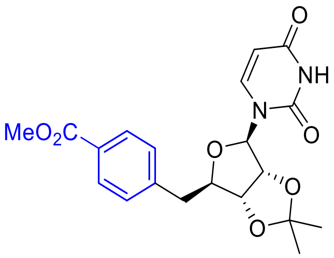

This compound was prepared according to method C using methyl 4-bromobenzoate and 1-((3a*R*,4*R*,6*R*,6a*R*)-6-(hydroxymethyl)-2,2-dimethyltetrahydrofuro[3,4-d][1,3]dioxol-4-yl)pyrimidine-2,4(1*H*,3*H*)-dione. Purification by flash column chromatograph to afford **60** as a colorless oil (14.5 mg, 36% yield).

^{1}H NMR (600 MHz, CDCl$_3$) δ 8.10 – 8.03 (m, 2H), 7.75 (s, 1H), 7.23 (d, *J* = 8.2 Hz, 2H), 6.11 (s, 1H), 4.63 (d, *J* = 5.5 Hz, 1H), 4.49 (d, *J* = 5.6 Hz, 1H), 4.41 (dd, *J* = 4.3, 1.8 Hz, 1H), 3.93 (s, 3H), 3.63 (d, *J* = 11.8 Hz, 1H), 3.57 (td, *J* = 11.4, 4.7 Hz, 1H), 1.77 (ddd, *J* = 13.7, 11.0, 4.2 Hz, 1H).

^{13}C NMR (151 MHz, CDCl$_3$) δ 168.5, 166.5, 150.6, 137.5, 130.7, 130.6, 129.7, 113.3, 86.1, 83.6, 81.2, 80.0, 54.8, 52.5, 51.0, 31.8, 26.0, 24.9.

Methyl 4-((3a*R*,5*S*,6*S*,6a*R*)-5-((R)-2,2-dimethyl-1,3-dioxolan-4-yl)-2,2-dimethyltetrahydrofuro[2,3-d][1,3]dioxol-6-yl)benzoate (61)[4]

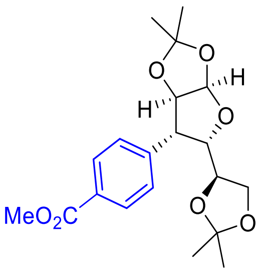

This compound was prepared according to method C using methyl 4-bromobenzoate and (3a*R*,5*S*,6*S*,6a*R*)-5-((R)-2,2-dimethyl-1,3-dioxolan-4-yl)-2,2-dimethyltetrahydrofuro[2,3-d][1,3]dioxol-6-ol. Purification by flash column chromatograph to afford **61** as an off-white solid (22.3 mg, 59% yield).

^{1}H NMR (600 MHz, CDCl$_3$) δ 7.99 – 7.90 (m, 2H), 7.36 – 7.29 (m, 2H), 6.00 (d, *J* = 3.7 Hz, 1H), 4.58 (d, *J* = 3.7 Hz, 1H), 4.30 (dd, *J* = 7.2, 3.9 Hz, 1H), 4.22 (d, *J* = 3.9 Hz, 1H), 3.90

(s, 3H), 3.71 (ddd, J = 10.0, 7.2, 2.9 Hz, 1H), 3.09 (dd, J = 14.4, 2.9 Hz, 1H), 2.86 (dd, J = 14.4, 9.8 Hz, 1H), 1.60 (s, 2H), 1.50 (s, 3H), 1.33 (d, J = 7.9 Hz, 6H), 1.13 (s, 3H).

^{13}C NMR (151 MHz, CDCl$_3$) δ 167.3, 144.0, 129.6, 129.4, 128.4, 112.4, 106.5, 101.1, 84.2, 83.2, 75.1, 72.5, 52.1, 39.8, 27.4, 26.7, 24.1, 23.8.

1-Dodecyl-2-methylbenzene (64)[20]

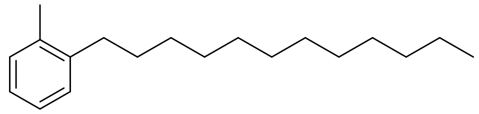

This compound was prepared according to method A (for electron-rich aryl iodides) using 1-iodo-2-methylbenzene and 1-bromododecane. Purification by flash column chromatograph to afford **64** a colorless oil (22.5 mg, 86% yield)

^{1}H-NMR (600 MHZ, CDCl$_3$) δ 7.19 – 7.05 (m, 4H), 2.62 – 2.55 (m, 2H), 2.31 (s, 3H), 1.62 – 1.51 (m, 2H), 1.41 – 1.21 (m, 19H), 0.89 (t, J = 7.0 Hz, 3H).

^{13}C NMR (151 MHz, CDCl$_3$) δ 141.1, 135.9, 130.0, 128.7, 125.8, 125.7, 33.3, 31.9, 30.3, 29.7, 29.7, 29.7, 29.6, 29.6, 29.6, 29.4, 22.7, 19.3, 14.1.

Unsuccessful Examples:

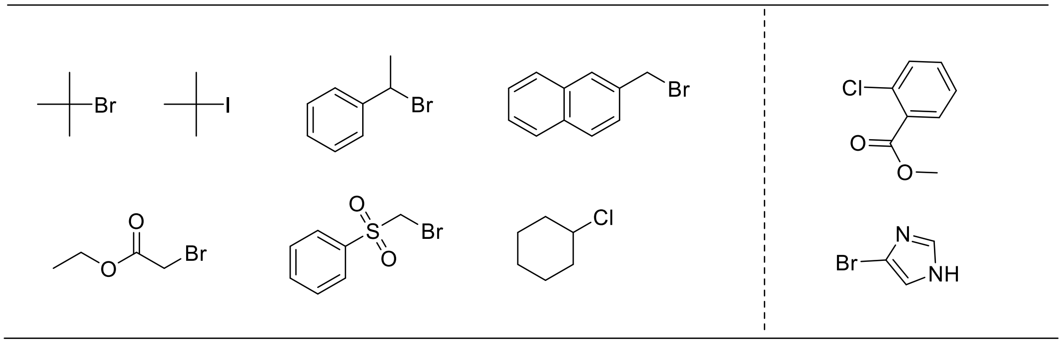

Part 4. Homogeneous Reaction conditions

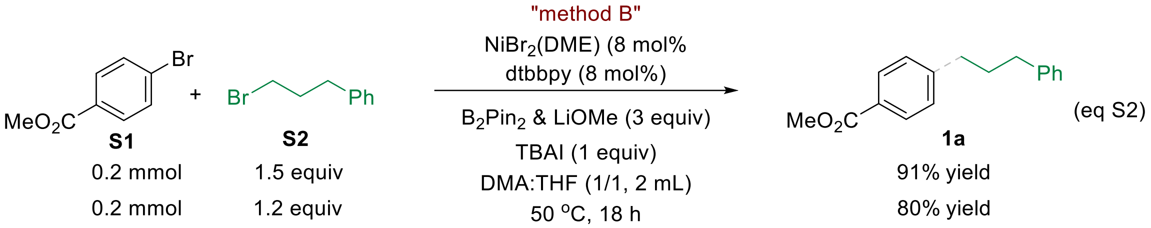

Method B for homogeneous reaction of S1 and S2: To a flame-dried tube charged with a stir bar was added 4-bromobenzoate (43 mg, 0.2 mmol, 1.0 equiv), $NiBr_2$(DME) (5.0 mg, 8 mol %) and dtbbpy (4.3 mg, 8 mol %). The flask was transferred into an N_2-filled glovebox, B_2pin_2 (152.4 mg, 0.6 mmol, 3 equiv), TBAI (74 mg, 0.2 mmol, 1 equiv) and LiOMe (22.8 mg, 0.6 mmol, 3 equiv) were added. The flask was sealed, removed from the glovebox and charged with DMA/THF (v/v=1:1, 2 mL). The mixture was stirred vigorously, alkyl bromide (59.7 mg, 0.6 mmol, 1.5 equiv) was added to the flask. The mixture was stirred vigorously at 50 °C for 24 h (detected by GCMS). After that, the mixture was quenched with water (10 mL) and extracted with EtOAc (3 x 10 mL) and the combined organic layers were concentrated under reduced pressure. The residue was purified by column chromatography on silica gel (eluted with 0-3% EtOAc/Petroleum ether) to give product **1a** (46 mg, 91%).

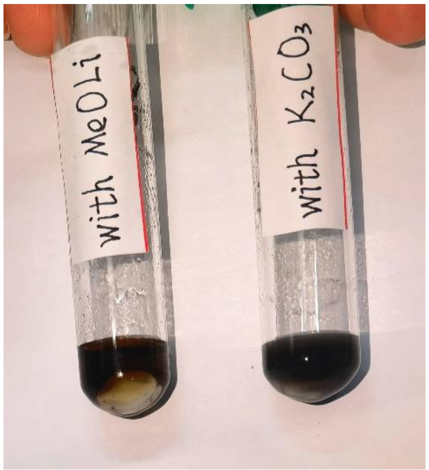

Part 5. Initial Test of a Three-Component Reaction of Alkyl Halide/Alkene/Aryl Halides

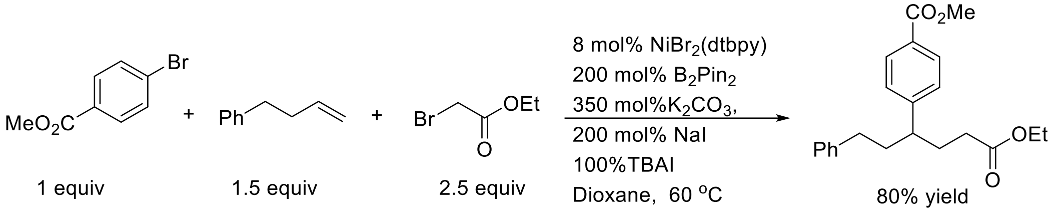

(eq S3)

To a flame-dried tube charged with a stir bar was added 4-bromobenzoate (22 mg, 0.1 mmol, 1.0 equiv) and $NiBr_2$(dtbbpy) (4.0 mg, 8 mol %). The flask was transferred into an N_2-filled glovebox, B_2pin_2 (50.8 mg, 0.2 mmol, 2 equiv), TBAI (37 mg, 0.1 mmol, 1 equiv), NaI (30 mg, 0.2 mmol, 2.0 equiv) and K_2CO_3 (49.5 mg, 0.35 mmol, 3.5 equiv) were added. The tube was sealed, removed from the glovebox and charged with dioxane (1.0 mL). The mixture was stirred vigorously, but-3-en-1-ylbenzene (19.9 mg, 0.15mmol, 1.5 equiv) and alkyl bromide (41.8 mg, 0.25 mmol, 2.5 equiv) were added to the tube. The mixture was stirred vigorously at 60 °C for 48 h. After that, the reaction mixture was quenched with water (10 mL), extracted with EtOAc (3 x 15 mL). The combined organic layers were washed with brine (2 x 10 mL), dried with $MgSO_4$, filtered, and concentrated under reduced pressure. The residue was purified by chromatography on silica gel, eluting with ethyl acetate/petroleum ether to give **1c** (28.4 mg, 80% yield).

Methyl 4-(6-ethoxy-6-oxo-1-phenylhexan-3-yl)benzoate(1c)

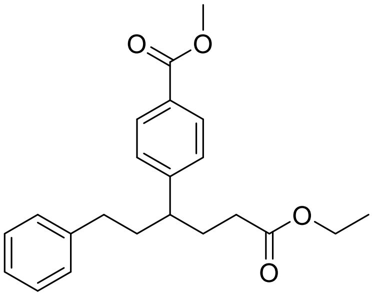

^{1}H-NMR (600 MHZ, $CDCl_3$) δ 8.00 (d, *J* = 8.3 Hz, 2H), 7.28 – 7.23 (m, 4H), 7.19 – 7.14 (m, 1H), 7.10 – 7.05 (m, 2H), 4.05 (qd, *J* = 7.2, 3.0 Hz, 2H), 3.92 (s, 3H), 2.64 (tt, *J* = 9.5, 4.6 Hz, 1H), 2.44 (t, *J* = 7.9 Hz, 2H), 2.14 – 1.83 (m, 6H), 1.20 (t, *J* = 7.1 Hz, 3H).

^{13}C NMR (151 MHz, $CDCl_3$) δ 173.3, 167.0, 149.8, 141.8, 129.9, 128.5, 128.3, 128.3, 127.8, 125.8, 60.3, 52.0, 44.9, 38.1, 33.5, 32.2, 31.6, 14.1.

Part 6. Mechanistic consideration

1. Suzuki coupling with method A

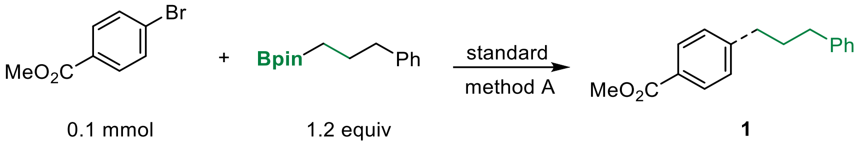

To a flame-dried tube charged with a stir bar was added methyl 4-bromobenzoate (0.1 mmol, 1.0 equiv), $NiBr_2$(dtbbpy) (4 mg, 8 mol %). The tube was transferred into an N_2-filled glovebox, B_2pin_2 (38 mg, 0.15 mmol, 1.5 equiv), NaI (7.5 mg, 0.05 mmol, 0.5 equiv) and K_2CO_3 (25 mg, 0.18 mmol, 1.8 equiv) were added. The tube was sealed, removed from the glovebox and charged with DMA (0.5 mL). The mixture was stirred vigorously, 4,4,5,5-tetramethyl-2-(3-phenylpropyl)-1,3,2-dioxaborolane (0.12 mmol, 1.2 equiv) was added to the tube. The mixture was stirred vigorously at 60 °C for 24 h. After that, the reaction mixture was quenched with water (2 mL), extracted with EtOAc (2 mL) and analyzed by GCMS. Trace of **1** was detected.

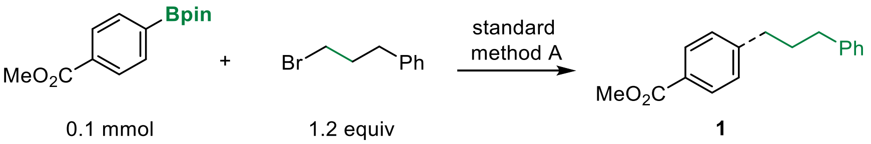

To a flame-dried tube with a stir bar was added methyl 4-(4,4,5,5-tetramethyl-1,3,2-di oxaborolan-2 -yl)benzoate (0.1 mmol, 1.0 equiv), $NiBr_2$(dtbbpy) (4 mg, 8 mol %). The tube was transferred into an N_2-filled glovebox, B_2pin_2 (38 mg, 0.15 mmol, 1.5 equiv), NaI (7.5 mg, 0.05 mmol, 0.5 equiv) and K_2CO_3 (25 mg, 0.18 mmol, 1.8 equiv) were added. The tube was sealed, removed from the glovebox and charged with DMA (0.5 mL). The mixture was stirred vigorously, (4-bromobutyl)benzene (0.12 mmol, 1.2 equiv) was added to the tube. The mixture was stirred vigorously at 60 °C for 24 h. After that, the reaction was quenched with water (2 mL), extracted with EtOAc (2 mL) and analyzed by GCMS. Trace of **1** was detected.

2. Radical clock reaction

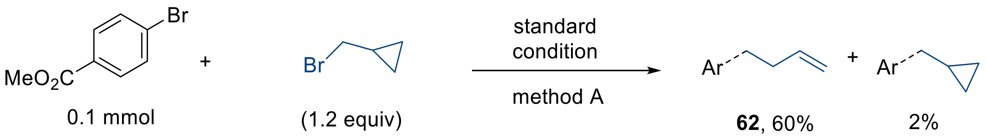

To a flame-dried tube charged with a stir bar was added methyl 4-bromobenzoate (21.5 mg, 0.1 mmol, 1.0

equiv), $NiBr_2$(dtbbpy) (4 mg, 8 mol %). The tube was transferred into an N_2-filled glovebox, B_2pin_2 (38 mg, 0.15 mmol, 1.5 equiv), NaI (7.5 mg, 0.05 mmol, 0.5 equiv) and K_2CO_3 (25 mg, 0.18 mmol, 1.8 equiv) were added. The tube was sealed, removed from the glovebox and charged with DMA (0.5 mL). The mixture was stirred vigorously, (bromomethyl)cyclopropane (0.12 mmol, 1.2 equiv) was added to the tube. The mixture was stirred vigorously at 60 °C for 24 h. After that, the reaction mixture was quenched with water (10 mL), extracted with EtOAc (3 x 15 mL). The combined organic layers were washed with brine (2 x 10 mL), dried with $MgSO_4$, filtered, and concentrated under reduced pressure. The residue was purified by chromatography on silica gel, eluting with ethyl acetate/petroleum ether to give the target molecular. The yield was determined by ^{1}H NMR (using 2,5-dimethylfuran as the internal standard).

3. Radical-chain experiment

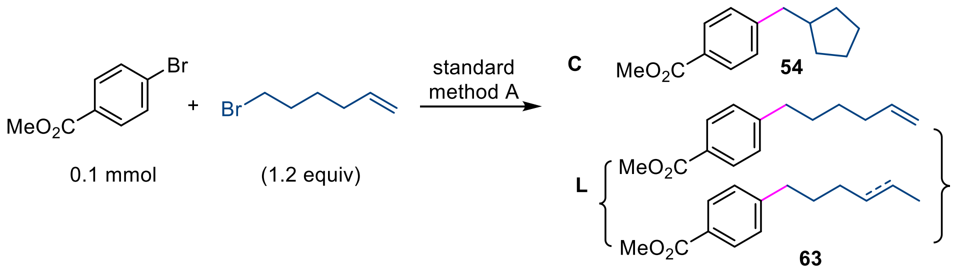

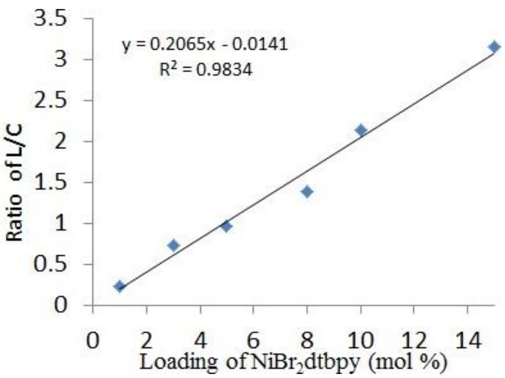

Figure S0. Linear Dependence of the ratio of L/C on the concentration of (dtbbpy)$NiBr_2$.

(1) Similar to the literature procedure, [5-6] the following solution was prepared.

Solution 1: $NiBr_2$(dtbbpy) (48.4 mg, 0.1 mmol) was dissolved in 2 mL of DMA giving a Ni solution of 0.05 M. For each catalyst loading, namely 1 mol %, 3.0 mol %, 5 mol %, 8 mol %, 10 mol % and 15 mol %, the corresponding volumes of solution **1** were 20 µL, 60 µL, 100 µL, 160 µL, 200µL and 300 µL, respectively.

(2) To a flame-dried tube equipped with a stir bar was added a methyl 4-bromobenzoate (21.5 mg, 0.1 mmol,

1.0 equiv). The tube was transferred into an N_2-filled glovebox, to which B_2pin_2 (38 mg, 0.15 mmol, 1.5 equiv), NaI (7.5 mg, 0.05 mmol, 0.5 equiv) and K_2CO_3 (25 mg, 0.18 mmol, 1.8 equiv) were added. After a measured volume of solution **1** containing $NiBr_2$(dtbbpy**)** in DMA was injected via a syringe, didodecane (internal standard, 22 µL, 0.1 mmol) and 6-bromohex-1-ene (0.13 mmol，1.2 equiv) were injected via syringes. The reaction mixture was adjusted to 0.5 mL by addition of corresponding volumes of DMA. Then the mixture was allowed to stir at 60 °C for 24 h under N_2 atmosphere. The reaction was quenched by 2 mL of H_2O, followed by exaction with 2 mL of diethyl ether. The organic layer was taken and transferred to a GC sampling tube via a pipette, and analyzed by GC.

Following the reaction conditions for method A on a 0.10 mmol scale using different catalyst loading (1 mol%, 3 mol%, 5 mol, 8 mol%, 10 mol% and 15 mol%). The results are in favor of a radical chain mechanism as a linear dependence of ratios of L/C on the catalyst loading was observed.

4. Reduction of (dtbbpy)Ni(II)Br_2 (Ni-a) to Ni(0) and preparation of Ni-b

(1) In situ formation of **Ni-b**

a. In the presence of alkyl-Br:

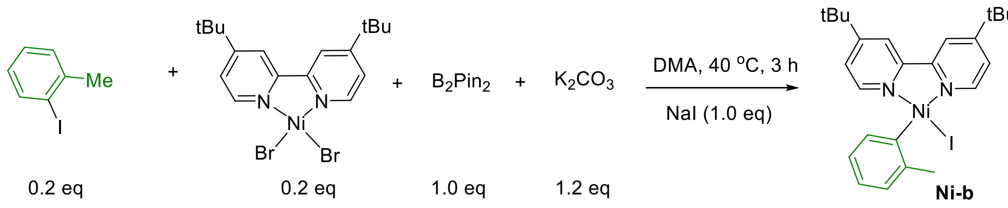

To a flame-dried tube with a stir bar was added $NiBr_2$(dtbbpy) (9.7 mg, 20 mol %). The tube was transferred into an N_2-filled glovebox, B_2pin_2 (25 mg, 0.1 mmol, 1.0 equiv), NaI (15 mg, 1.0 equiv) and K_2CO_3 (18 mg, 1.2 equiv) were added. The tube was sealed, removed from the glovebox and charged with DMA (0.5 mL). The mixture was stirred vigorously, methyl 1-iodo-2-methylbenzene (21.8 mg, 0.1 mmol, 1.0 equiv) and 1-bromododecane (1.2 equiv) were added to the tube. The mixture was stirred vigorously at 40 °C for 3 h. After that, the reaction mixture was filtered through a syringe filter in N_2-filled glovebox and the resultant homogenous solution was used to obtain a non-deuterated solvent (non-D) ^{1}H NMR spectrum (Figure S1-a).

b. in the absence of alkyl-Br

To a flame-dried tube with a stir bar was added $NiBr_2$(dtbbpy) (9.7 mg, 0.02 mmol). The tube was transferred into an N_2-filled glovebox, B_2pin_2 (25 mg, 0.1 mmol, 1.0 equiv), NaI (15 mg, 1.0 equiv) and K_2CO_3 (18 mg, 1.2 equiv) were added. The tube was sealed, removed from the glovebox and charged with DMA (0.5 mL). The mixture was stirred vigorously, methyl 1-iodo-2-methylbenzene (4.4 mg, 0.02 mmol, 0.2 equiv) was added to the tube. The mixture was stirred vigorously at 40 °C for 3 h. After that, the reaction mixture was filtered through a syringe filter in N_2-filled glovebox and the resultant homogenous solution was used directly to obtain a non-deuterated solvent (non-D) ^{1}H NMR spectrum (Figure S1-b).

c. preformed Ni-b in DMA

Based on the ^{1}H NMR spectrum, **Ni-b** is formed whose characteristic peaks are in agreement with those for the pre-prepared **Ni-b** complex in non-D DMA (Figure S1-c).[6]

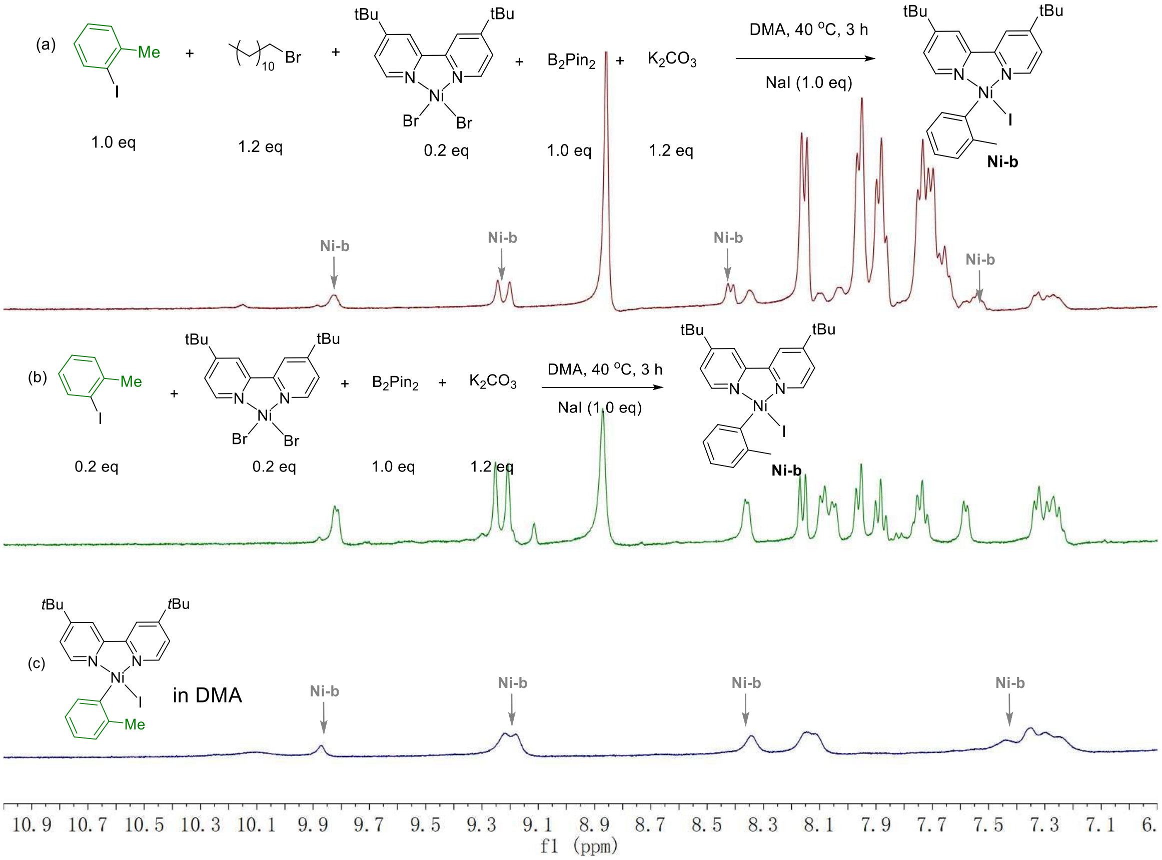

Figure S1 ^{1}H NMR spectra of the reaction mixture of (dtbbpy)$NiBr_2$, B_2Pin_2, Ar^1–I, in the presence or albescence of $nC_{12}H_{25}Br$. (a) in the presence of $nC_{12}H_{25}Br$; (b) in the absence of $nC_{12}H_{25}Br$; (c) pre-prepared **Ni-b** in DMA. Note: Full spectra of the reaction mixture and Ni complexes are available on Page S115-S116.

We also attempted to quantify the yields of Ni-b using triazine as the internal standard. A characteristic peak of triazine in DMA using non-D NMR technique was set at 9.96 ppm (Figure S2). The molar ratio of triazine to **Ni-b** was determined by the ratio of the peak of triazine at 9.96 to that of the doublet of Ni-b from 9.2-9.3 ppm (Figure S3). Accordingly, the yields for the reactions at Figure 5e (also as below in Figure S3) were determined to be 12% (with $nC_{12}H_{25}Br$) and 51% (without $nC_{12}H_{25}Br$), respectively. Full spectra of the reaction mixture and Ni complexes of Figures S2-S4 are available on Page S116-S118.

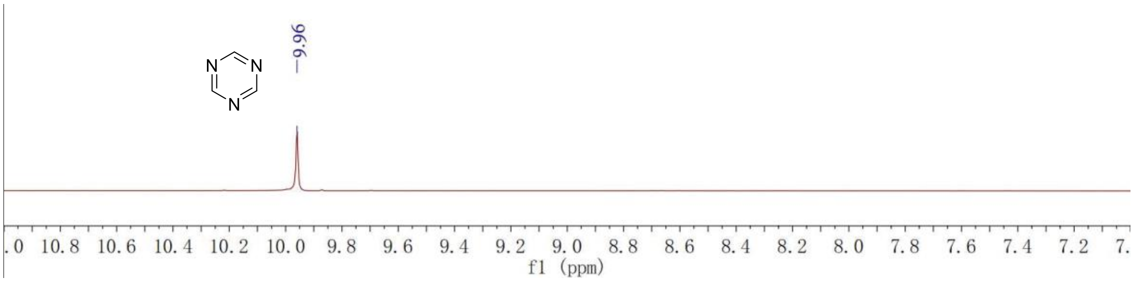

Figure S2. The ^{1}H NMR peak of trazine in DMA obtained with a non-D NMR technique.

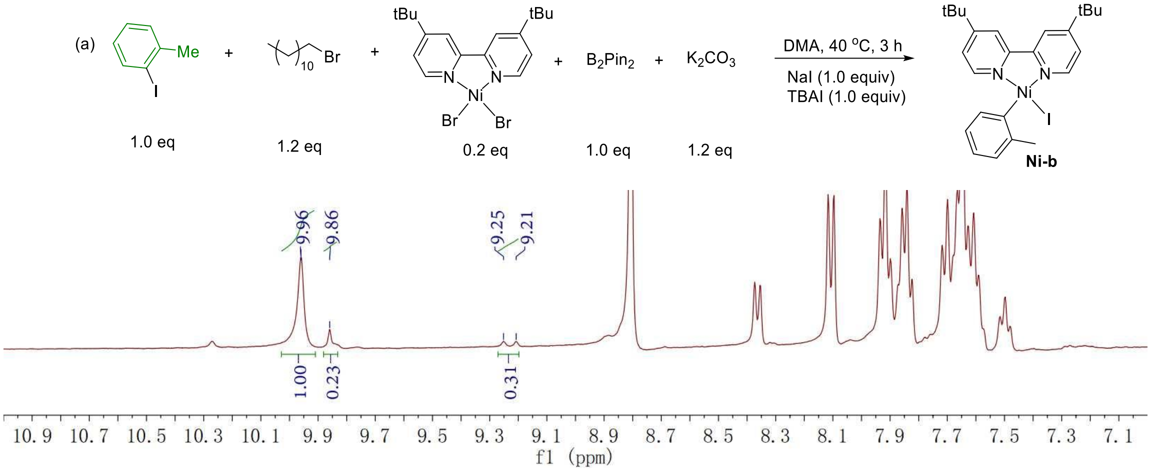

Figure S3. ^{1}H NMR spectra of the reaction mixture of (dtbbpy)$NiBr_2$, B_2Pin_2, Ar^1–I, in the presence or albescence of $nC_{12}H_{25}Br$. Note: 1,3,5-triazine was added after reaction the reaction was run for 3 hours.

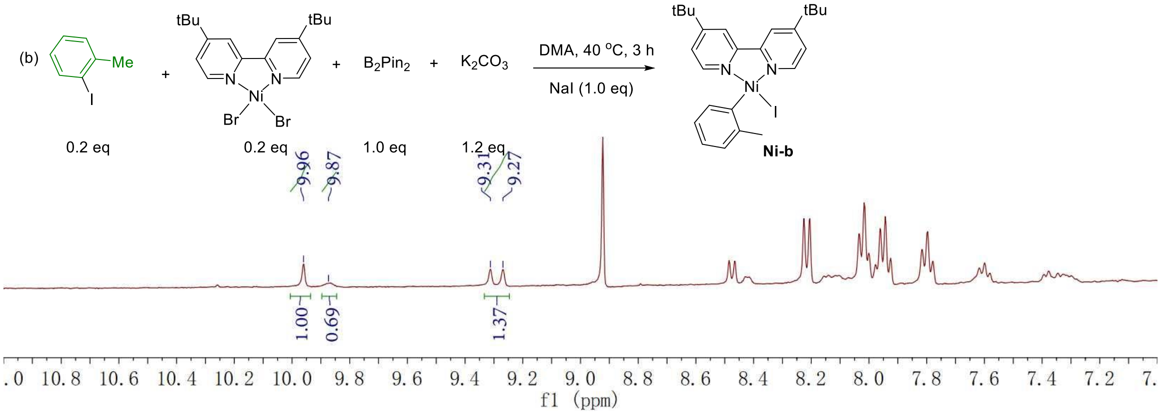

Figure S4. ^{1}H NMR spectra of the reaction mixture of (dtbbpy)$NiBr_2$, B_2Pin_2, Ar^1–I, in the presence or albescence of $nC_{12}H_{25}Br$. Note: 1,3,5-triazine was added after reaction the reaction was run for 3 hours.

(2) In situ formation of **Ni-c** in the presence or absence of alkyl-Br

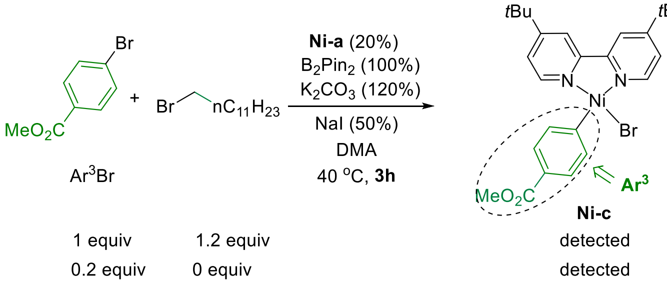

To a flame-dried tube with a stir bar was added 4-bromobenzoate (0.1 mmol, 1.0 equiv) and $NiBr_2$(dtbbpy) (9.7 mg, 20 mol %). The tube was transferred into an N_2-filled glovebox, B_2pin_2 (25 mg, 0.1 mmol, 1.0 equiv), NaI (15 mg, 1.0 equiv) and K_2CO_3 (18 mg, 1.2 equiv) were added. The tube was sealed, removed from glovebox and charged with DMA (0.5 mL). After 1-bromododecane (1.2 equiv if applicable) was added to the tube, the mixture was stirred vigorously at 40 °C for 3 h. After that, a solution was obtained for (non-D) ^{1}H NMR studies upon filtering the reaction mixture through a syringe filter in glovebox.

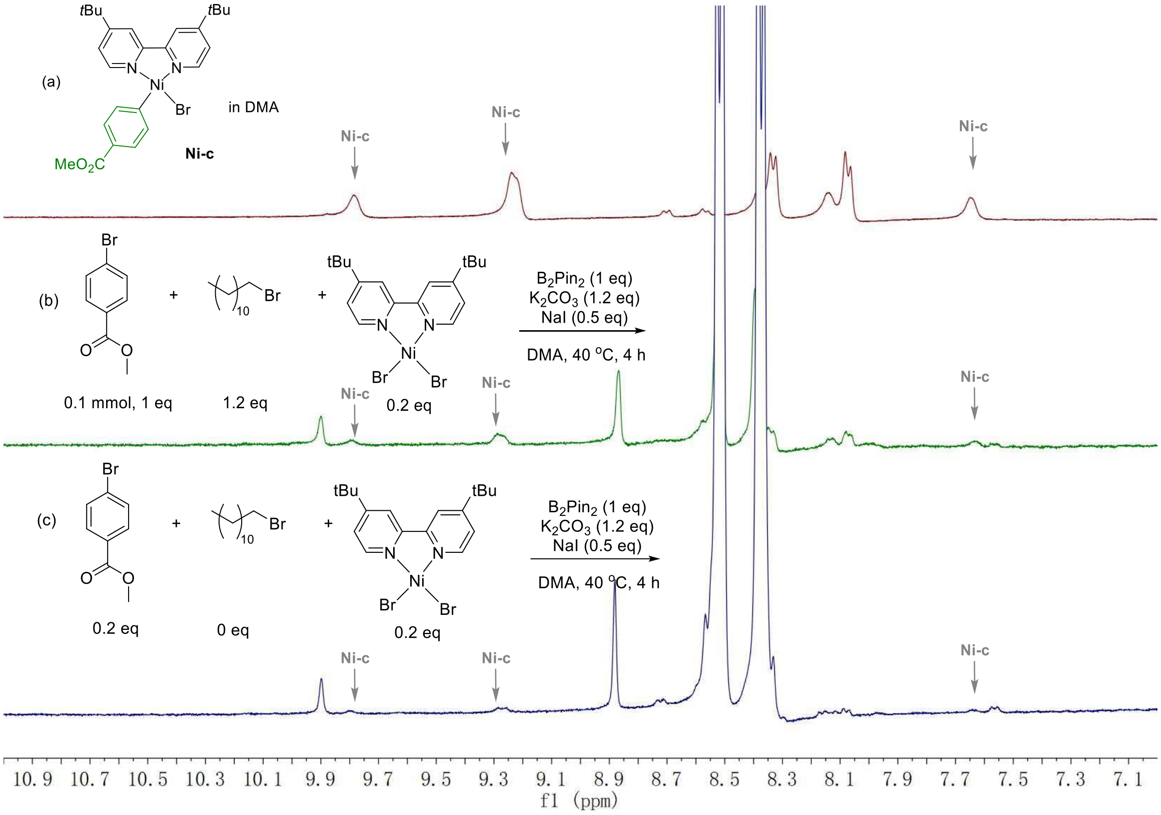

Figure S5. ^{1}H NMR spectra of the reaction mixture of (dtbbpy)$NiBr_2$, B_2Pin_2, Ar^2–Br, in the presence $nC_{12}H_{25}Br$. (a) pre-prepared **Ni-c** in DMA; (b) 0.2 equiv of **S2** and $nC_{12}H_{25}Br$; (c) 1 equiv of **S2** and 1.2 equiv of $nC_{12}H_{25}Br$. Note: Full spectra of the reaction mixture and Ni complexes are available on Page S118-S119

Based on the ^{1}H NMR spectrum, **Ni-c** formed in the absence (Figure S5-a) and presence (Figure S5-b) of alkyl bromide whose characteristic peaks are in agreement with those for the pre-prepared **Ni-c** complex (Figure S2-c) in non-D DMA.

(3) Reduction of (dtbbpy)Ni(II)Br_2 (**Ni-a**) to Ni(0)

To a flame-dried tube equipped with a stir bar was added $NiBr_2$(dtbbpy) (9.7 mg, 20 mol %). The tube was transferred into an N_2-filled glovebox, B_2pin_2 (25 mg, 0.1 mmol, 1.0 equiv), NaI (15 mg, 1.0 equiv) and K_2CO_3 (18 mg, 1.2 equiv) were added. The tube was sealed, removed from the glovebox and charged with DMA (0.5 mL). The mixture was stirred vigorously. After COD (1.0 equiv) was added to the reaction mixture, it was stirred vigorously at 40 °C for 3 h. After that, the reaction mixture was filtered through a syringe filter in N_2-filled glovebox and the resultant homogenous solution was used directly to obtain a non-deuterated solvent (non-D) ^{1}H NMR spectrum.

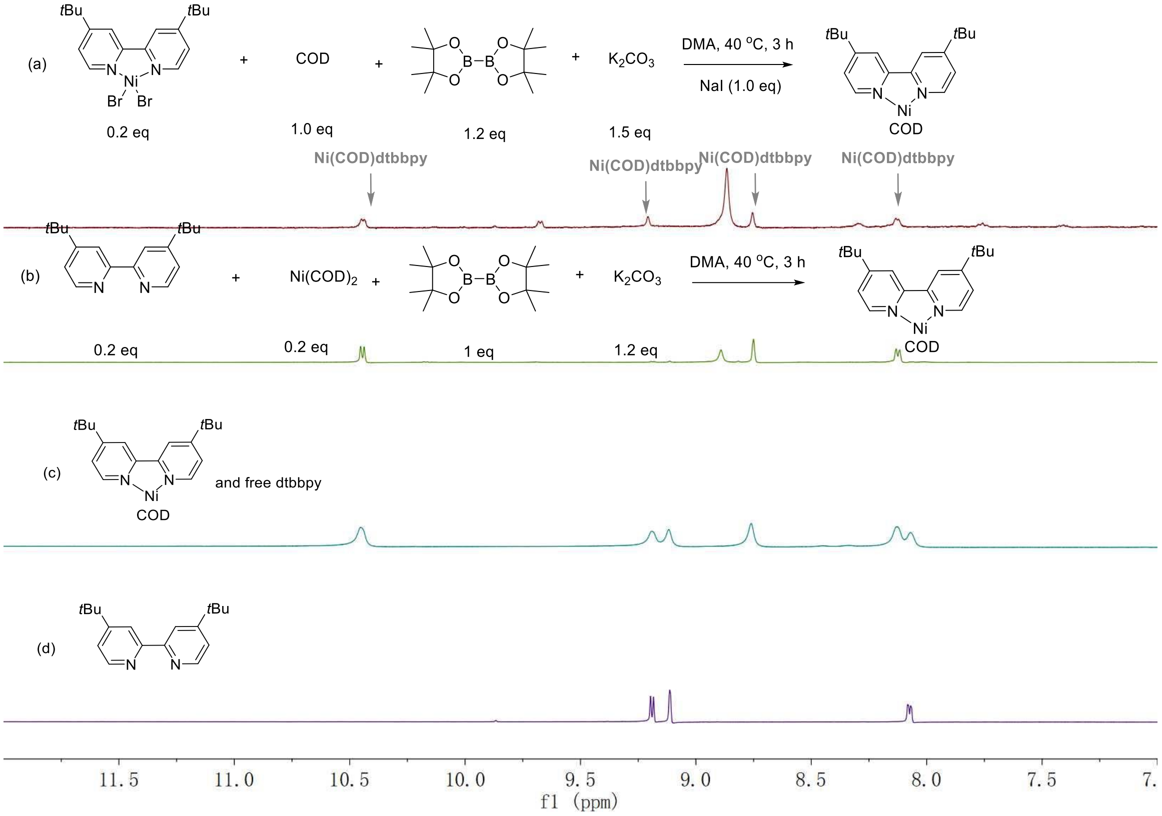

Figure S6 Reduction of (dtbbpy)$NiBr_2$ with B_2Pin_2 in the absence of Ar[1]–I. (a) reaction of (dtbbpy)$NiBr_2$ with B_2Pin_2 and COD; (b) reaction of (dtbbpy)Ni(0)(COD) with B_2Pin_2; (c) (dtbbpy)Ni(0)(COD) in DMA; (d) dtbbpy in DMA. Note: Full spectra of the reaction mixture and Ni complexes are available on Page S119-S121.

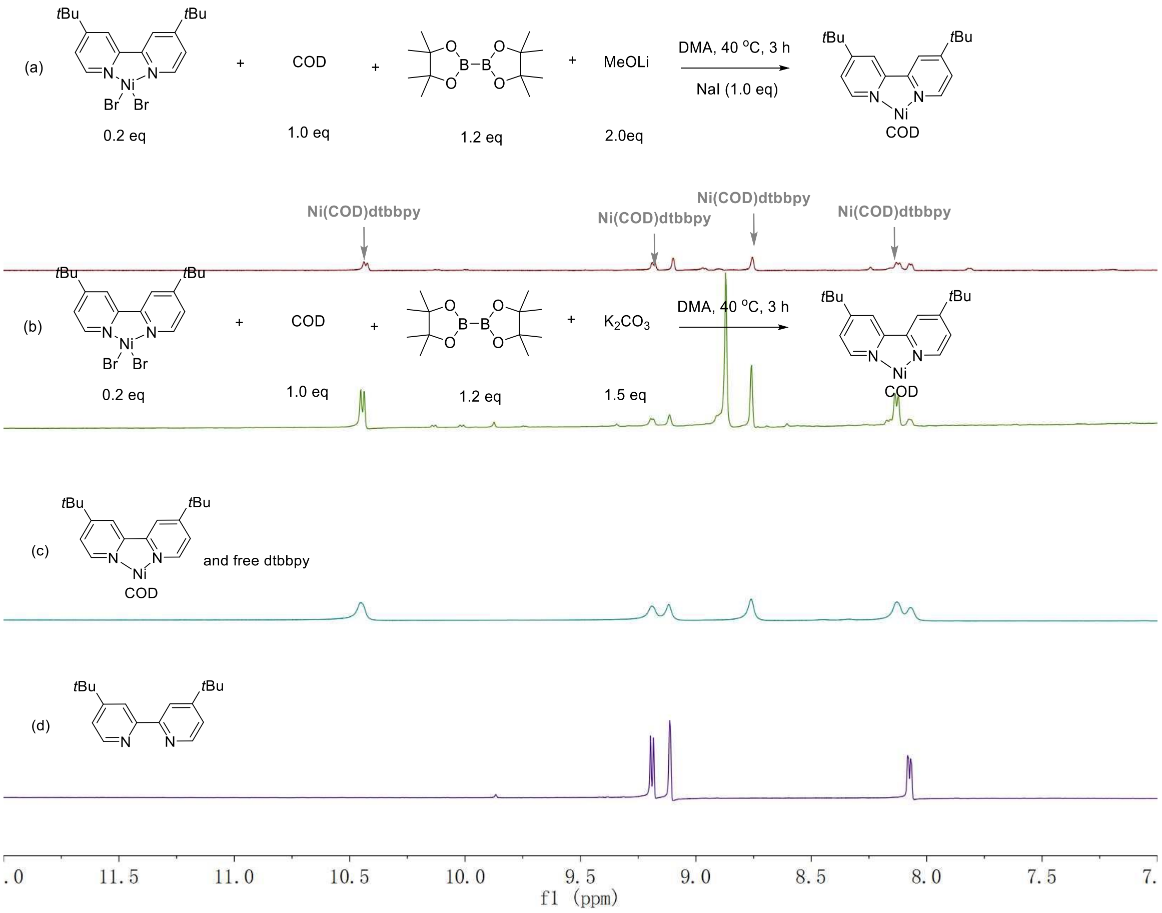

Figure S7. Similar to the conditions run in Figure S6. (a) reaction of (dtbbpy)$NiBr_2$ with B_2Pin_2, MeOLi, NaI and COD; (b) reaction of (dtbbpy)$NiBr_2$ with B_2Pin_2, K_2CO_3 and COD; (c) (dtbbpy)Ni(0)(COD) in DMA; (d) dtbbpy in DMA.

5. Stoichiometric competition reaction

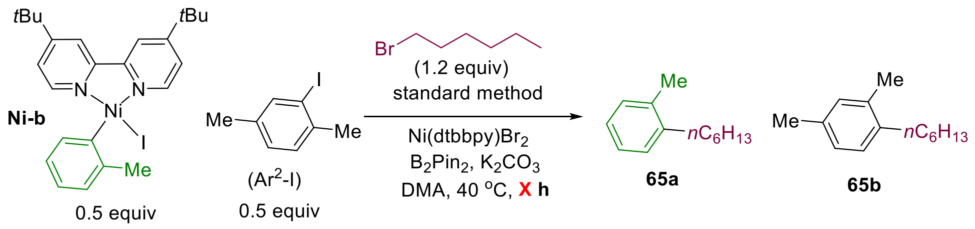

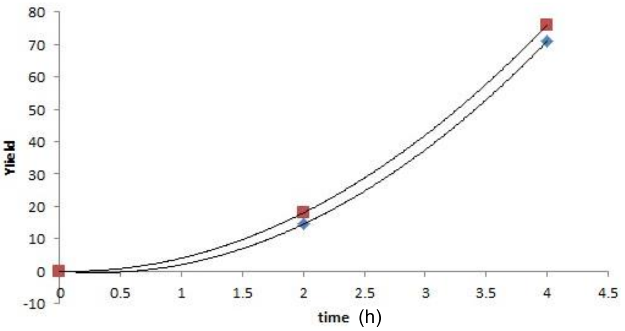

To a flame-dried tube with a stir bar was added $NiBr_2$(dtbbpy) (4 mg, 8 mol %). The tube was transferred into an N_2-filled glovebox, B_2pin_2 (38 mg, 0.15 mmol, 1.5 equiv), NaI (30 mg, 2.0 equiv), **Ni-b** (27.2 mg, 0.05 mmol) and K_2CO_3 (25 mg, 1.8 equiv) were added. The tube was sealed, removed from the glovebox and charged with DMA (0.5 mL). The mixture was stirred vigorously, 1-iodo-2,4-dimethylbenzene (11.6 mg, 0.05 mmol) and 1-bromohexane (1.2 equiv) were added to the tube. The mixture was stirred vigorously at 40 °C for 2 h or 4 h. The reaction was quenched by 2 mL of H_2O, followed by exaction with 2 mL of diethyl ether. The organic layer was taken and transferred to a GC sampling tube via a pipette, and analyzed by GC.

6. The stability of Ni-b in DMA in the presence of B_2Pin_2

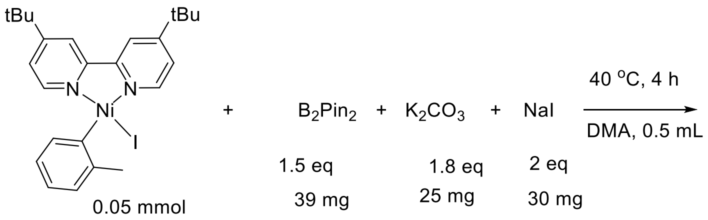

To a flame-dried tube with a stir bar was transferred into an N_2-filled glovebox, B_2pin_2 (39 mg, 0.15 mmol, 1.5 equiv), NaI (30 mg, 2.0 equiv), **Ni-b** (27.2 mg, 0.05 mmol), $NiBr_2$(dtbbpy) (4 mg, 8 mol %, if needed) and K_2CO_3 (25 mg, 1.8 equiv) were added. The tube was sealed, removed from the glovebox and charged with DMA (0.5 mL). The mixture was stirred vigorously at 40 °C for 4 h. After that, the reaction mixture was filtered through a syringe filter in N_2-filled glovebox and the resultant homogenous solution was used directly to obtain a non-deuterated solvent (non-D) ^{1}H NMR spectrum.

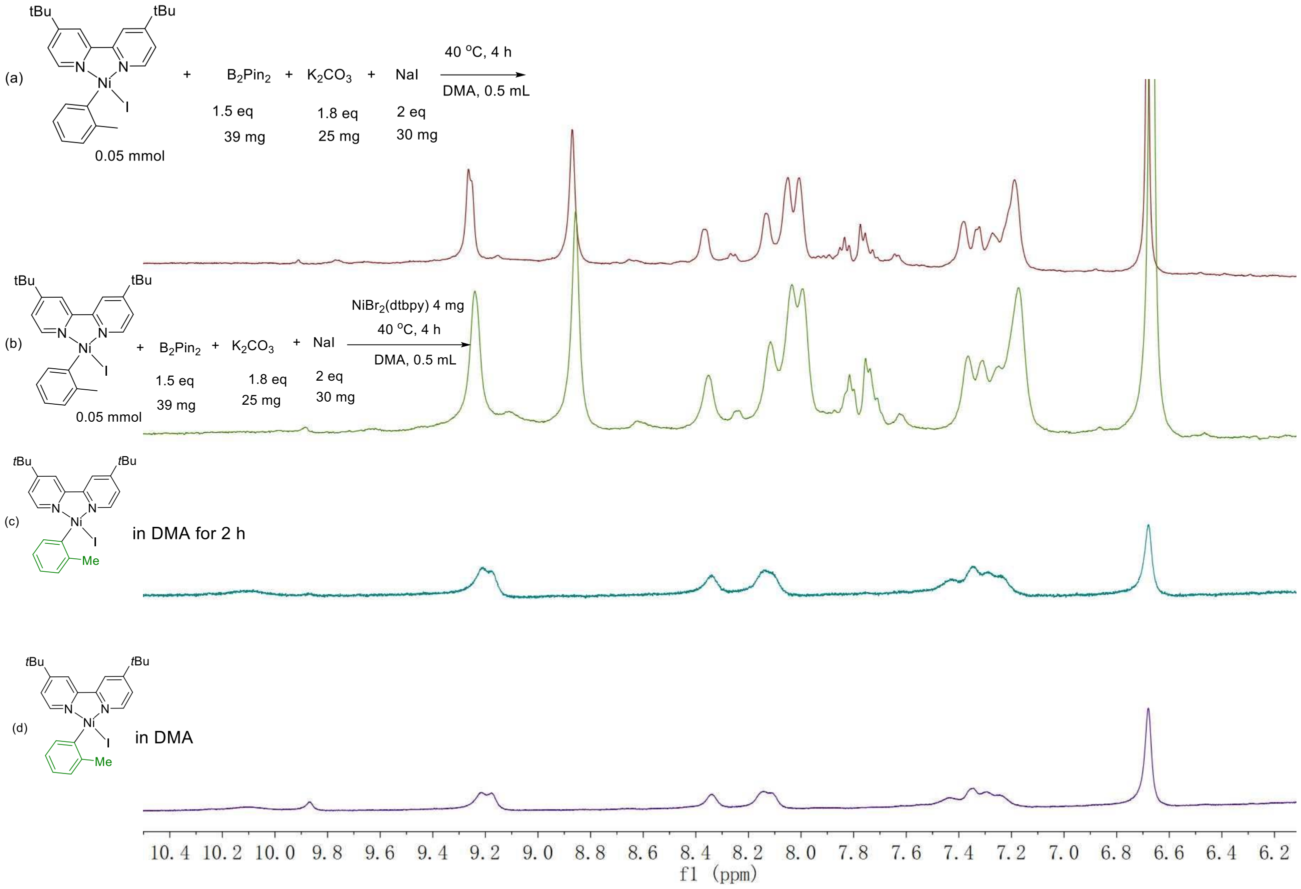

Figure S8. ^{1}H NMR spectra of the stability of **Ni-b** in DMA in the presence of B_2Pin_2/K_2CO_3/NaI. (a) without (dttbpy)$NiBr_2$; (b) with (dttbpy)$NiBr_2$; (c) **Ni-b** in DMA for 2 hours at 40 °C; (d) **Ni-b** in DMA for 10 min at rt. Note: Full spectra of the reaction mixture and Ni complexes are available on Page S121-S122.

7. ^{1}H NMR spectra of the reaction mixture of $[Ni\text{-}d]_2$ with Ar^1-I and B_2Pin_2

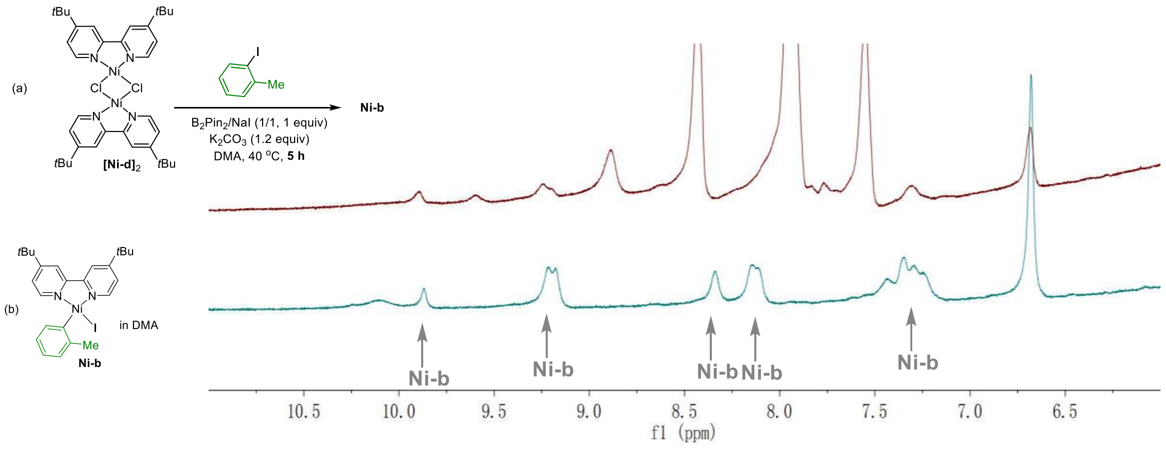

Figure S9 Reduction of Ni(I) complex [**Ni-d**]$_2$ with B_2Pin_2 in the presence of Ar^1–I. (a) A mixture of [**Ni-d**]$_2$ and B_2Pin_2 and Ar^1–I; (b) **Ni-b** in DMA. Note: Full spectra of the reaction mixture and Ni complexes are available on Page S106-S114.

We observed that the reaction of [(dtbbpy)Ni^{I}Cl]$_2$ (**Ni-d**) with Ar^{1}I produced the complex **Ni-b** in detectable NMR yield (Figure S5). Although the detail of this transformation is not clear, it suggests that L_nNi^I can be consumed upon treatment with ArX in the presence of B_2Pin_2.

Part 7. Kinetic studies[19]

1. **General Method for Kinetic Studies:**

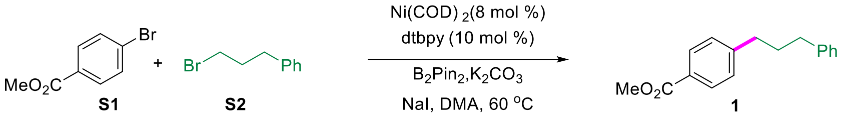

(a) Tube 1: To a flame-dried tube equipped with a stir bar was loaded with 4-bromobenzoate (86 mg, 0.4 mmol, 1.0 equiv), B_2pin_2 (152.4 mg, 0.6 mmol, 1.5 equiv), K_2CO_3 (99.5 mg, 0.72 mmol, 1.8 equiv), and sodium iodide (30 mg, 0.2 mmol, 0.5 equiv). The tube was sealed with a rubber septum, purged with N_2.

(b) Tube 2: To a second flame-dried flask equipped with a stir bar were added Ni(COD)$_2$ (44 mg, 0.16 mmol) and dtbbpy (53.7 mg, 0.2 mmol). The tube was sealed with a rubber septum, and purged with N_2, to which anhydrous DMA (10 mL) was added and the mixture was stirred at r.t. for 1 h.

(c) To the tube 1 was added to 2 mL of the Ni solution by a syringe, followed by addition of **S2** (95.6 mg, 0.48 mmol, 1.2 equiv) and dodecane (47 µL) as an internal standard. The tube was slowly shaken twice to wash down any solids from the walls then placed on a stir plate (at a rate of 1500 rpm). At the appropriate time points, approximately 50 µL of the solution was taken by a syringe (Note: the syringe and its needle were pre-flushed with N_2), which was loaded onto a short silica plug (~ a height of 1 cm) in a plastic pipette packed with cotton, washed with EtOAc (~2 mL) to give a solution that was directly used for GC-MS analysis.

(d) All data runs obtained from the GC-FID instrument were appropriately integrated for the product and the dodecane standard. The integrated data points were further processed by normalizing each product area value by its corresponding standard area value. The normalized areas were then converted to concentration by using calculated response factors obtained from preparing known mixtures of the standard and purified reaction product. Each reaction was analyzed and graphed to show the product concentration (M) as a function of reaction time (min). All data points were plotted with black markers (●) as shown below, while only the data points included in the linear fit are shown with red markers (●). The best-fit linear regression line is shown and the y=mx+b equation is given.

Note: The molar ratio of [K_2CO_3] /[B_2Pin_2] is 6:5 throughout the kinetic studies.

(e) Tracking the reaction progress for the coupling of **S1** and **S2** with method A:

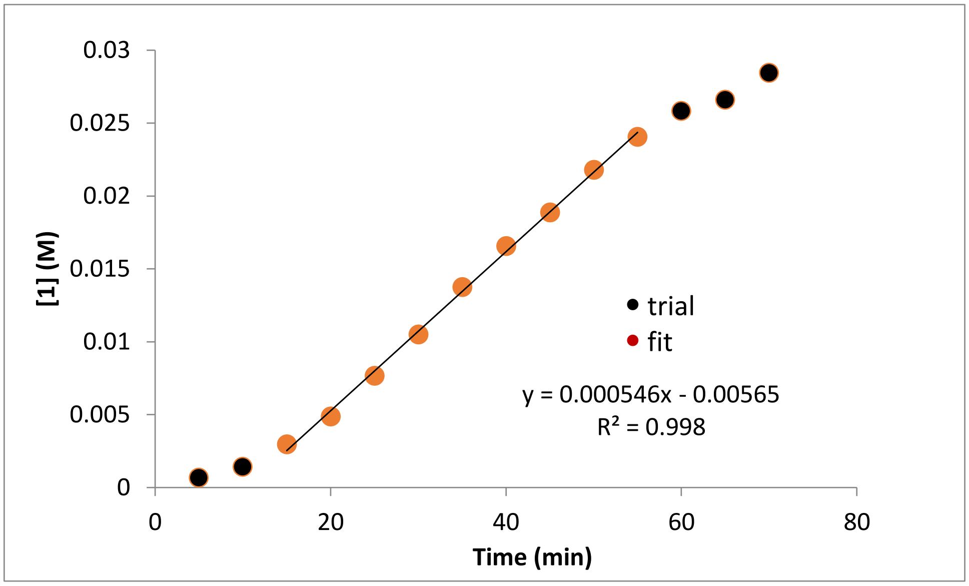

Figure S10. Results of standard reaction conditions runs with linear fit regions highlighted: $[S1]_0$ = 0.2M, $[S2]_0$ = 0.24 M, [L·Ni] = 0.016 M, [B_2Pin_2] = 0.3 M.

2. Variation of the concentrations of [L_n-Ni]

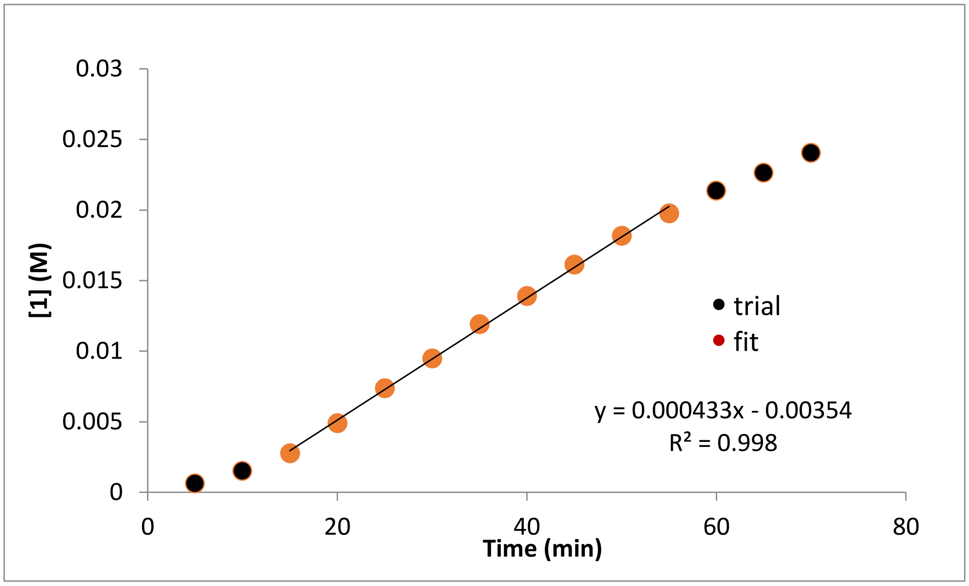

Figure S11. $[S1]_0$ = 0.2 M, $[S2]_0$ = 0.24 M, [L·Ni] = 0.08 M, [B_2Pin_2] = 0.3 M.

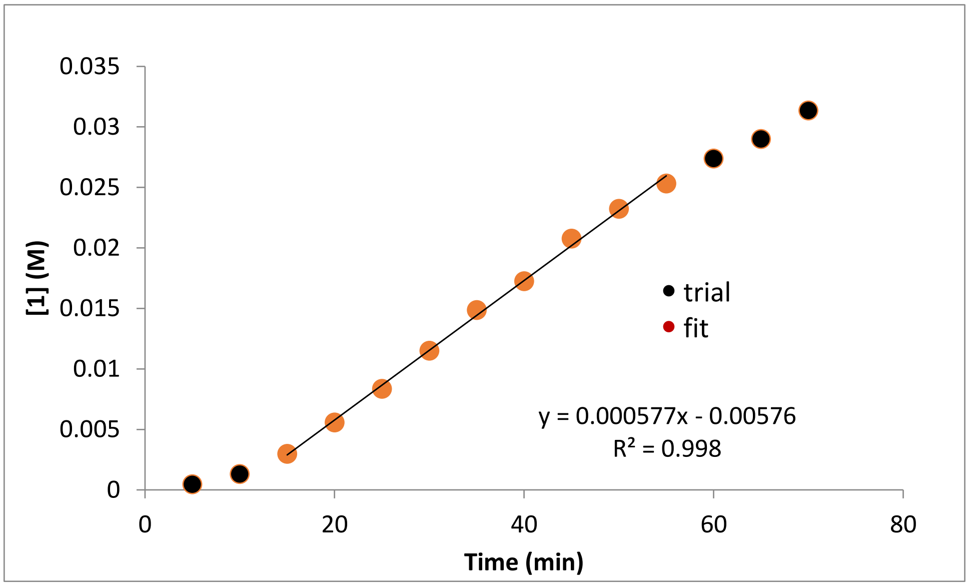

Figure S12. $[S1]_0$ = 0.2 M, $[S2]_0$ = 0.24 M, [L·Ni] = 0.32 M, [B_2Pin_2] = 0.3 M.

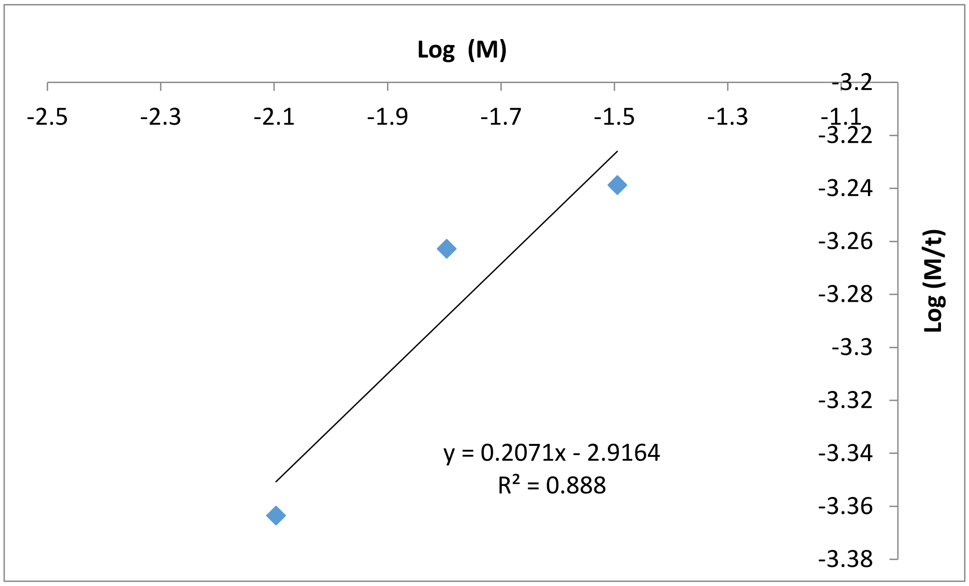

Figure S13. Reaction order of [Ni] for the C(sp^3)–C(sp^2) coupling reaction.

3. Variation of the concentrations of [B_2Pin_2 + K_2CO_3]

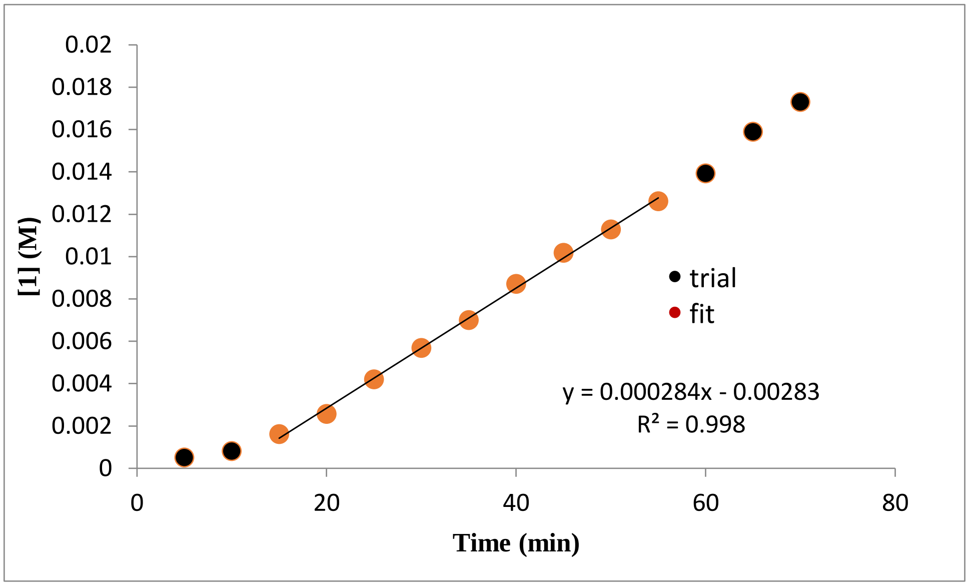

Figure S14. $[S1]_0$ = 0.2 M, $[S2]_0$ = 0.24 M, [L·Ni] = 0.16 M, $[B_2Pin_2]$ = 0.2 M.

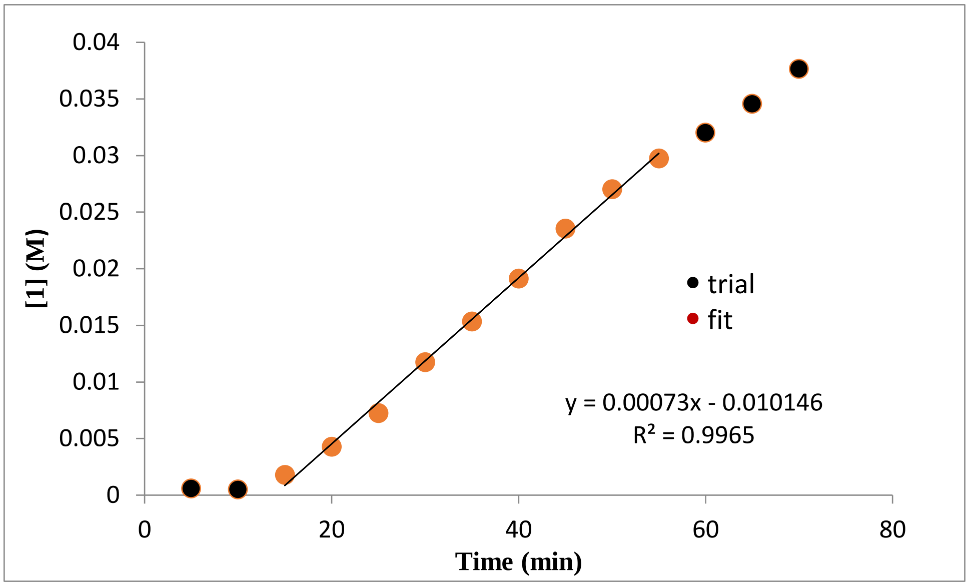

Figure S15. $[S1]_0$ = 0.2 M, $[S2]_0$ = 0.24 M, [L·Ni] = 0.16 M, $[B_2Pin_2]$ = 0.4 M.

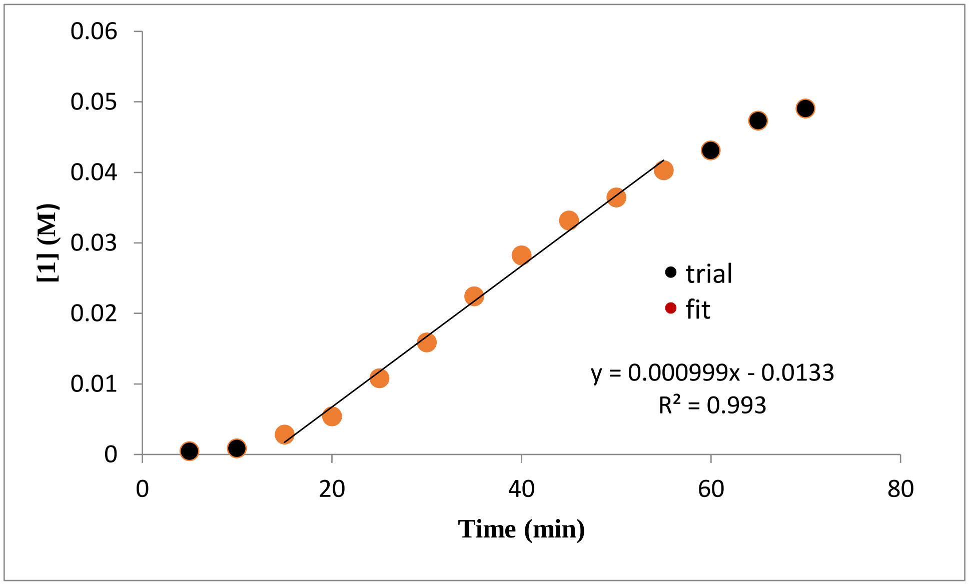

Figure S16. $[S1]_0$ = 0.2 M, $[S2]_0$ = 0.24 M, [L·Ni] = 0.16 M, $[B_2Pin_2]$ = 0.6 M.

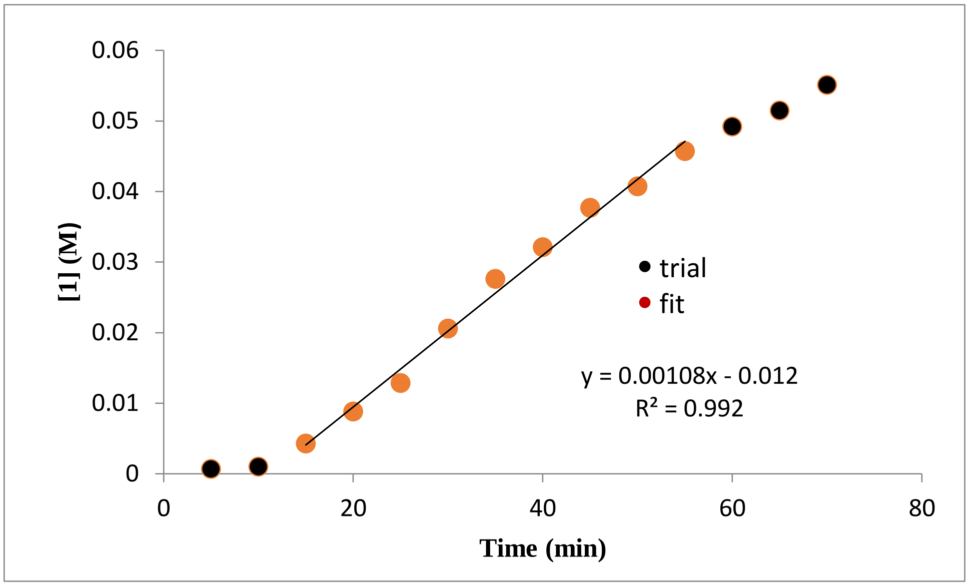

Figure S17. $[S1]_0$ = 0.2 M, $[S2]_0$ = 0.24 M, [L·Ni] = 0.16 M, $[B_2Pin_2]$ = 0.8 M.

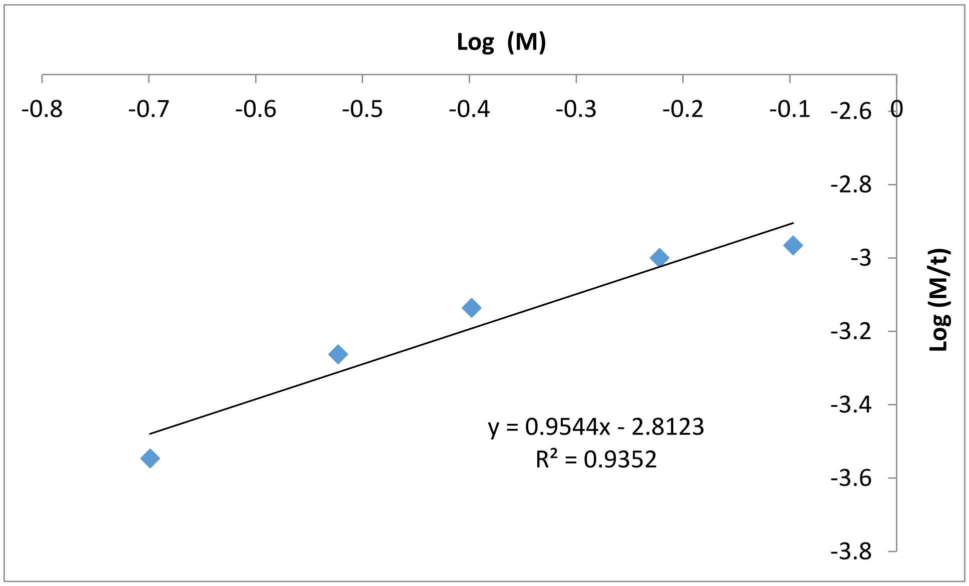

Figure S18. Reaction order of $[B_2Pin_2]$ for the $C(sp^3)$–$C(sp^2)$ coupling reaction.

4. Variation of the concentrations of [S1]

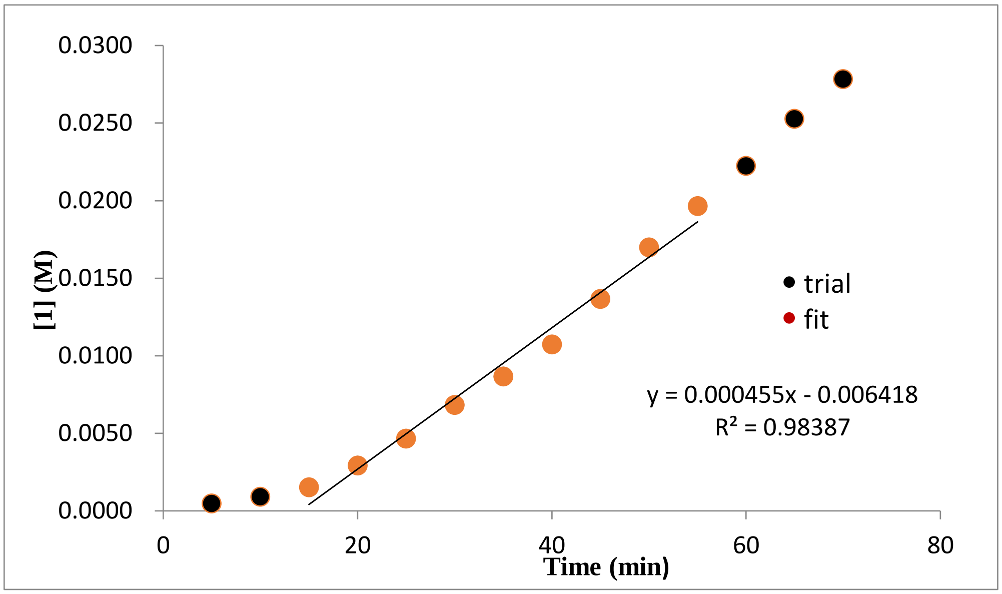

Figure S19. $[S1]_0 = 0.3$ M, $[S2]_0 = 0.24$ M, [L·Ni] = 0.16 M, $[B_2Pin_2] = 0.3$ M.

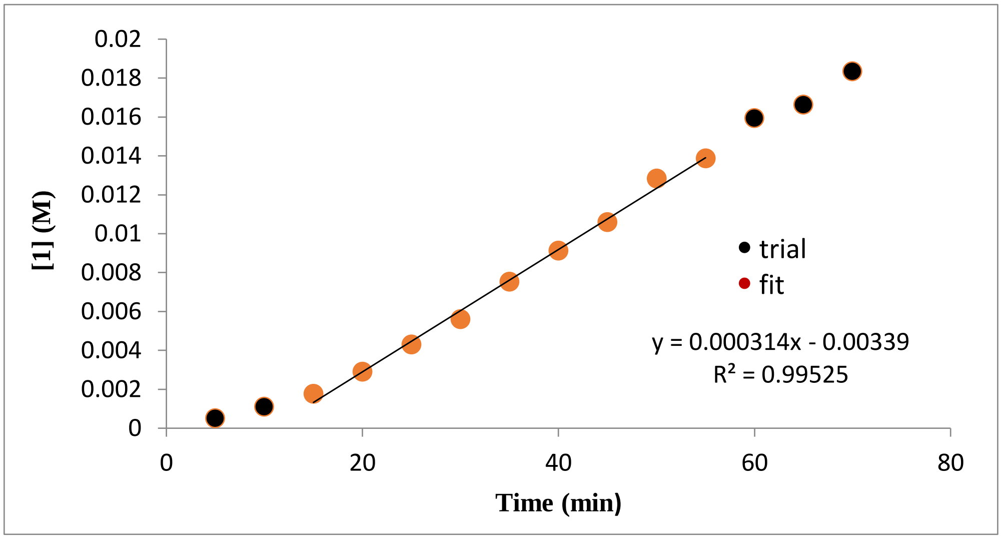

Figure S20. $[S1]_0 = 0.4$ M, $[S2]_0 = 0.24$ M, [L·Ni] = 0.16 M, $[B_2Pin_2] = 0.3$ M.

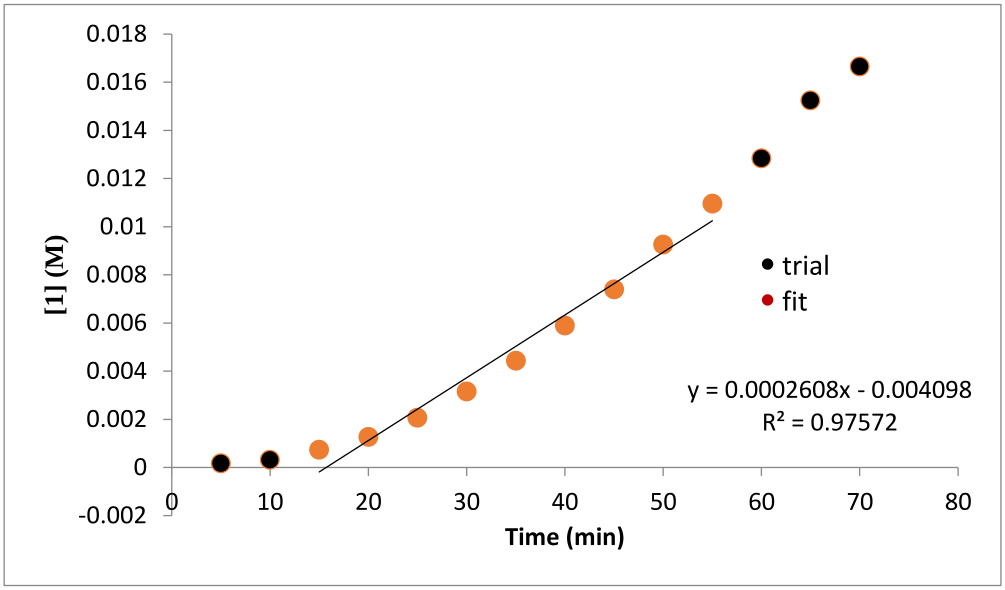

Figure S21. $[S1]_0$ = 0.6 M, $[S2]_0$ = 0.24 M, [L·Ni] = 0.16 M, $[B_2Pin_2]$ = 0.3 M.

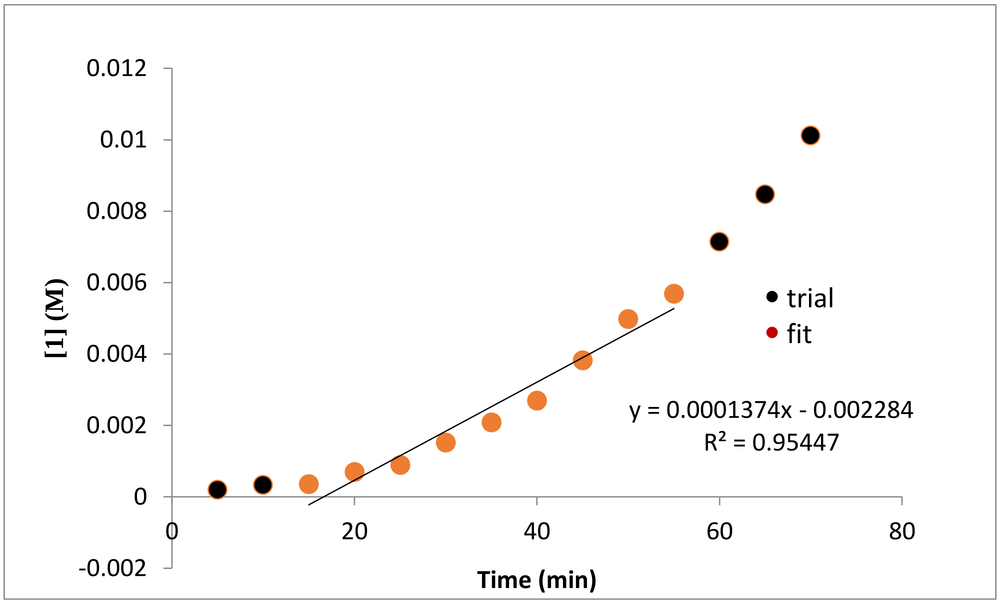

Figure S22. $[S1]_0$ = 0.8M, $[S2]_0$ = 0.24 M, [L·Ni] = 0.16 M, $[B_2Pin_2]$ = 0.3 M.

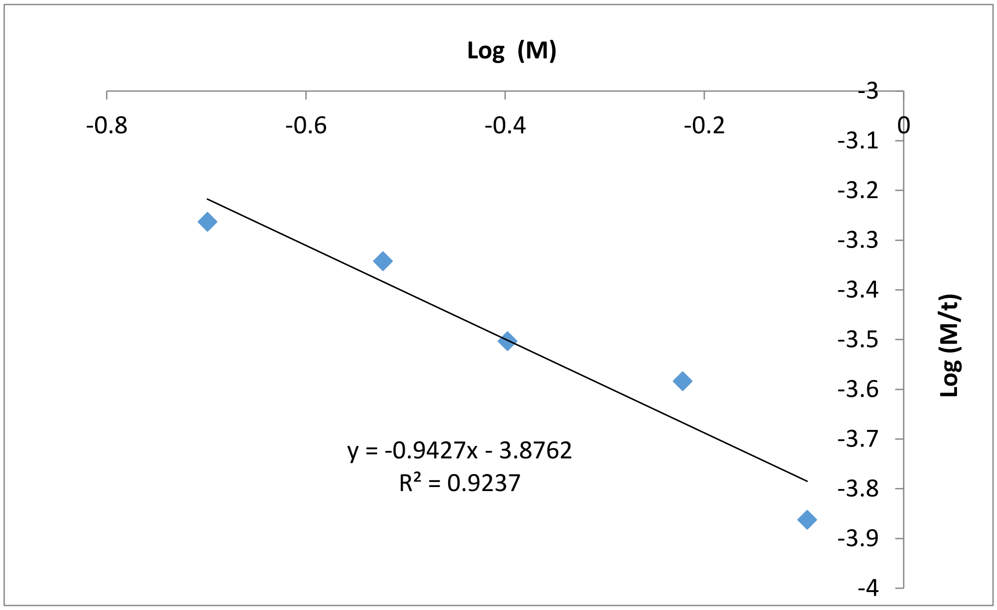

Figure S23. Reaction order of [**S1**] for the C(sp^3)–C(sp^2) coupling reaction.

5. Variation of the concentrations of [S2]

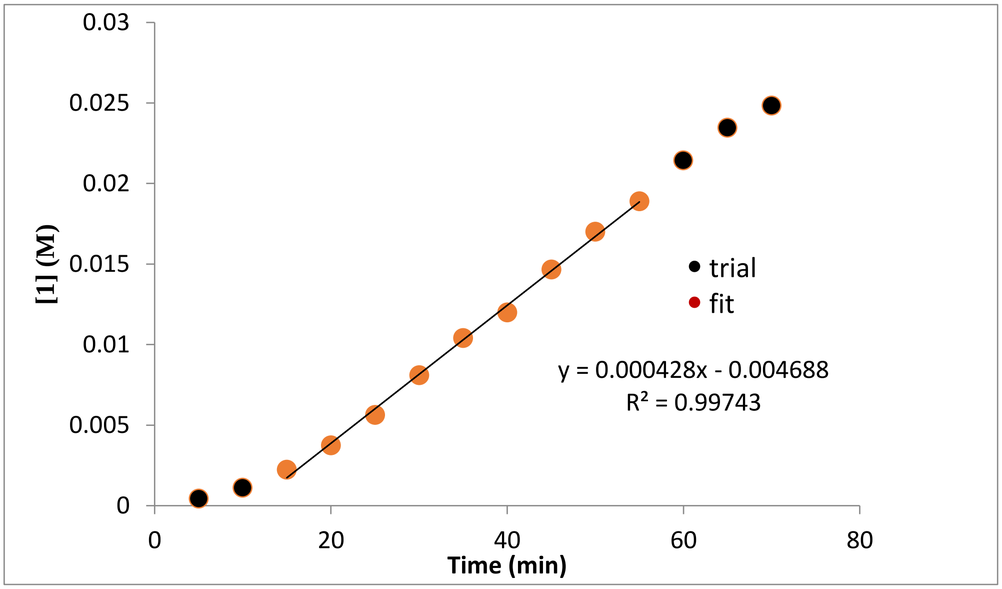

Figure S24. $[S1]_0$ = 0.2 M, $[S2]_0$ = 0.36 M, [L·Ni] = 0.16 M, $[B_2Pin_2]$ = 0.3 M.

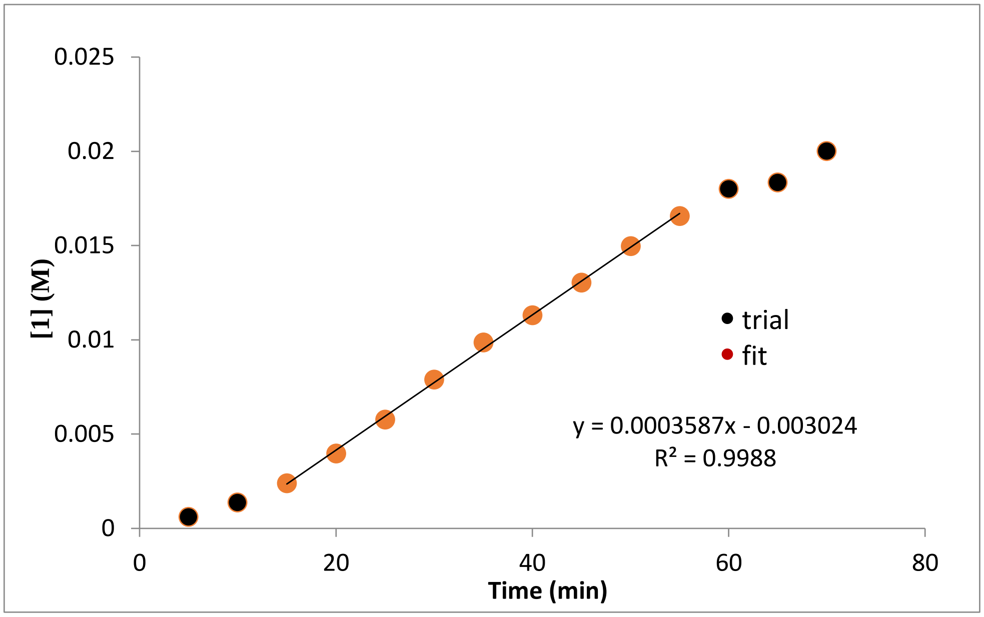

Figure S25. $[S1]_0 = 0.2$ M, $[S2]_0 = 0.48$ M, [L·Ni] = 0.16 M, $[B_2Pin_2] = 0.3$ M.

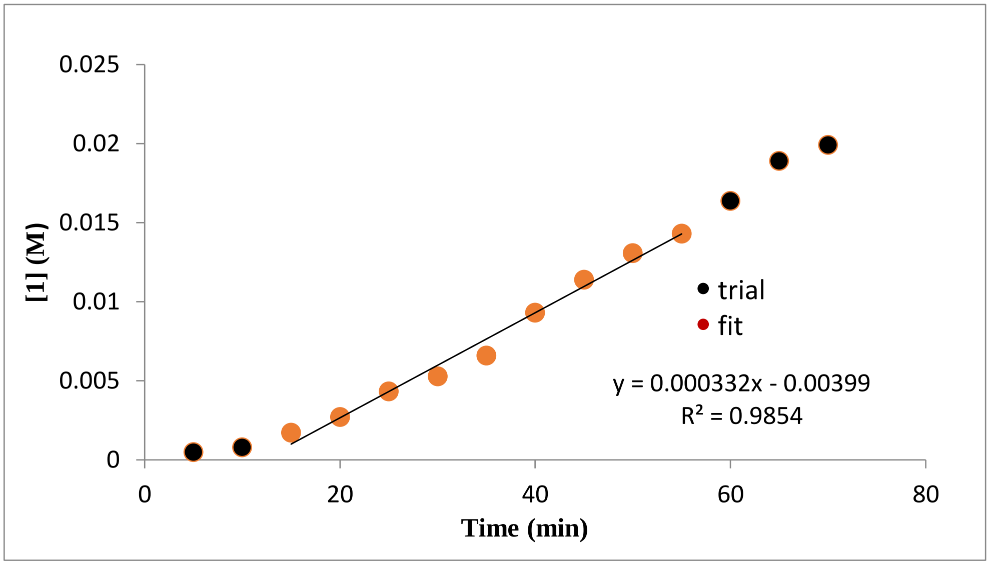

Figure S26. $[S1]_0 = 0.2$ M, $[S2]_0 = 0.72$ M, [L·Ni] = 0.16 M, $[B_2Pin_2] = 0.3$ M.

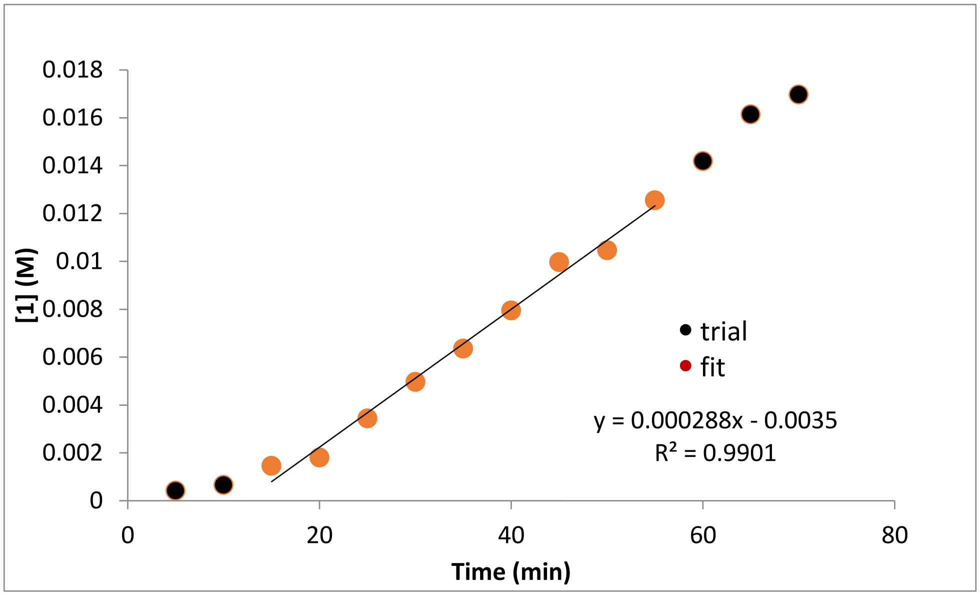

Figure S27. $[S1]_0$ = 0.2 M, $[S2]_0$ = 0.96 M, [L·Ni] = 0.16M, $[B_2Pin_2]$ = 0.3M.

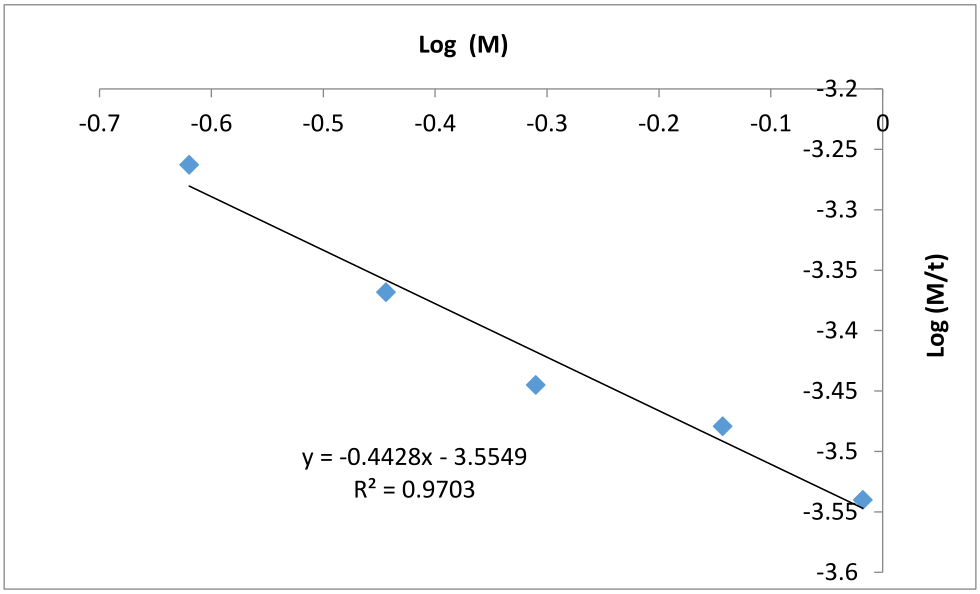

Figure S28. Reaction order of [**S2**] for the C(sp^3)–C(sp^2) coupling reaction.

II. Reference

1. Anka-Lufford, L. L.; Huihui, K. M.; Gower, N. J.; Ackerman, L. K.; and Weix, D. J. Nickel-Catalyzed Cross-Electrophile Coupling with Organic Reductants in Non-Amide Solvents. *Chem. Eur. J.* **2016**, *22*, 11564.
2. Jezorek, R. L.; Zhang, N.; Leowanawat, P.; Bunner, M. H.; Gutsche, N.; Pesti, A. K. R.; Olsen, J. T.; Percec, V. Air Stable Nickel Precatalysts for Fast and Quantitative Cross Coupling of Aryl Sulfamates with Aryl Neopentylglycolboronates at Room Temperature. *Org. Lett.* **2014**, *16*, 6326.
3. Basch, C. H.; Liao, J. N.; Xu, J. Y.; Piane, J. J.; Watson, M. P. Harnessing Alkyl Amines as Electrophiles for Nickel-Catalyzed Cross Couplings via C–N Bond Activation. *J. Am. Chem. Soc.* **2017**, *139*, 5313.
4. Lin, Q.; Ma, G.; Gong, H. Ni-catalyzed Formal Cross-Electrophile Coupling of Alcohols with Aryl Halides. *Acs. Catal.* **2021**, *11*, 14102.
5. Zhao, C.; Jia, X.; Wang, X.; Gong, H. Ni-Catalyzed Reductive Coupling of Alkyl Acids with Unactivated Tertiary Alkyl and Glycosyl Halides. *J. Am. Chem. Soc.* **2014**, *136*, 17645.
6. Biswas, S.; Weix. D. J. Mechanism and Selectivity in Nickel-Catalyzed Cross-Electrophile Coupling of Aryl Halides with Alkyl Halides. *J. Am. Chem. Soc.* **2013**, *135*, 16192.
7. Perkins, R. J.; Hughes, A. J.; Weix, D. J.; Hansen, E. C. Metal-Reductant-Free Electrochemical Nickel -Catalyzed Couplings of Aryl and Alkyl Bromides in Acetonitrile. *Org. Process Res. Dev.* **2019**, *23*, 1746.
8. Kondolff, I.; Doucet, H.; Santelli, M. Direct Method for Carbon–Carbon Bond Formation: The Functional Group Tolerant Cobalt-Catalyzed Alkylation of Aryl Halides. *Chem. Eur. J.* **2010**, *16*, 5848.
9. Amatore, M.; Gosmini, C. Direct Method for Carbon–Carbon Bond Formation: The Functional Group Tolerant Cobalt-Catalyzed Alkylation of Aryl Halide. *Chem. Eur. J.* **2010**, *16*, 5848.
10. Zeng, Y.; Qian, B.; Li, Y.; Bao, H. A Metal-Free Approach for Brønsted Acid Promoted C–H Alkylation of Heteroarenes with Alkyl Peroxides. *Synthesis* **2018**, *50*, 3250.
11. Lv, L.; Li, C-J. Ruthenium catalyzed β-selective alkylation of vinylpyridines with aldehydes/ketones via N_2H_4 mediated deoxygenative couplings. *Chem. Sci.* **2021**, *12*, 2870.

12. Hansen, E. C.; Li, C.; Yang, S.; Pedro, D.; Weix, D. J. Coupling of Challenging Heteroaryl Halides with Alkyl Halides via Nickel-Catalyzed Cross-Electrophile Coupling. *J. Org. Chem*. **2017**, *82*, 7085.
13. Duan, J.; Du, Y.-F.; Pang, X.; Shu, X.-Z. Ni-catalyzed cross-electrophile coupling between vinyl/aryl and alkyl sulfonates: synthesis of cycloalkenes and modification of peptides. *Chem. Sci*. **2019**, *10*, 8706.
14. Wang, J.; Ehehalt, L. E.; Huang, Z.; Beleh, O. M.; Guzei, I. A.; Weix, D. J. Formation of C(sp^2)–C(sp^3) Bonds Instead of Amide C–N Bonds from Carboxylic Acid and Amine Substrate Pools by Decarbonylative Cross-Electrophile Coupling. *J. Am. Chem. Soc*. **2023**, *145*, 9951.
15. Luo, S.; Peng, M.; Querard, P.; Li, C.-C.; Li, C-J. Copper-Catalyzed Conjugate Addition of Carbonyls as Carbanion Equivalent via Hydrazones. *J. Org. Chem*. **2021**, *86*, 13111.
16. Kondolff, I.; Doucet, H.; Santelli, M. Direct Synthesis of Protected Arylacetaldehydes by Tetrakis(phosphane)palladium-Catalyzed Arylation of Ethyleneglycol Vinyl Ether. *Eur. J. Org. Chem*. **2006**, 765.
17. Luridiana, A.; Mazzarella, D.; Capaldo, L.; Rincón, J. A.; García-Losada, P.; Mateos, C.; Frederick, M. O.; Nuño, M.; Jan Buma, W.; Noël, T. The Merger of Benzophenone HAT Photocatalysis and Silyl Radical-Induced XAT Enables Both Nickel-Catalyzed Cross-Electrophile Coupling and 1,2-Dicarbofunctionalization of Olefins. *ACS Catal*. **2022**, *12*, 11216.
18. Yang, G.; Wang, Y.; Qiu, Y. Electroreductive Cross-Electrophile Coupling of Aziridines and Aryl Bromides. *Chem. Eur. J*. **2023**, *29*, e202300959.
19. Turro, R.F.; Wahlman, J. L. H.; Tong, Z. J.; Chen, X.; Yang, M.; Chen, E. P.; Hong, X.; Hadt, R. G.; Houk, K. N.; Yang, Y.-F.; Reisman, S. E. Mechanistic Investigation of Ni-Catalyzed Reductive Cross-Coupling of Alkenyl and Benzyl Electrophiles. *J. Am. Chem. Soc*. **2023**, *145*, 14705.
20. Sun, C.-L.; Krause,H; Fürstner, A. A Practical Procedure for Iron-Catalyzed Cross-Coupling Reactions of Sterically Hindered Aryl-Grignard Reagents with Primary Alkyl Halides. *Adv.Synth.Catal*.**2014**, *356*, 1281.

III. NMR Data

1a, ^{1}H-NMR (600 MHZ, $CDCl_3$)

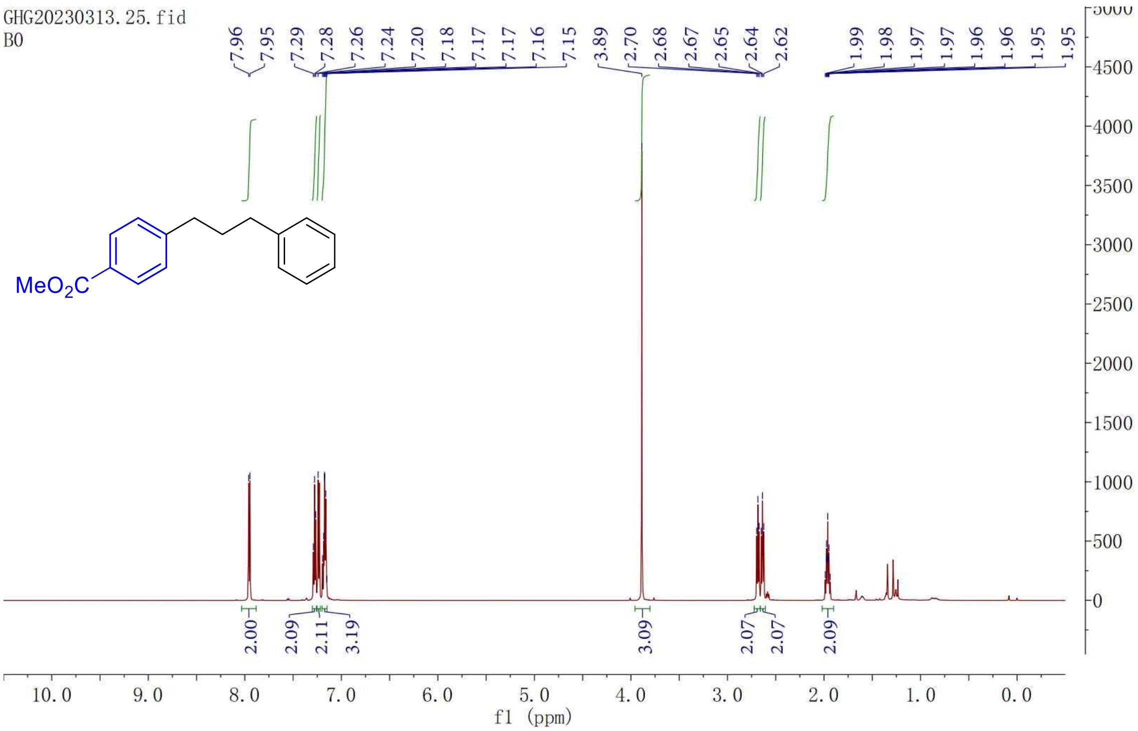

1a, ^{13}C NMR (151 MHz, $CDCl_3$)

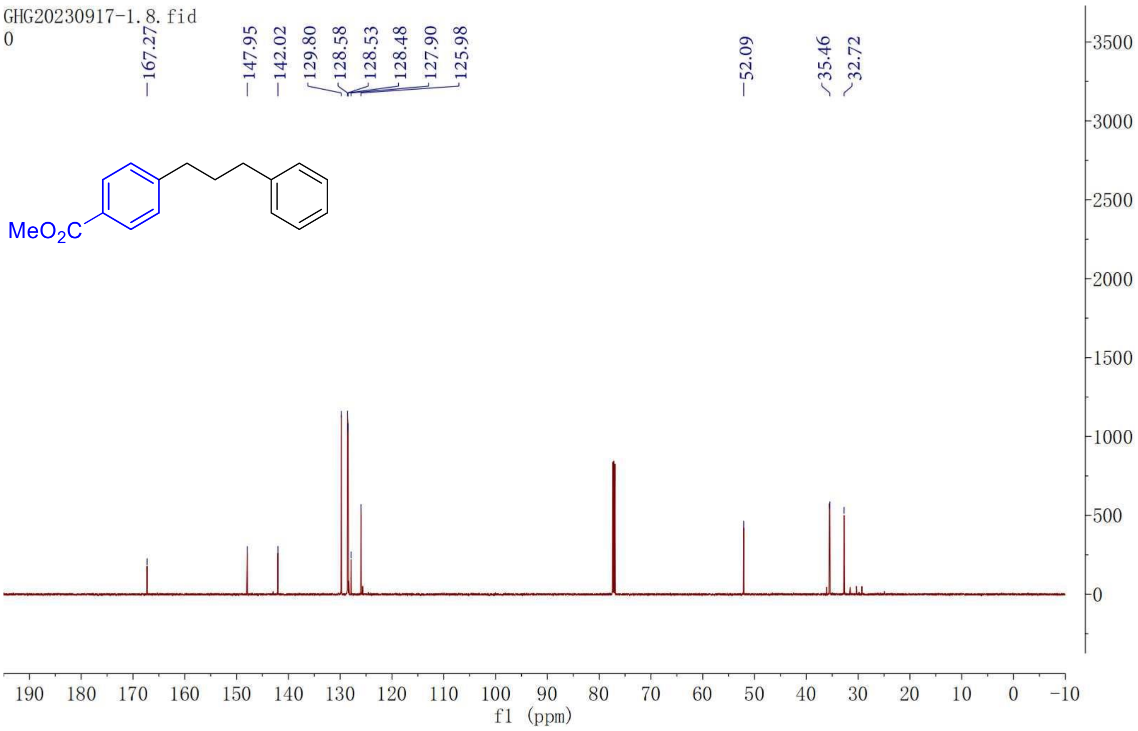

1b, ^{1}H-NMR (600 MHZ, $CDCl_3$)

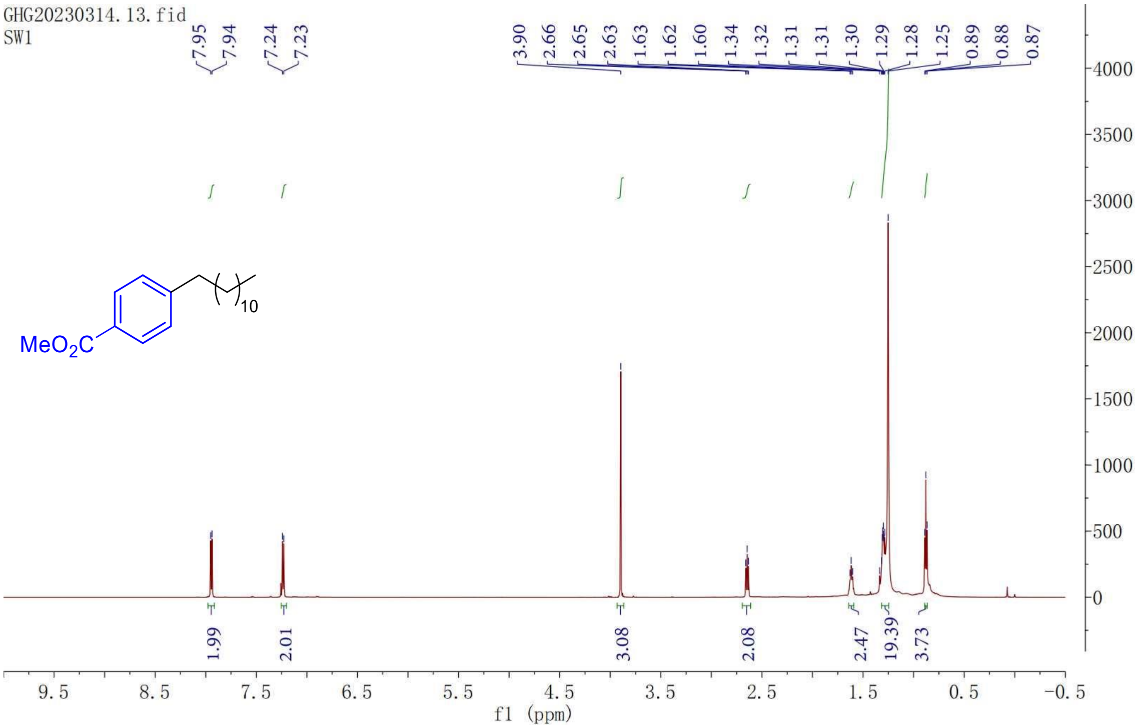

1b, ^{13}C NMR (151 MHz, $CDCl_3$)

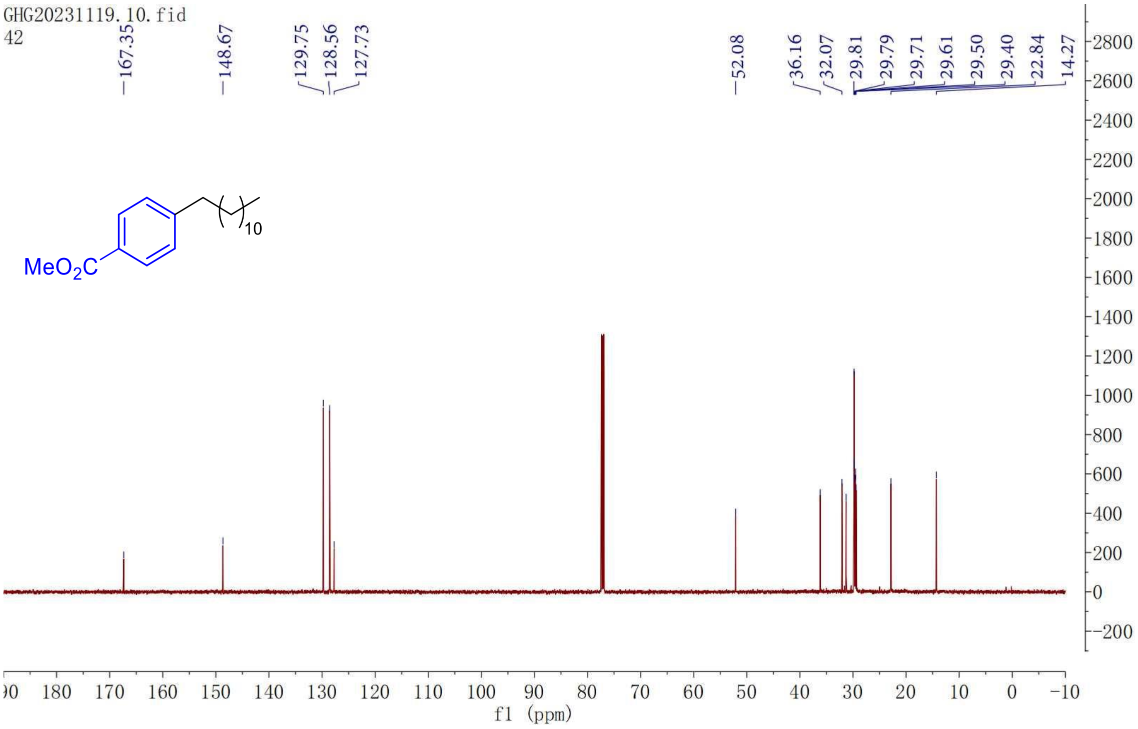

2, ^{1}H-NMR (600 MHZ, $CDCl_3$)

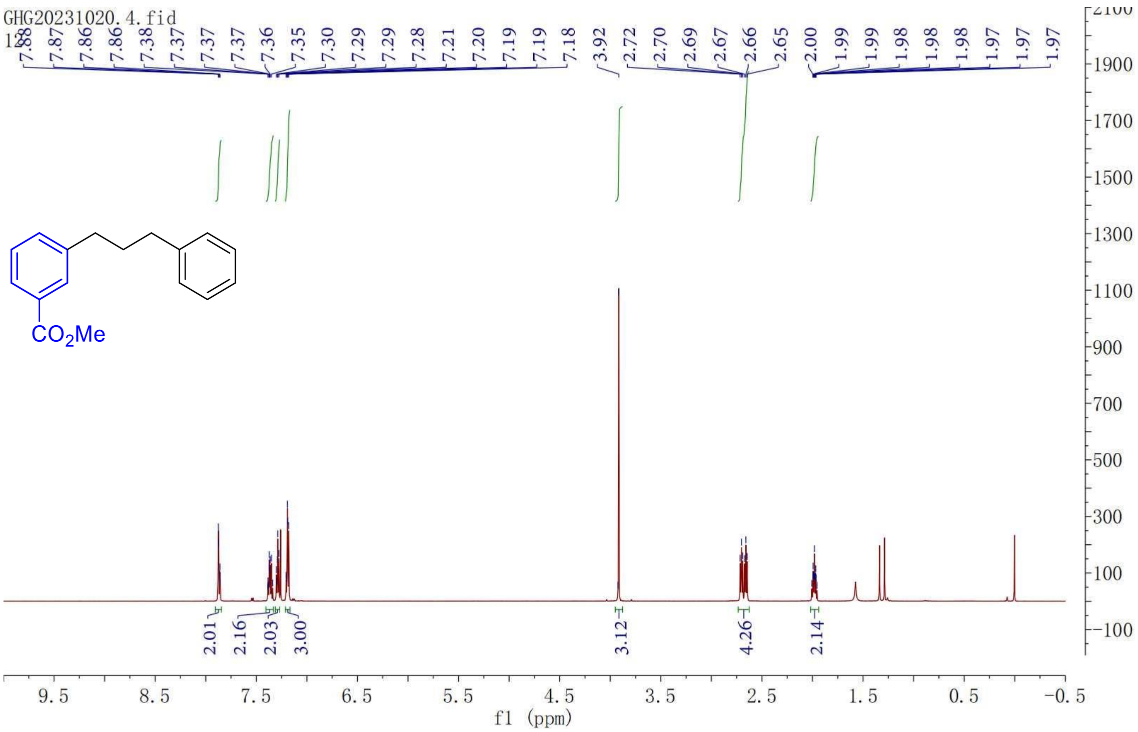

3, ^{1}H-NMR (600 MHZ, $CDCl_3$)

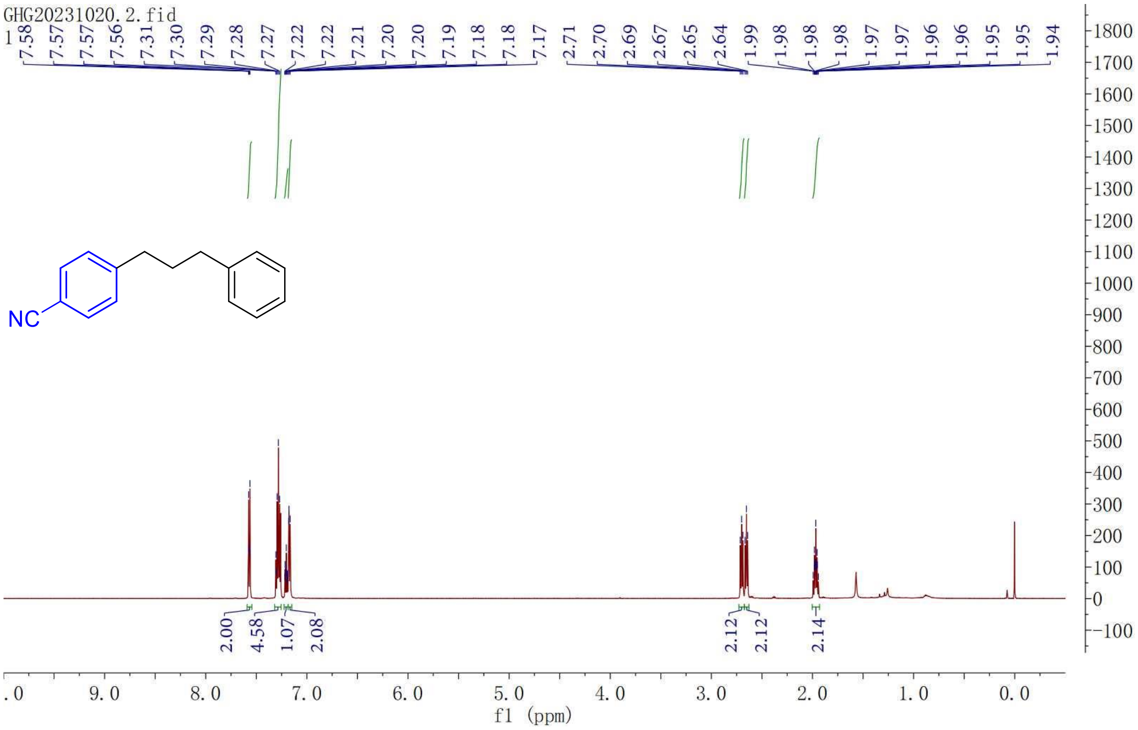

3, ^{13}C NMR (151 MHz, CDCl$_3$)

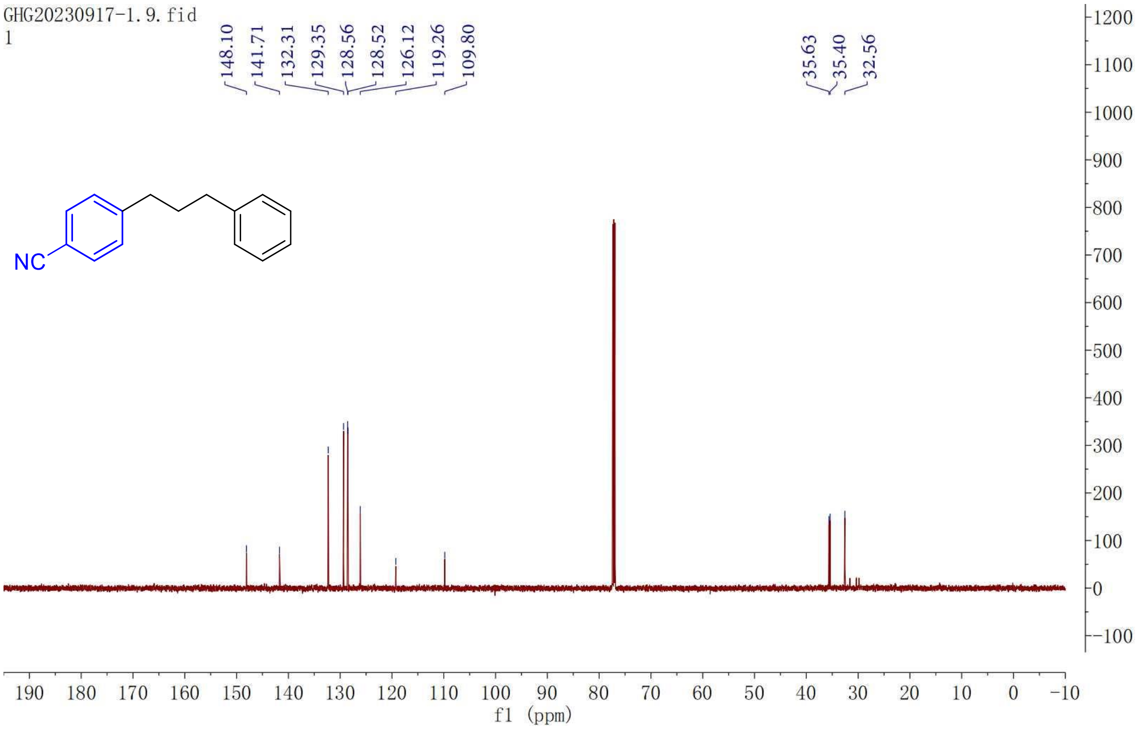

4, ^{1}H-NMR (600 MHZ, CDCl$_3$)

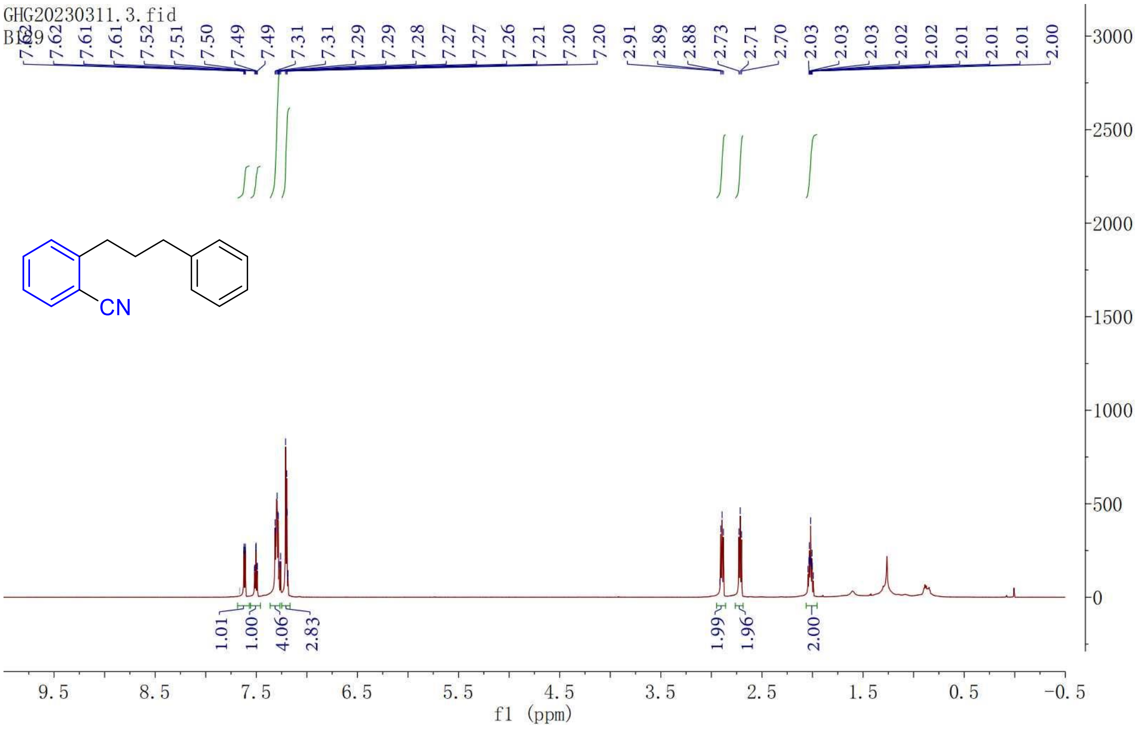

4, ^{13}C NMR (151 MHz, $CDCl_3$)

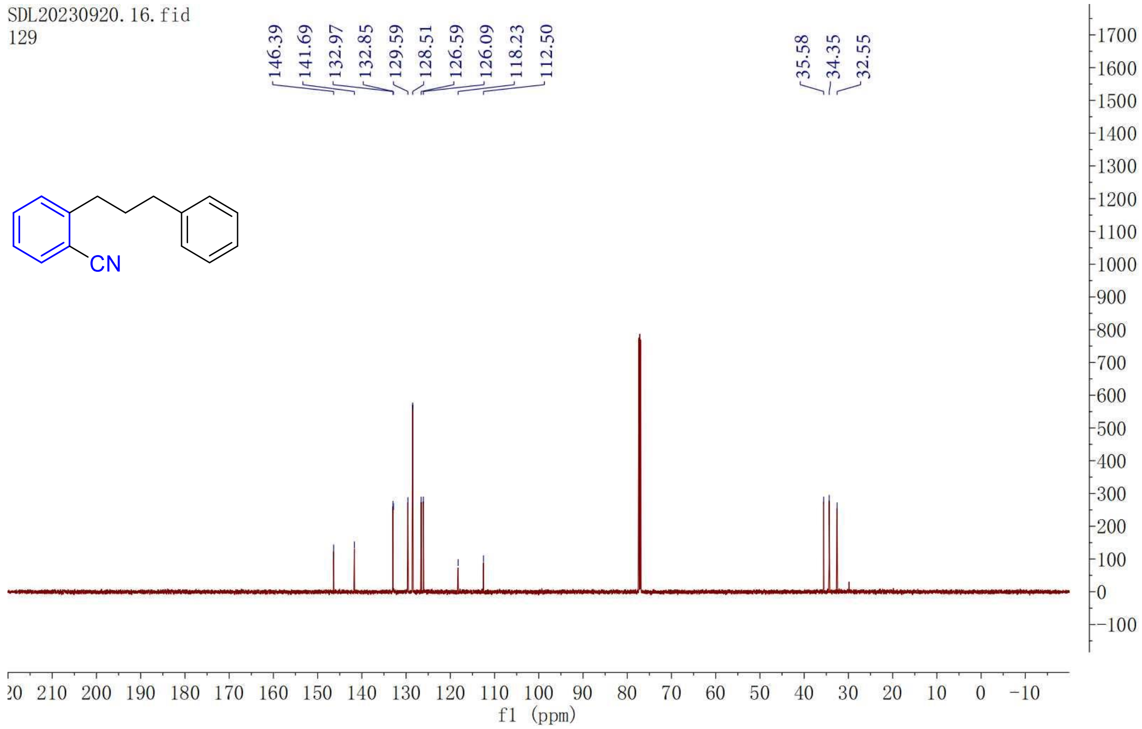

5, ^{1}H-NMR (600 MHZ, $CDCl_3$)

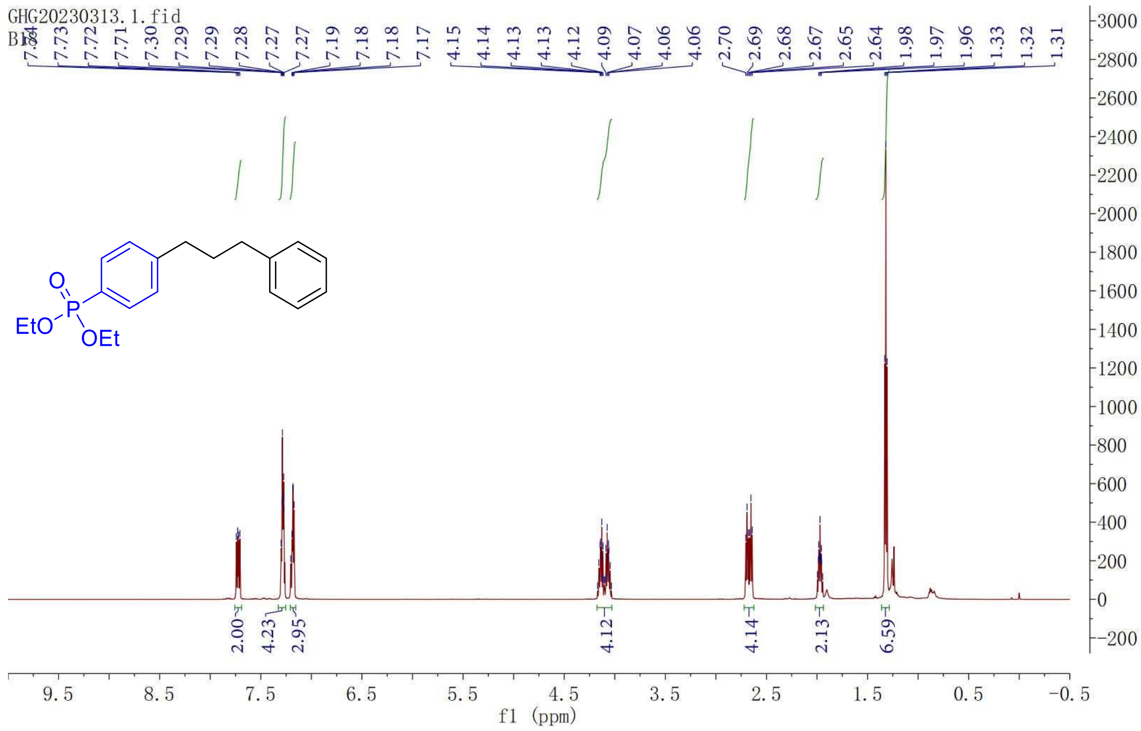

5, ^{13}C NMR (151 MHz, CDCl$_3$)

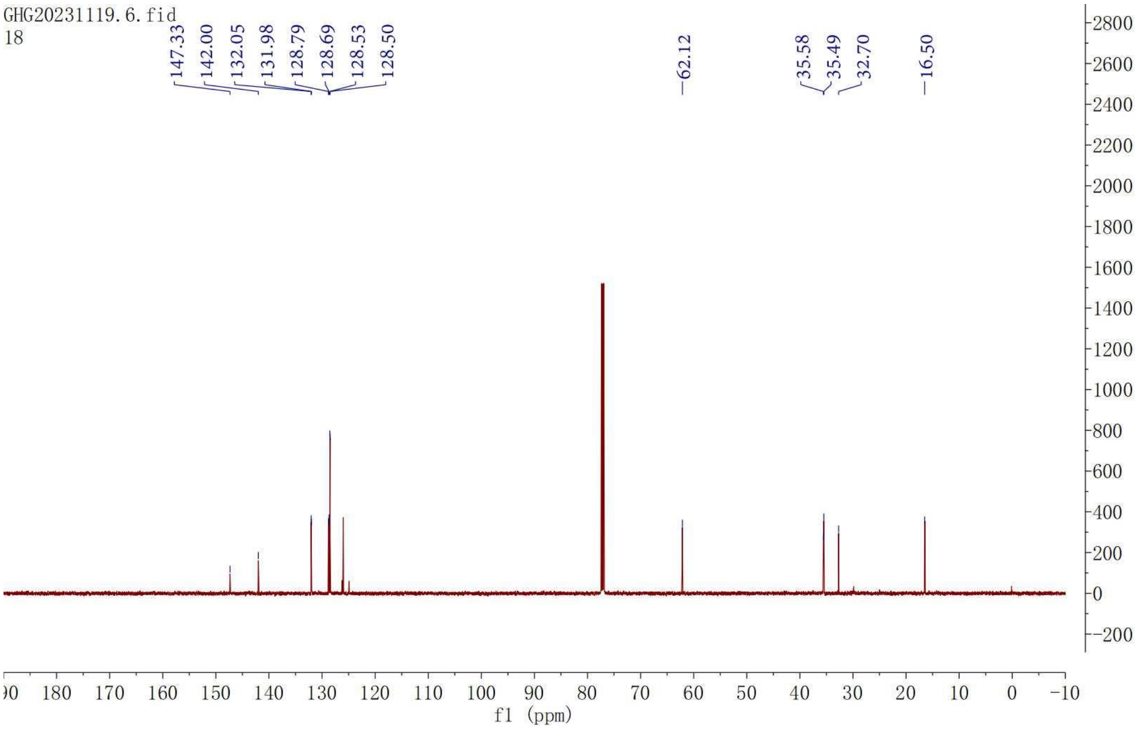

6, ^{1}H-NMR (600 MHZ, CDCl$_3$)

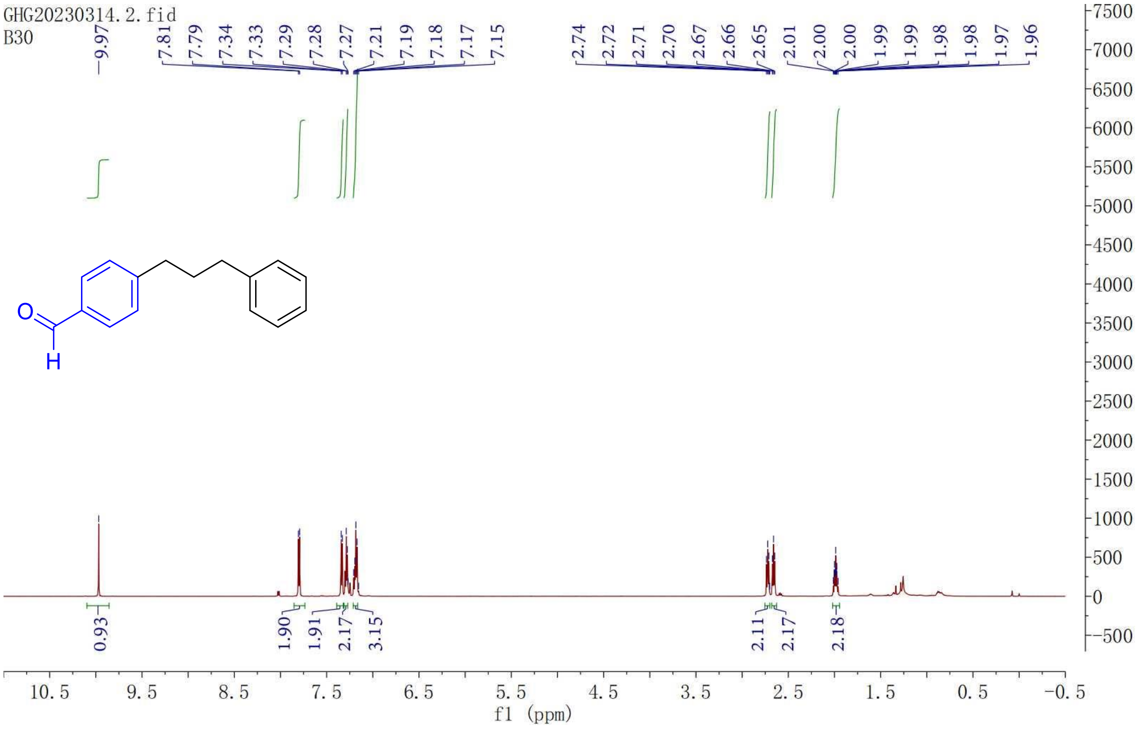

7, ^{1}H-NMR (600 MHZ, CDCl₃)

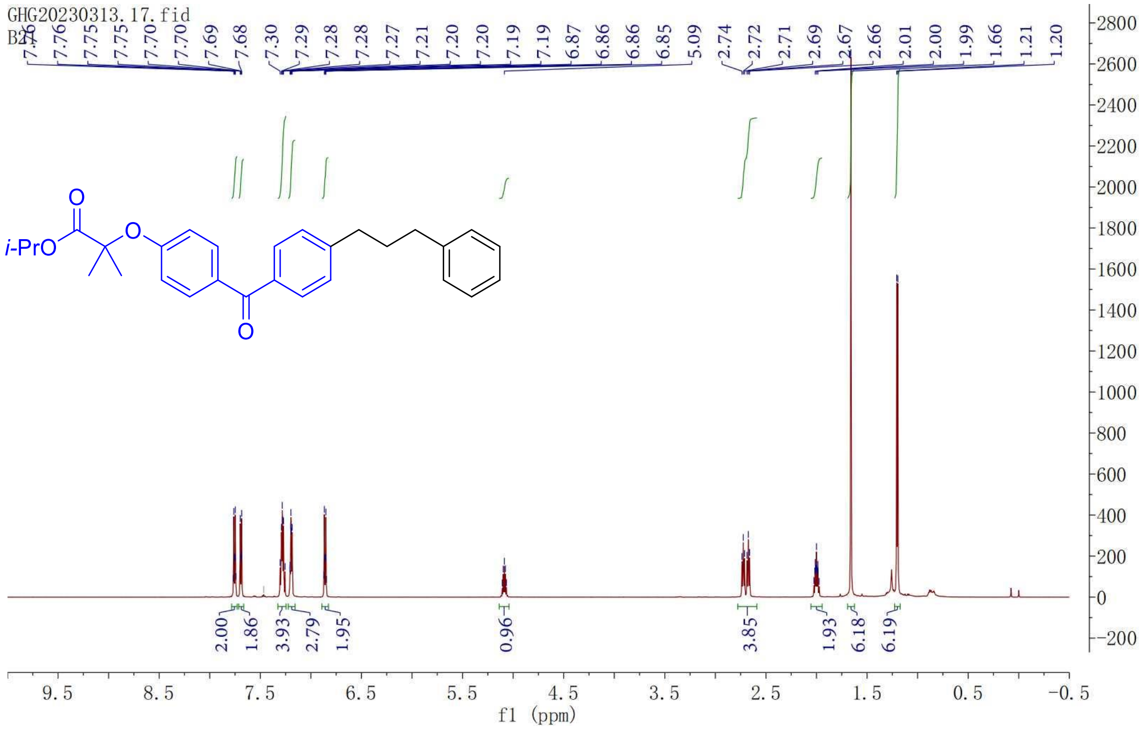

7, ^{13}C NMR (151 MHz, CDCl₃)

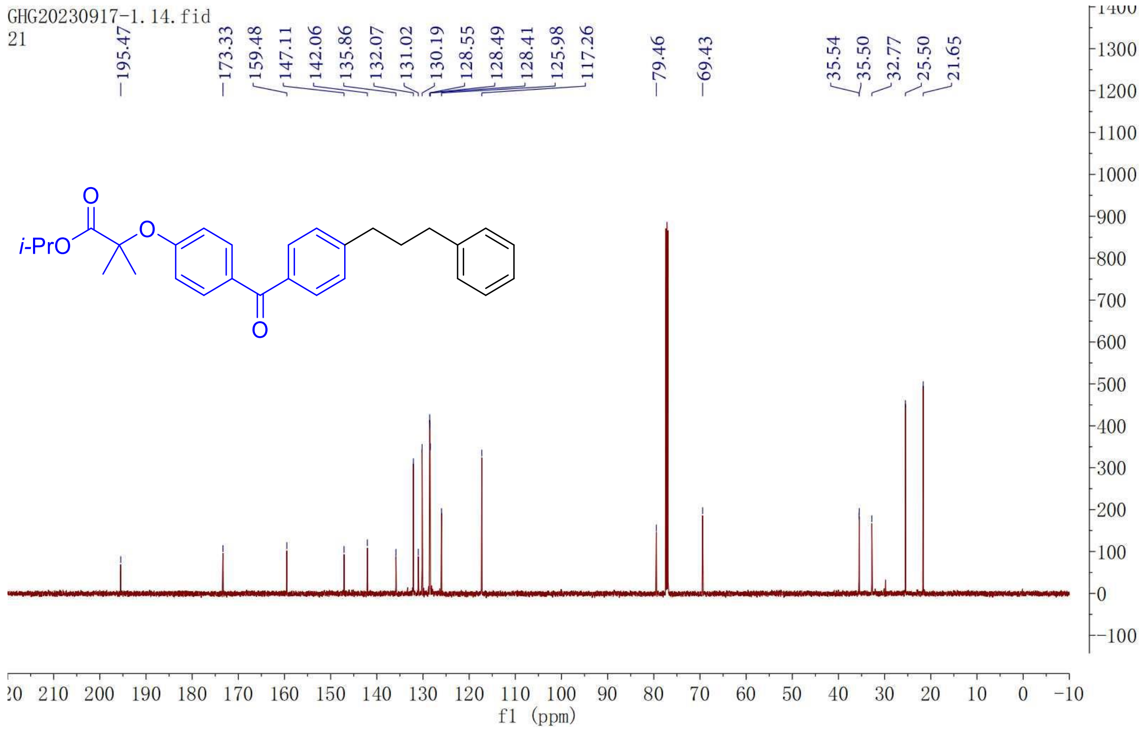

8, ^{1}H-NMR (600 MHZ, $CDCl_3$)

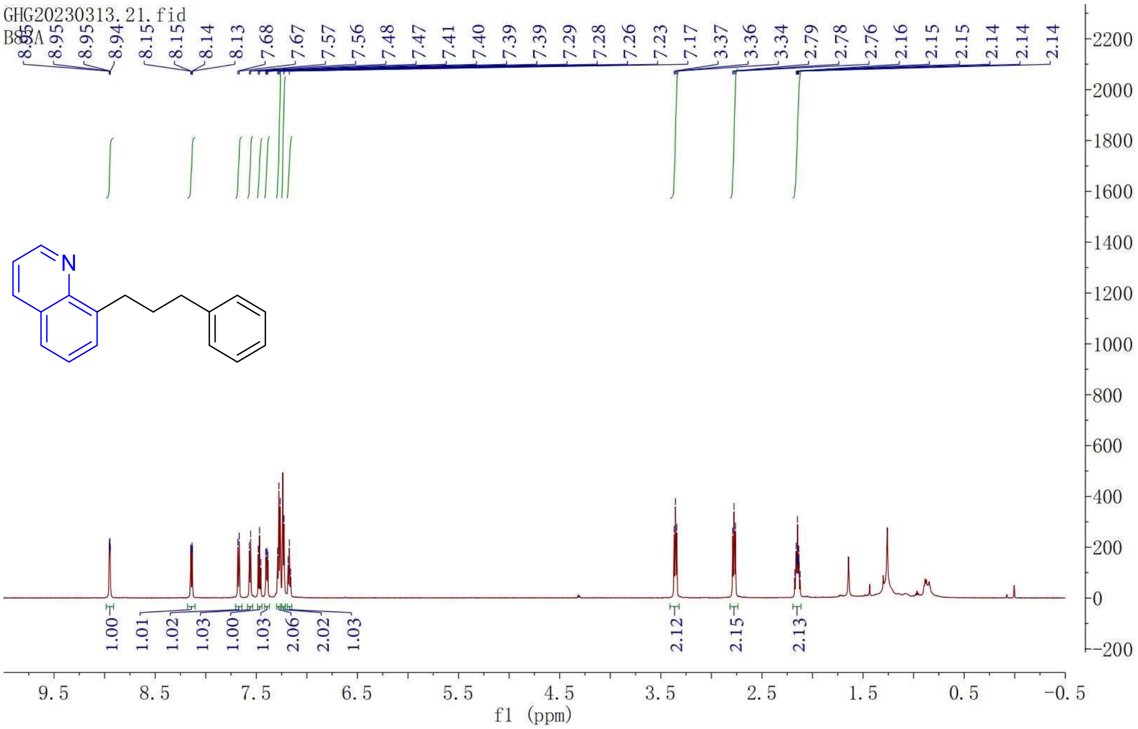

9, ^{1}H-NMR (600 MHZ, $CDCl_3$)

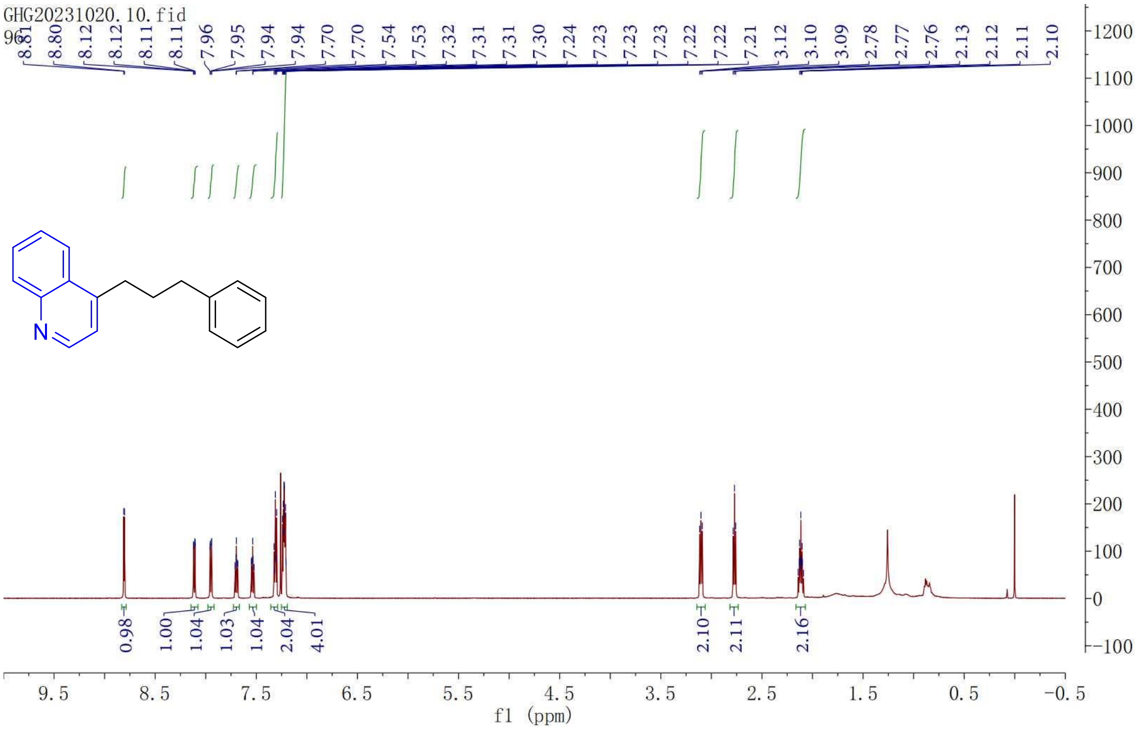

9, ^{13}C NMR (151 MHz, $CDCl_3$)

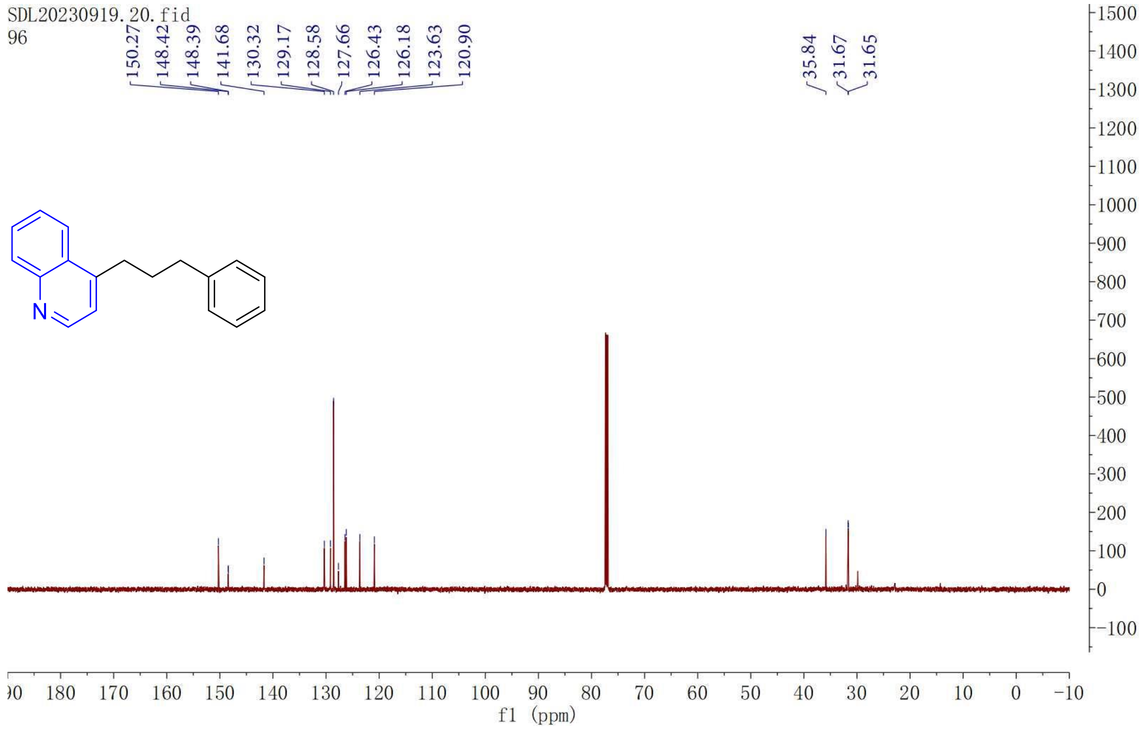

10, ^{1}H-NMR (600 MHZ, $CDCl_3$)

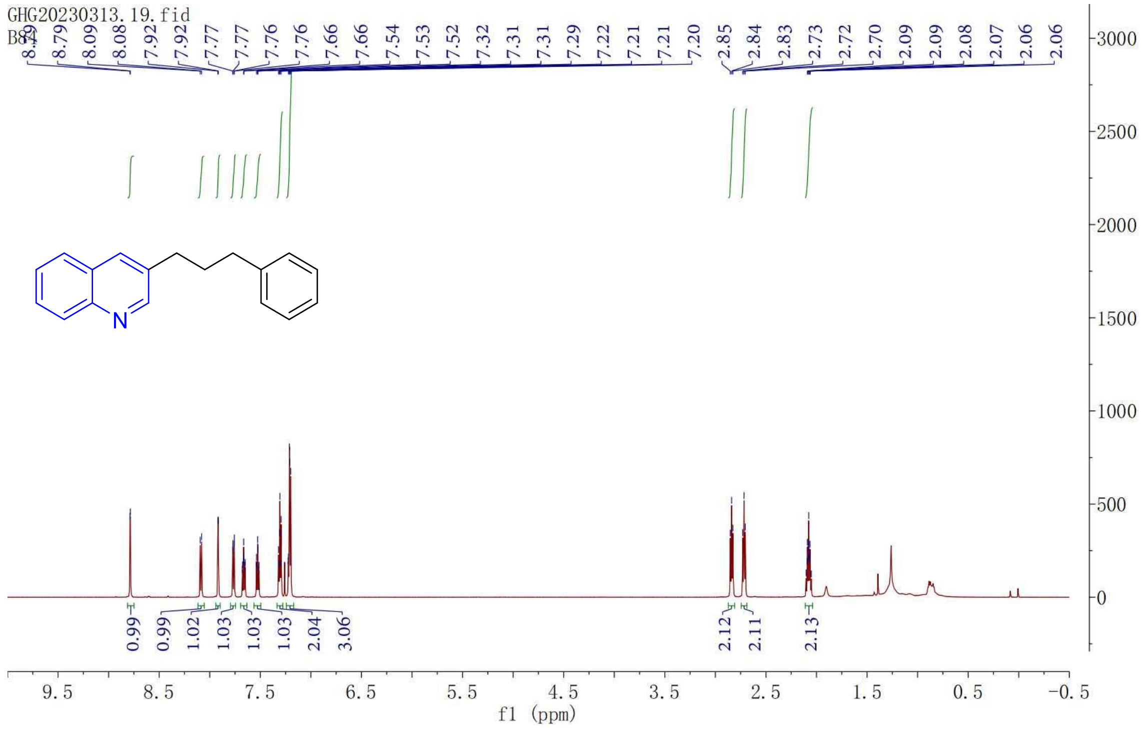

10, ^{13}C NMR (151 MHz, CDCl$_3$)

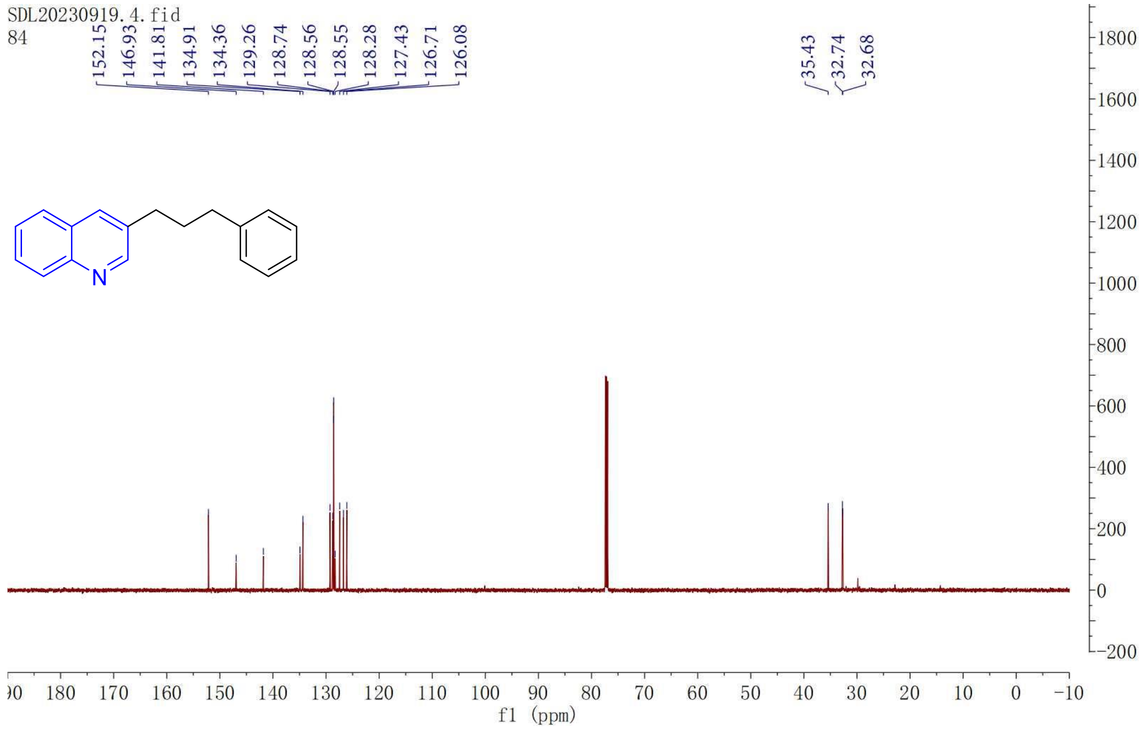

11, ^{1}H-NMR (600 MHZ, CDCl$_3$)

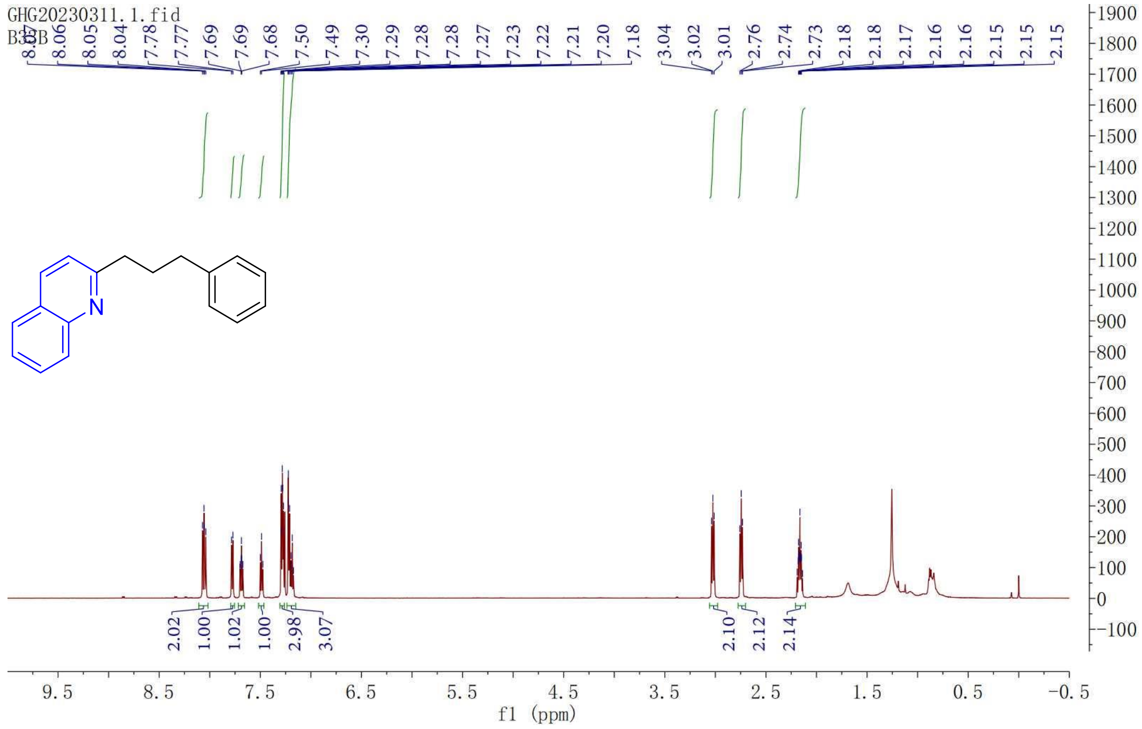

12, ^{1}H-NMR (600 MHZ, CDCl$_3$)

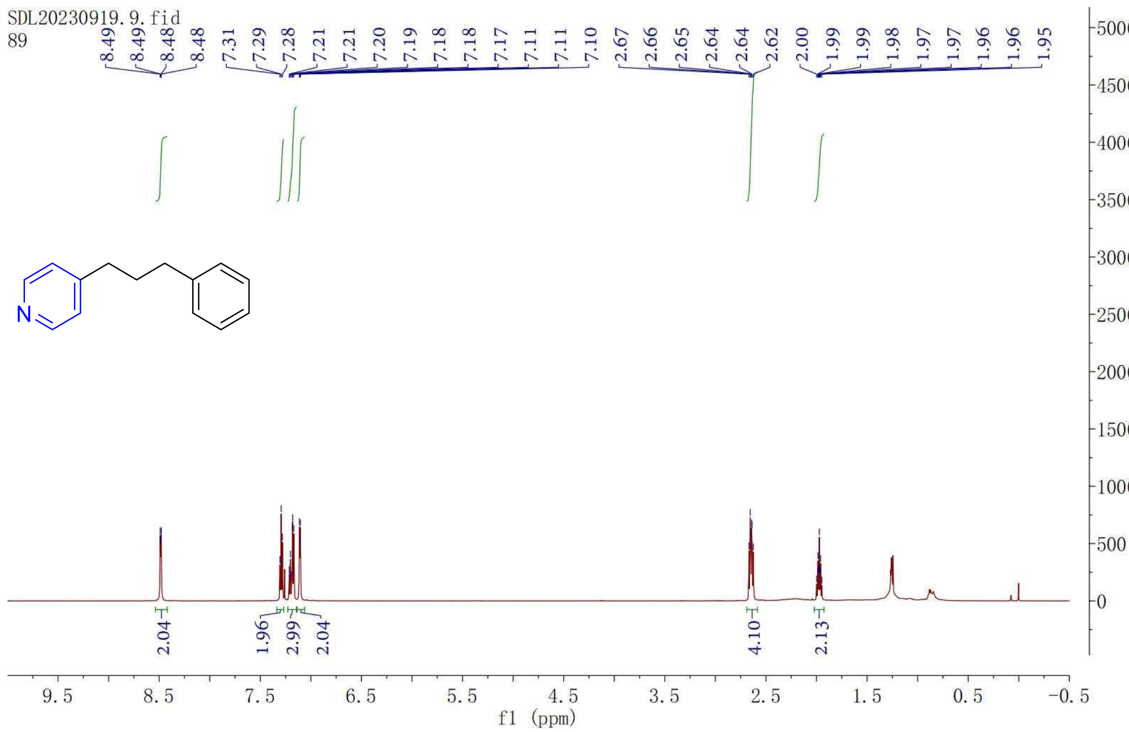

12, ^{13}C NMR (151 MHz, CDCl$_3$)

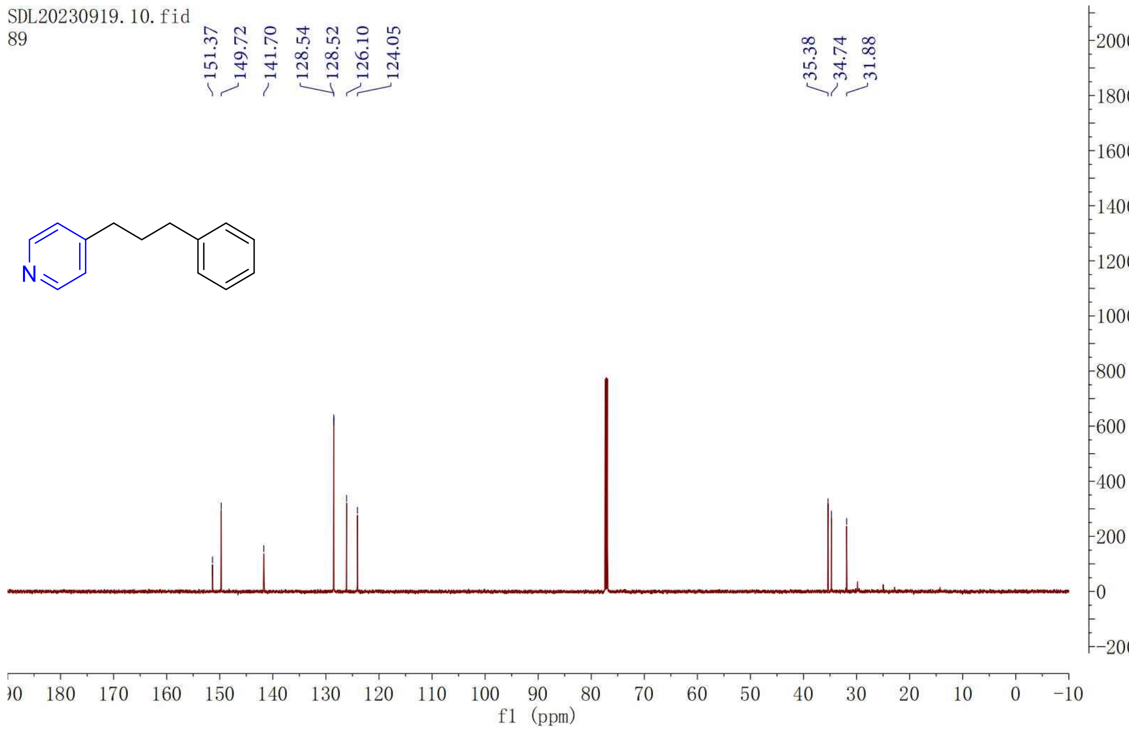

13, ¹H-NMR (600 MHZ, CDCl₃)

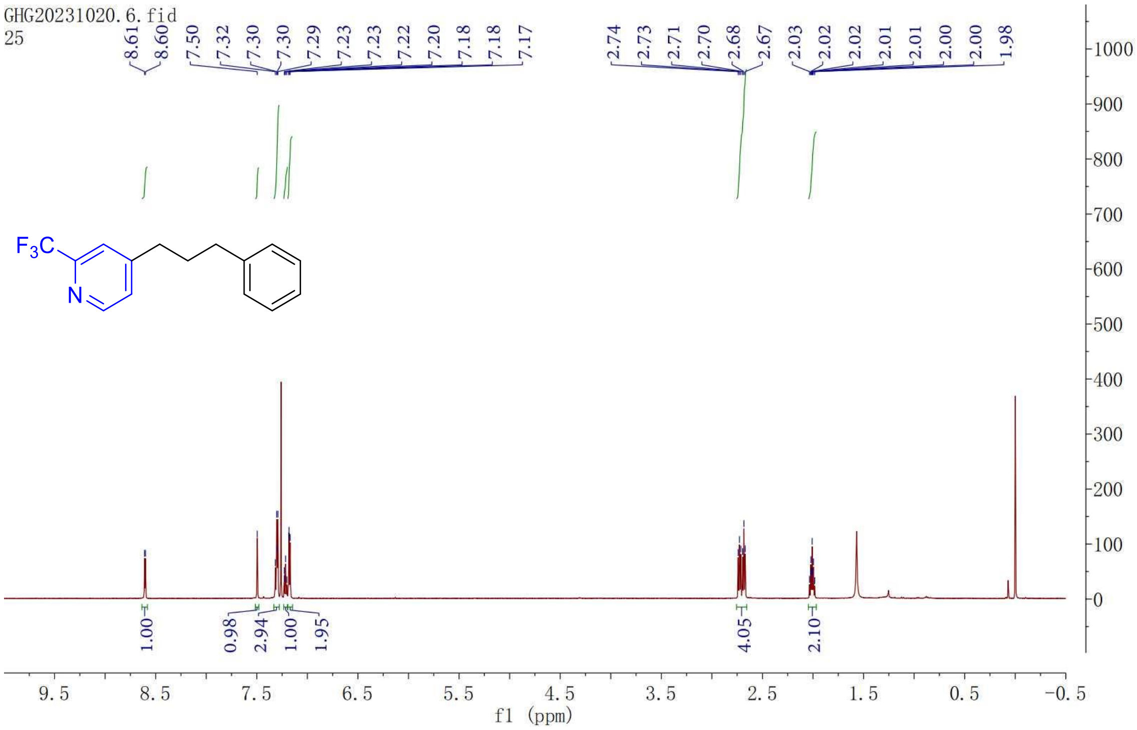

13, ¹³C NMR (151 MHz, CDCl₃)

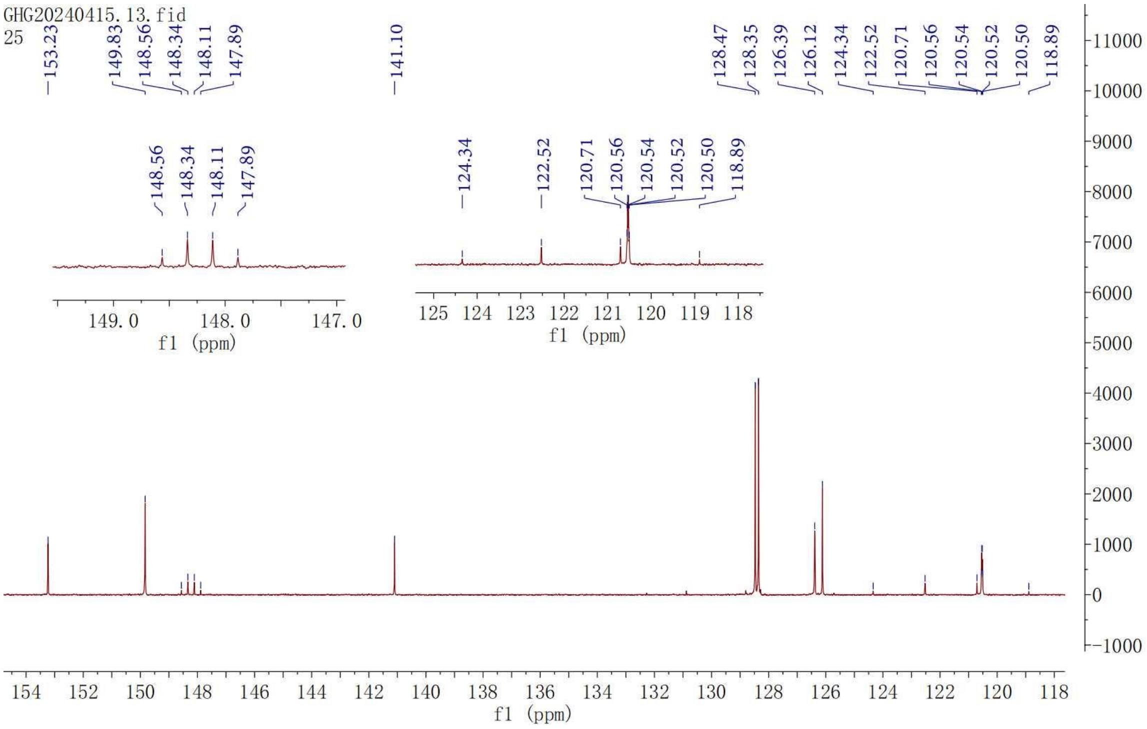

14, ^{1}H-NMR (600 MHZ, $CDCl_3$)

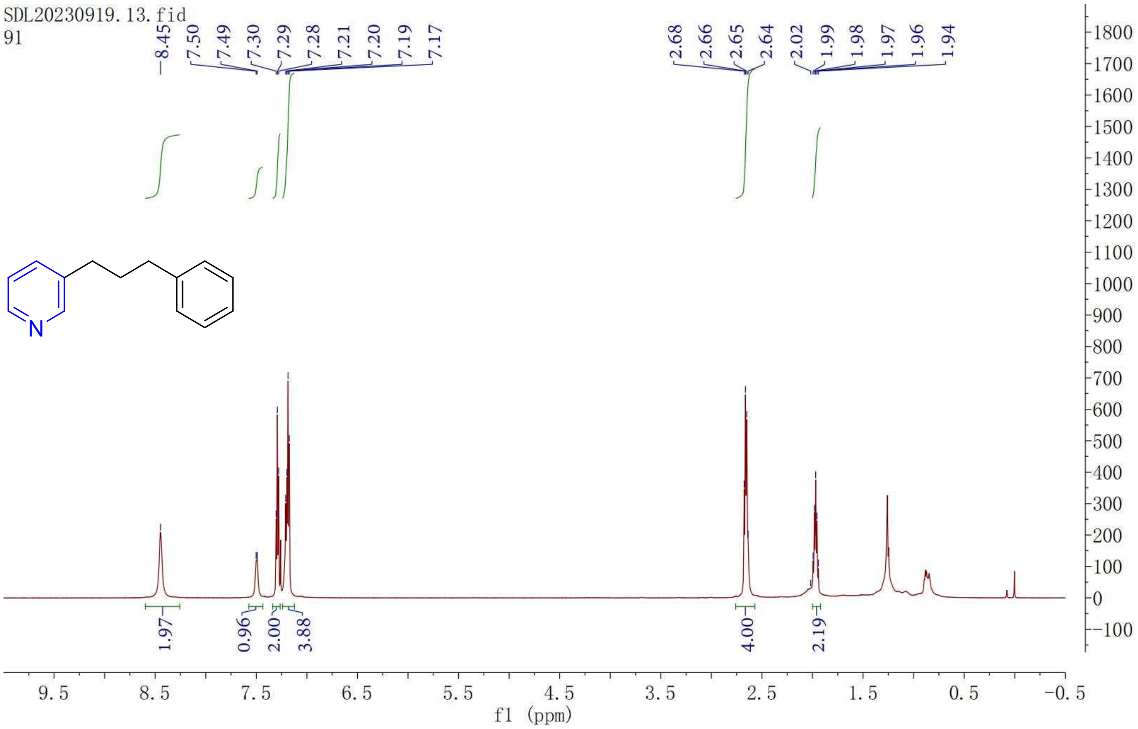

14, ^{13}C NMR (151 MHz, $CDCl_3$)

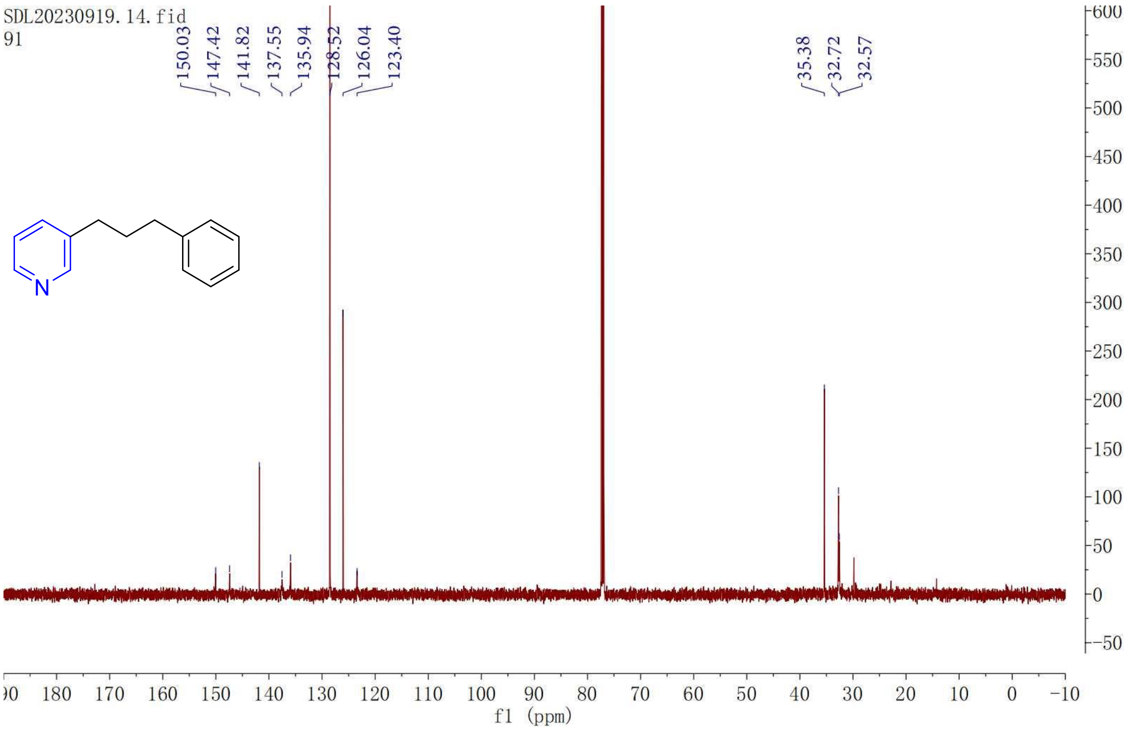

15, ^{1}H-NMR (600 MHZ, CDCl$_3$)

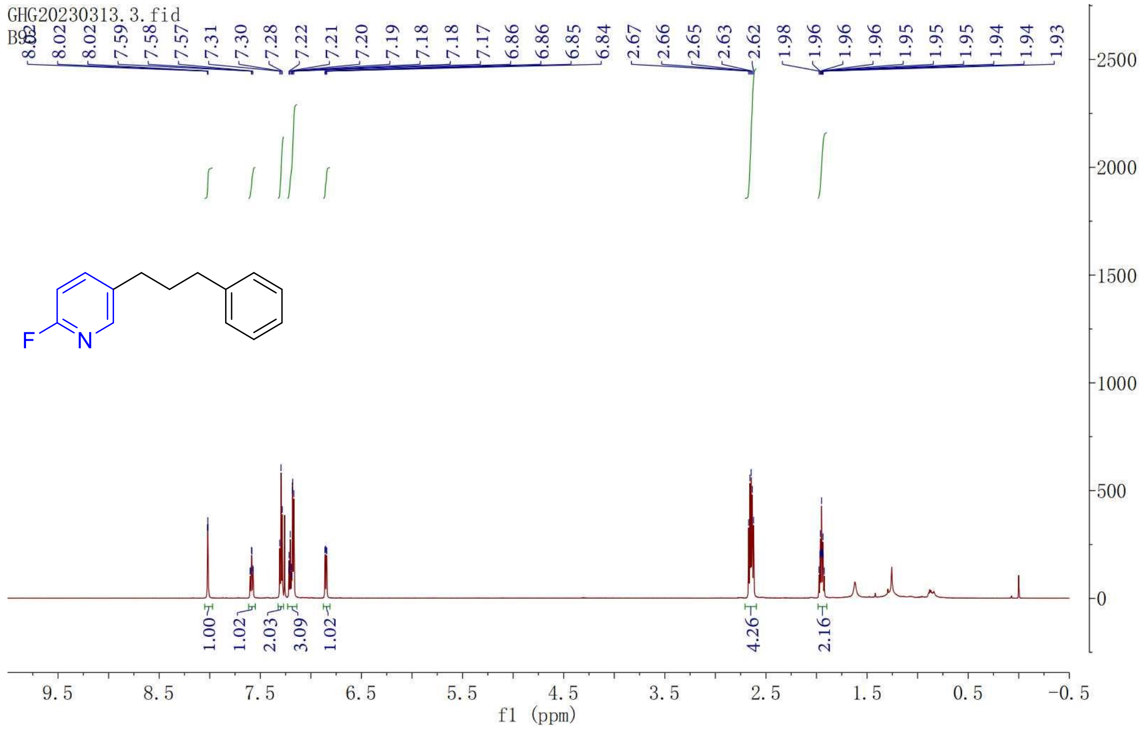

16, ^{1}H-NMR (600 MHZ, CDCl$_3$)

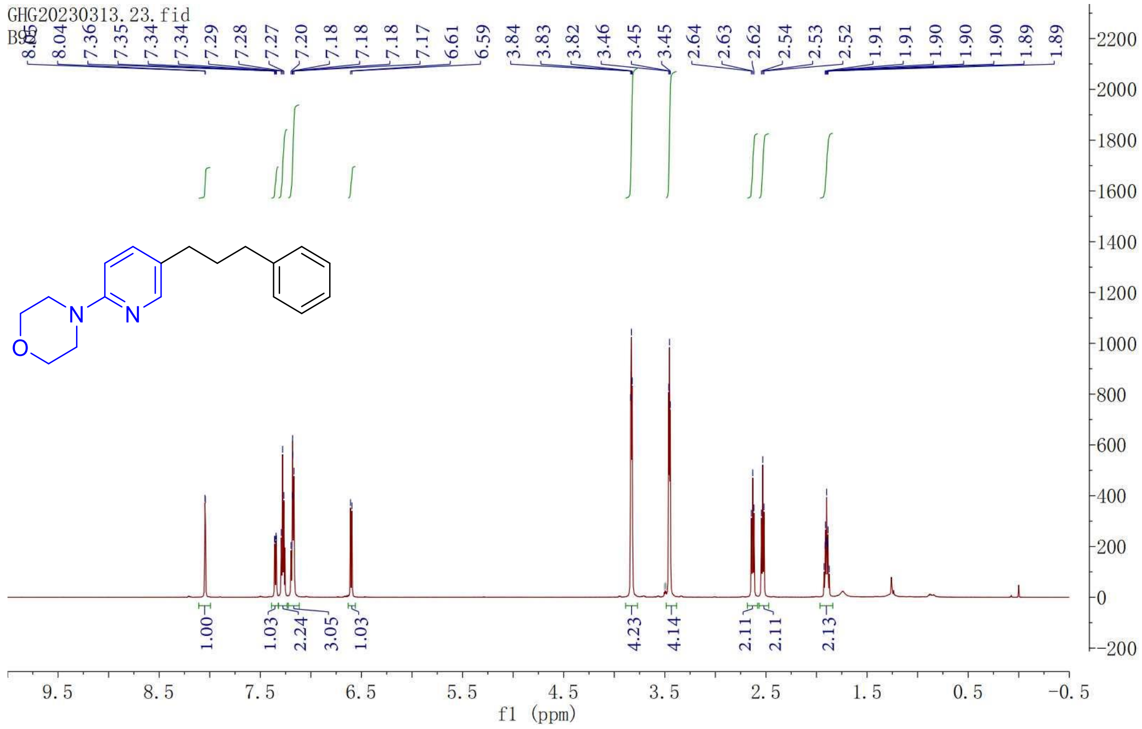

16, ^{13}C NMR (151 MHz, CDCl$_3$)

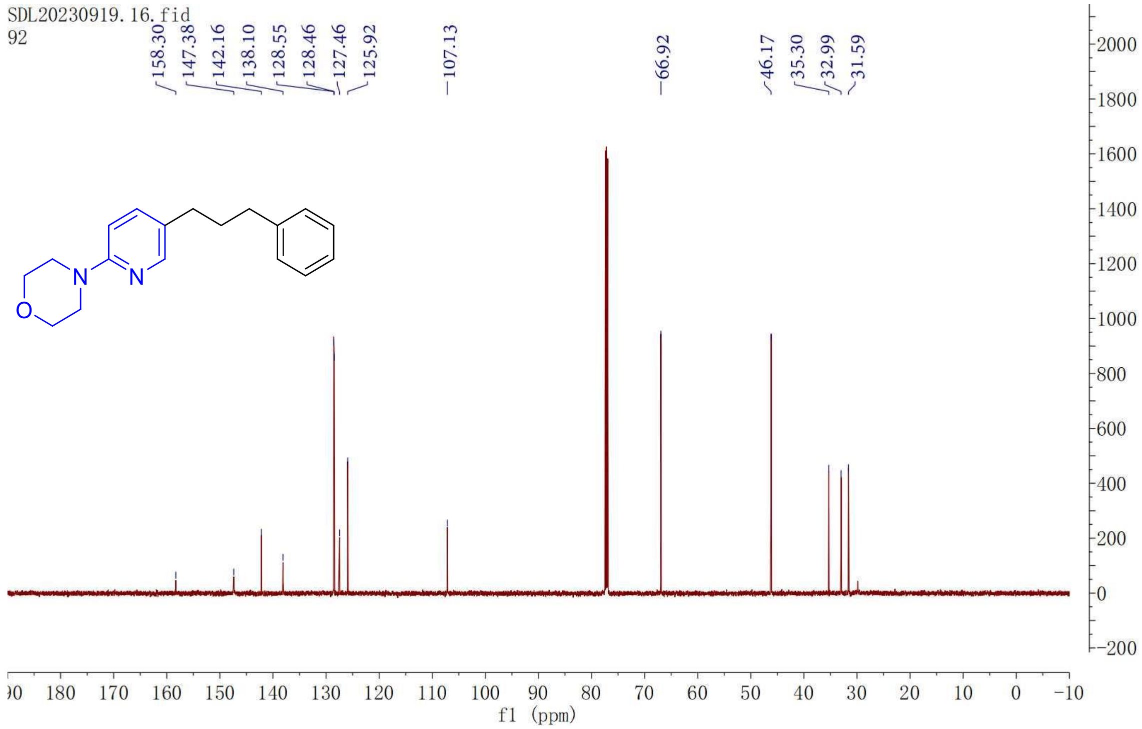

17, ^{1}H-NMR (600 MHZ, CDCl$_3$)

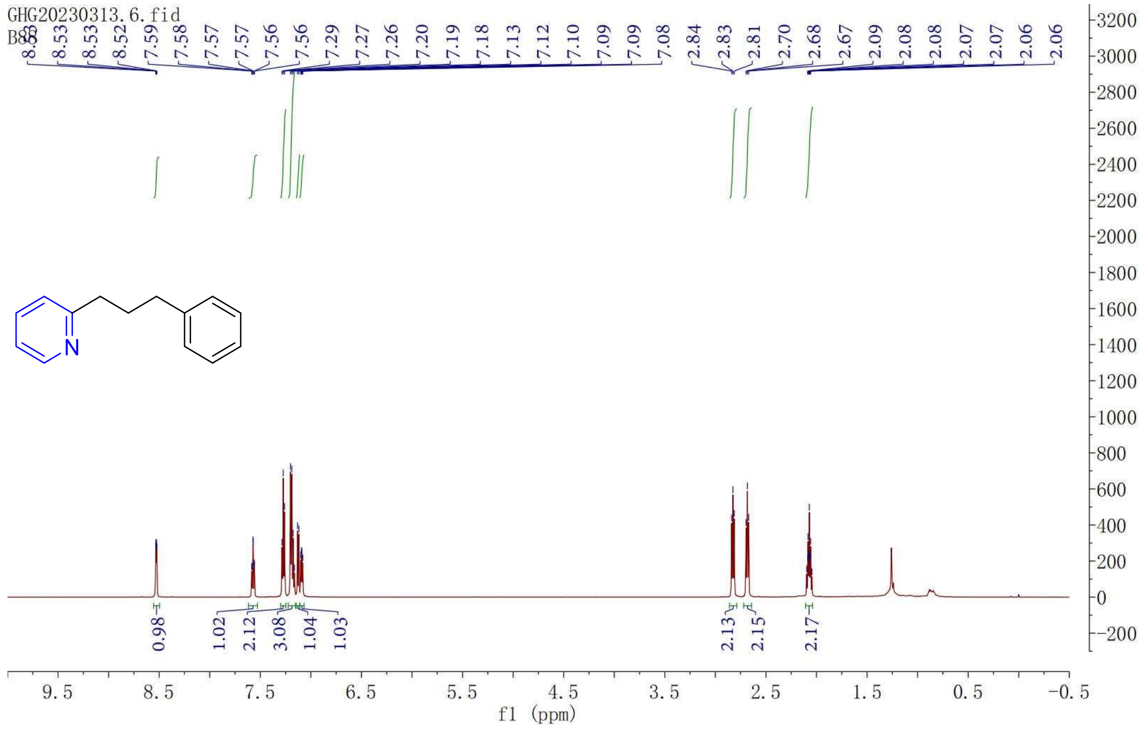

17, ^{13}C NMR (151 MHz, $CDCl_3$)

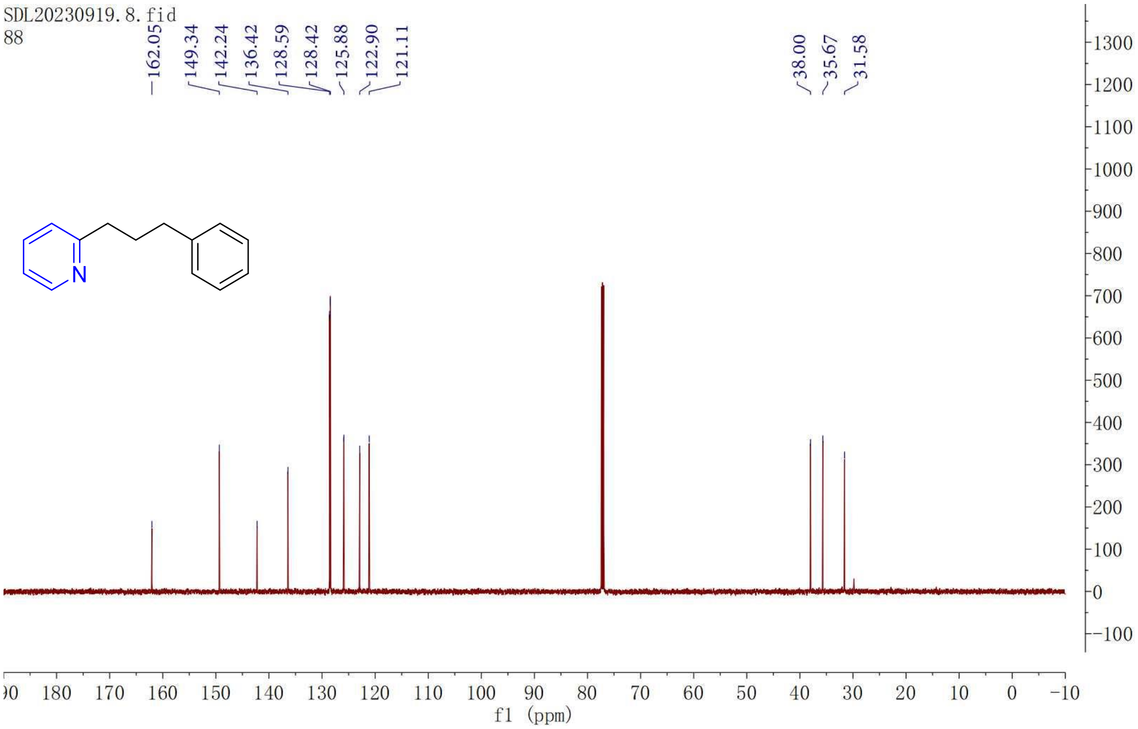

18, ^{1}H-NMR (600 MHZ, $CDCl_3$)

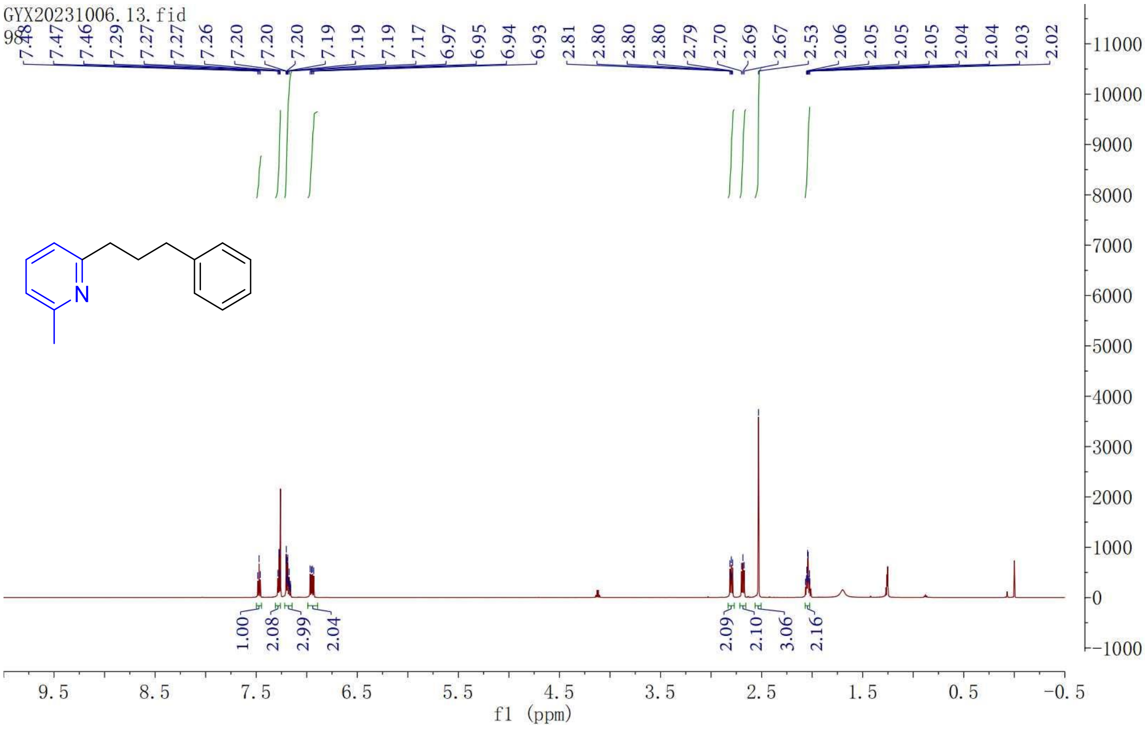

18, ^{13}C NMR (151 MHz, CDCl$_3$)

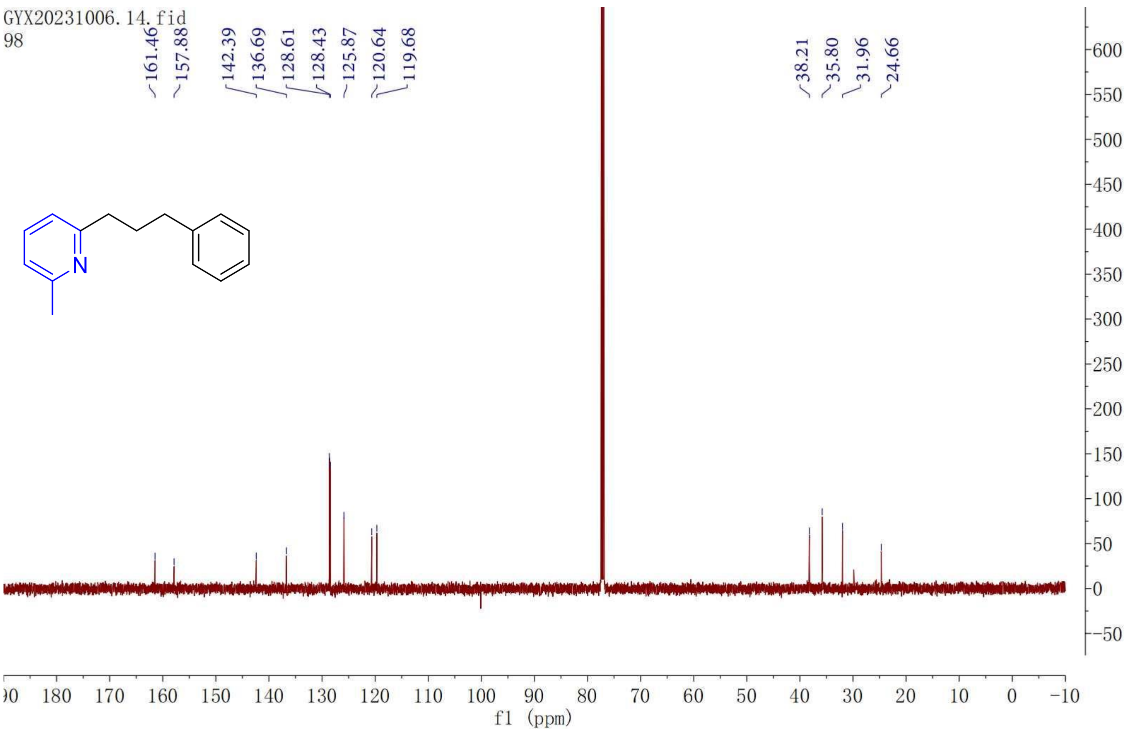

19, ^{1}H-NMR (600 MHZ, CDCl$_3$)

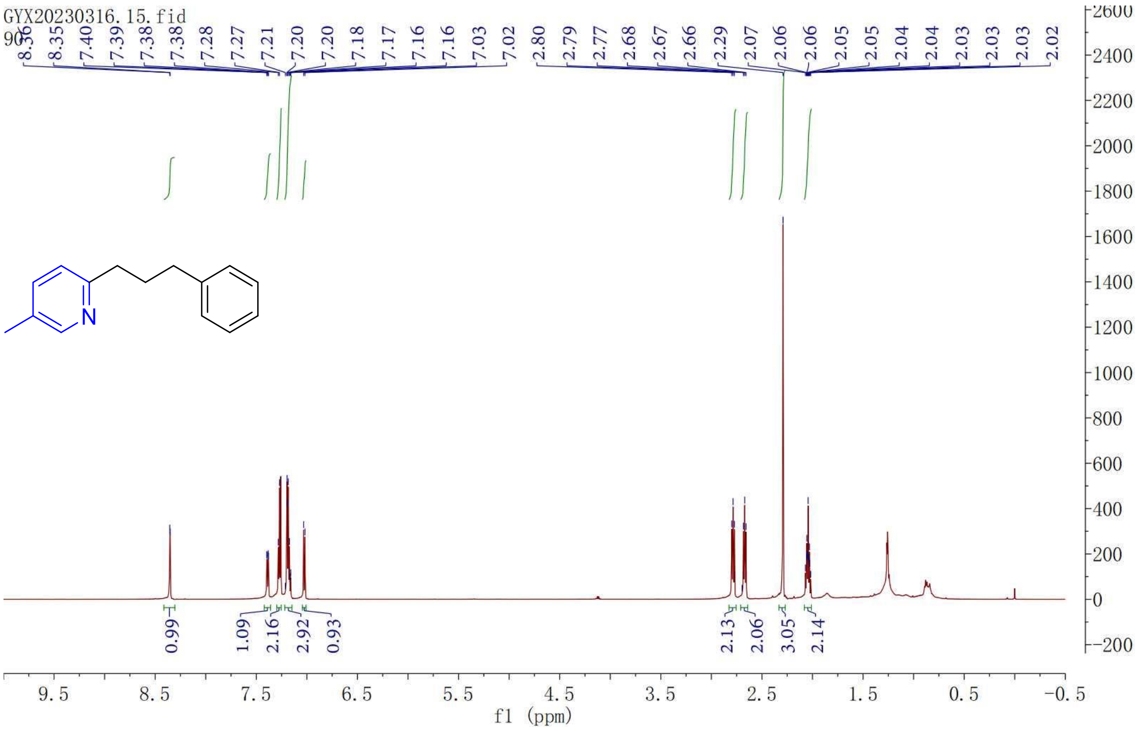

19, ^{13}C NMR (151 MHz, CDCl$_3$)

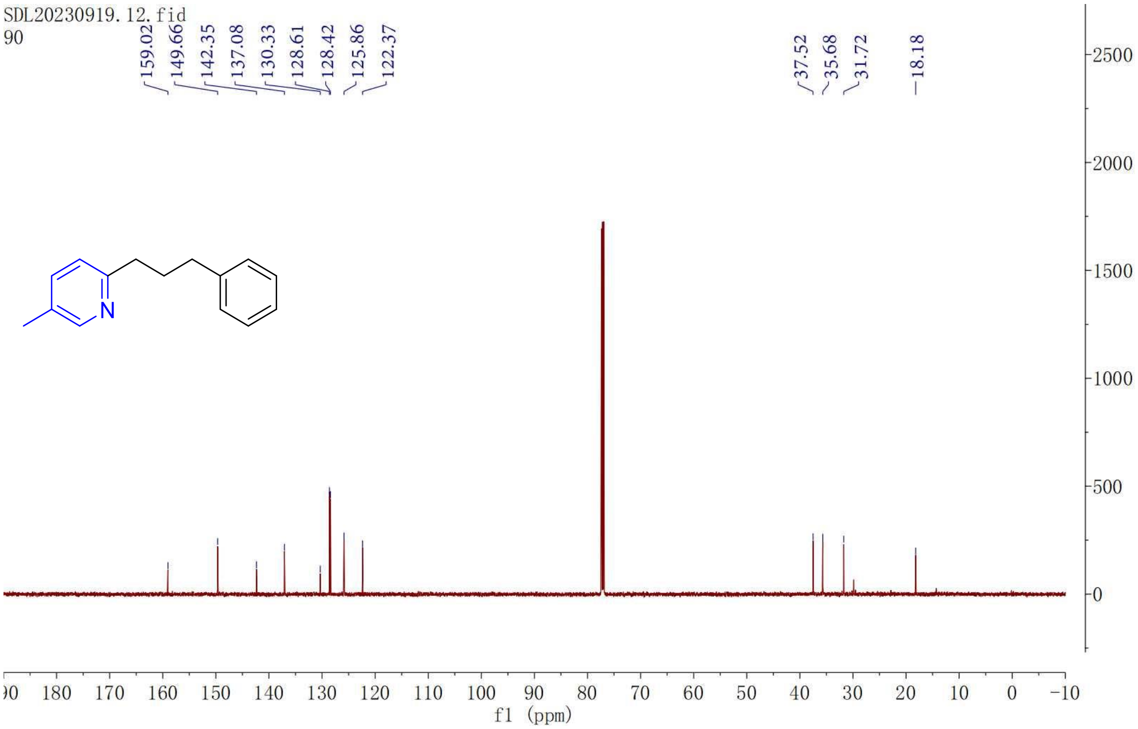

20, ^{1}H-NMR (600 MHZ, CDCl$_3$)

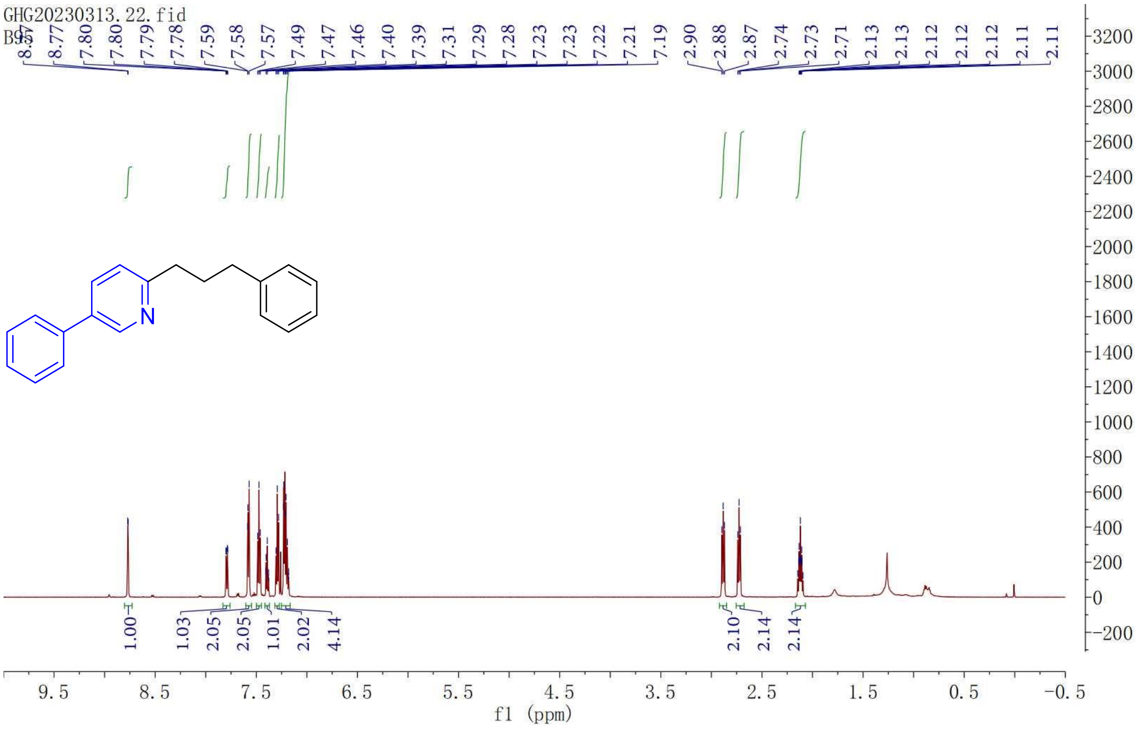

20, ^{13}C NMR (151 MHz, $CDCl_3$)

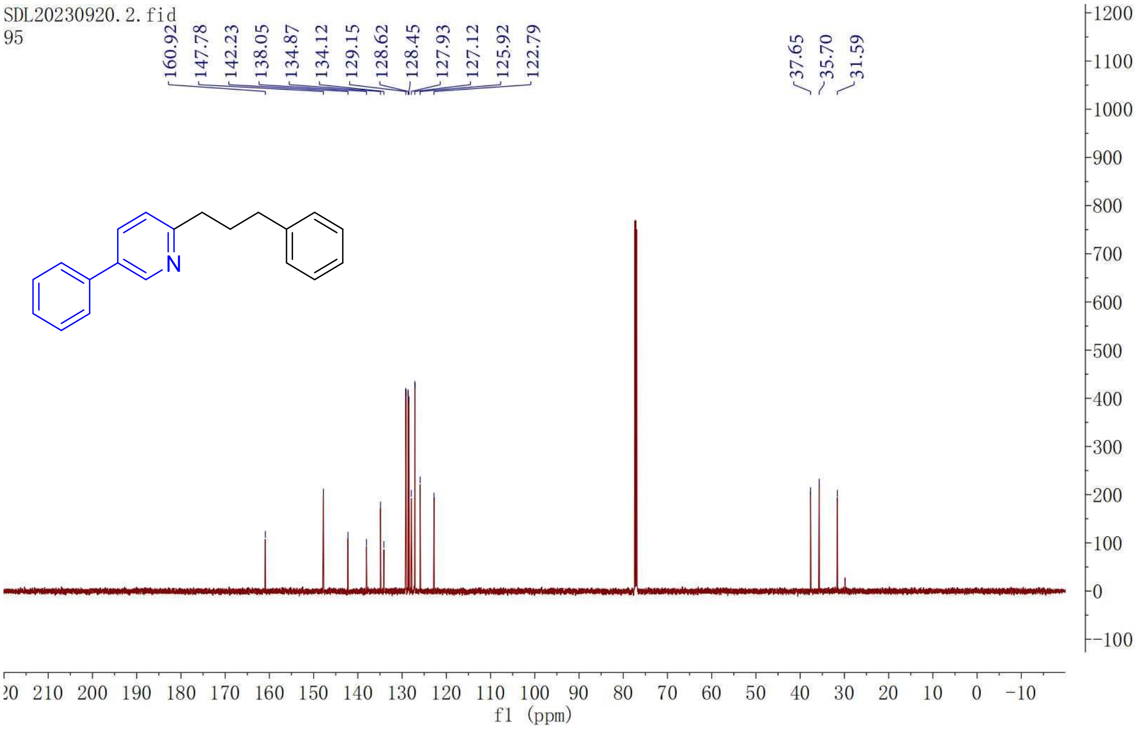

21, ^{1}H-NMR (600 MHZ, $CDCl_3$)

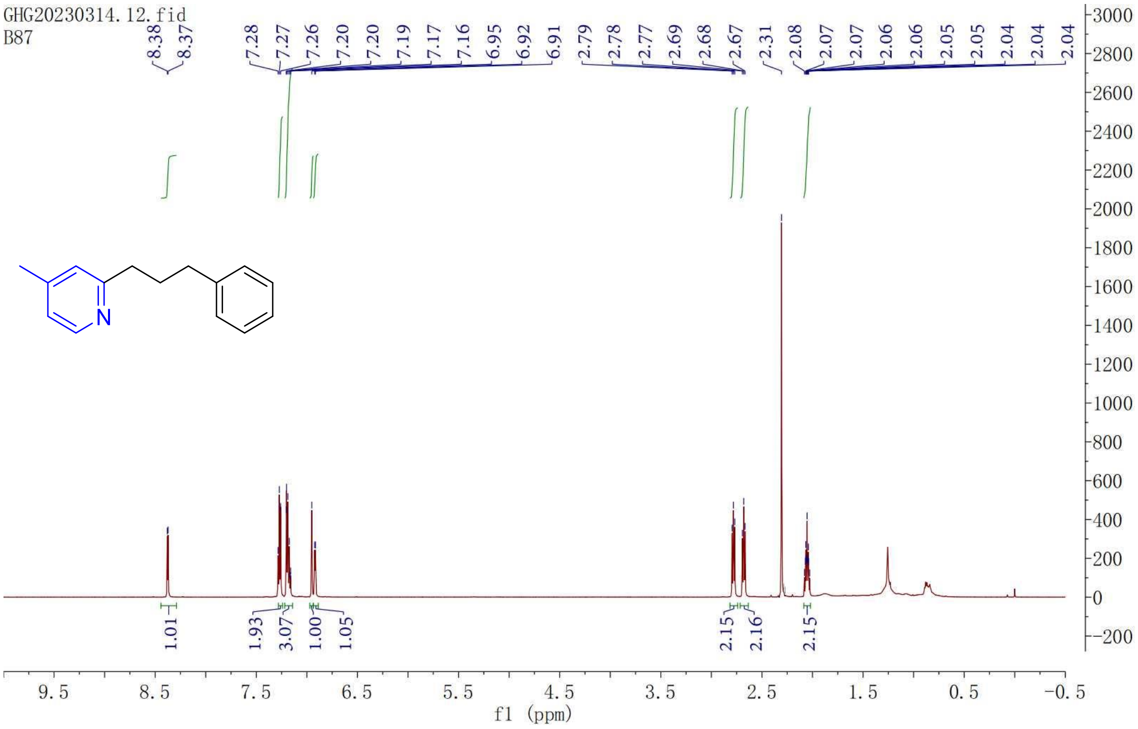

21, ^{13}C NMR (151 MHz, CDCl$_3$)

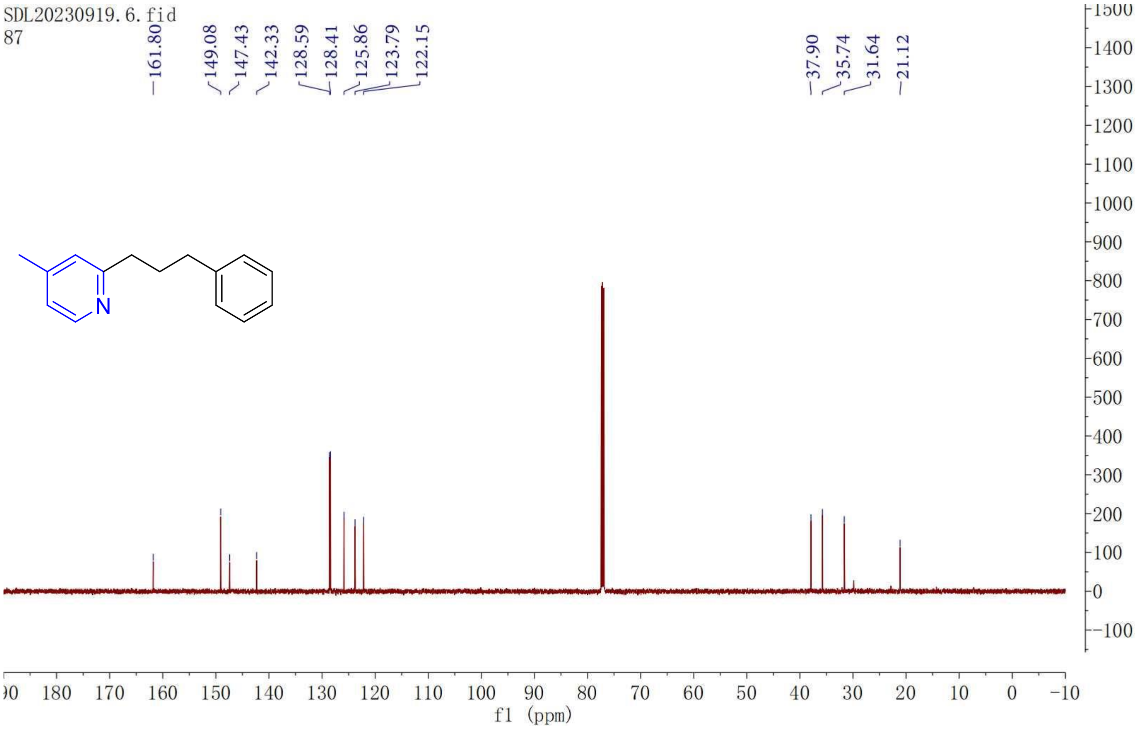

22, ^{1}H-NMR (600 MHZ, CDCl$_3$)

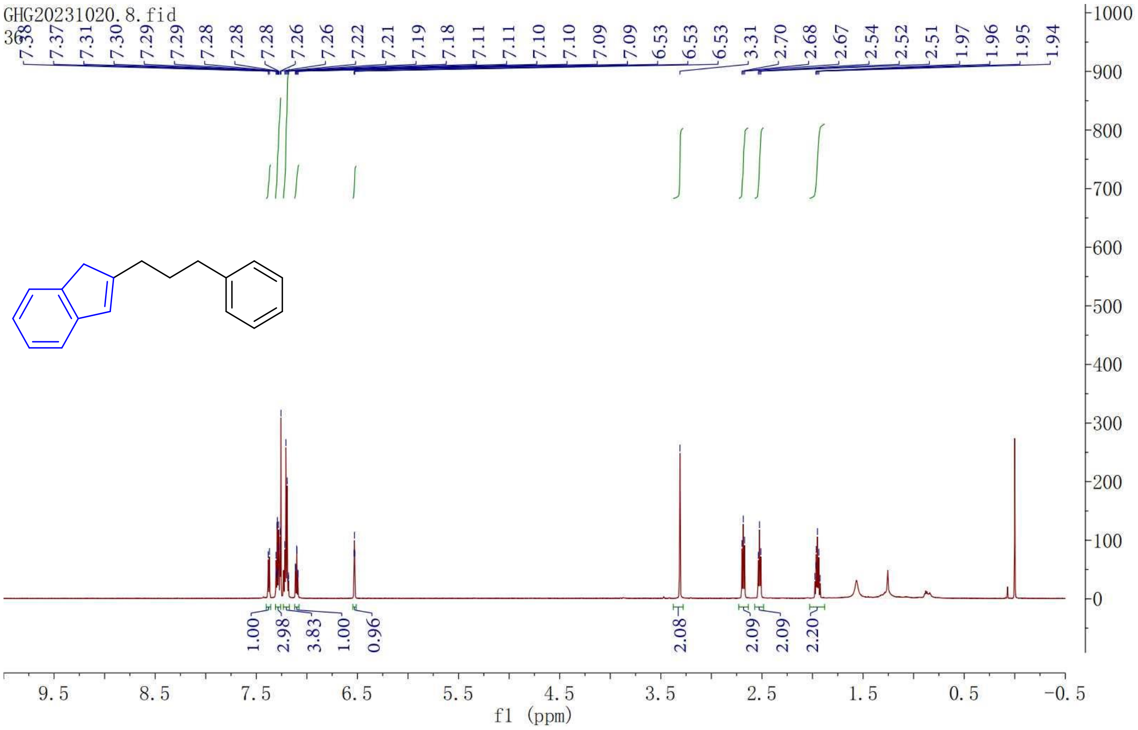

23, ^{1}H-NMR (600 MHZ, $CDCl_3$)

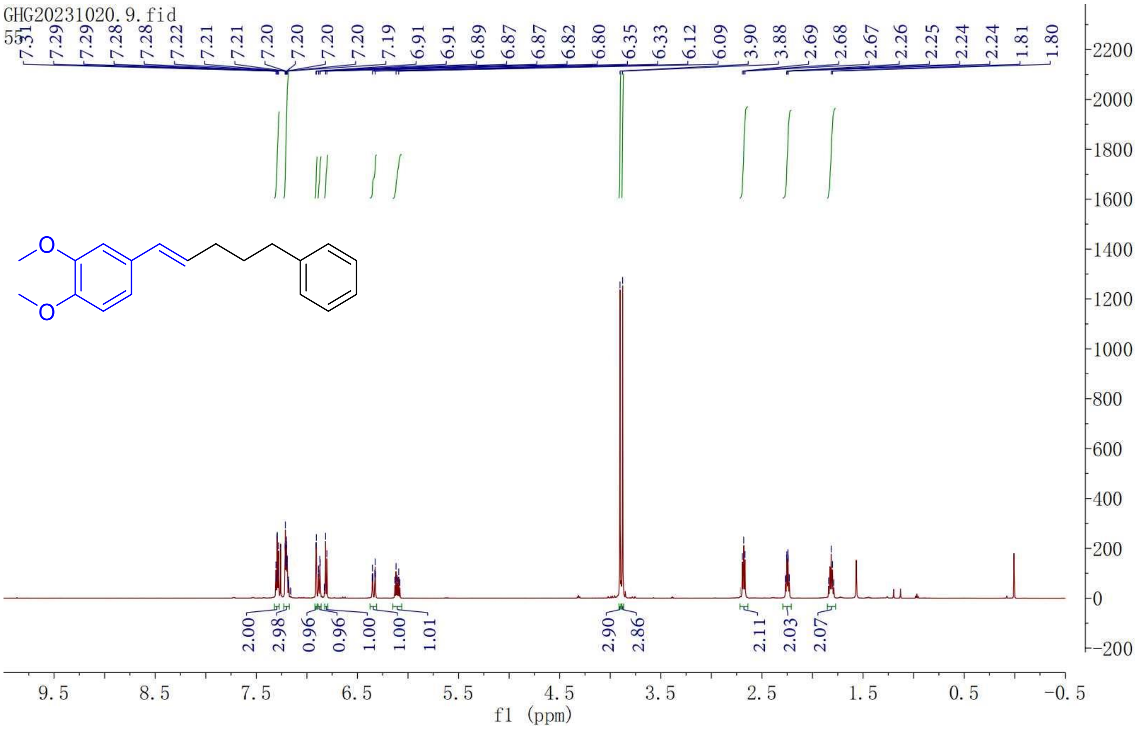

23, ^{13}C NMR (151 MHz, $CDCl_3$)

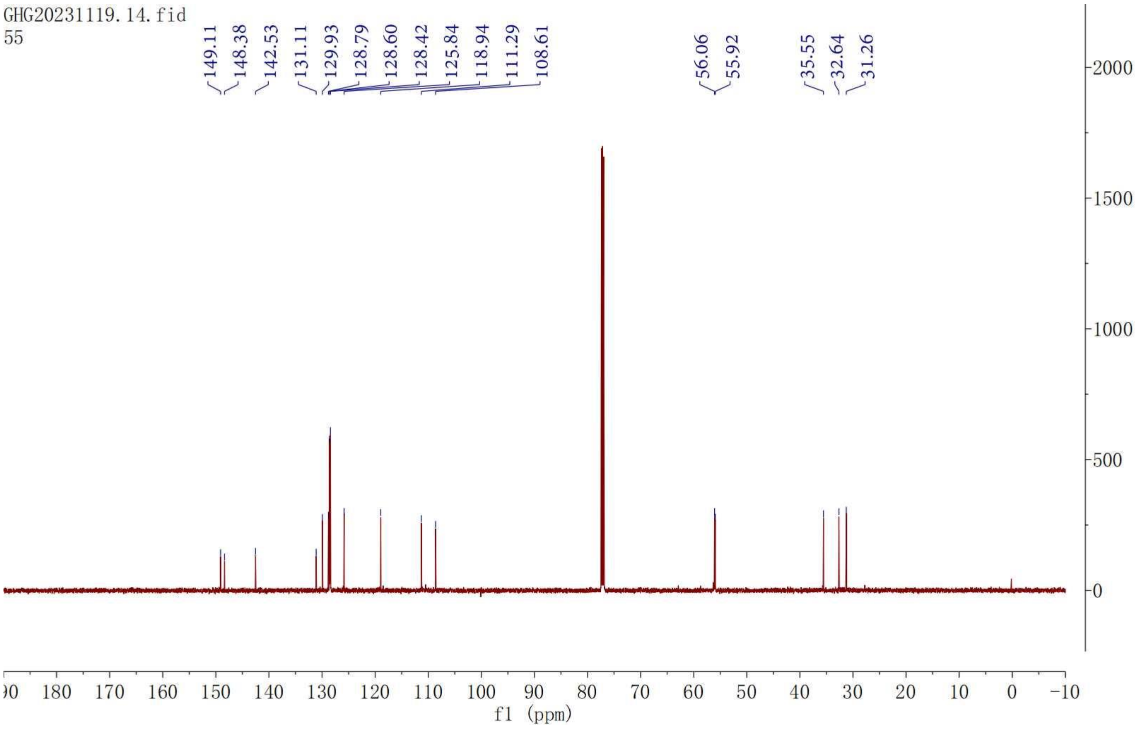

24, ^{1}H-NMR (600 MHZ, CDCl3)

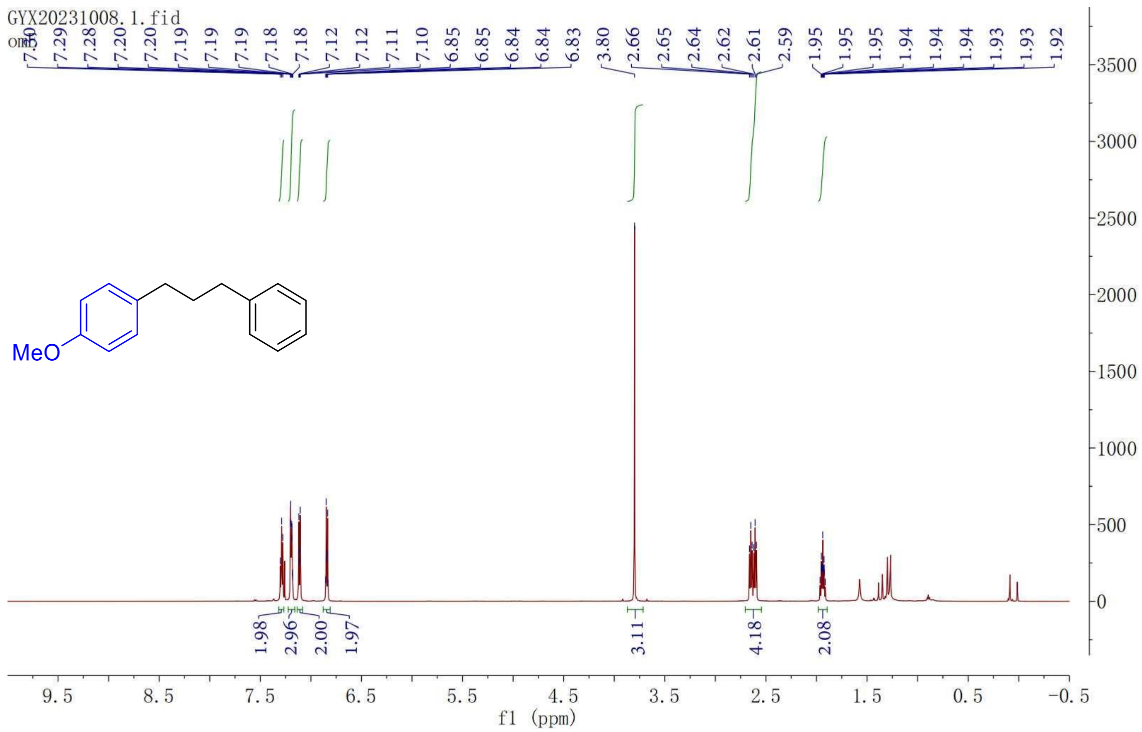

25, ^{1}H-NMR (600 MHZ, CDCl3)

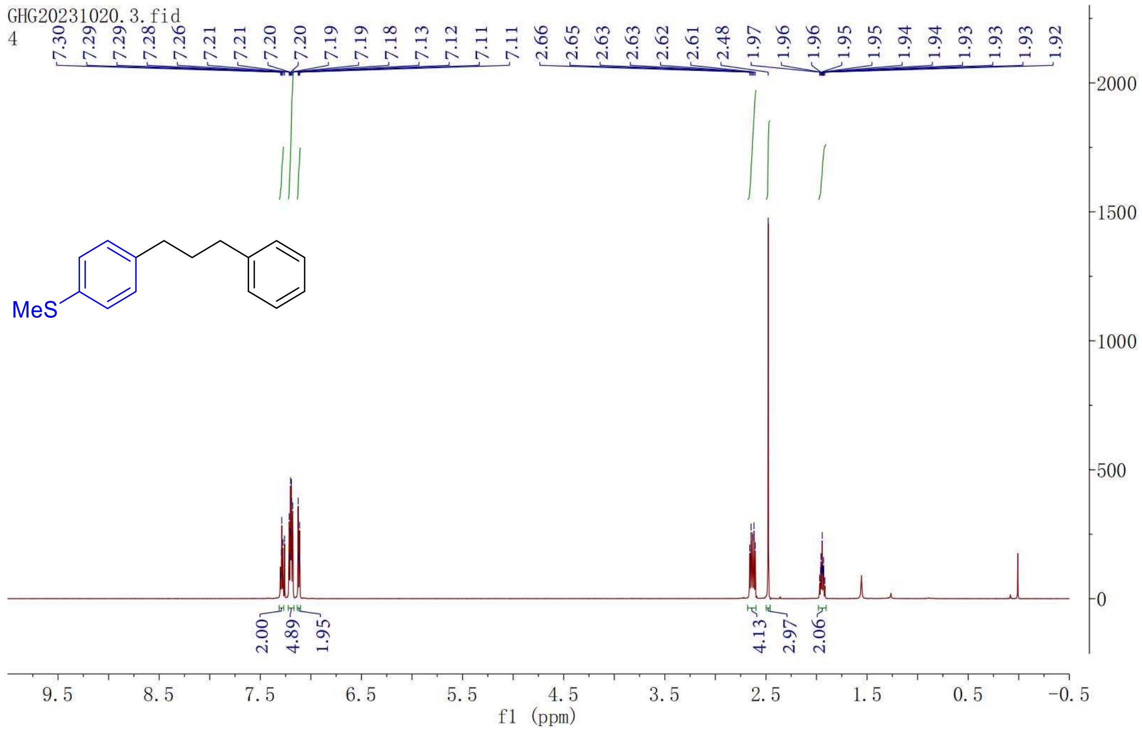

25, ^{13}C NMR (151 MHz, CDCl$_3$)

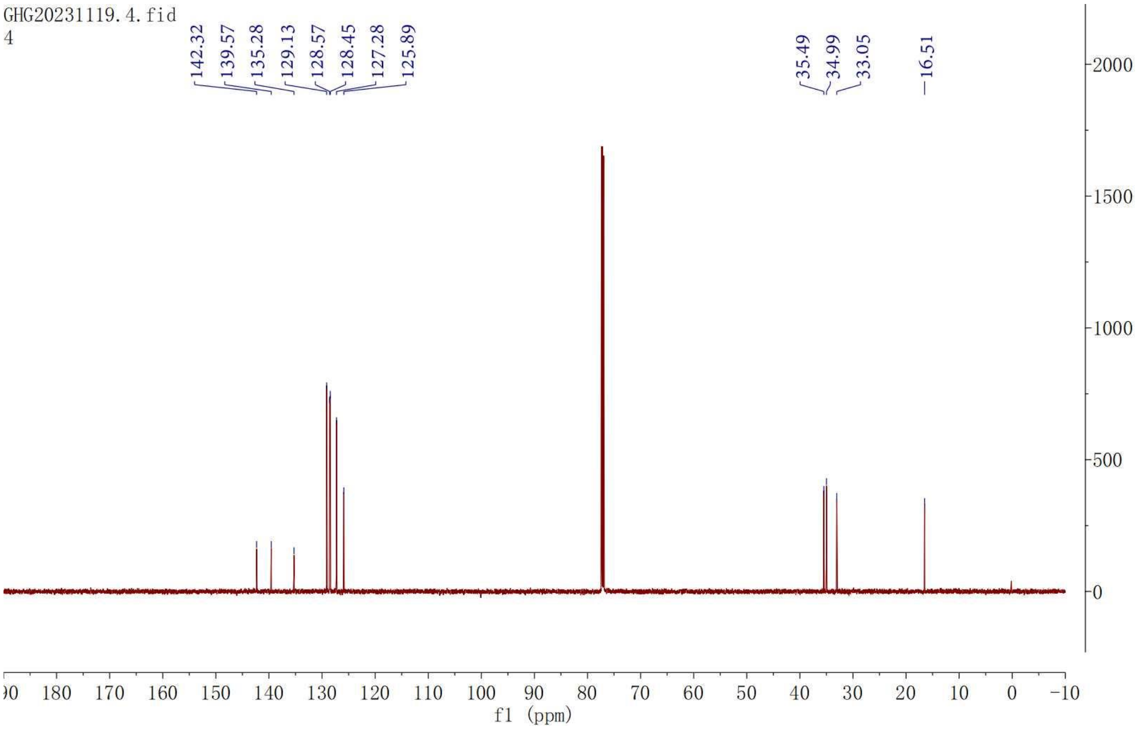

26, ^{1}H-NMR (600 MHZ, CDCl$_3$)

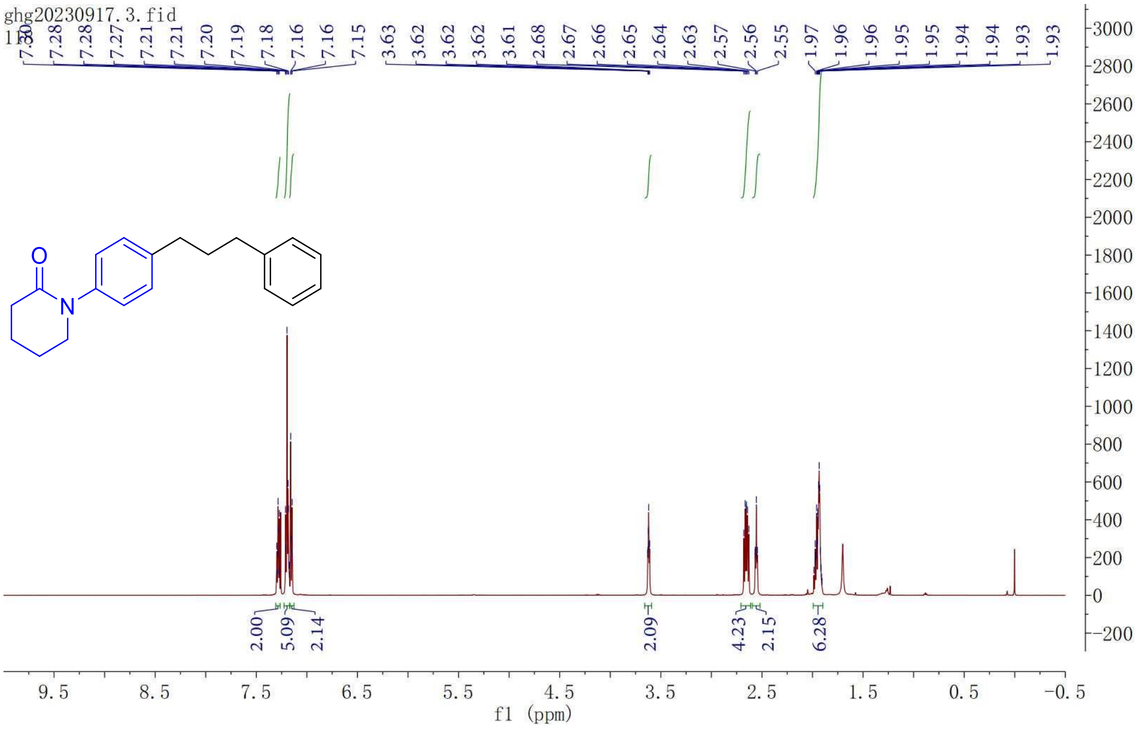

27, ^{1}H-NMR (600 MHZ, CDCl₃)

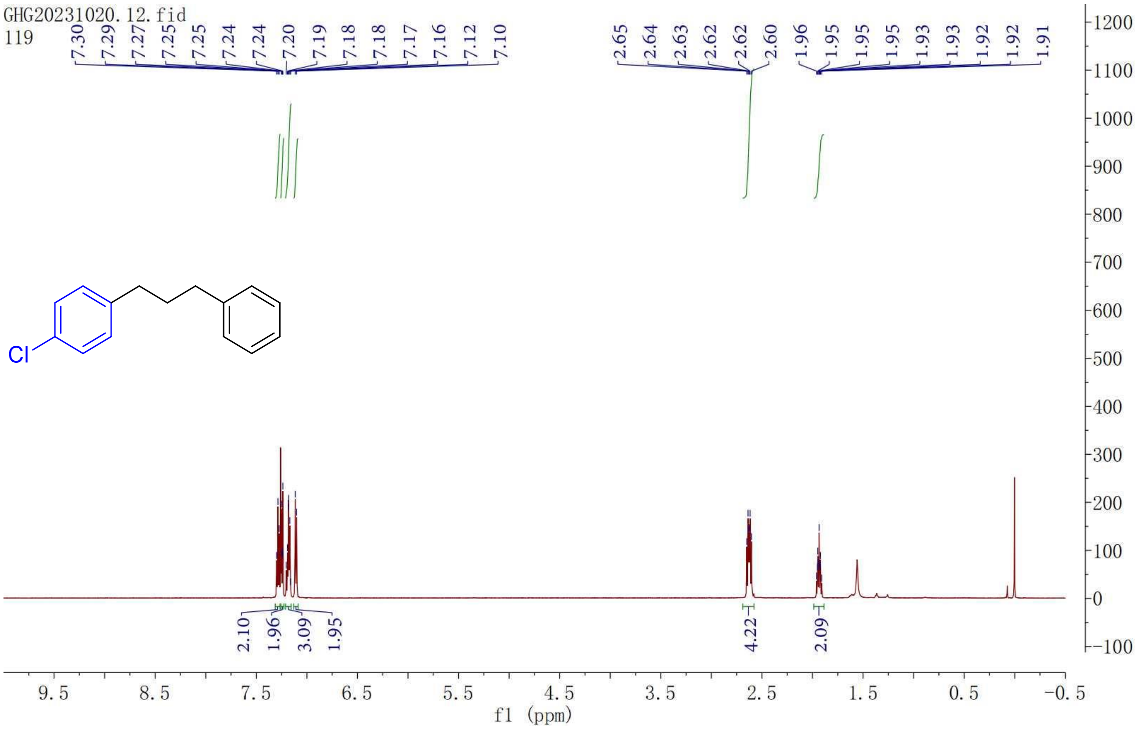

27, ^{13}C NMR (151 MHz, CDCl₃)

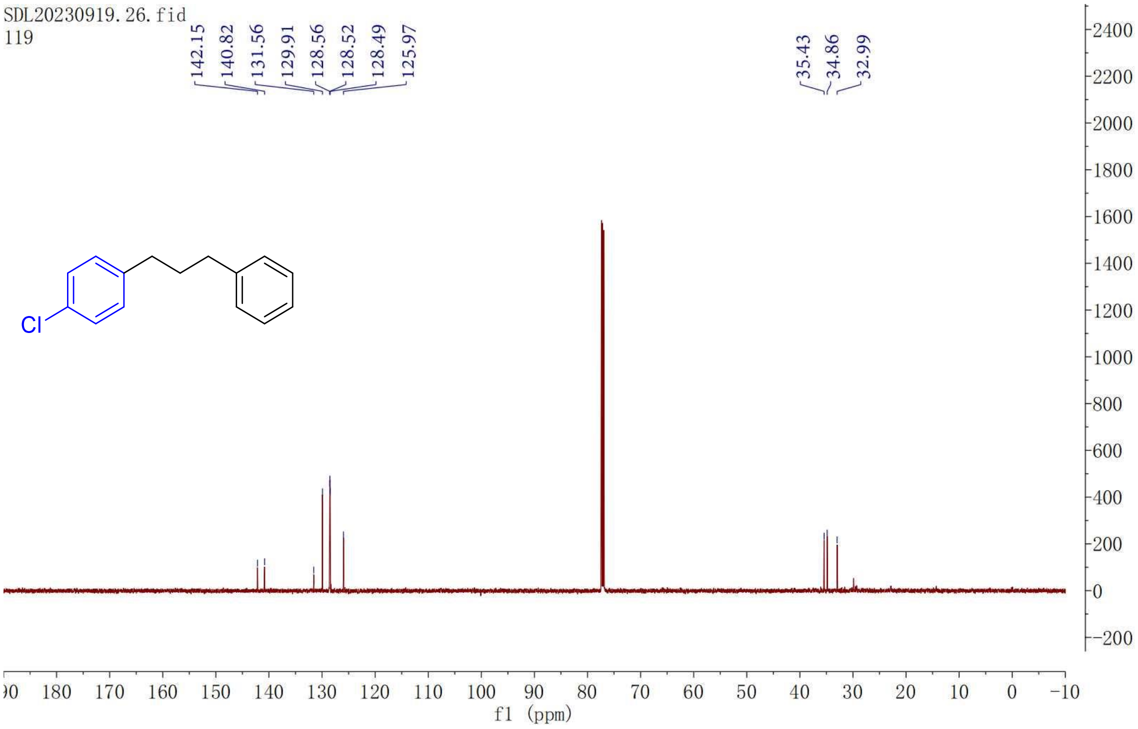

28, ^{1}H-NMR (600 MHZ, CDCl$_3$)

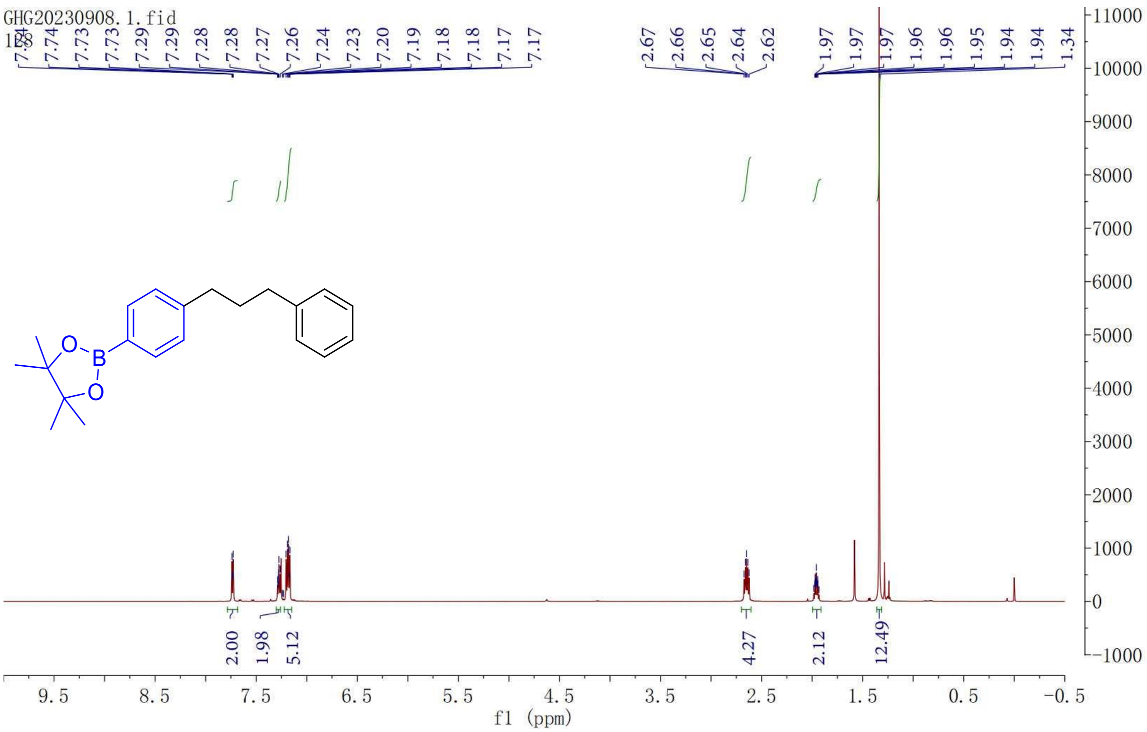

28, ^{13}C NMR (151 MHz, CDCl$_3$)

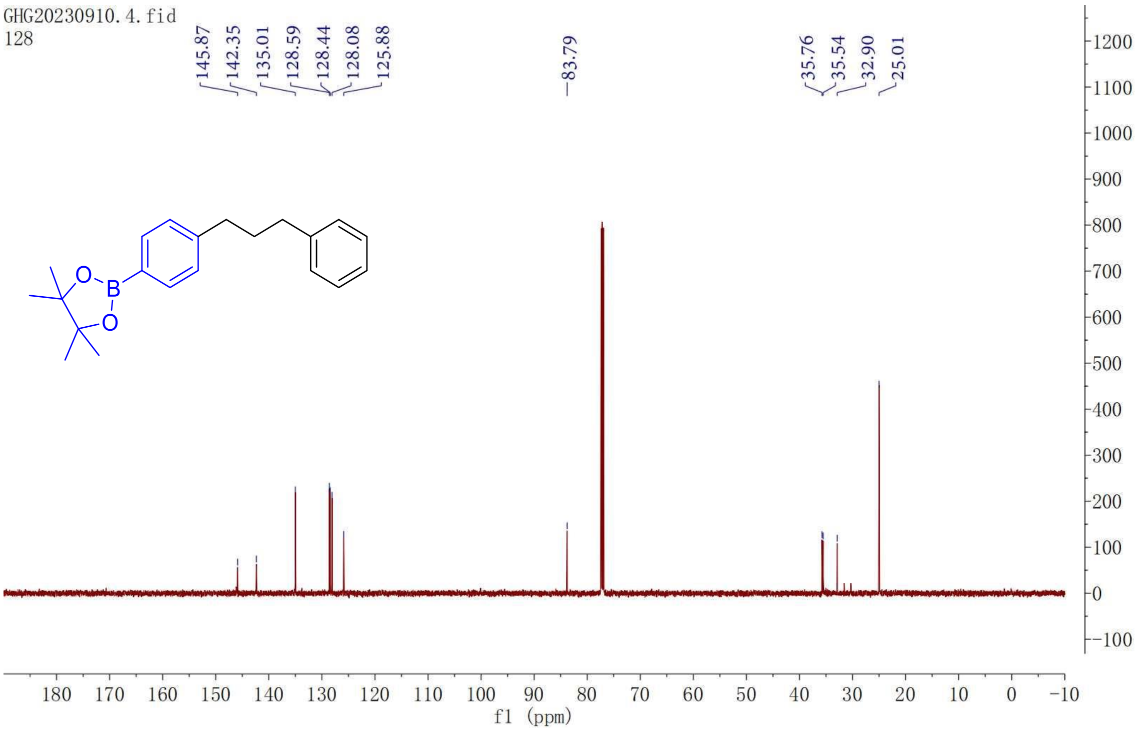

29, ^{1}H-NMR (600 MHZ, $CDCl_3$)

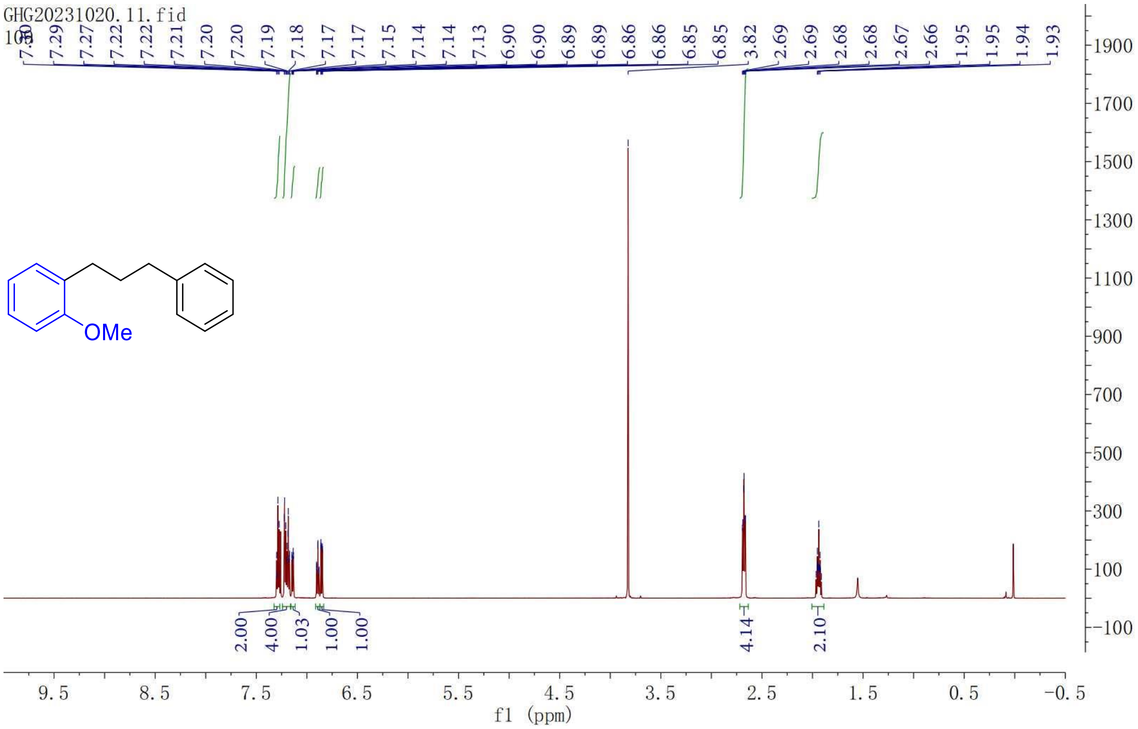

29, ^{13}C NMR (151 MHz, $CDCl_3$)

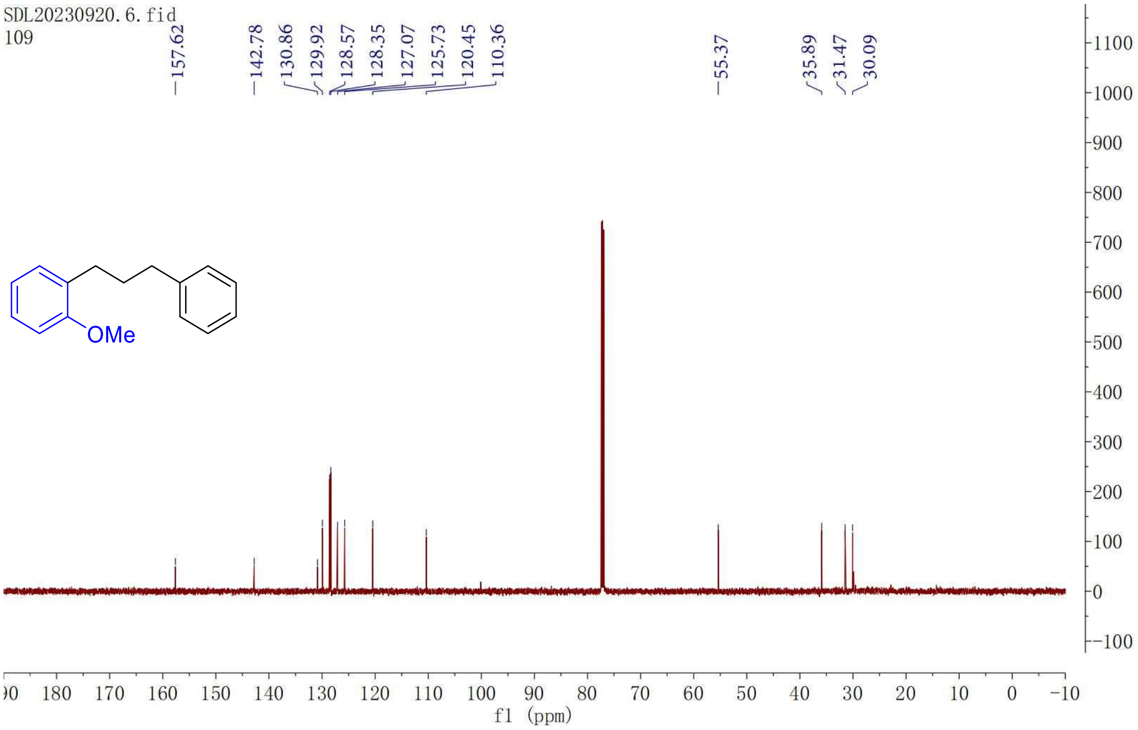

30, ^{1}H-NMR (600 MHZ, $CDCl_3$)

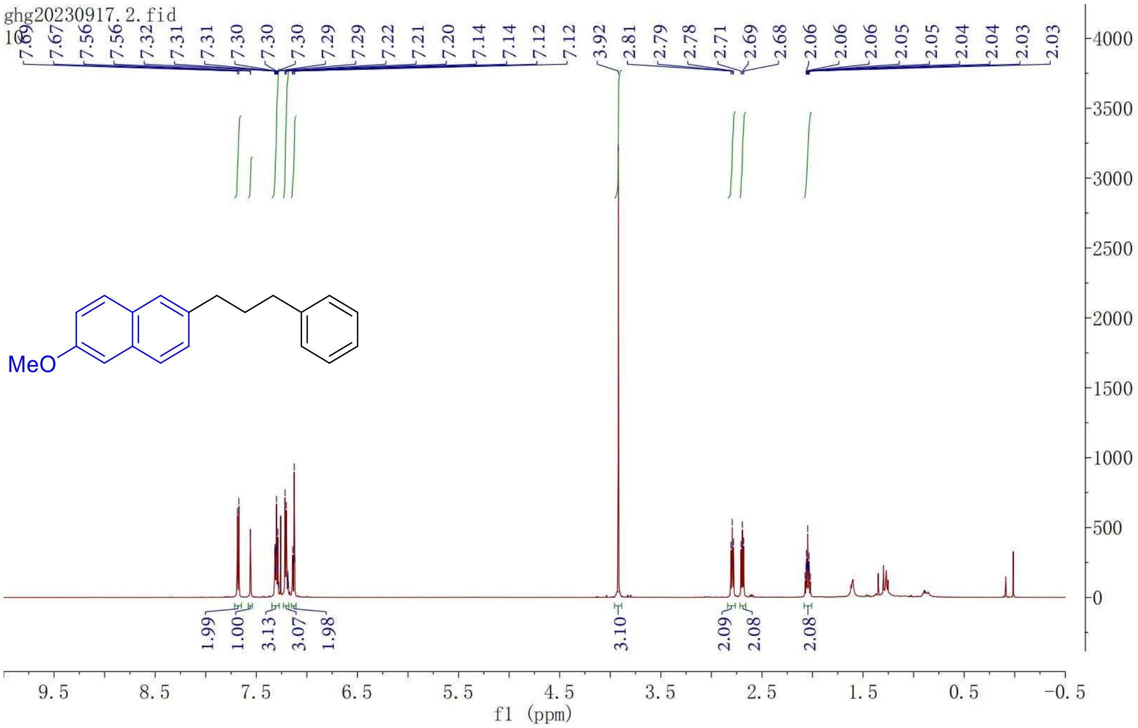

30, ^{13}C NMR (151 MHz, $CDCl_3$)

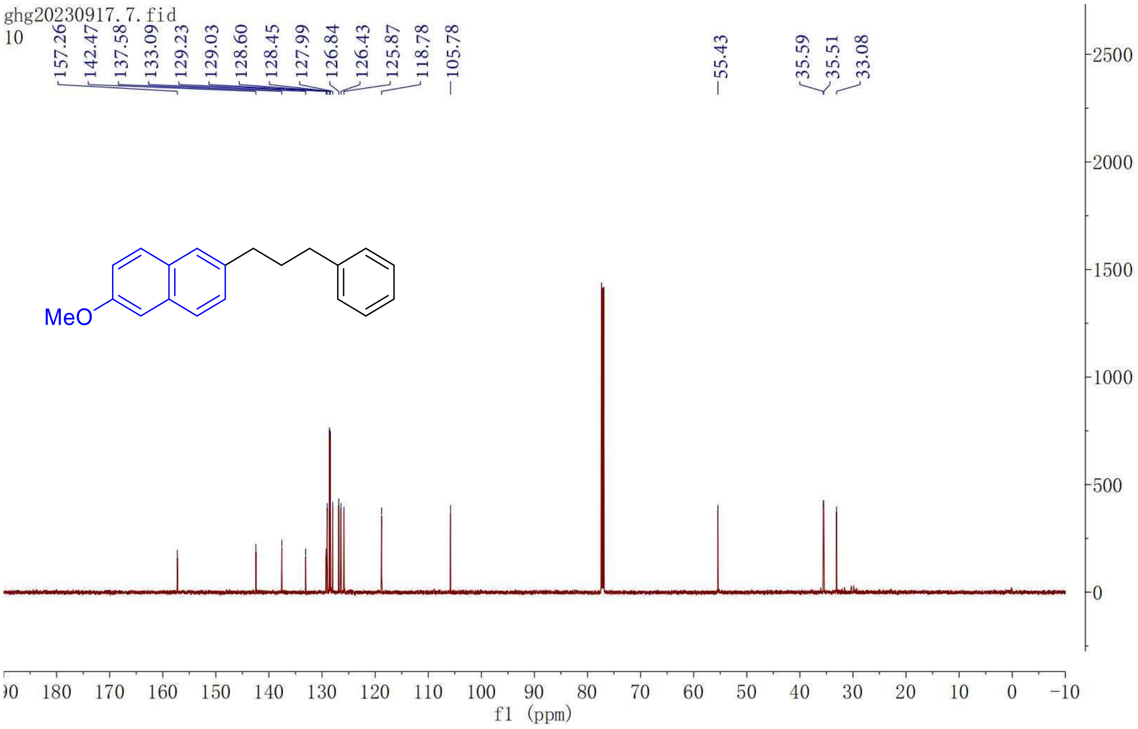

31, ^{1}H-NMR (600 MHZ, $CDCl_3$)

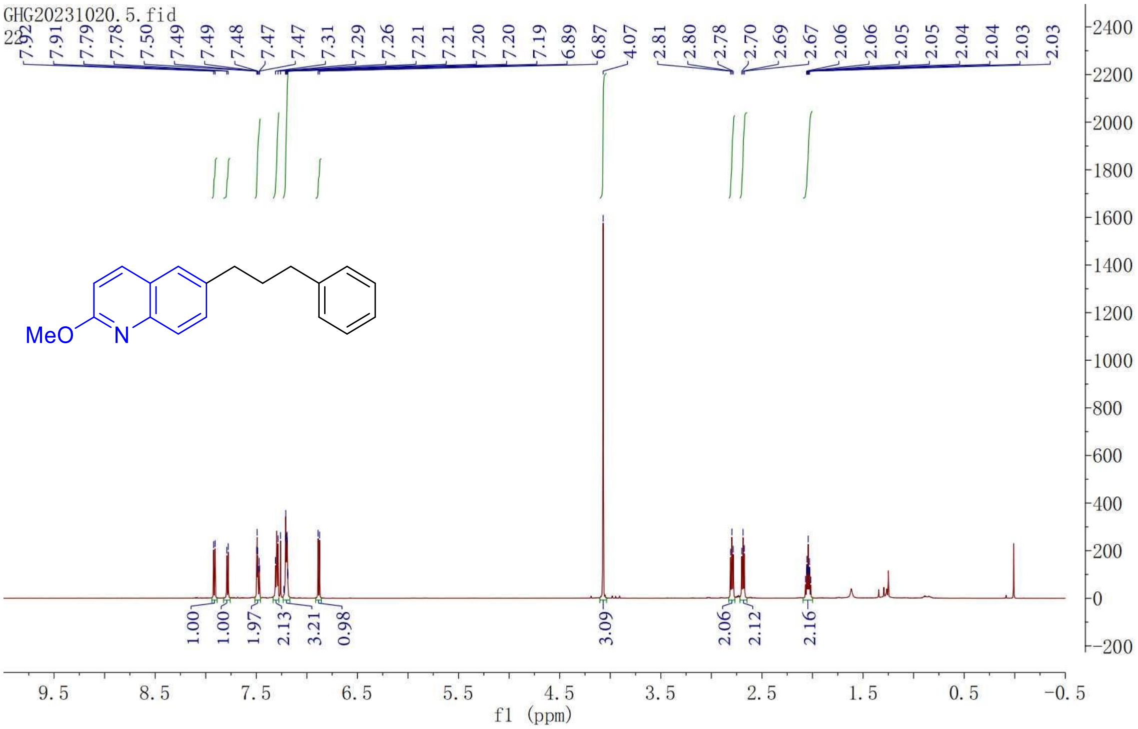

31, ^{13}C NMR (151 MHz, $CDCl_3$)

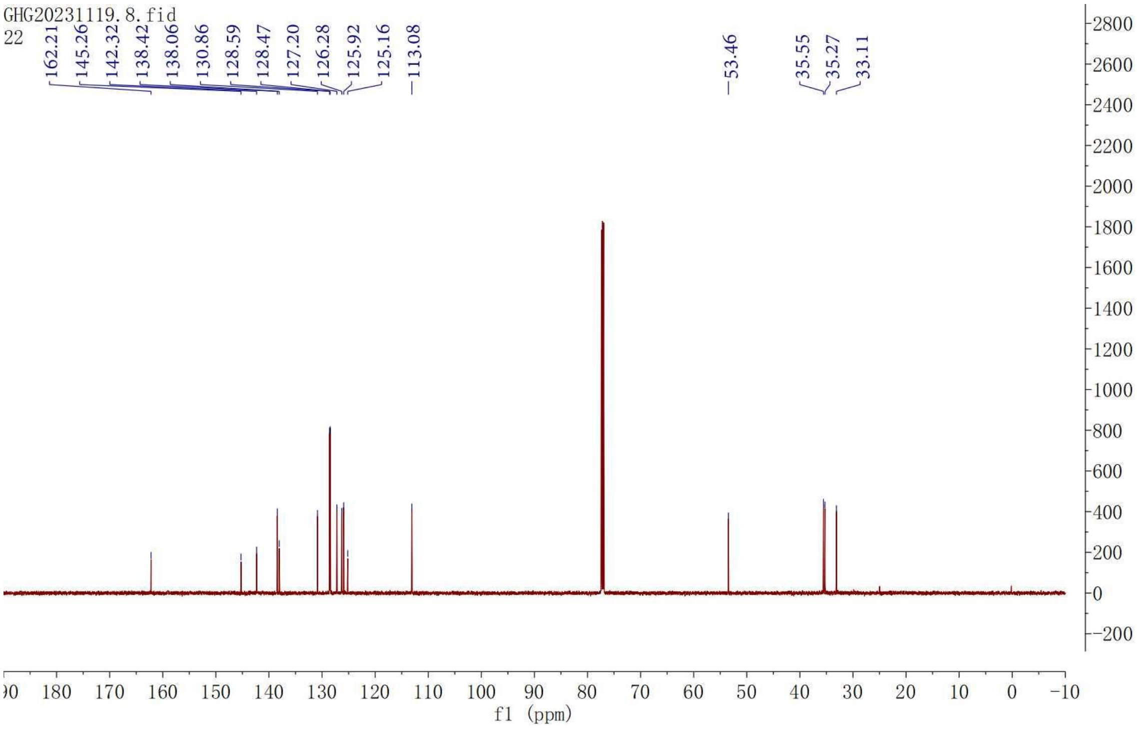

32, ^{1}H-NMR (600 MHZ, $CDCl_3$)

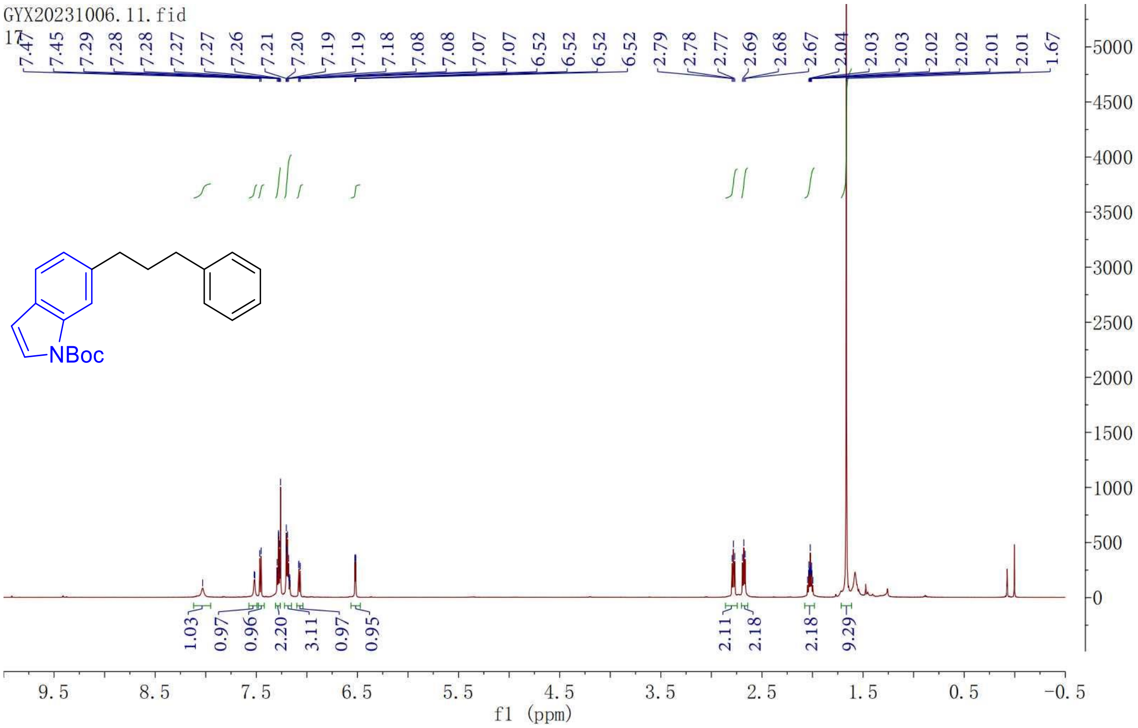

32, ^{13}C NMR (151 MHz, $CDCl_3$)

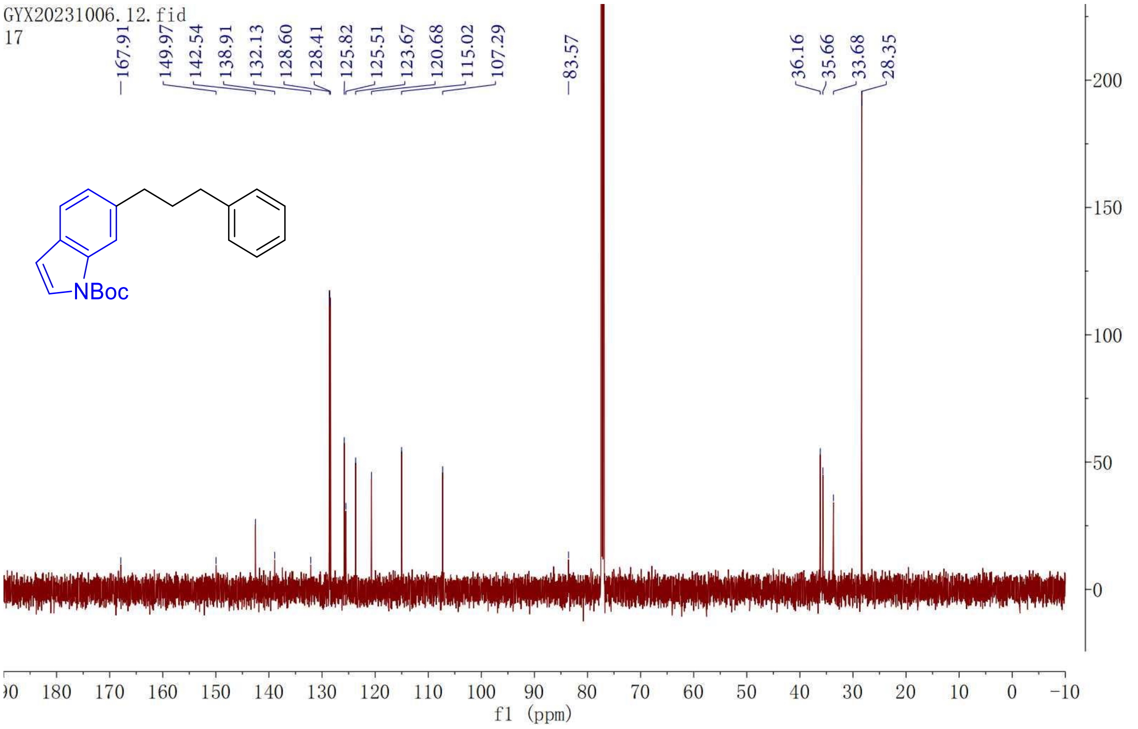

33, ^{1}H-NMR (600 MHZ, $CDCl_3$)

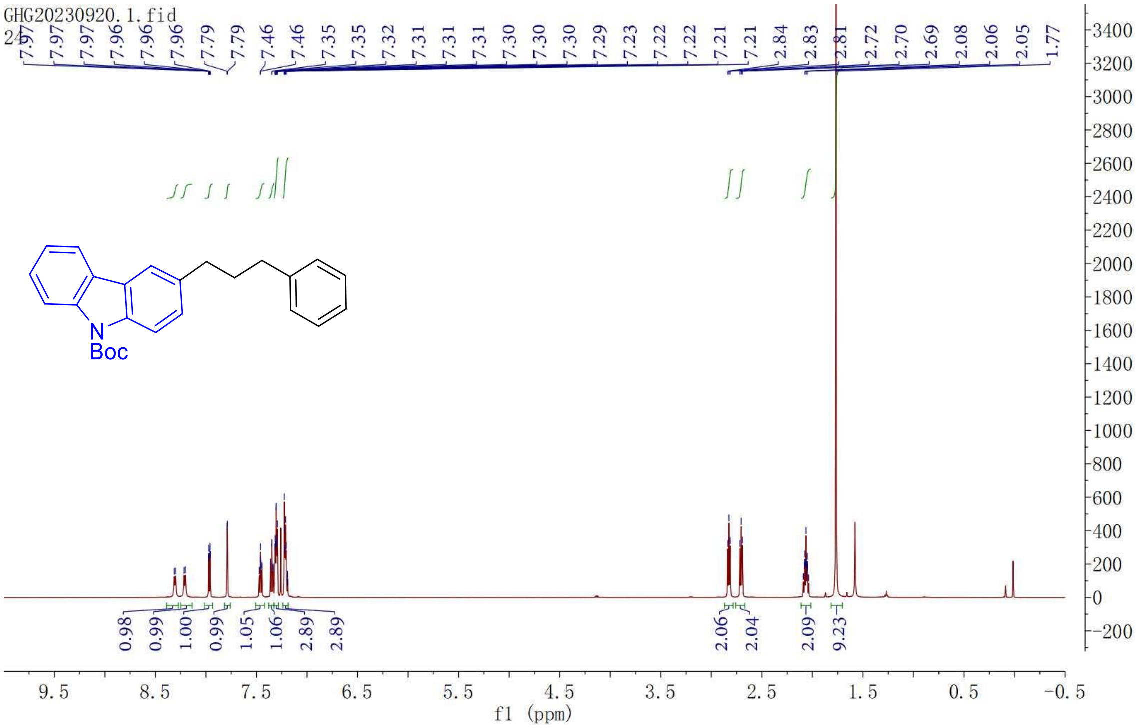

33, ^{13}C NMR (151 MHz, $CDCl_3$)

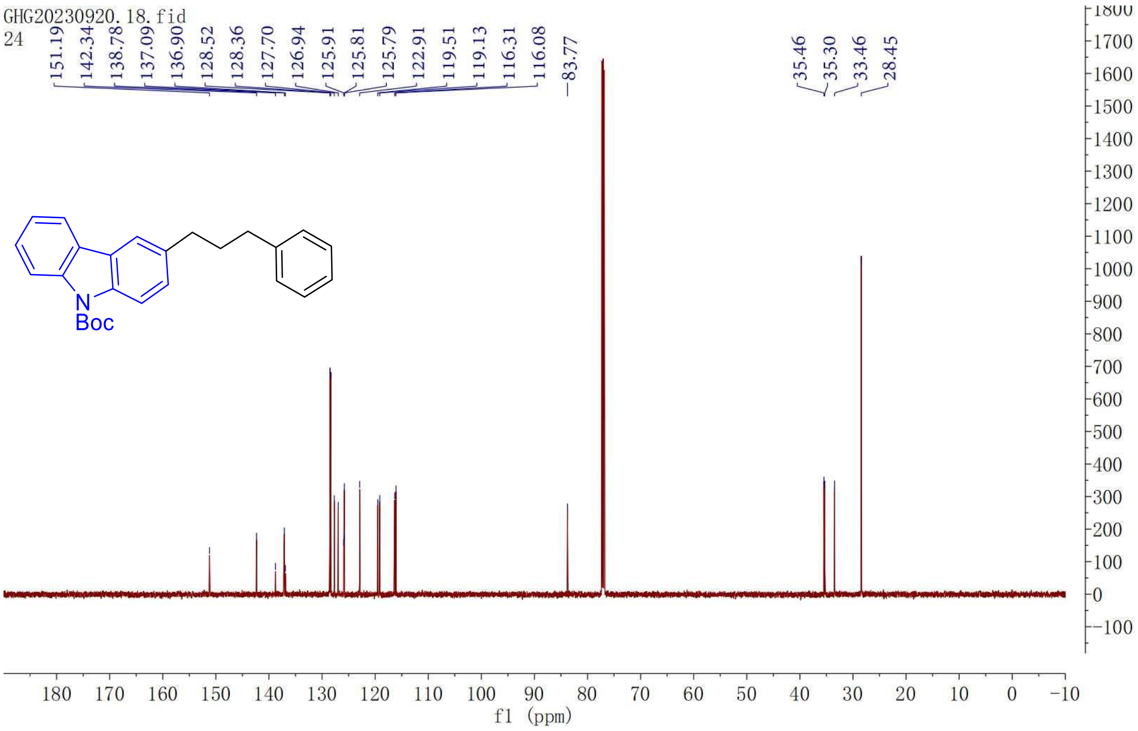

34, ^{1}H-NMR (600 MHZ, CDCl₃)

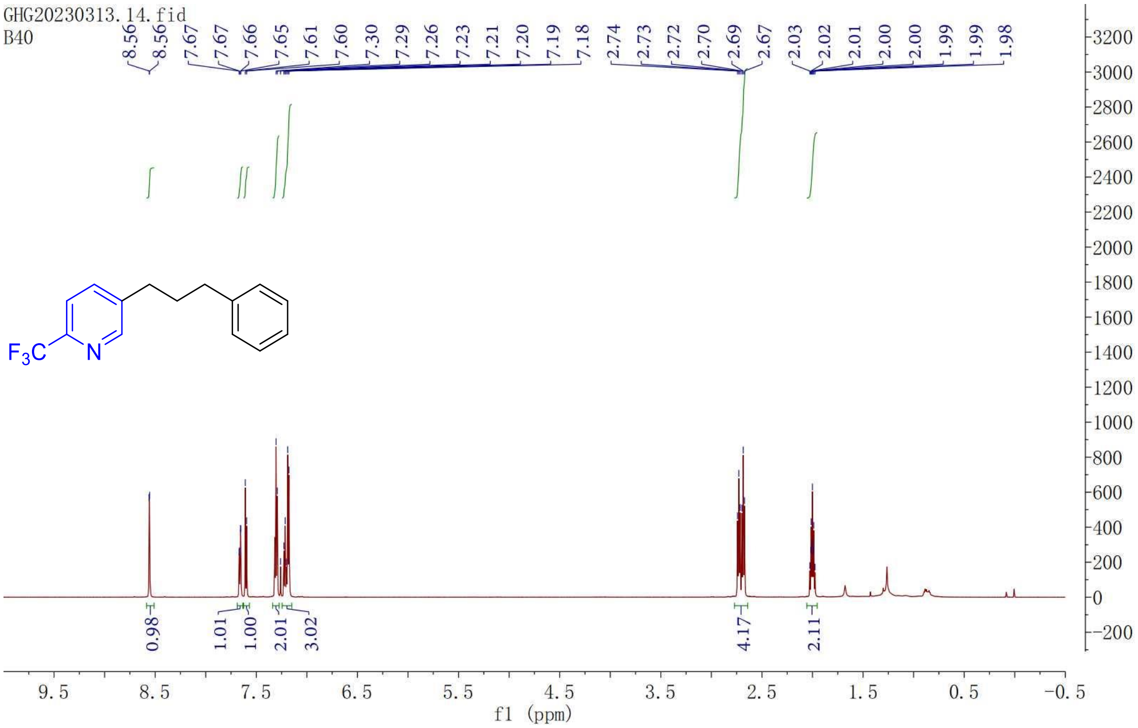

35, ^{1}H-NMR (600 MHZ, CDCl₃)

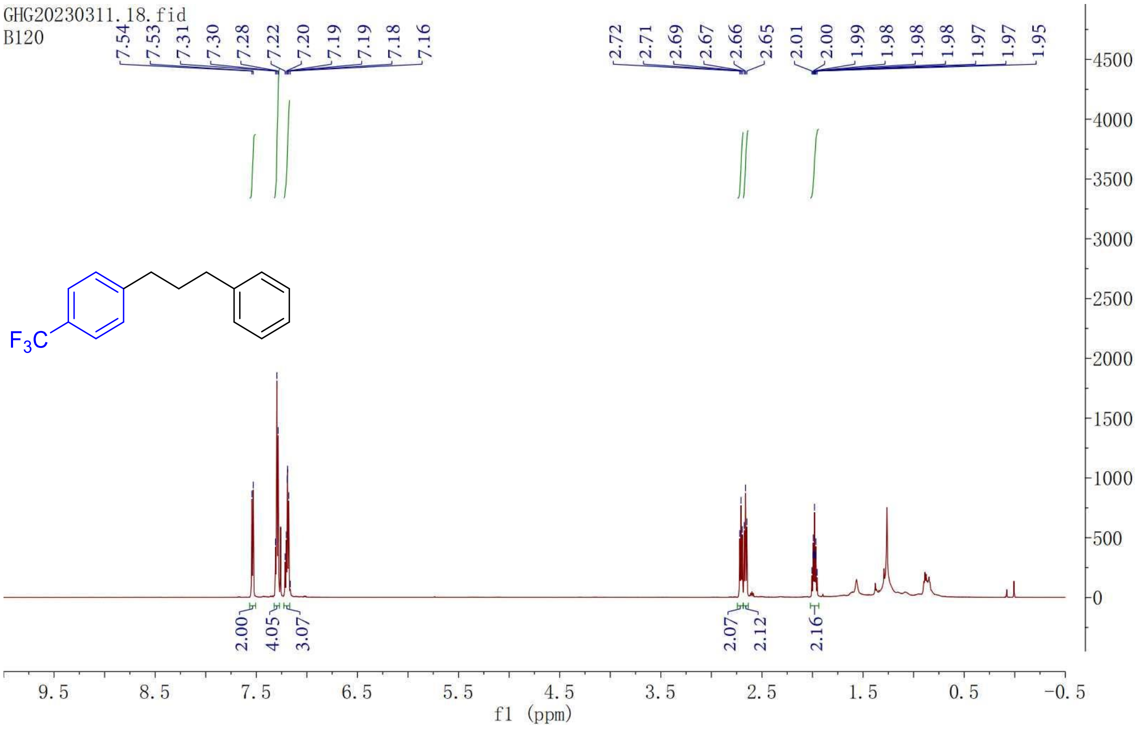

36, ^{1}H-NMR (600 MHZ, $CDCl_3$)

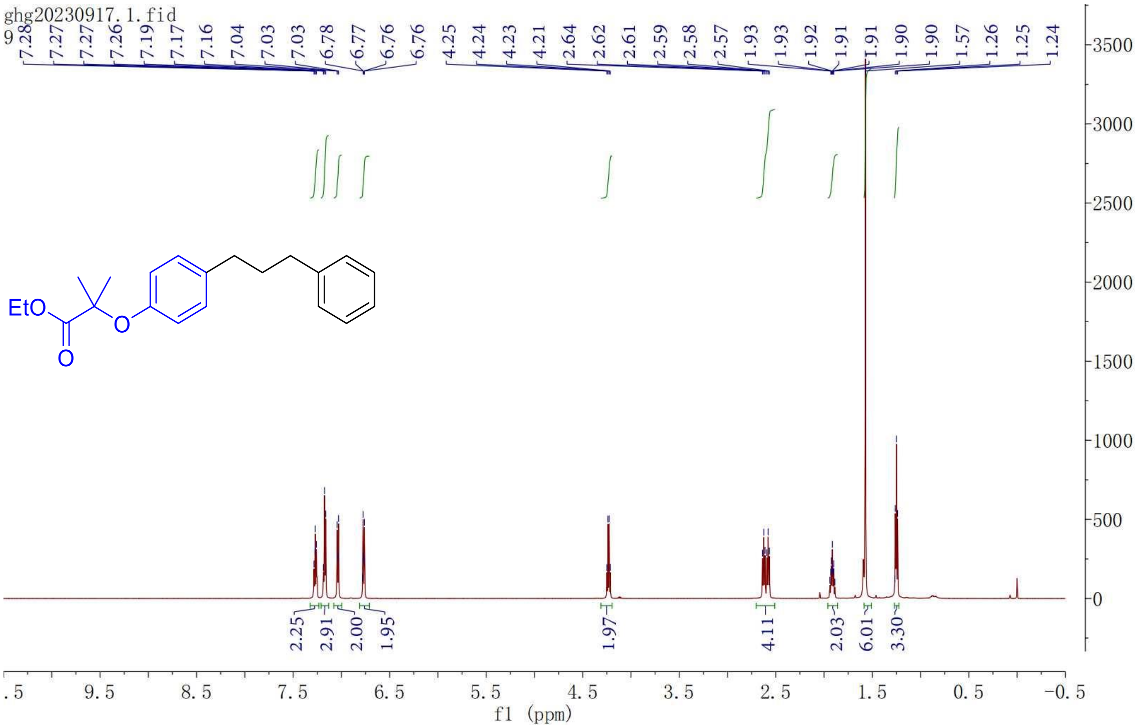

36, ^{13}C NMR (151 MHz, $CDCl_3$)

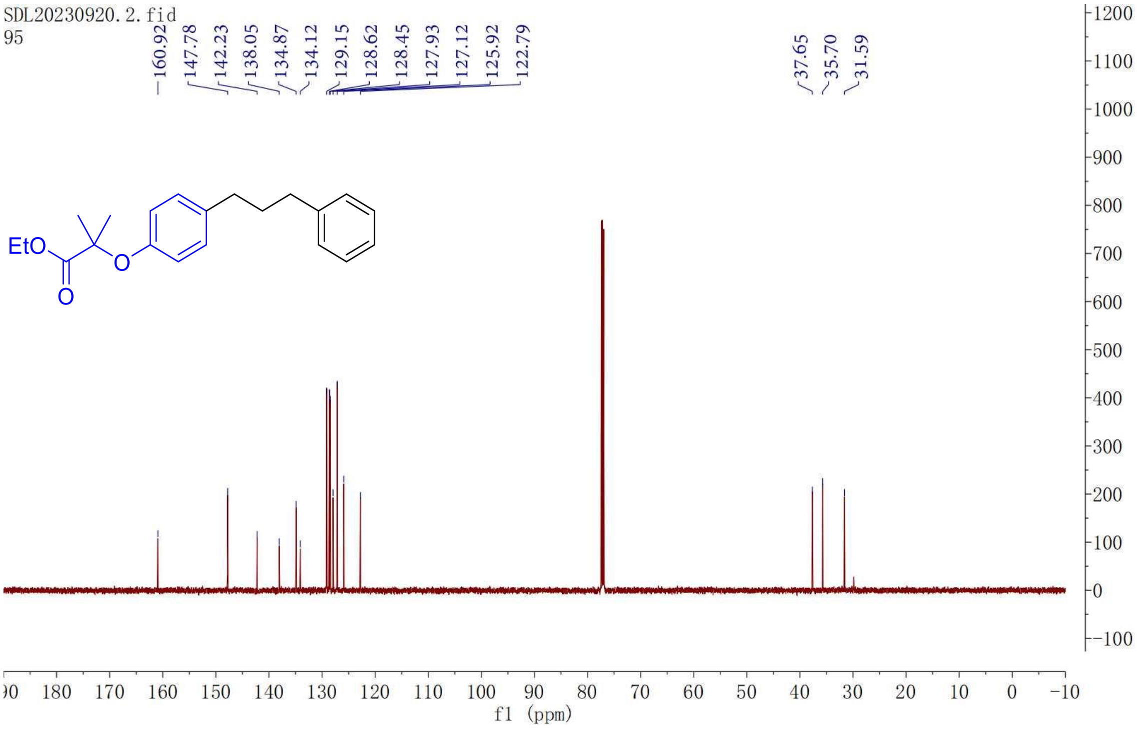

37, ¹H-NMR (600 MHZ, CDCl₃)

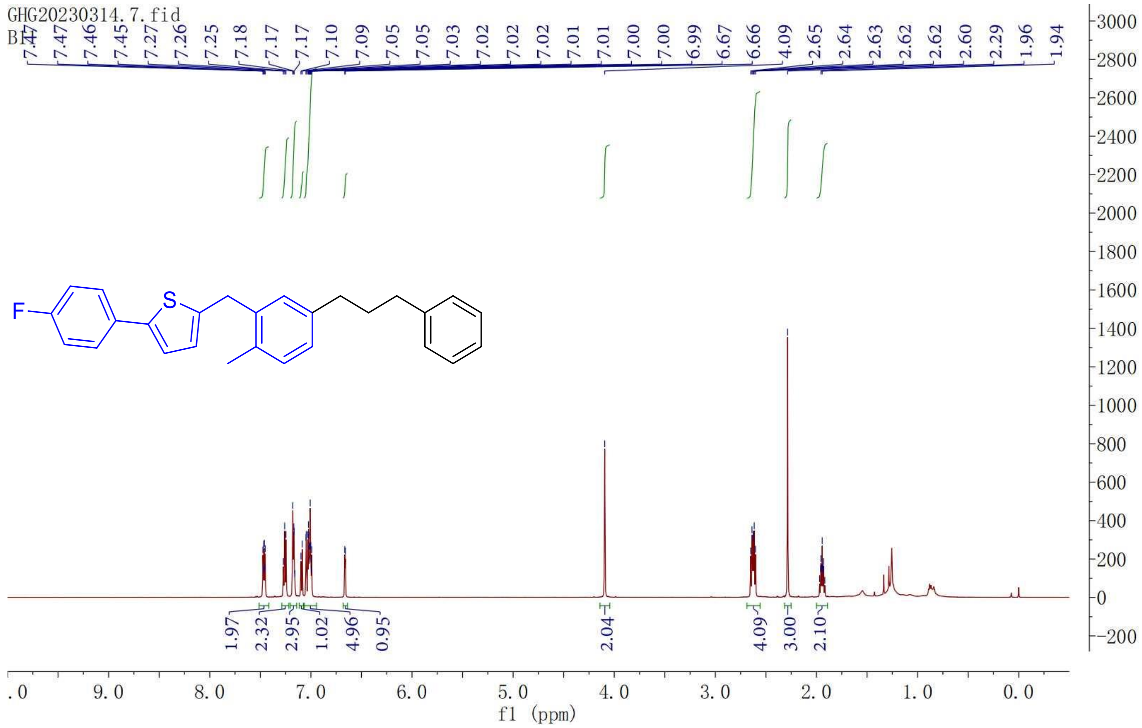

37, ¹³C NMR (151 MHz, CDCl₃)

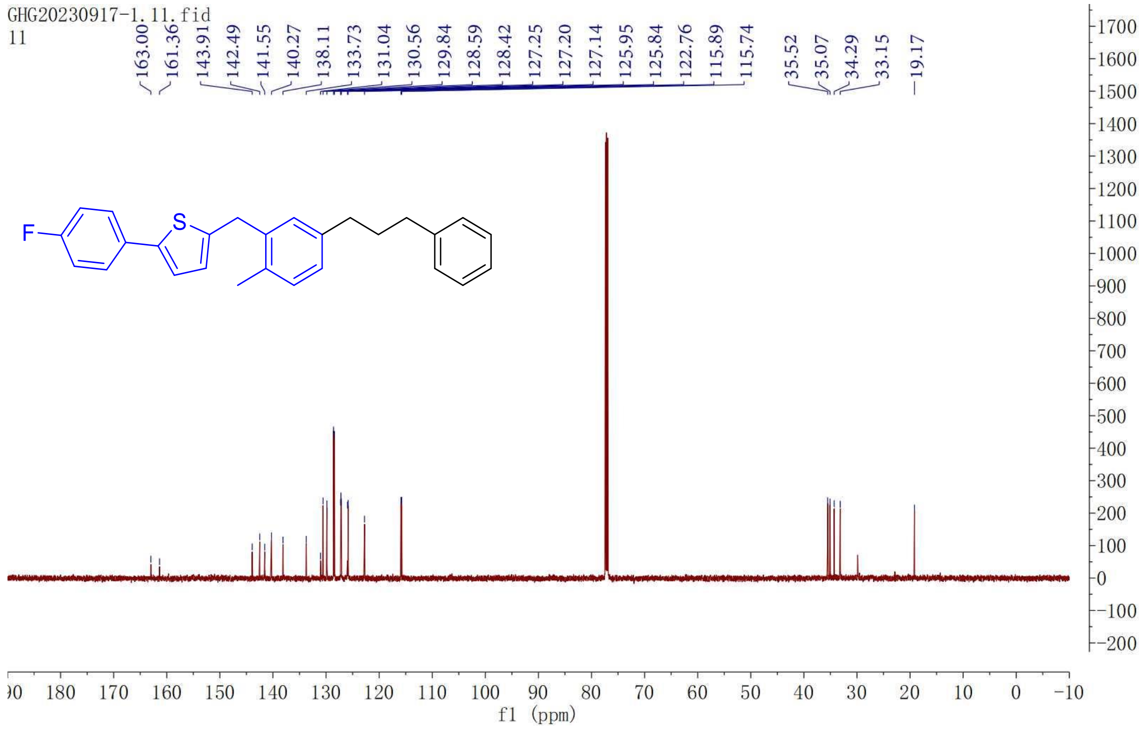

38, ¹H-NMR (600 MHZ, $CDCl_3$)

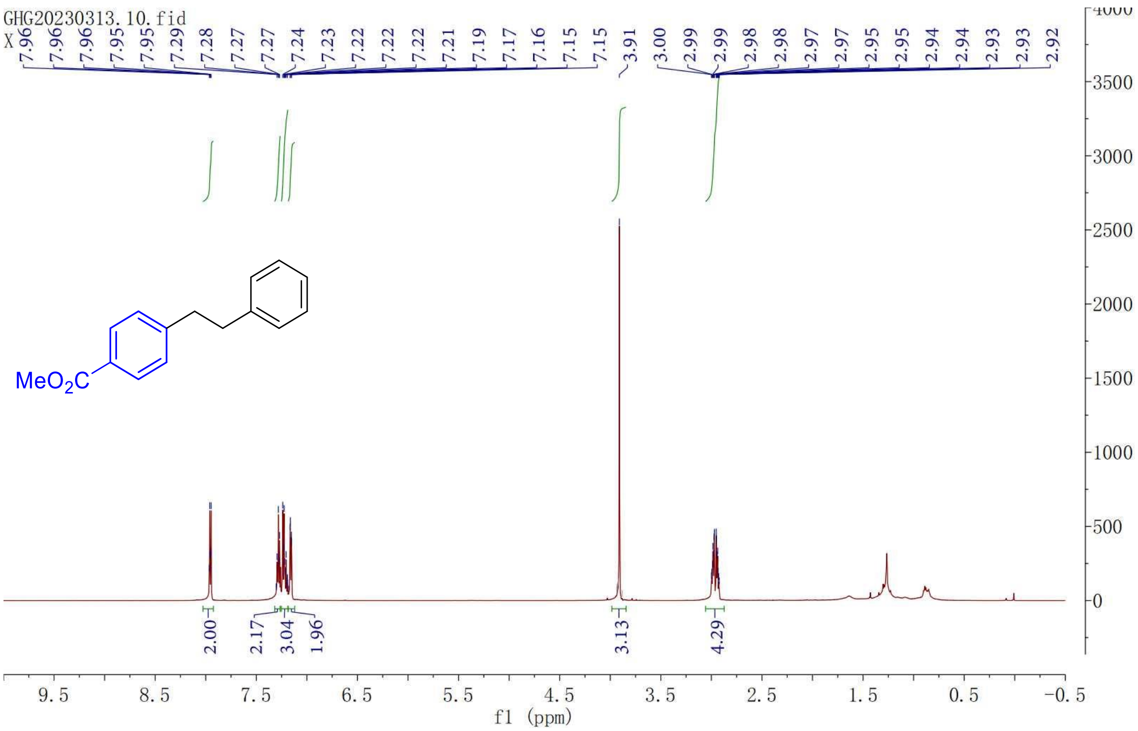

38, ¹³C NMR (151 MHz, $CDCl_3$)

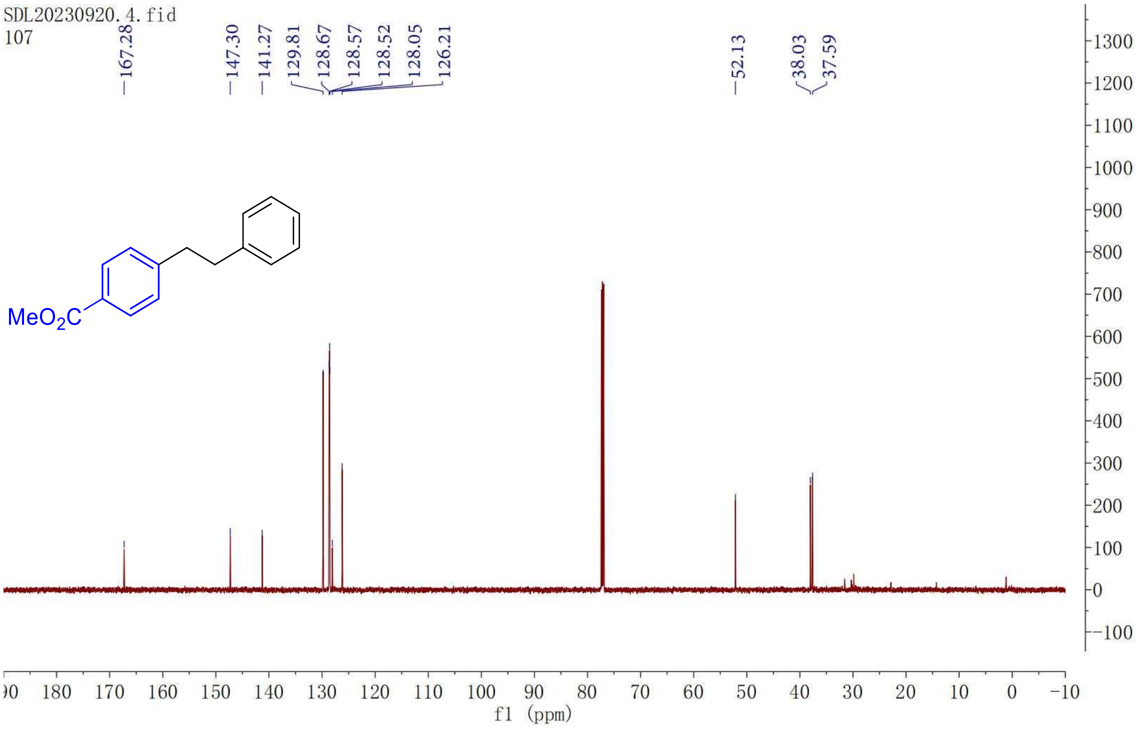

39, ^{1}H-NMR (600 MHZ, CDCl3)

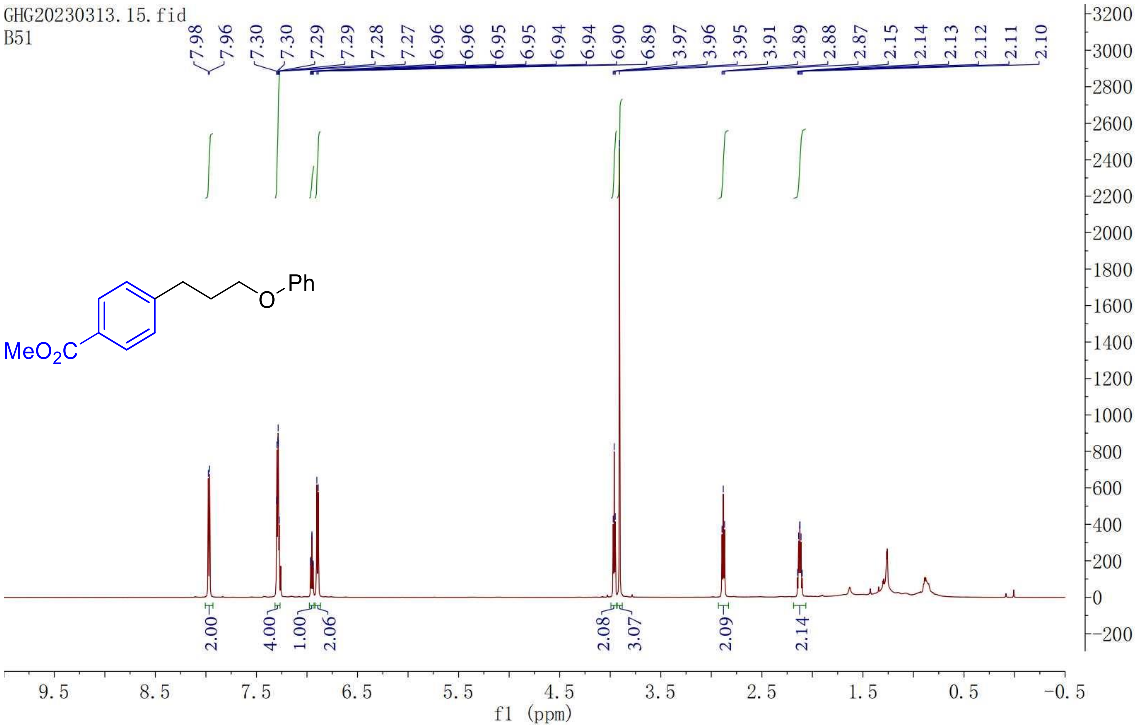

40, ^{1}H-NMR (600 MHZ, CDCl3)

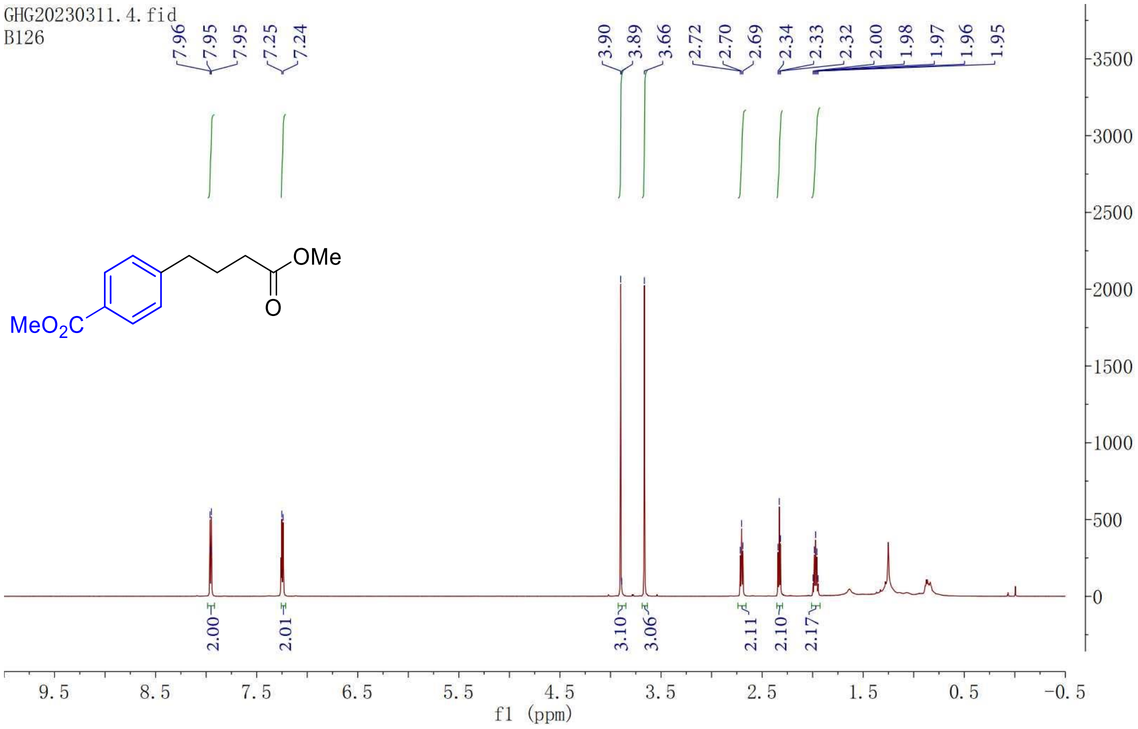

40, ^{13}C NMR (151 MHz, CDCl$_3$)

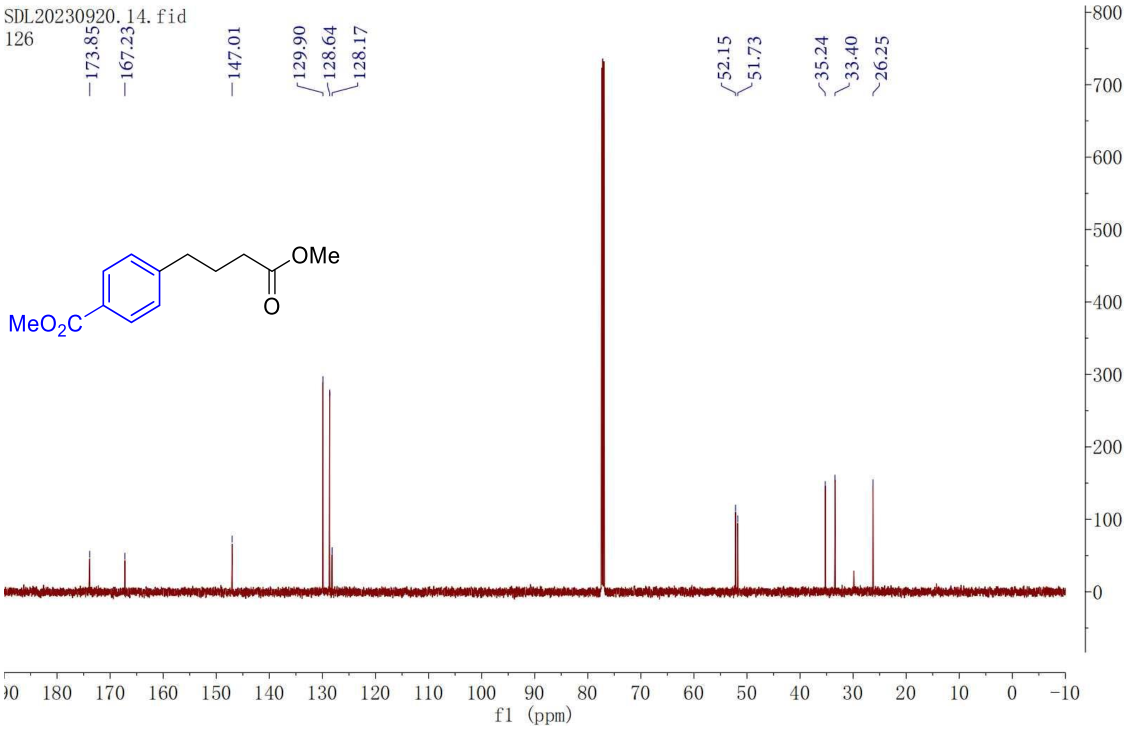

41, ^{1}H-NMR (600 MHZ, CDCl$_3$)

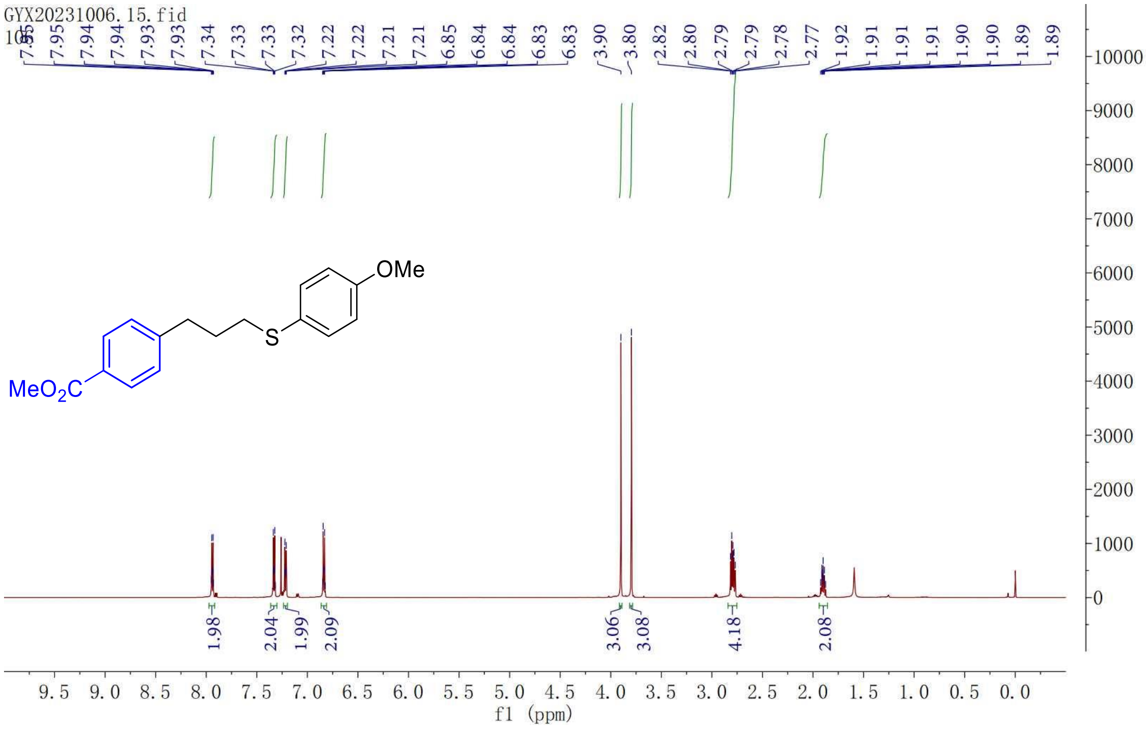

41, ^{13}C NMR (151 MHz, CDCl$_3$)

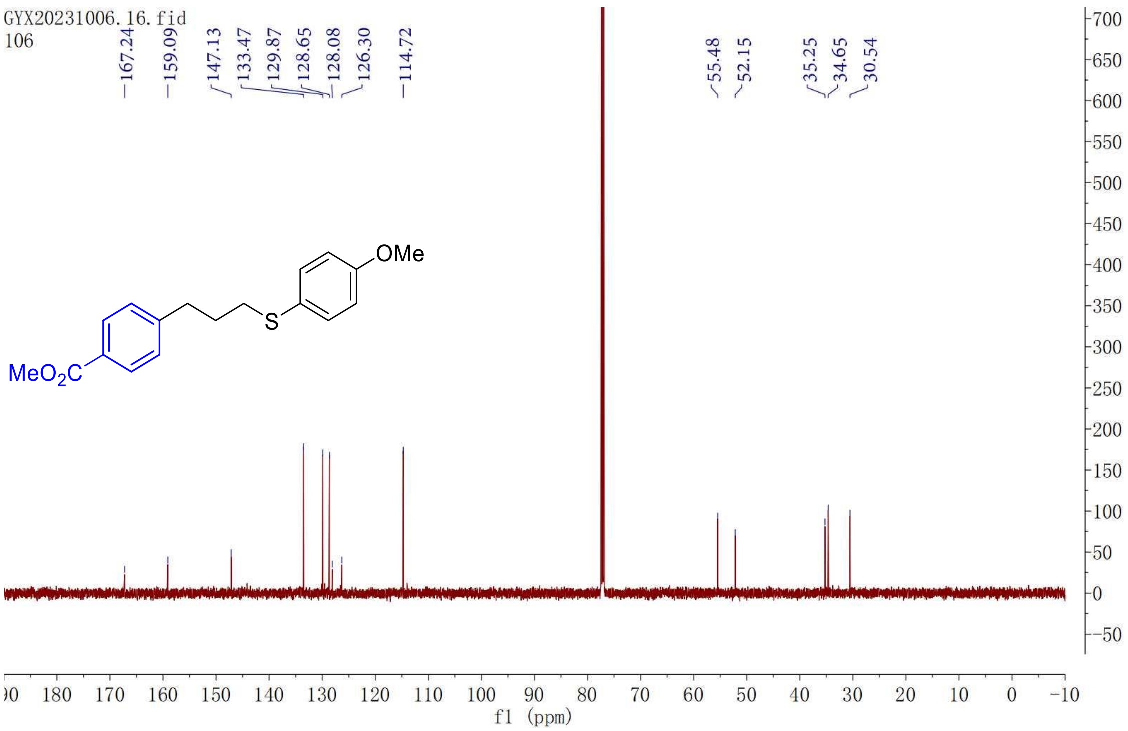

42, ^{1}H-NMR (600 MHZ, CDCl$_3$)

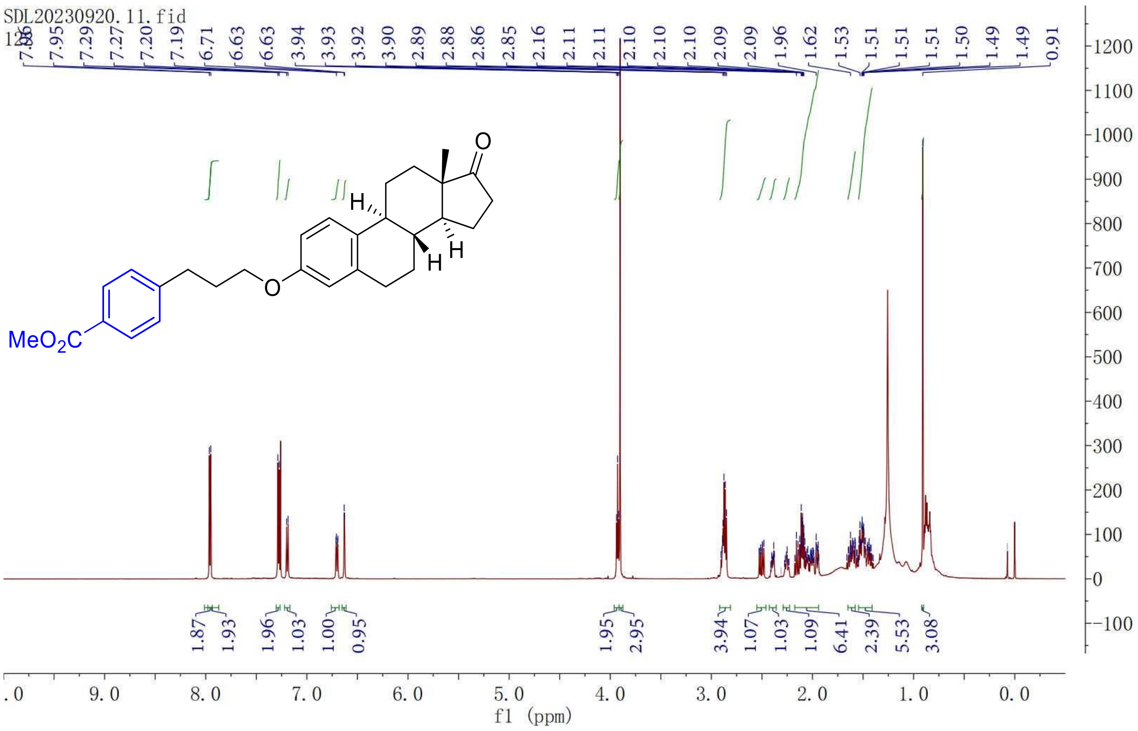

42, ^{13}C NMR (151 MHz, $CDCl_3$)

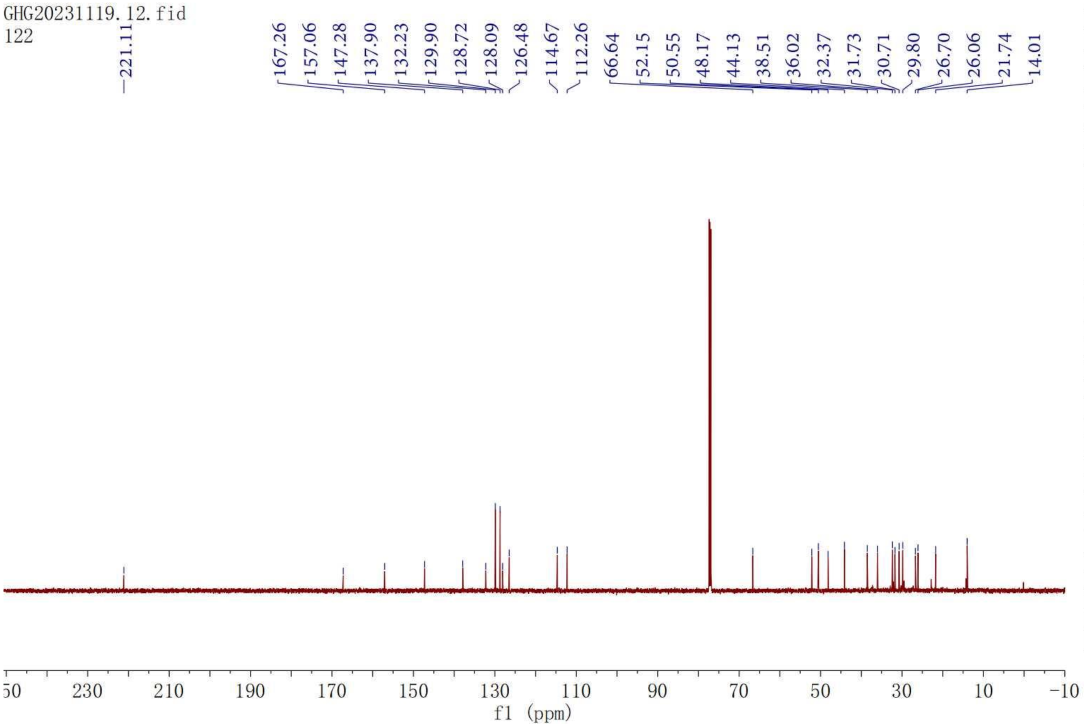

43, ^{1}H-NMR (600 MHZ, $CDCl_3$)

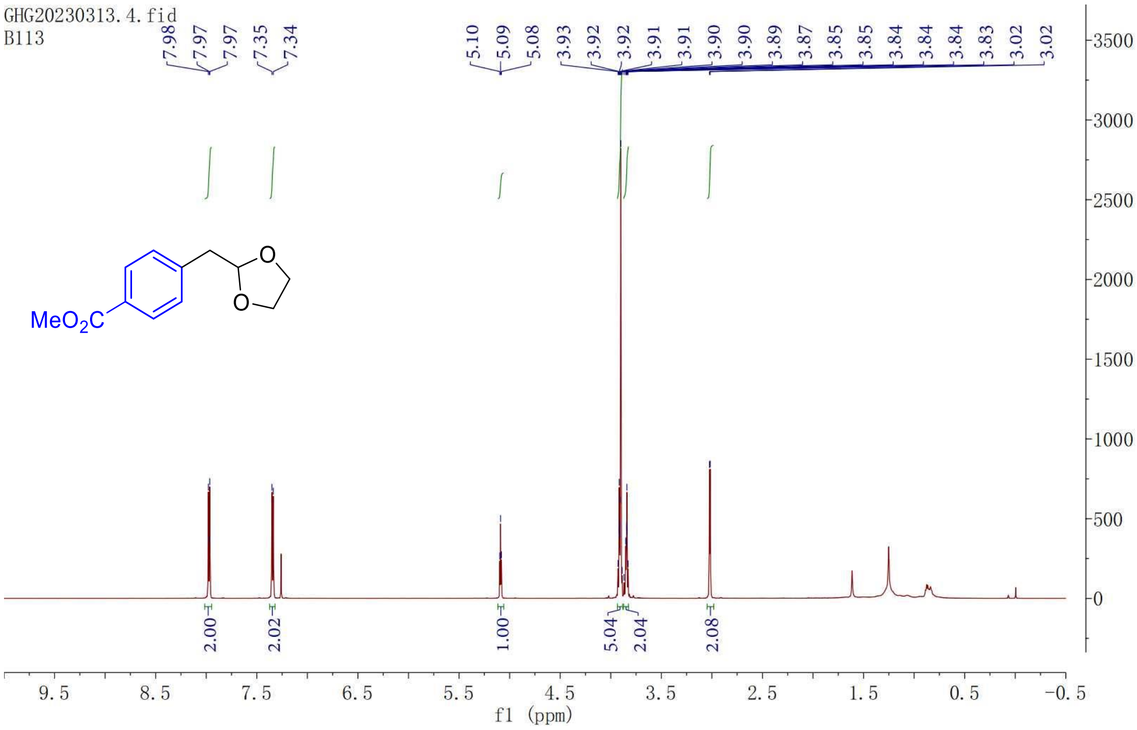

43, ^{13}C NMR (151 MHz, $CDCl_3$)

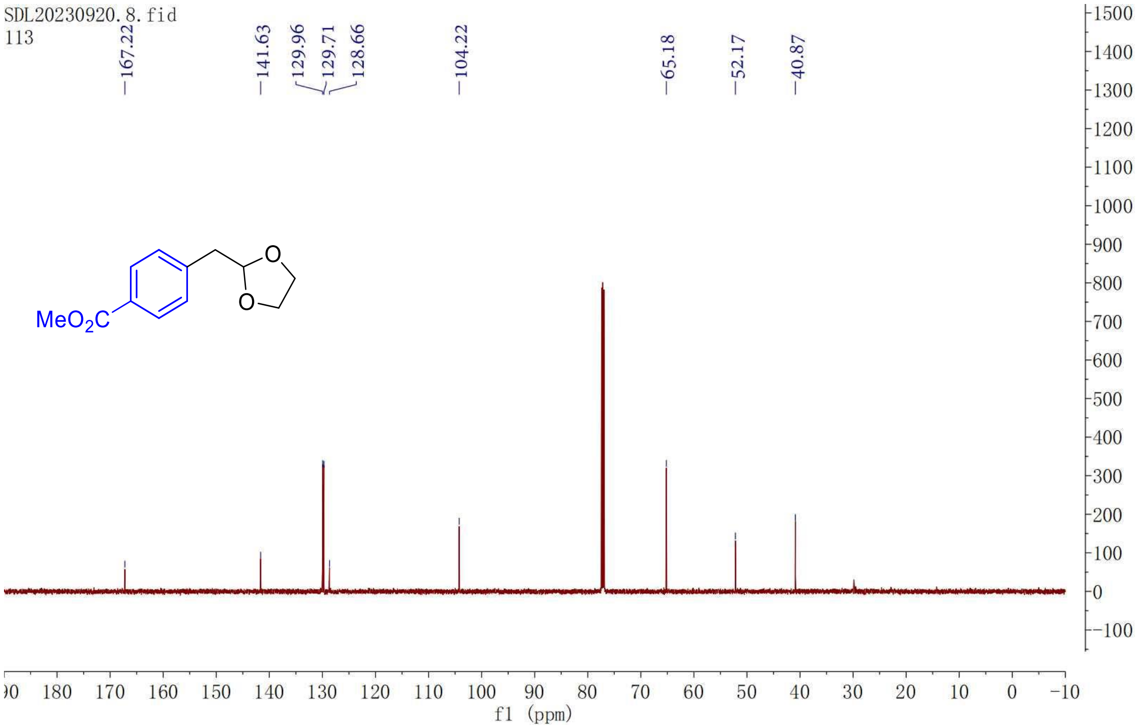

44, ^{1}H-NMR (600 MHZ, $CDCl_3$)

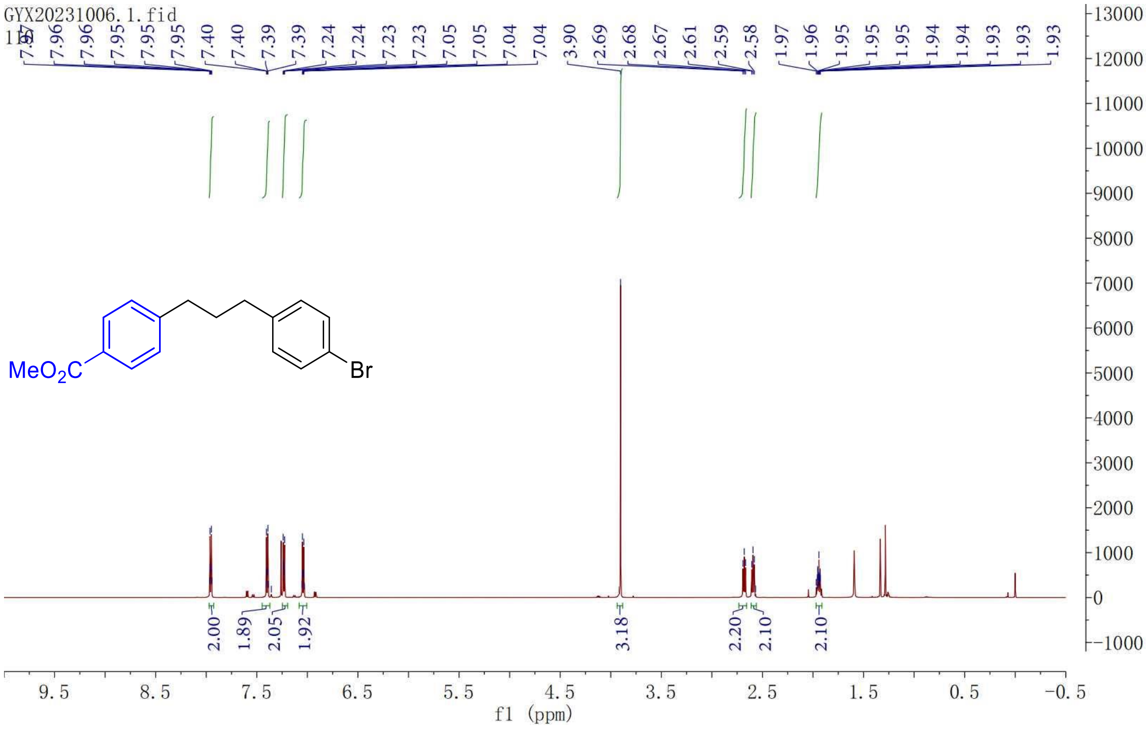

44, ^{13}C NMR (151 MHz, CDCl$_3$)

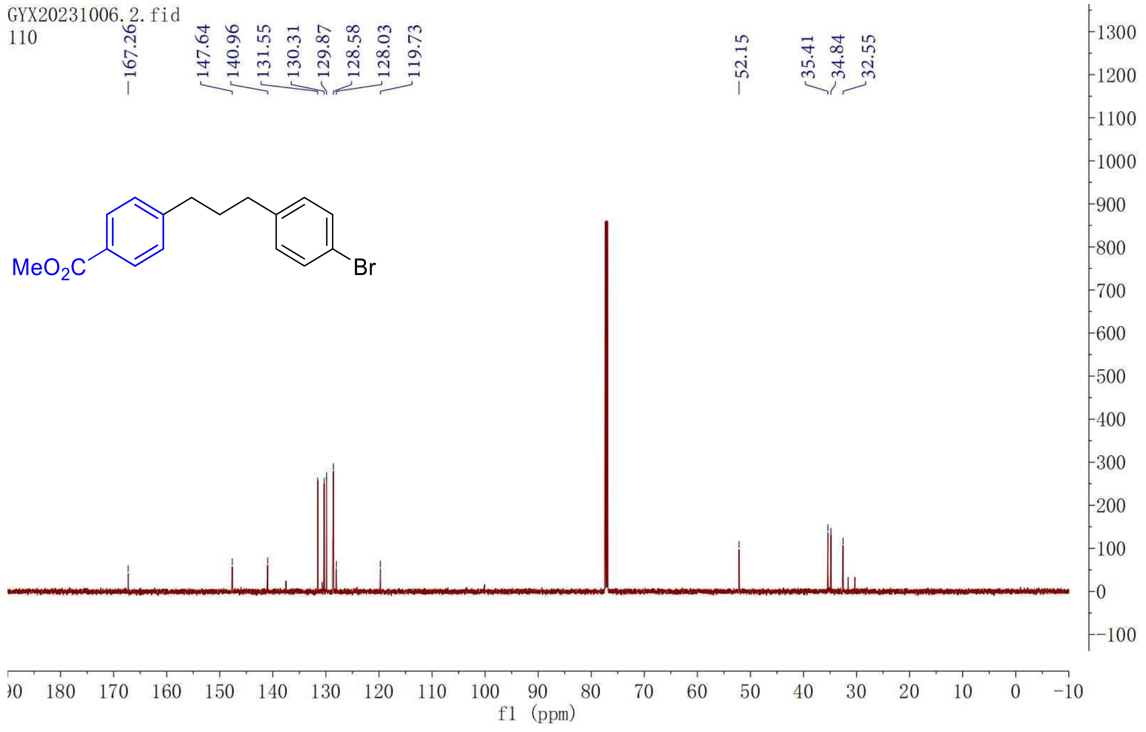

45, ^{1}H-NMR (600 MHZ, CDCl$_3$)

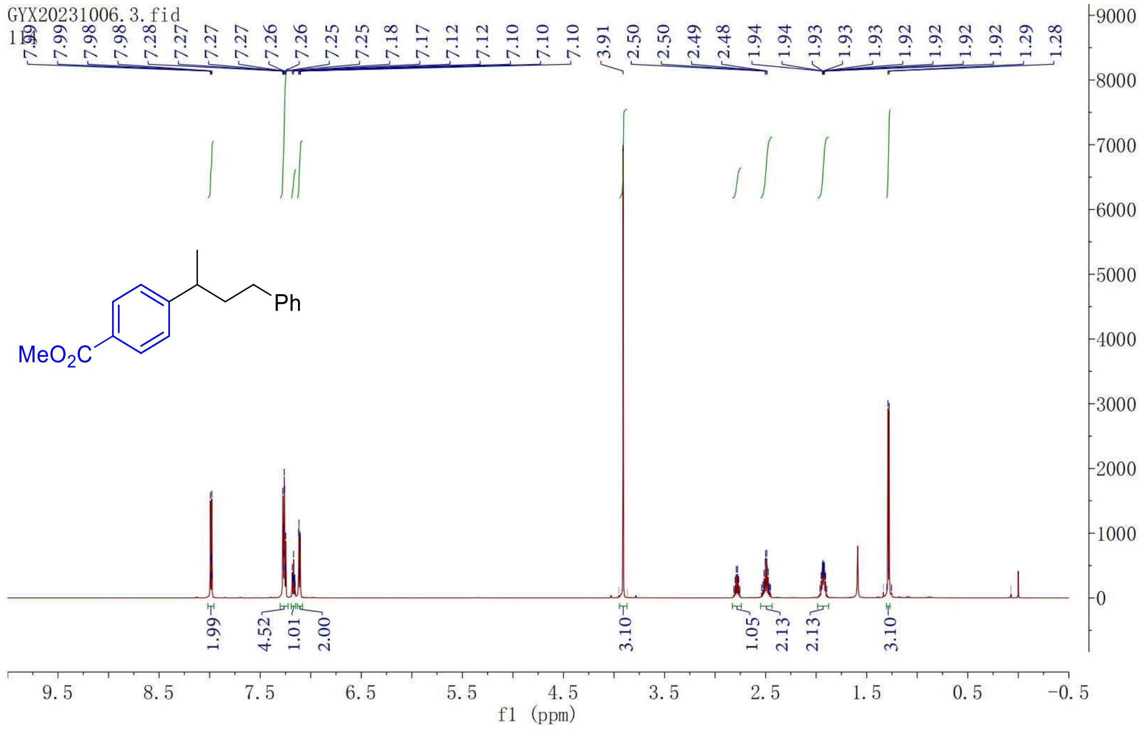

45, ^{13}C NMR (151 MHz, CDCl$_3$)

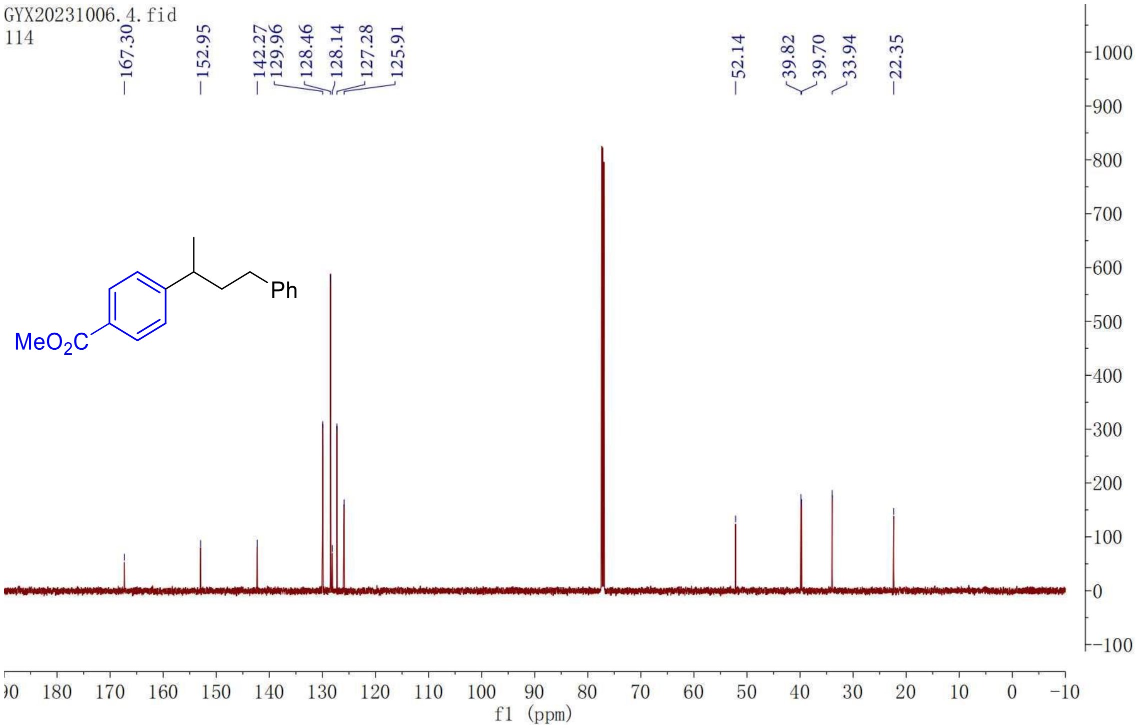

46, ^{1}H-NMR (600 MHZ, CDCl$_3$)

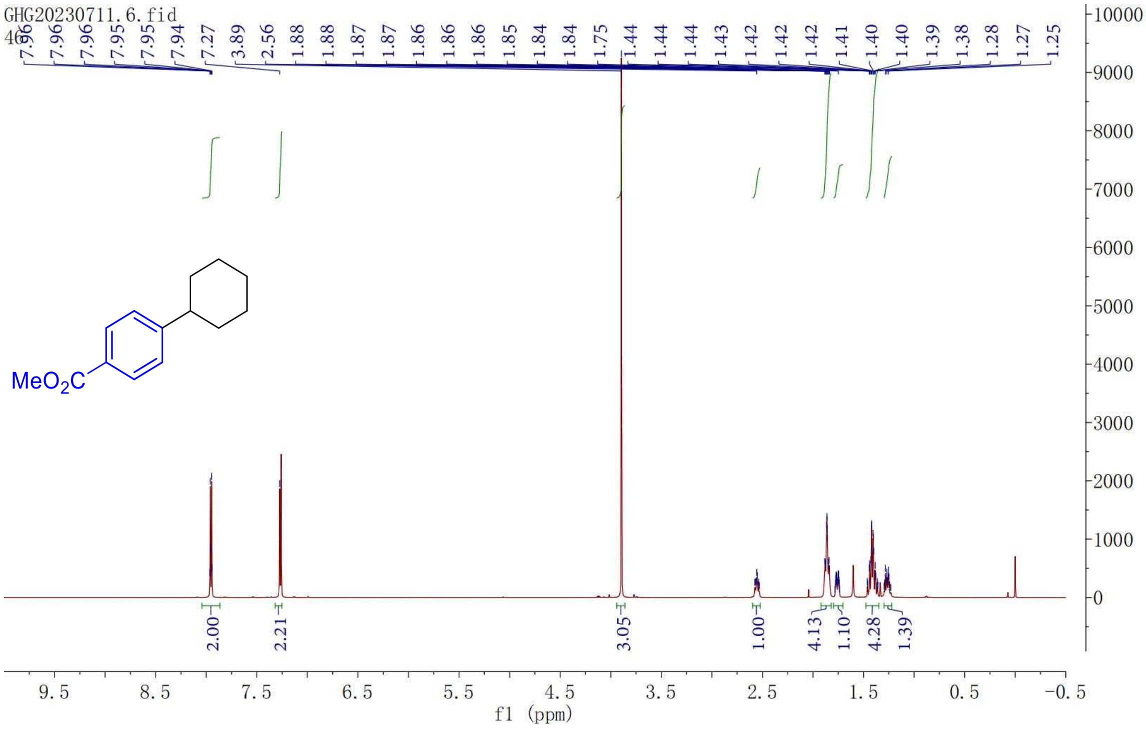

46, ^{13}C NMR (151 MHz, $CDCl_3$)

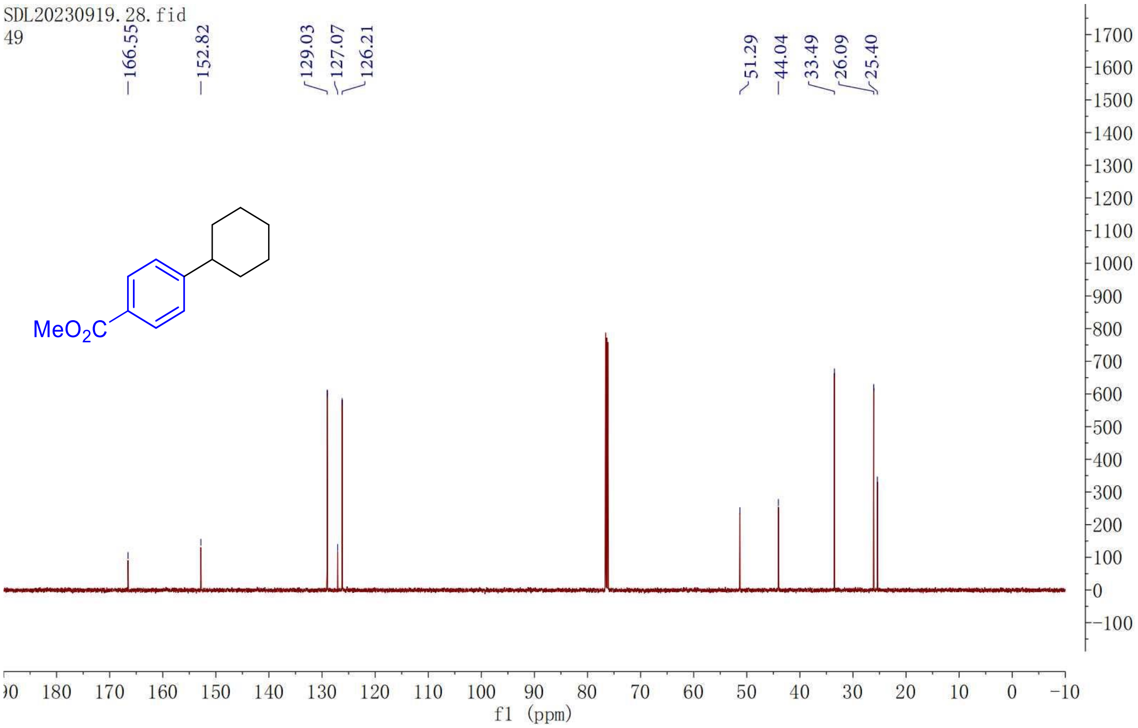

47, ^{1}H-NMR (600 MHZ, $CDCl_3$)

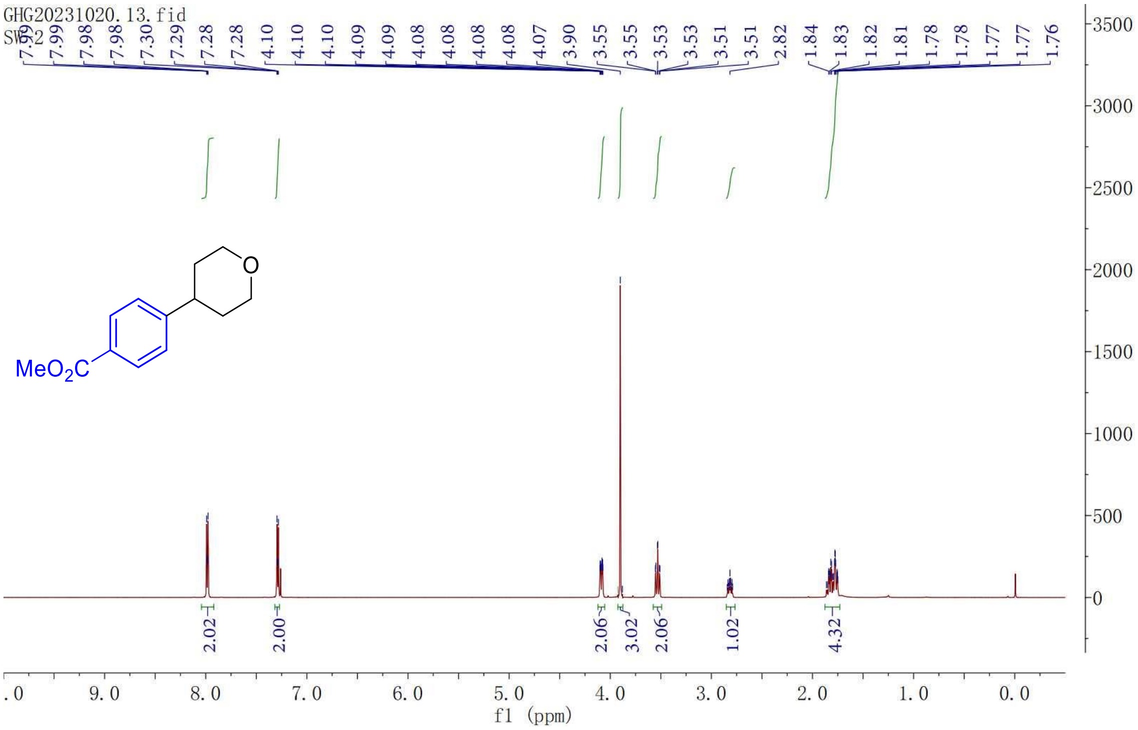

47, ^{13}C NMR (151 MHz, CDCl$_3$)

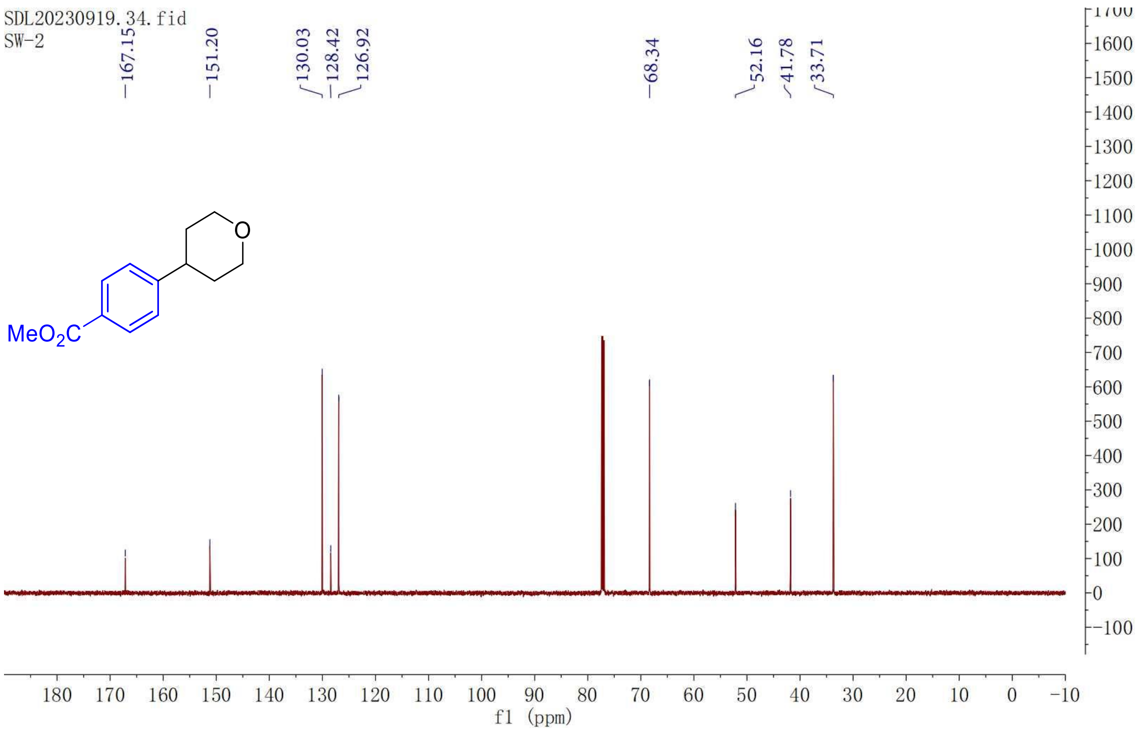

48, ^{1}H-NMR (600 MHZ, CDCl$_3$)

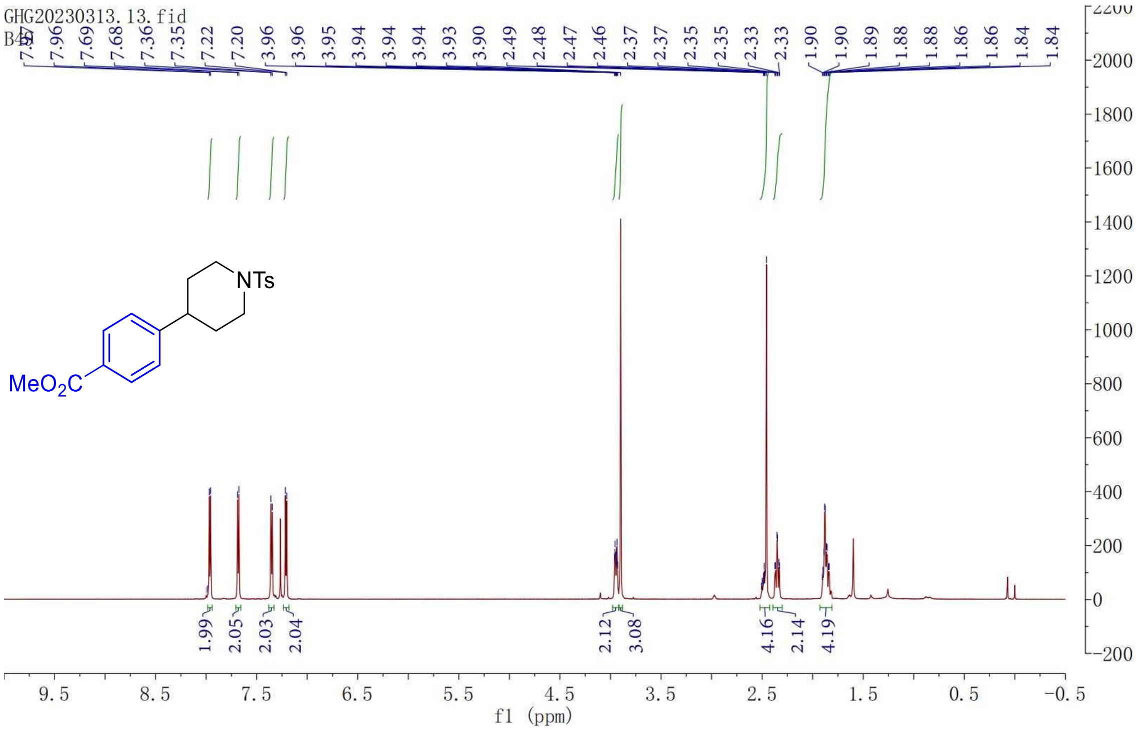

48, ^{13}C NMR (151 MHz, CDCl$_3$)

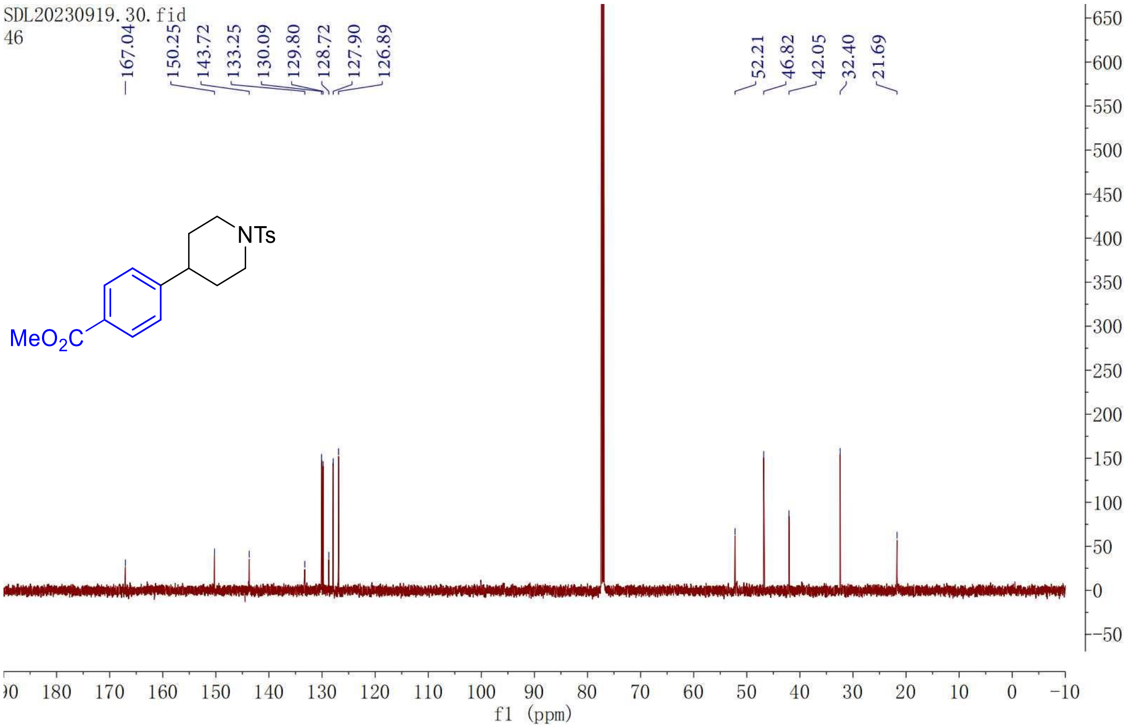

49, ^{1}H-NMR (600 MHZ, CDCl$_3$)

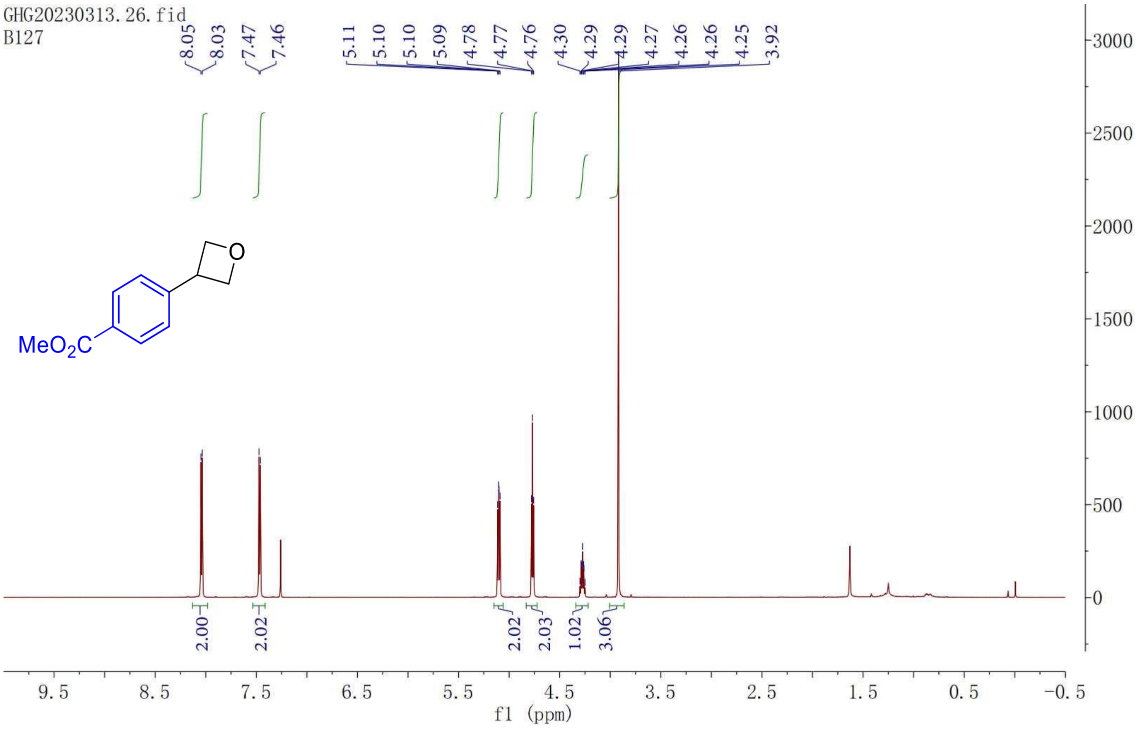

49, ^{13}C NMR (151 MHz, CDCl$_3$)

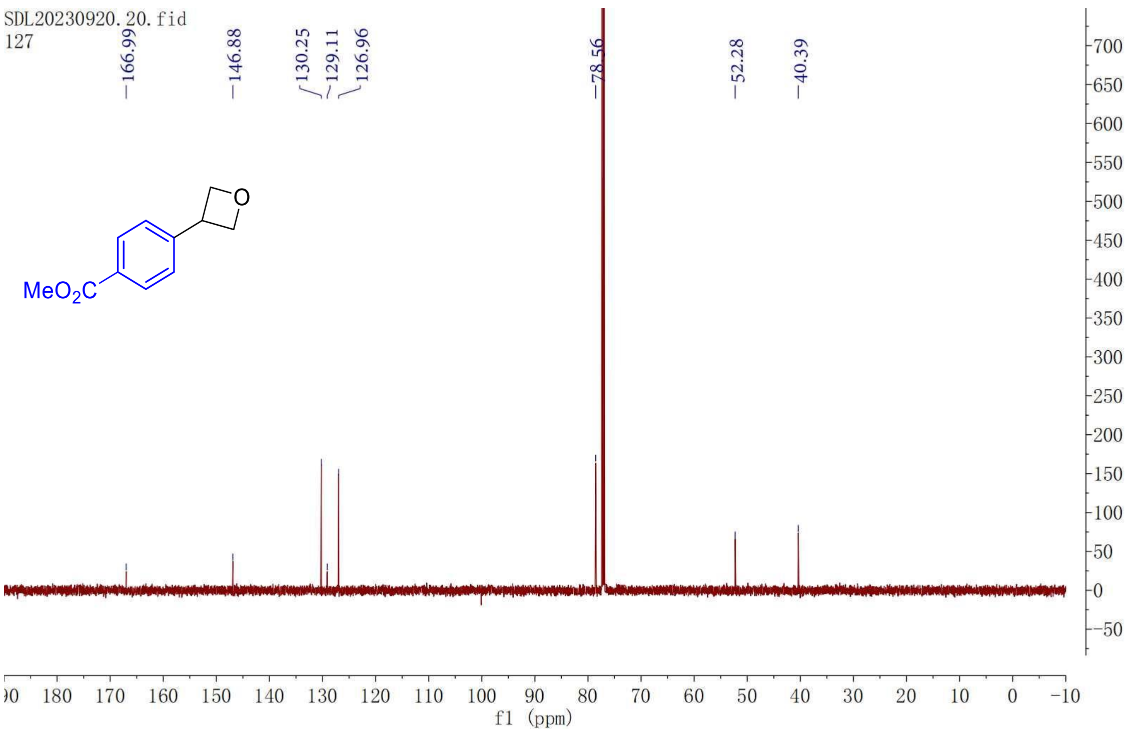

50, ^{1}H-NMR (600 MHZ, CDCl$_3$)

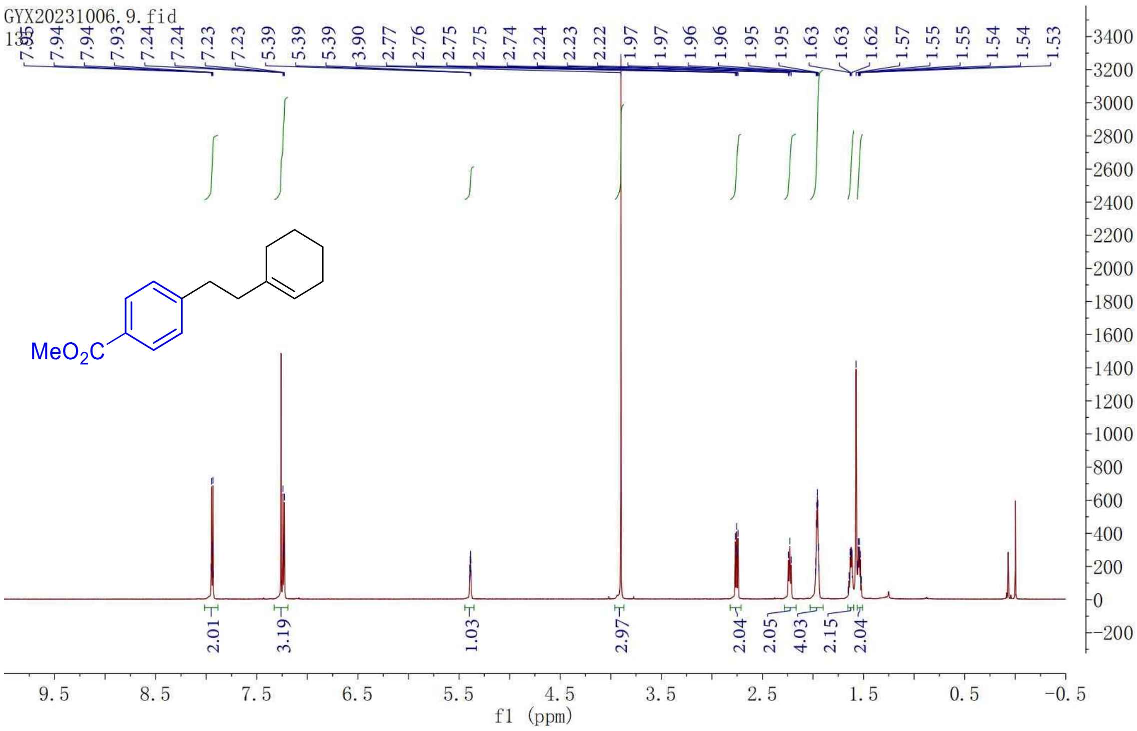

50, ^{13}C NMR (151 MHz, CDCl$_3$)

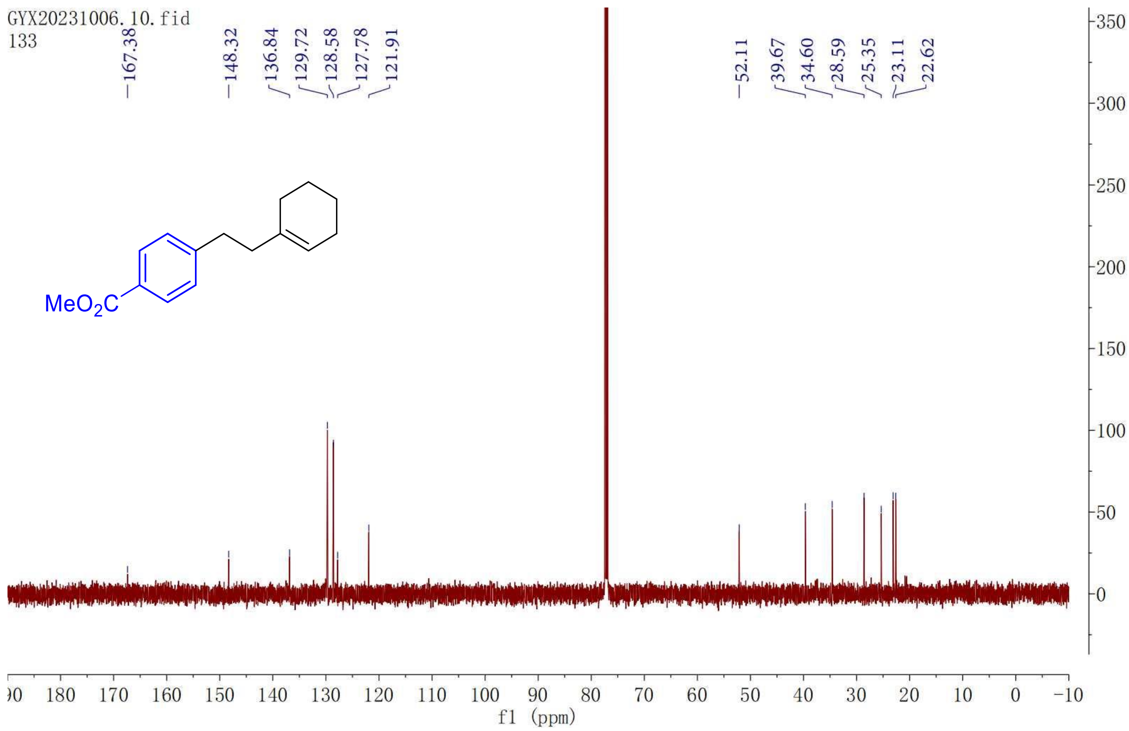

51, ^{1}H-NMR (600 MHZ, CDCl$_3$)

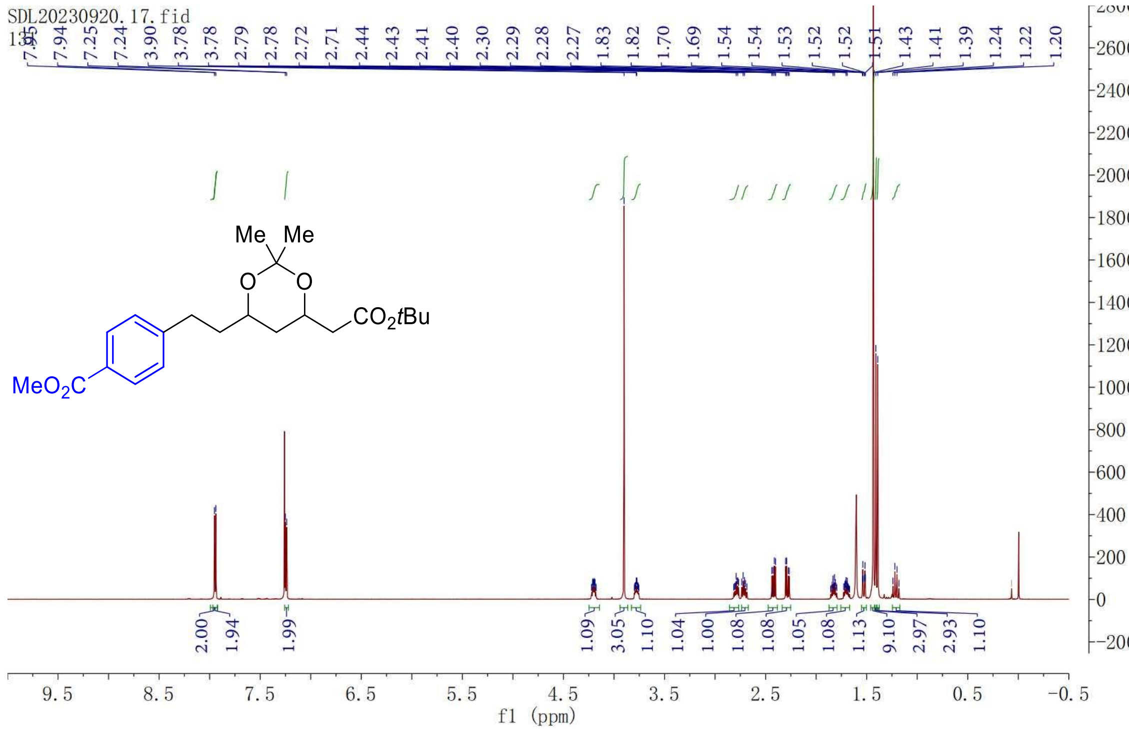

51, ^{13}C NMR (151 MHz, CDCl$_3$)

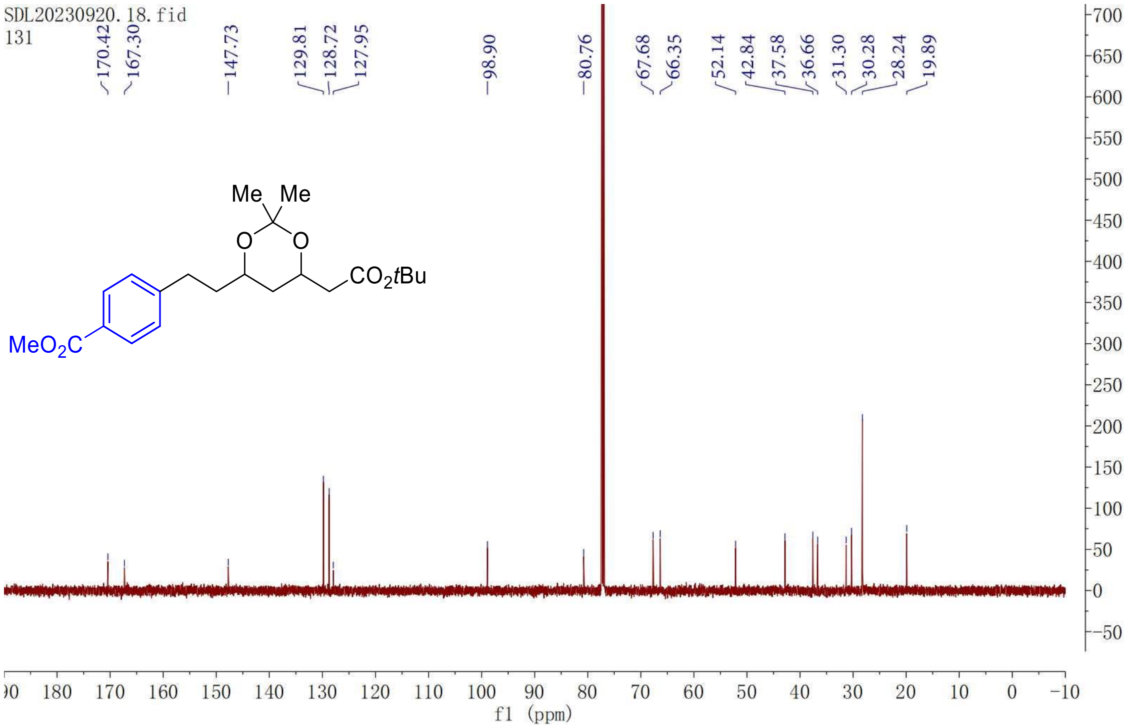

52, ^{1}H-NMR (600 MHZ, CDCl$_3$)

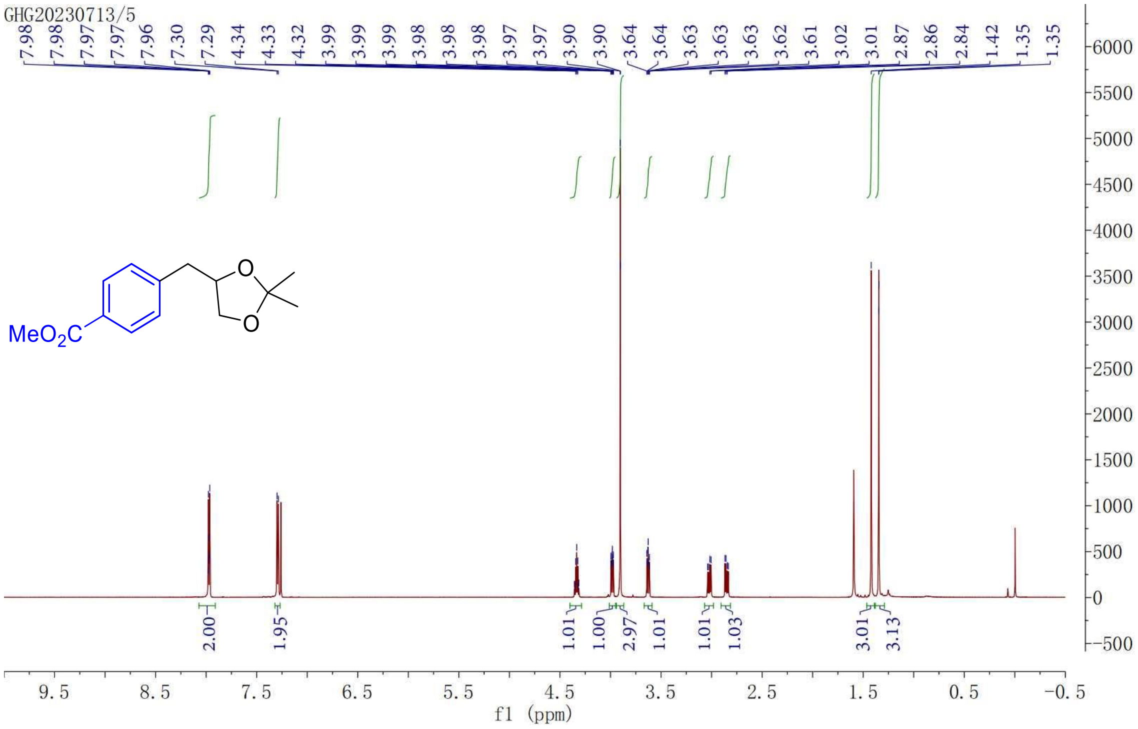

52, ^{13}C NMR (151 MHz, CDCl$_3$)

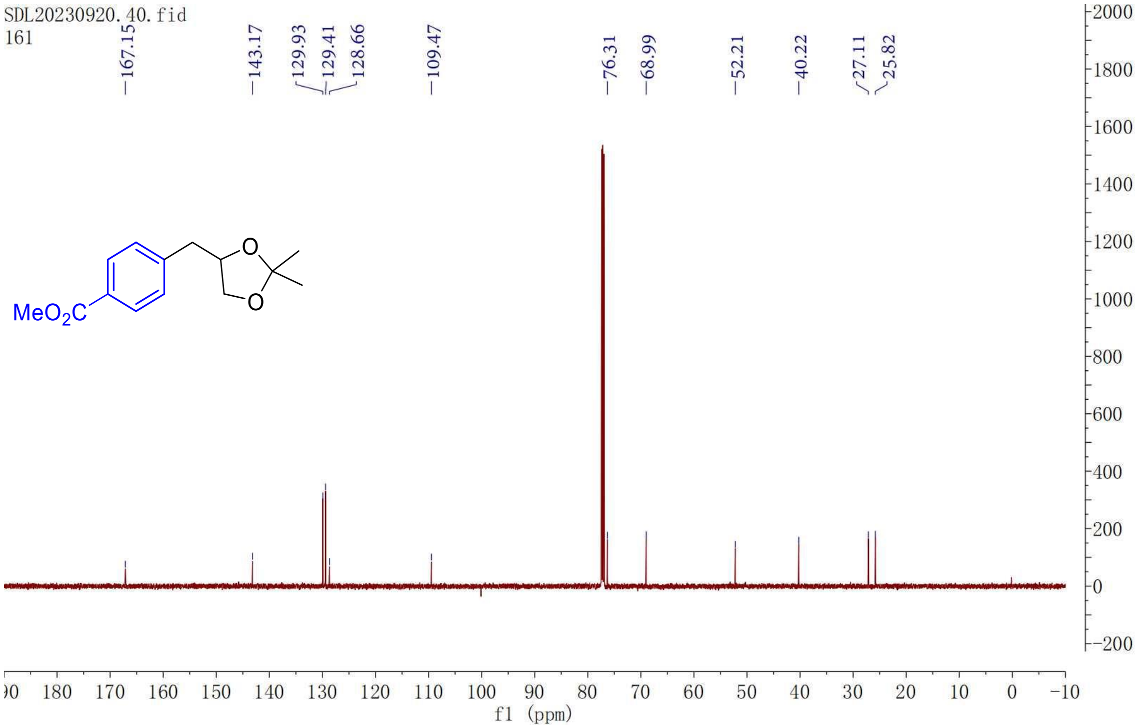

53, ^{1}H-NMR (600 MHZ, CDCl$_3$)

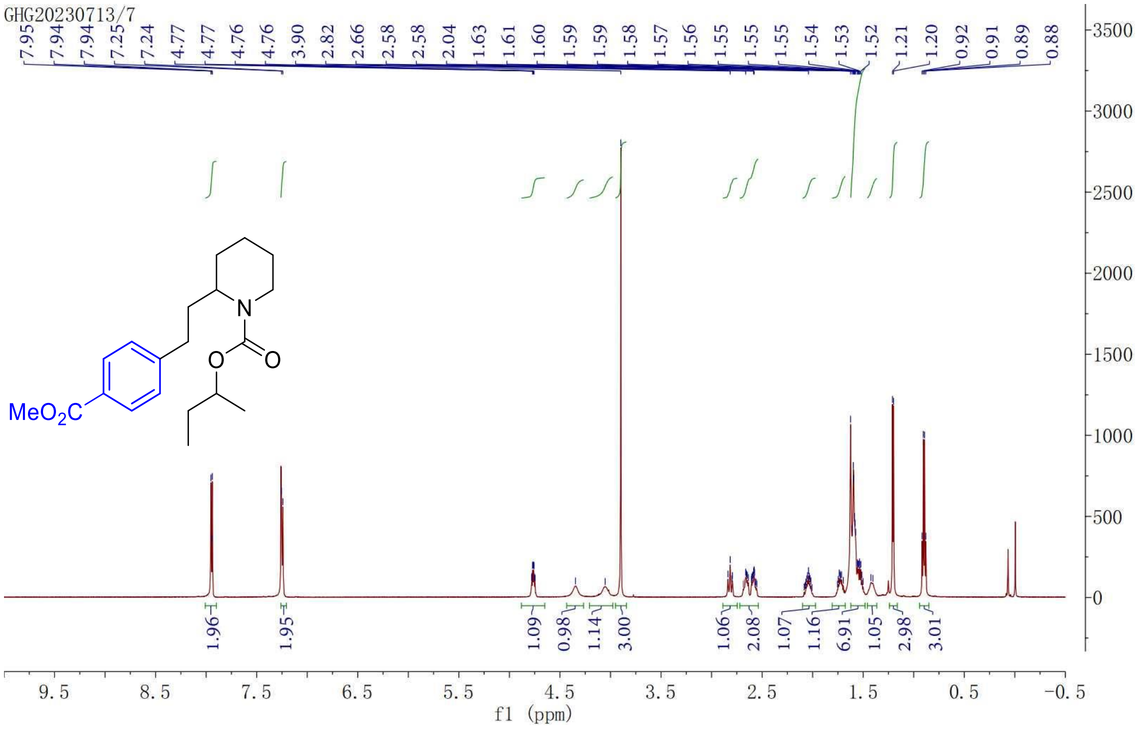

53, ^{13}C NMR (151 MHz, CDCl$_3$)

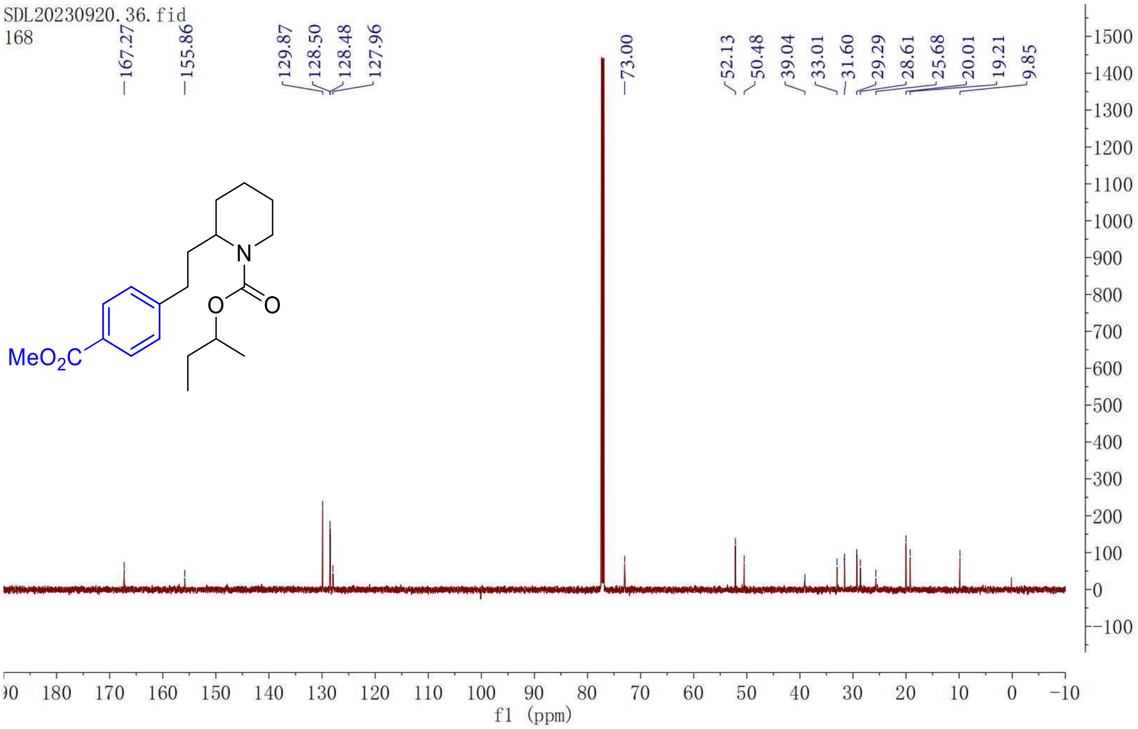

54, ^{1}H-NMR (600 MHZ, CDCl$_3$)

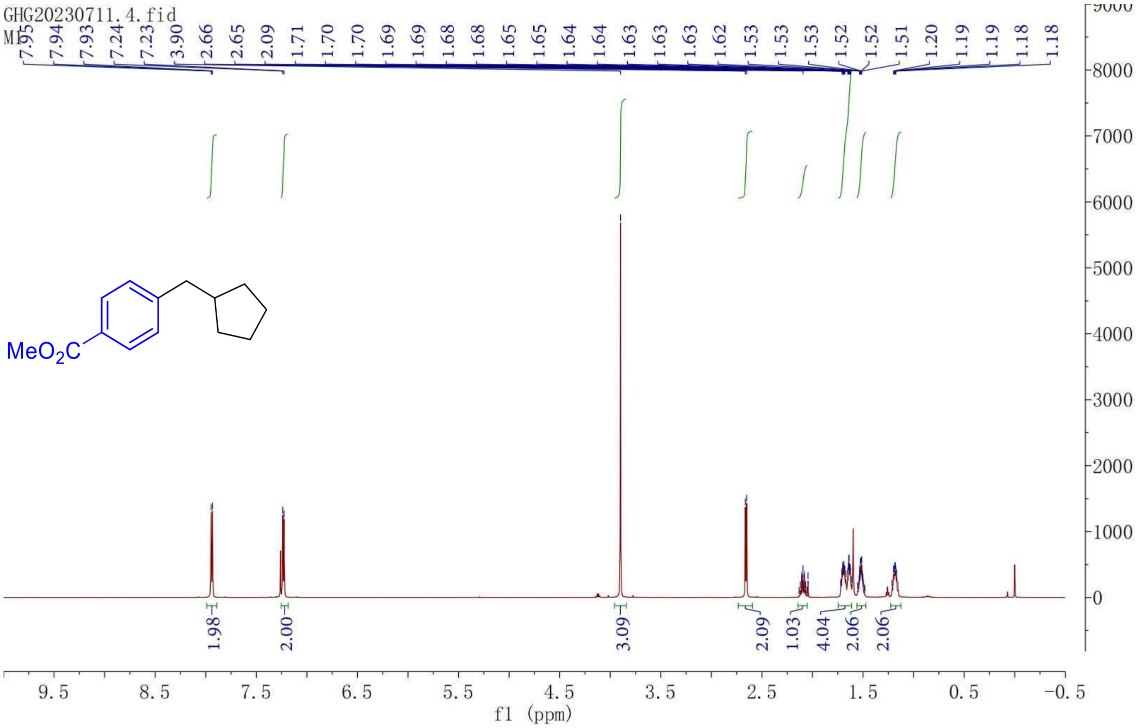

54, ^{13}C NMR (151 MHz, $CDCl_3$)

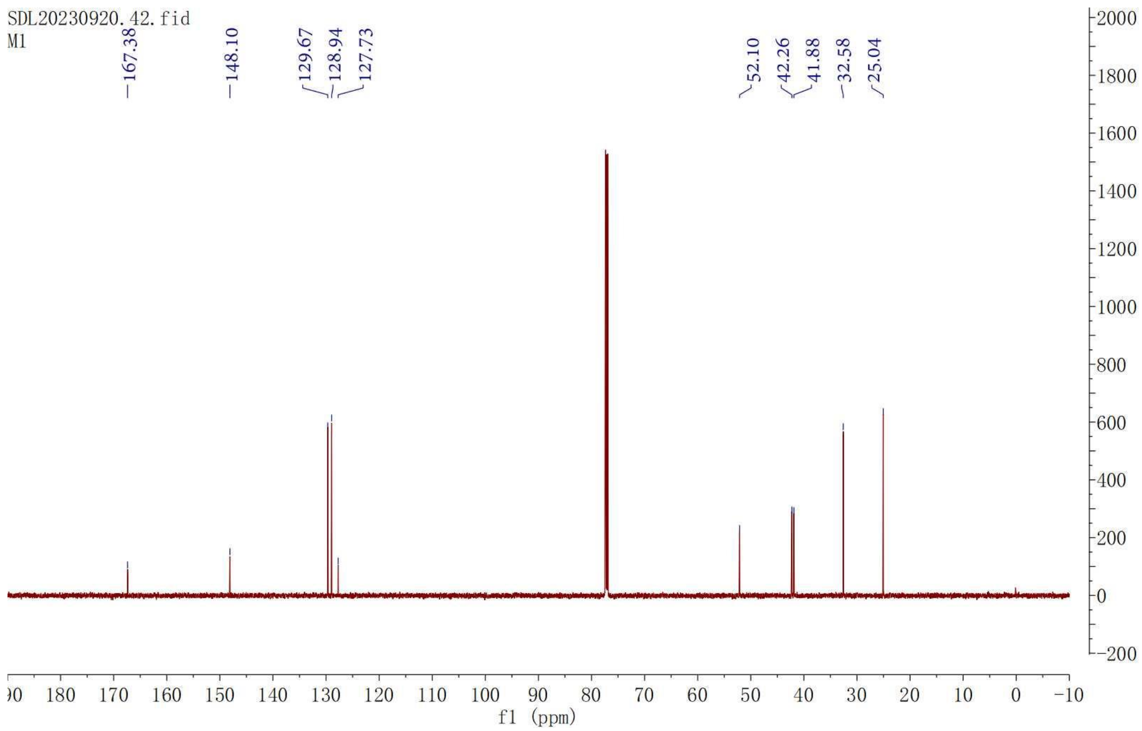

55, ^{1}H-NMR (600 MHZ, $CDCl_3$)

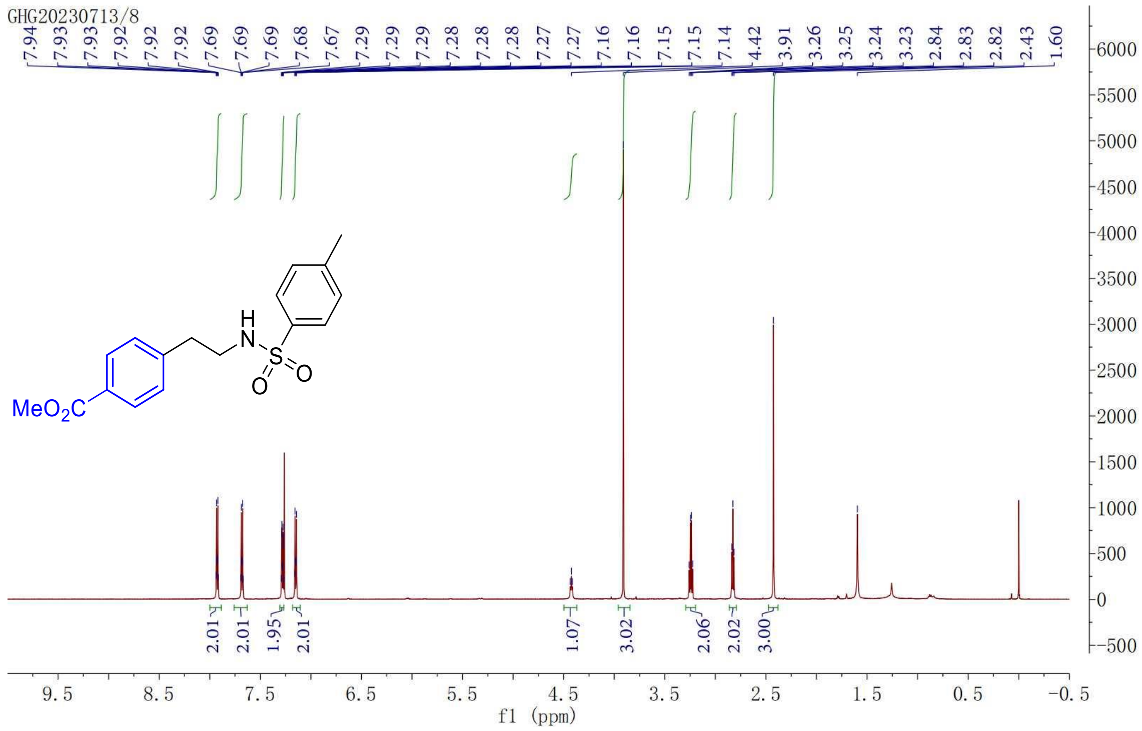

56, ^{1}H-NMR (600 MHZ, $CDCl_3$)

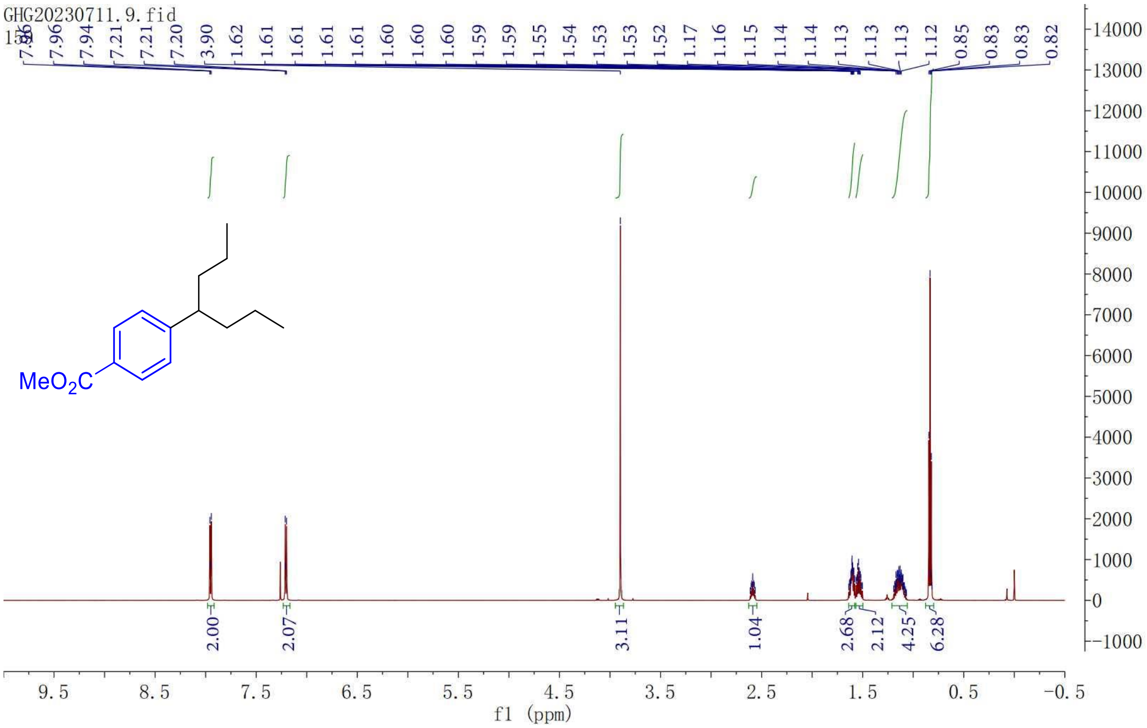

56, ^{13}C NMR (151 MHz, $CDCl_3$)

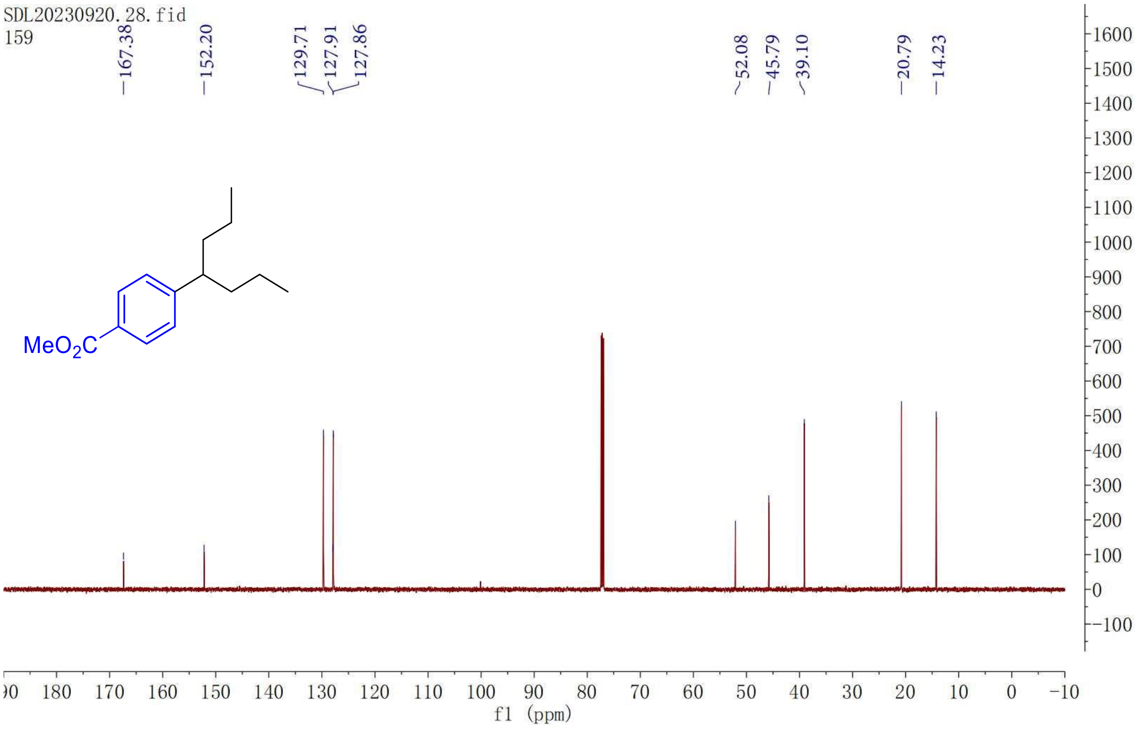

57, ^{1}H-NMR (600 MHZ, CDCl$_3$)

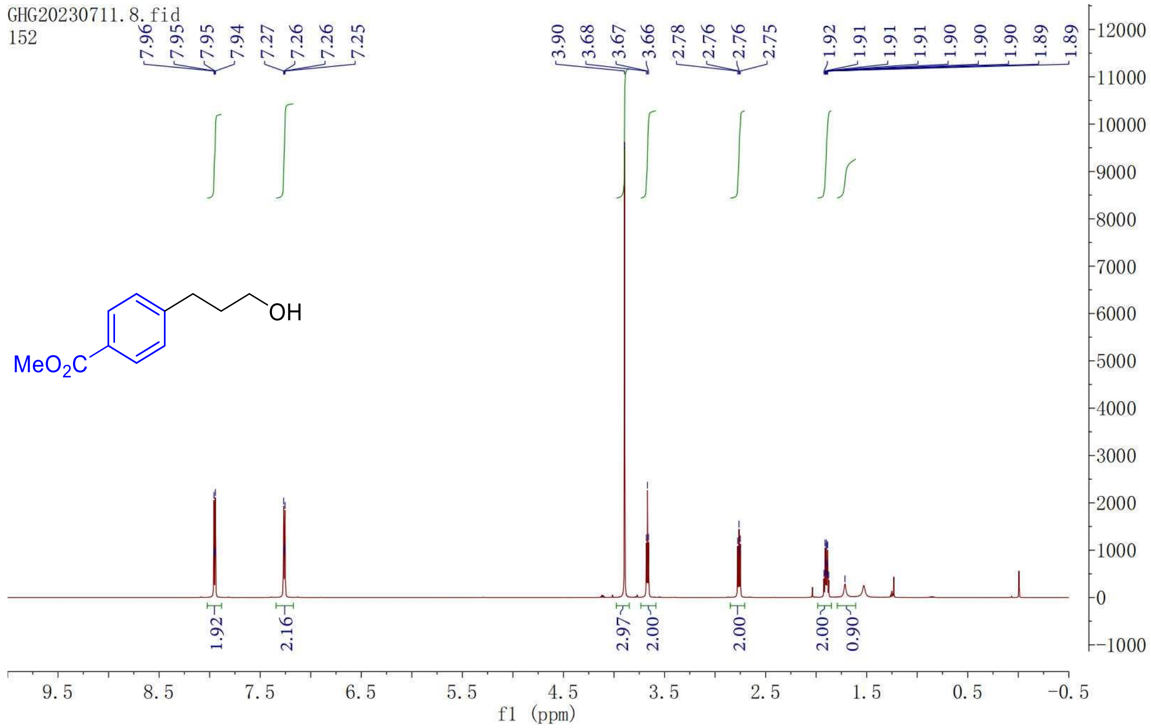

57, ^{13}C NMR (151 MHz, CDCl$_3$)

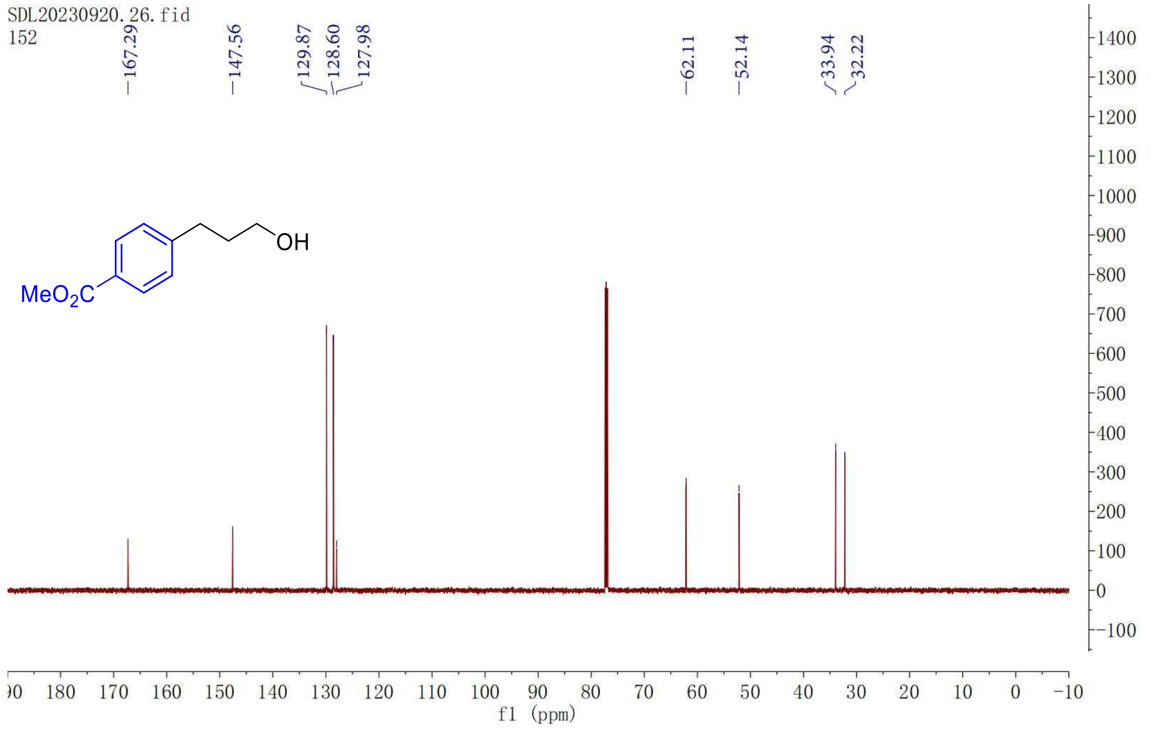

58, ^{1}H-NMR (600 MHZ, $CDCl_3$)

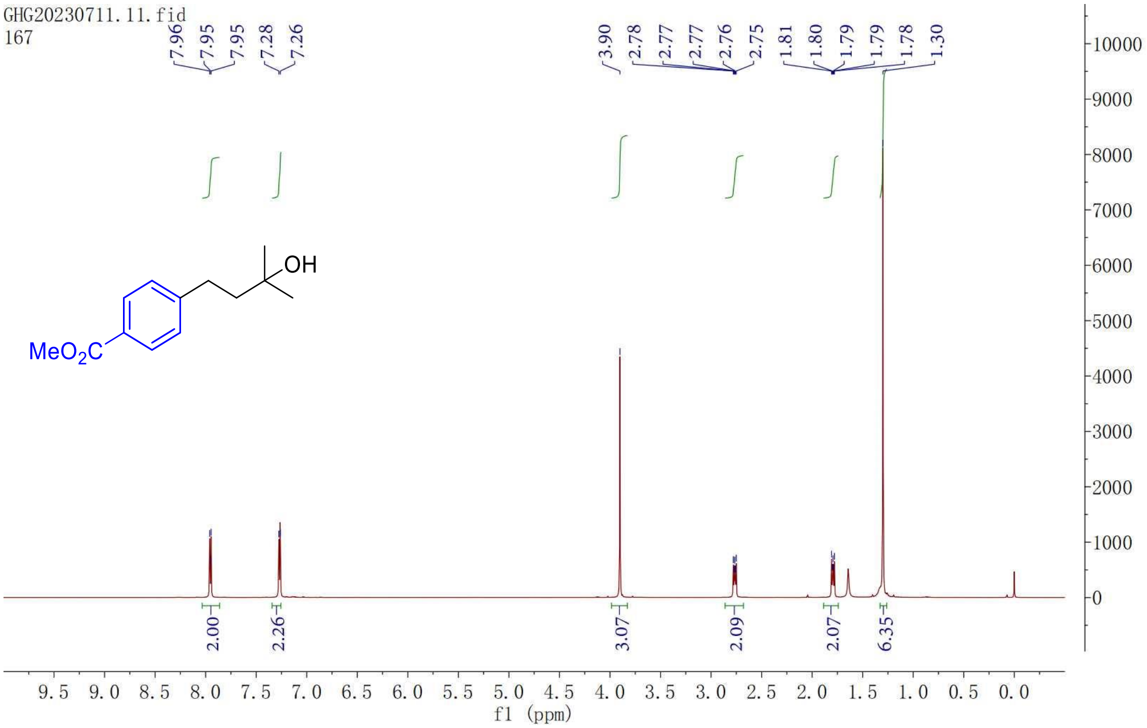

58, ^{13}C NMR (151 MHz, $CDCl_3$)

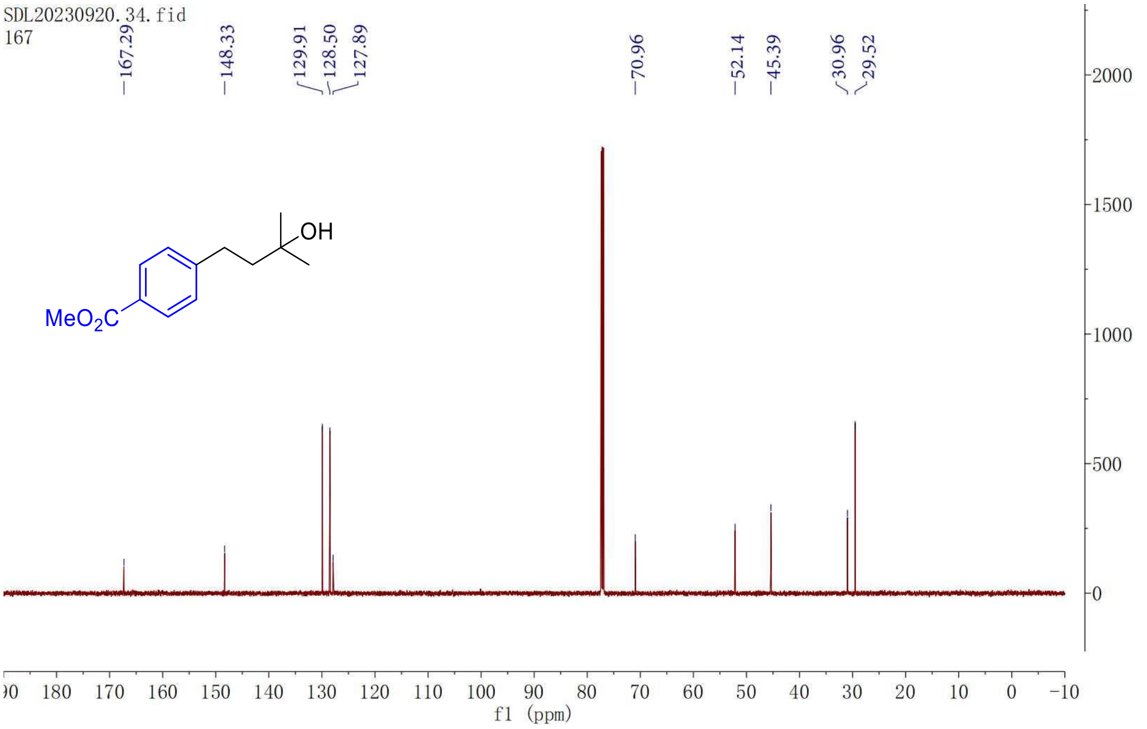

59, ^{1}H-NMR (600 MHZ, $CDCl_3$)

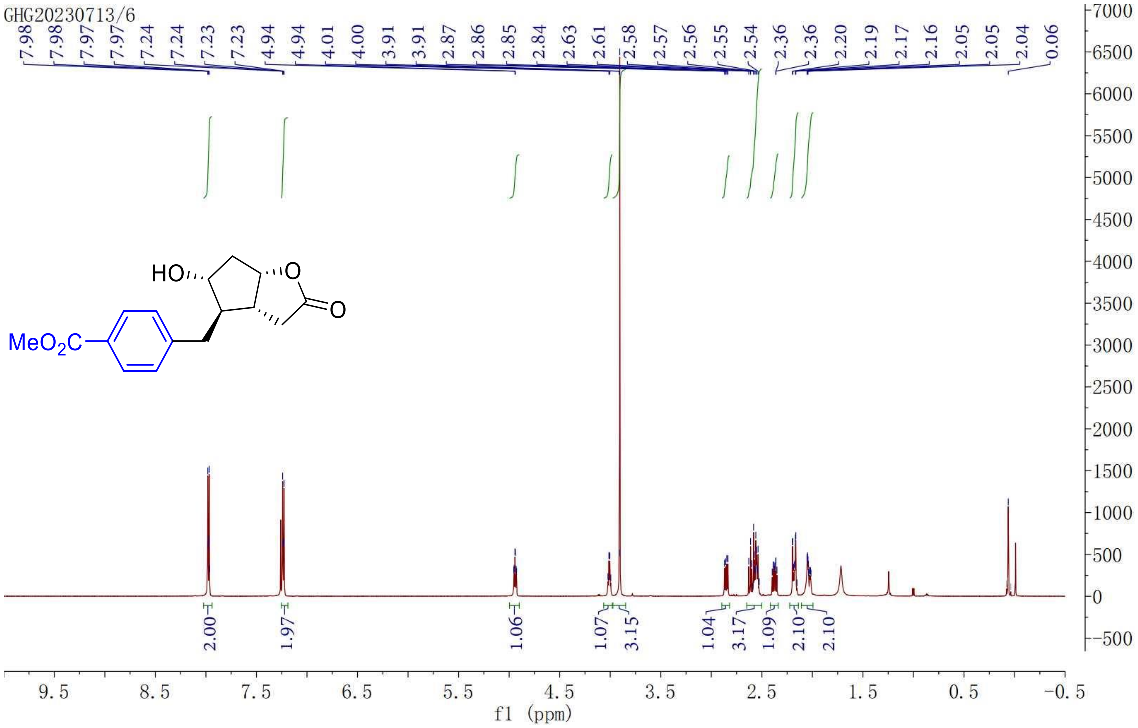

59, ^{13}C NMR (151 MHz, $CDCl_3$)

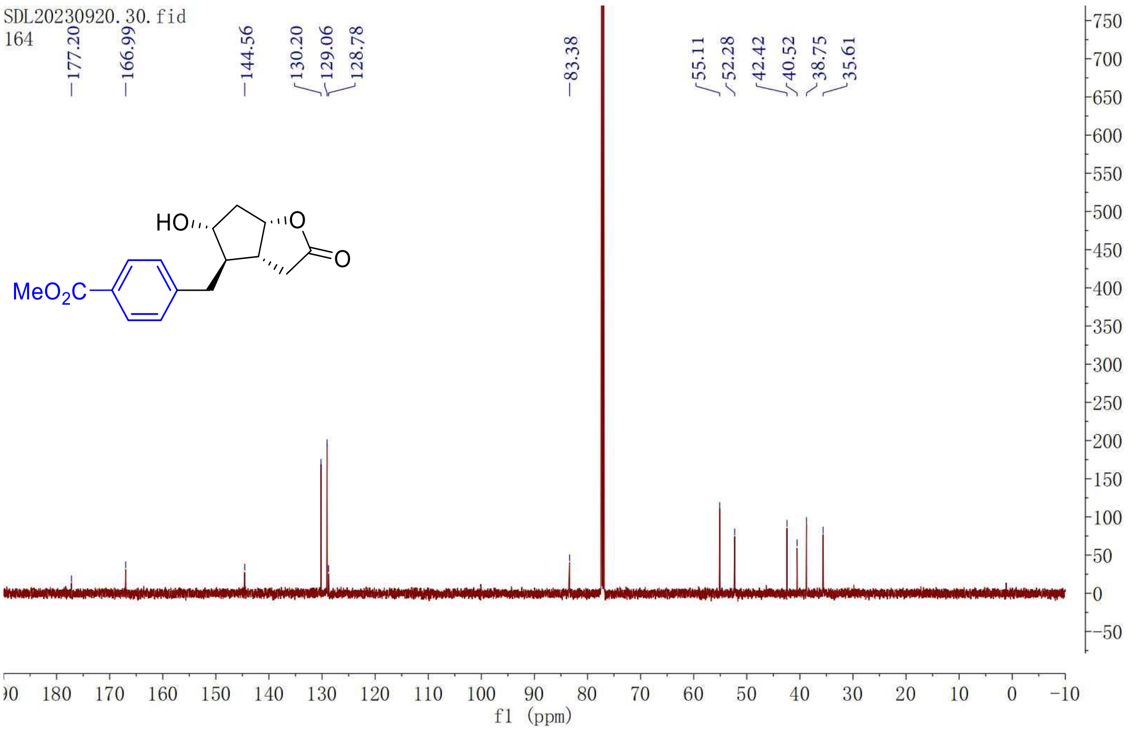

60, ¹H-NMR (600 MHZ, CDCl₃)

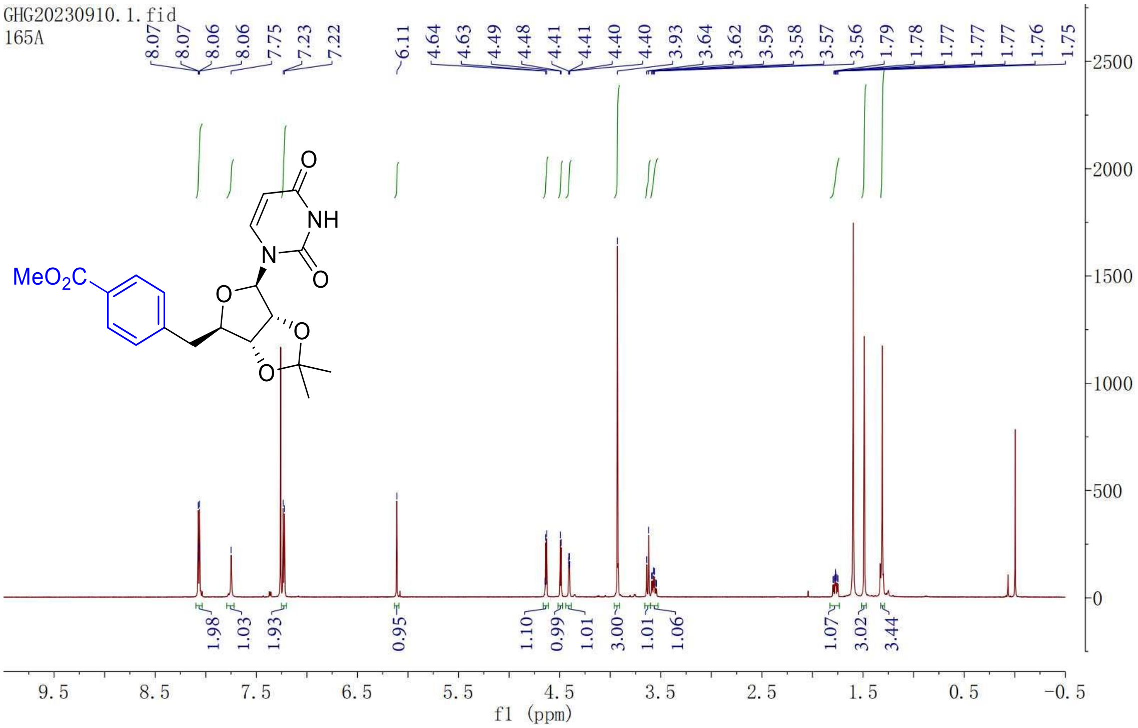

60, ^{13}C NMR (151 MHz, CDCl$_3$)

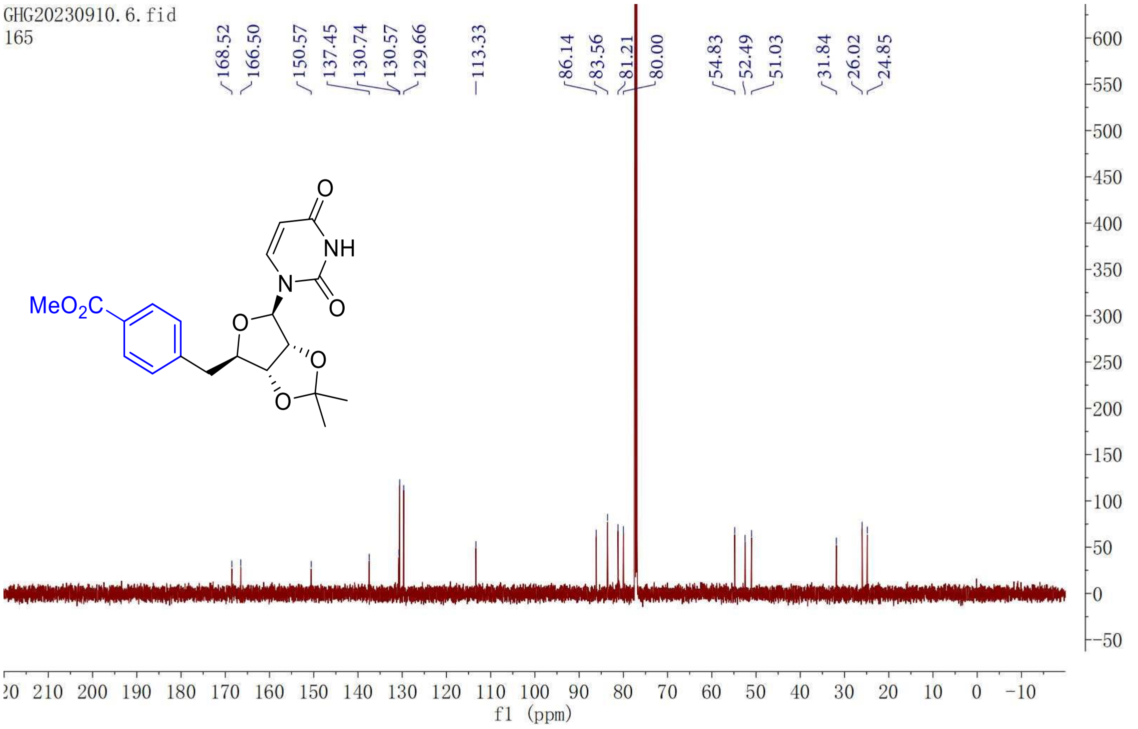

61, ¹H-NMR (600 MHZ, $CDCl_3$)

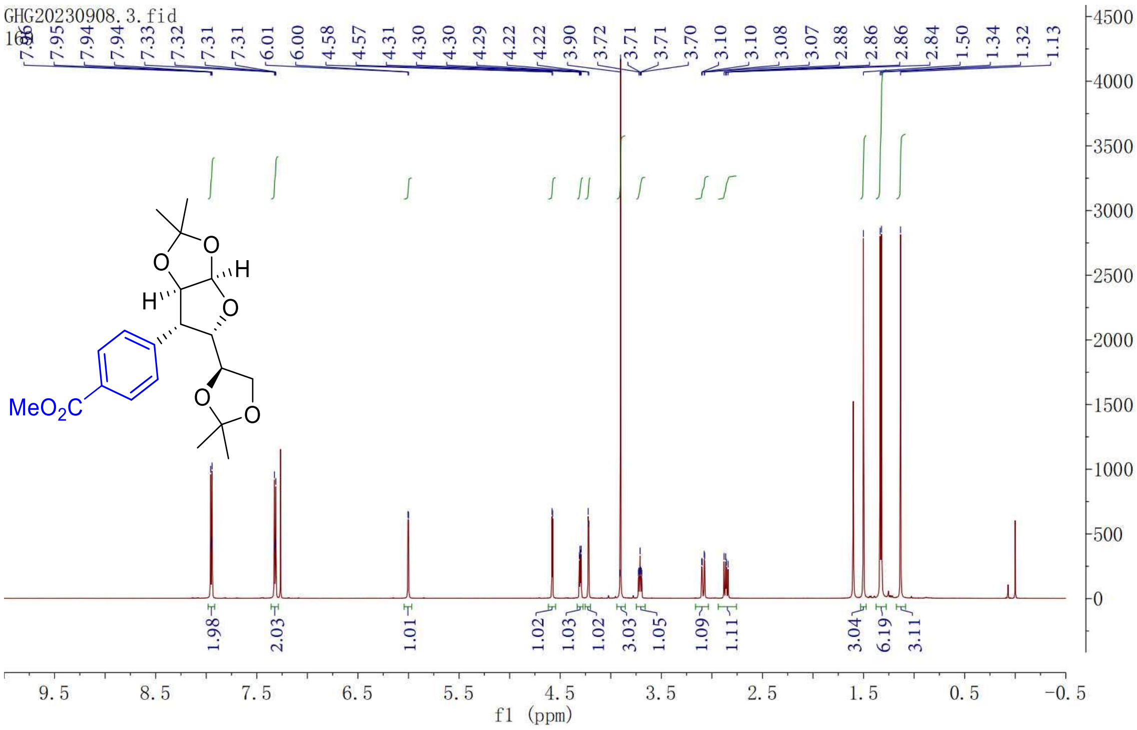

61, ¹³C NMR (151 MHz, $CDCl_3$)

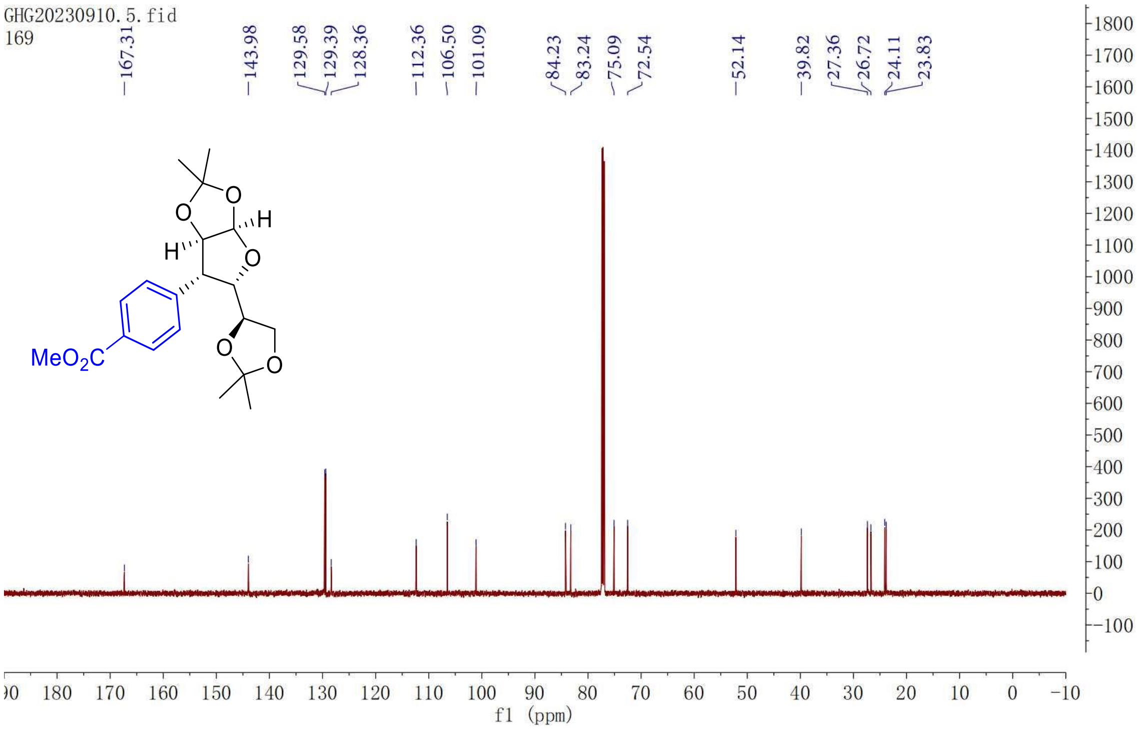

1c, **^{1}H-NMR (600 MHZ, CDCl$_3$)**

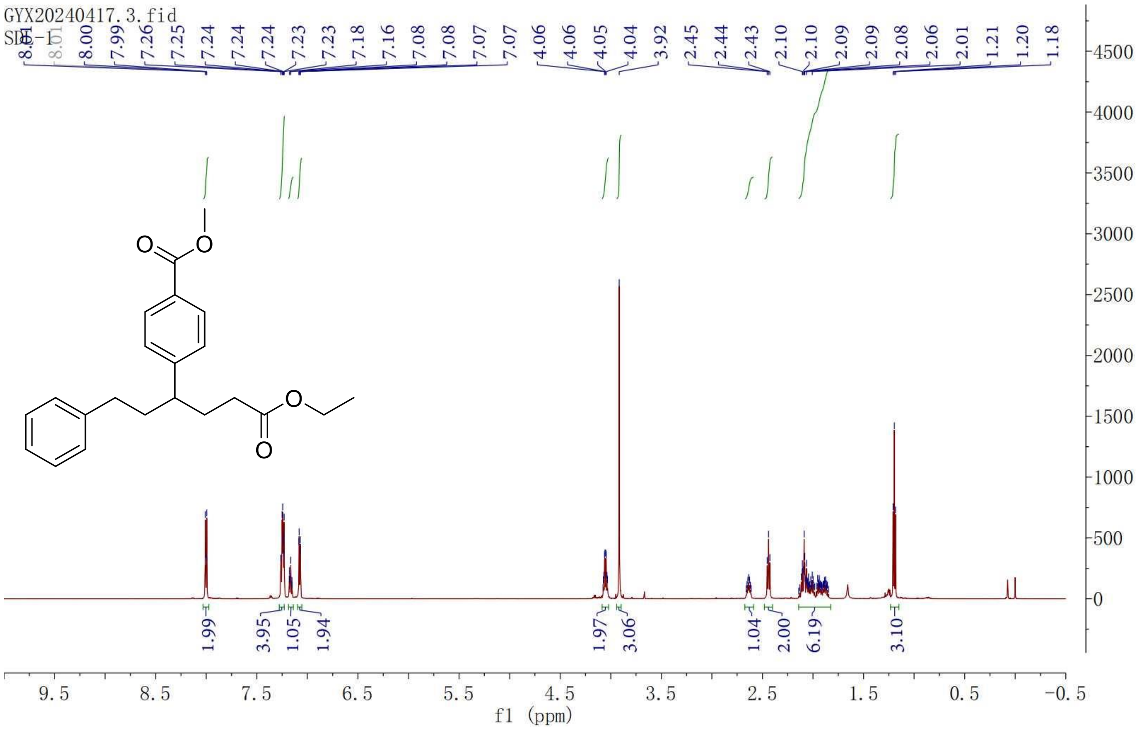

1c, **^{13}C NMR (151 MHz, CDCl$_3$)**

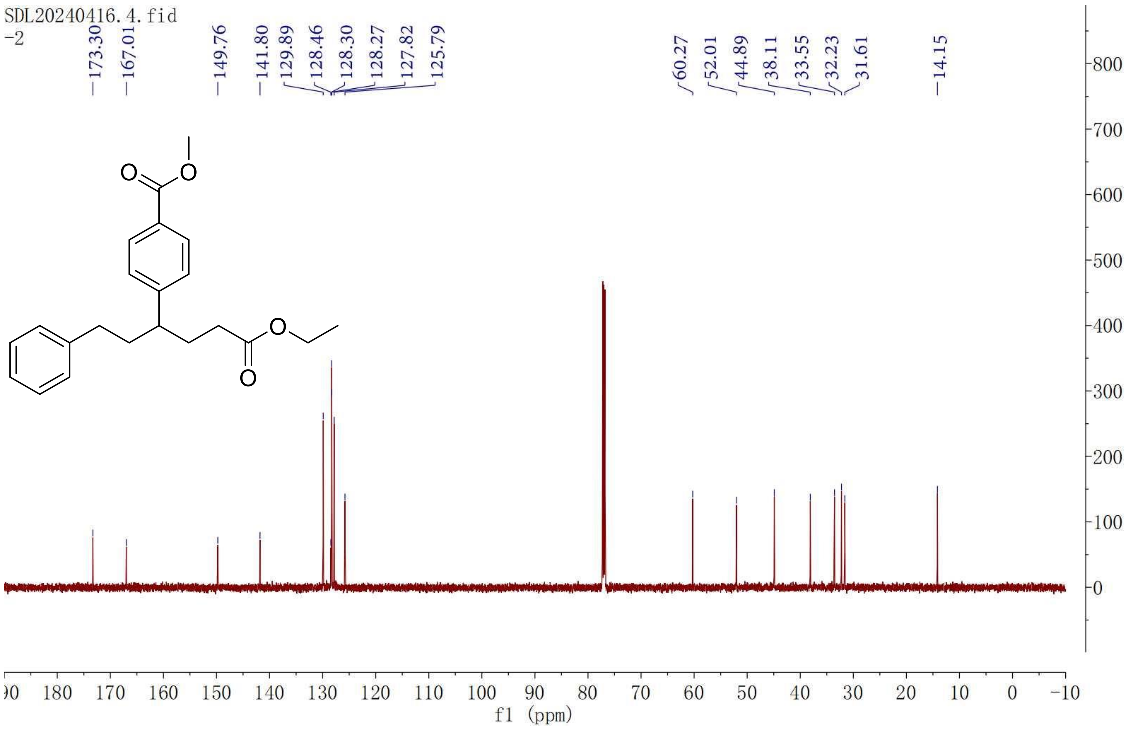

64, ¹H-NMR (600 MHZ, CDCl₃)

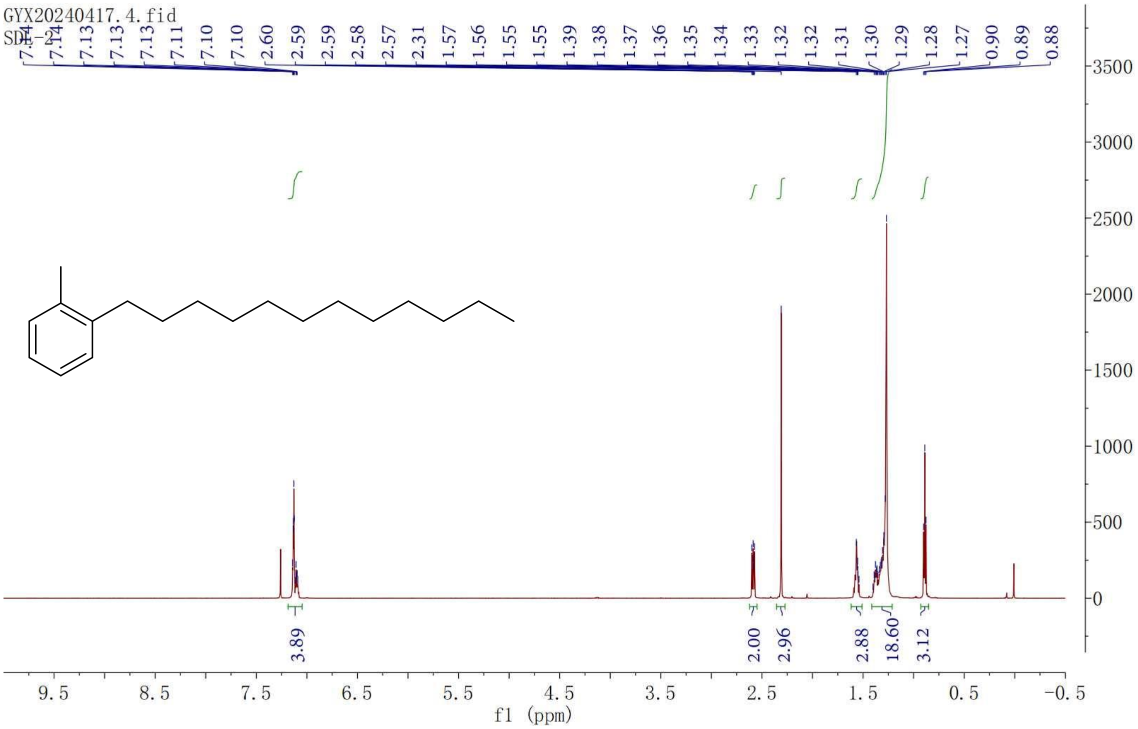

64, ¹³C NMR (151 MHz, CDCl₃)

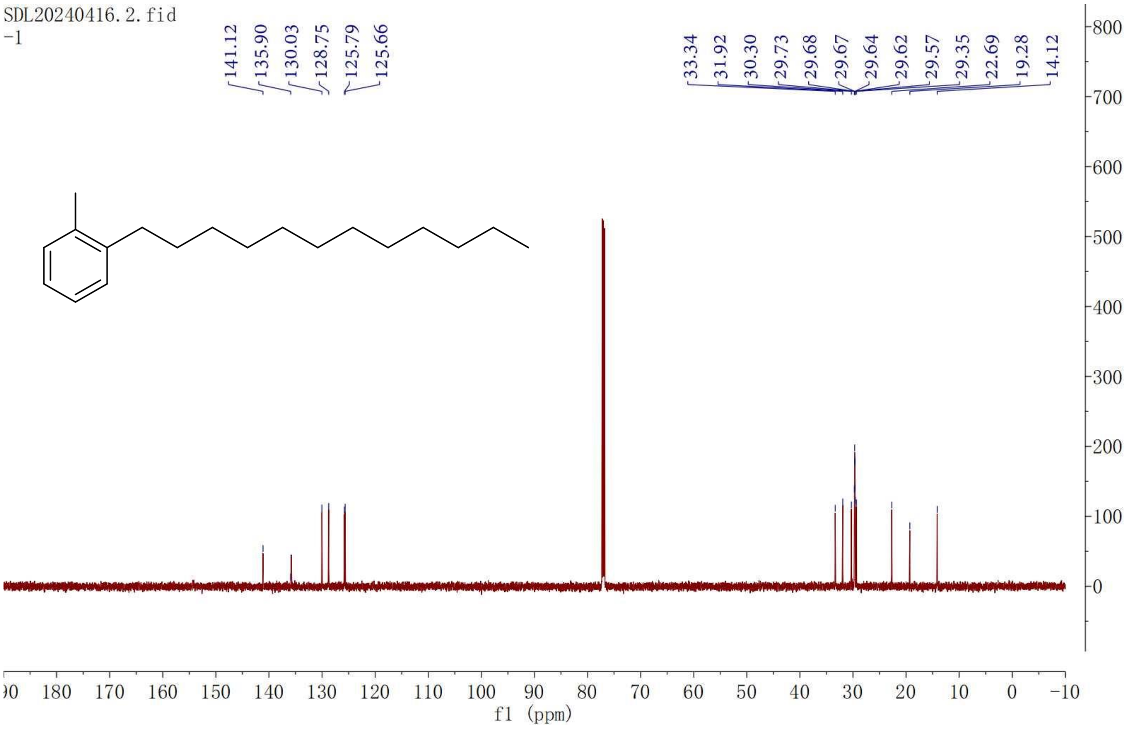

Full spectrum of Figure S1-a, ^{1}H-NMR (400 MHZ, DMA)

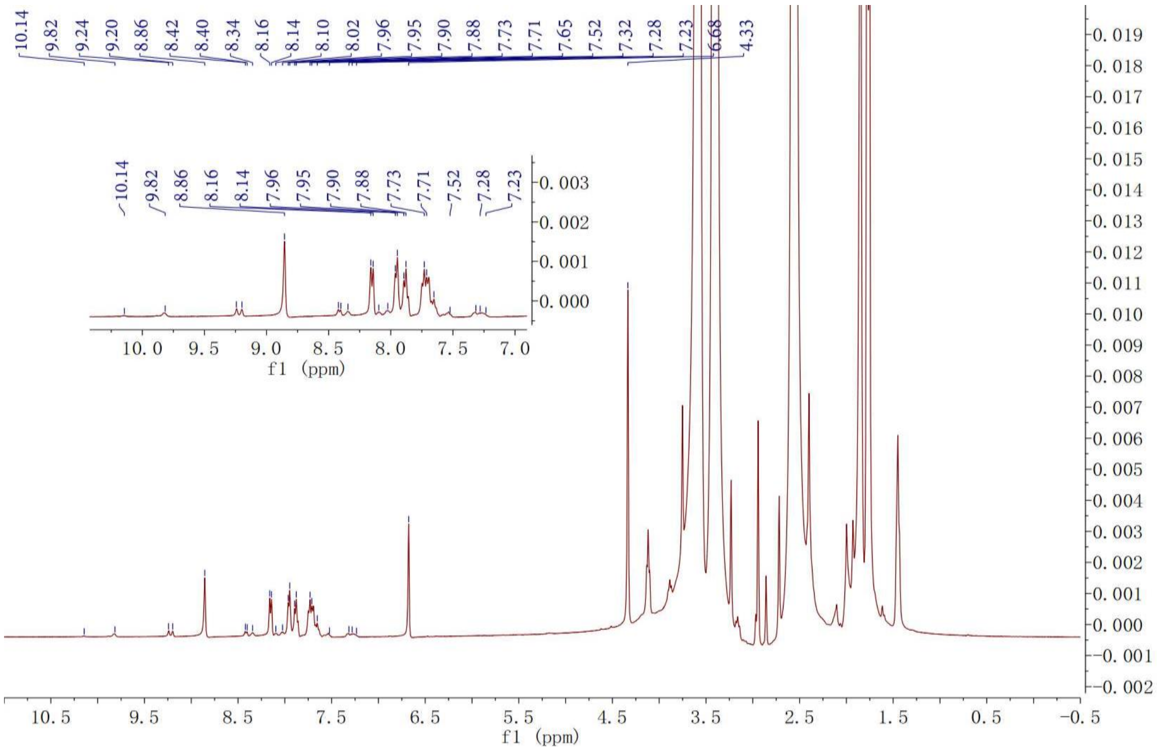

Full spectrum of Figure S1-b, ^{1}H-NMR (400 MHZ, DMA)

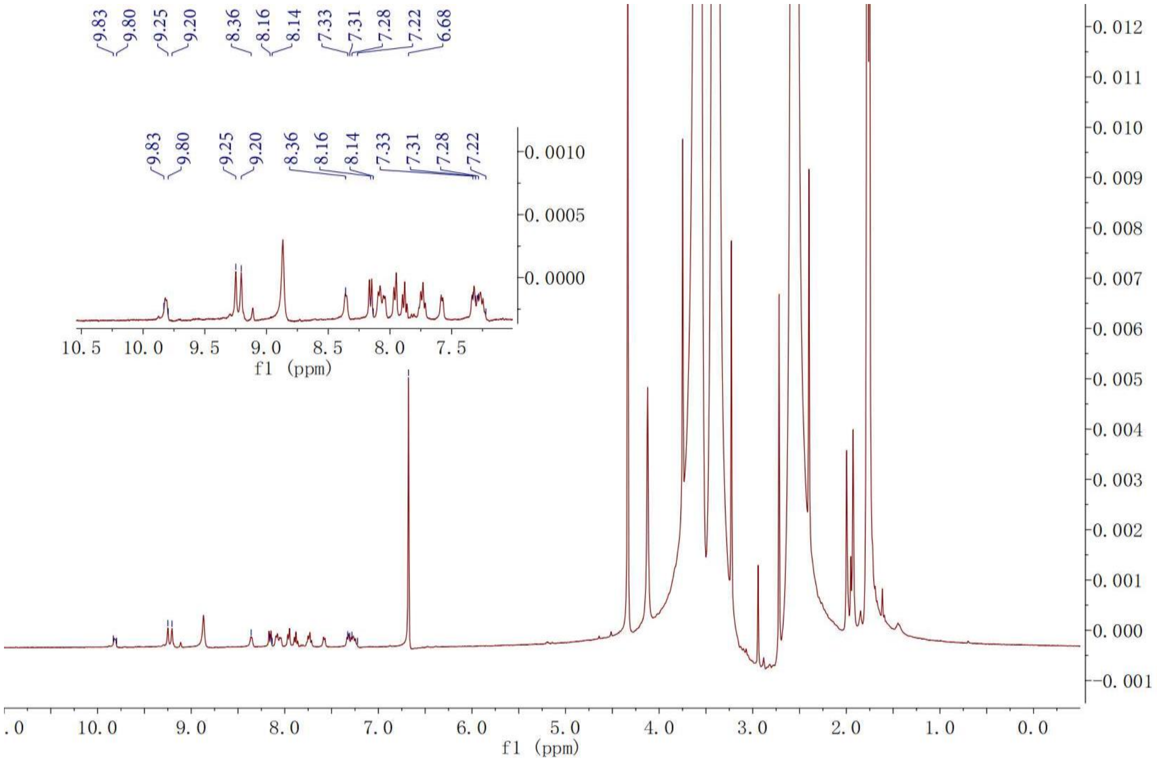

Full spectrum of Figure S1-c, ^{1}H-NMR (400 MHZ, DMA)

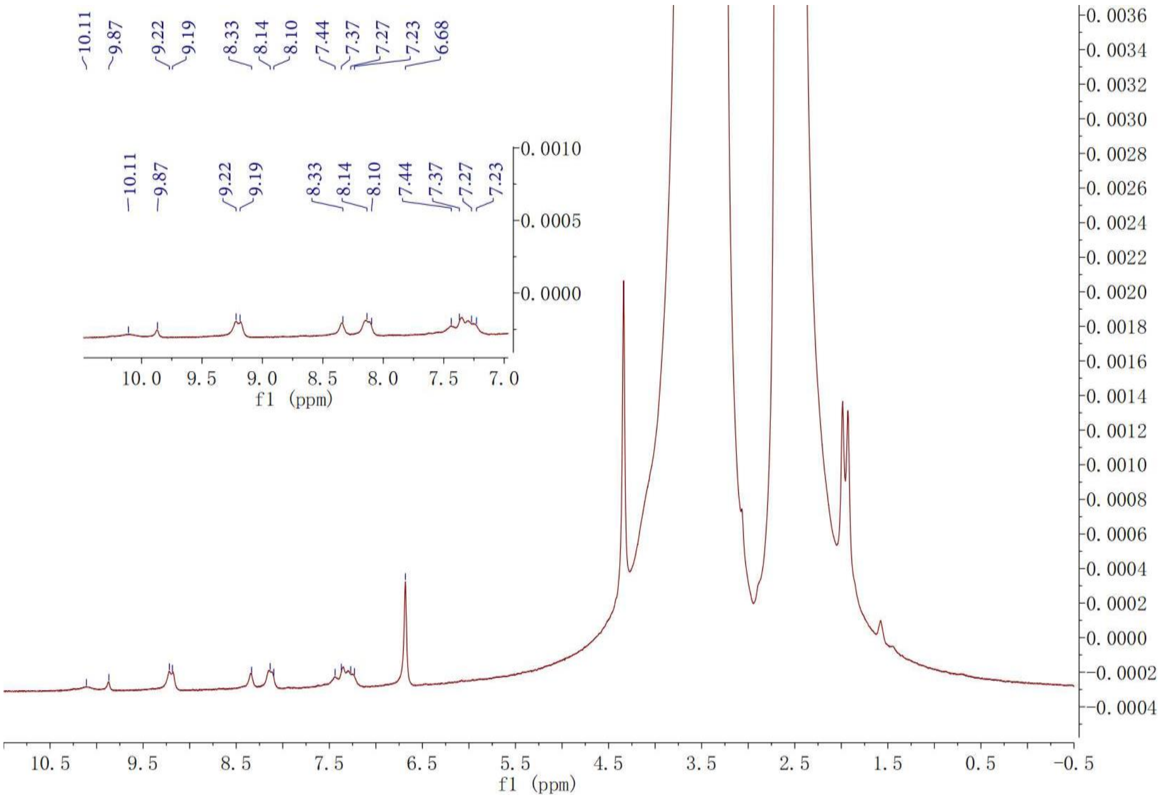

Full spectrum of 1,3,5-triazine, ^{1}H-NMR (400 MHZ, DMA)

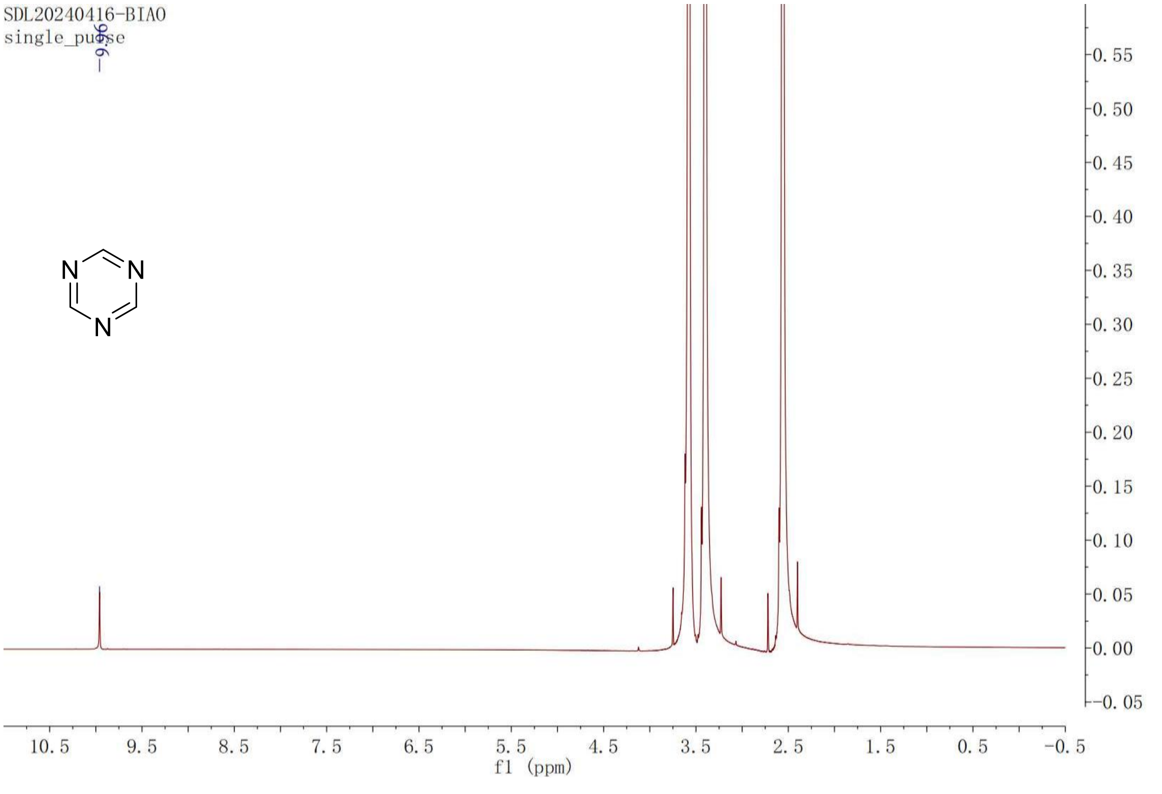

Full spectrum of Figure S3, ^{1}H-NMR (with 1,3,5-triazine 400 MHZ, DMA)

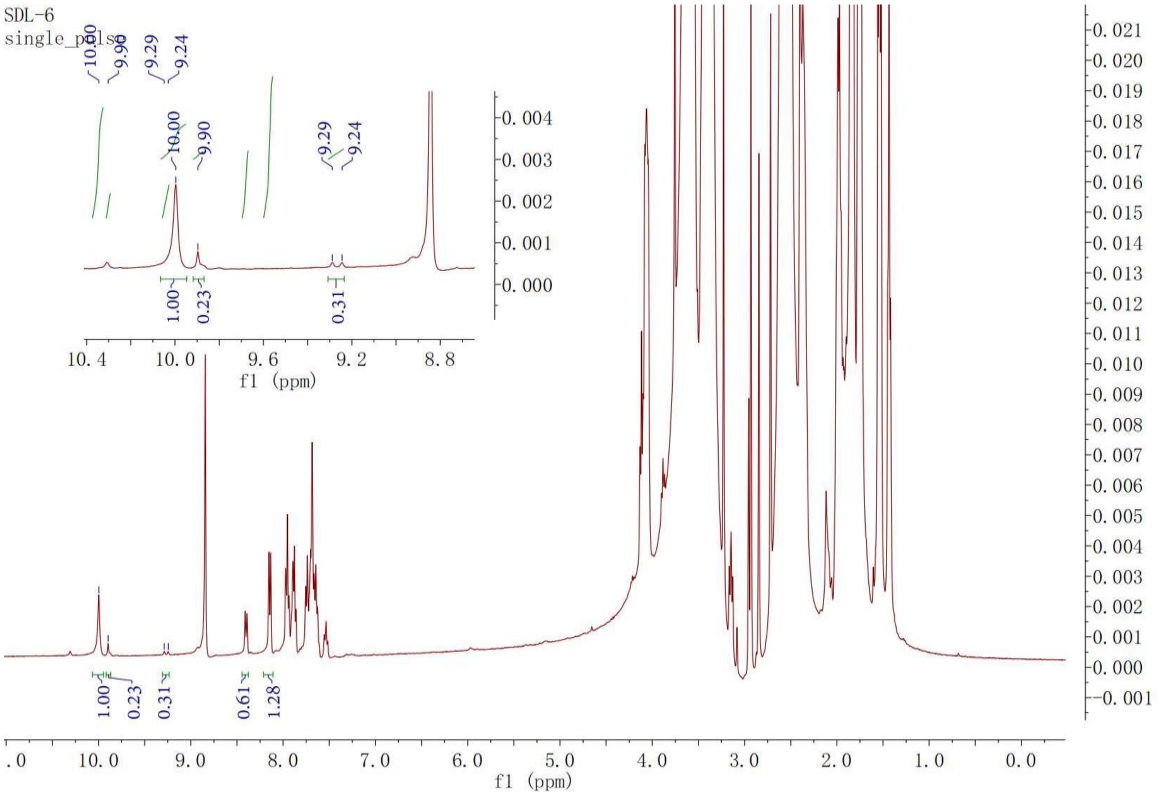

Full spectrum of Figure S4, ^{1}H-NMR (with 1,3,5-triazine 400 MHZ, DMA)

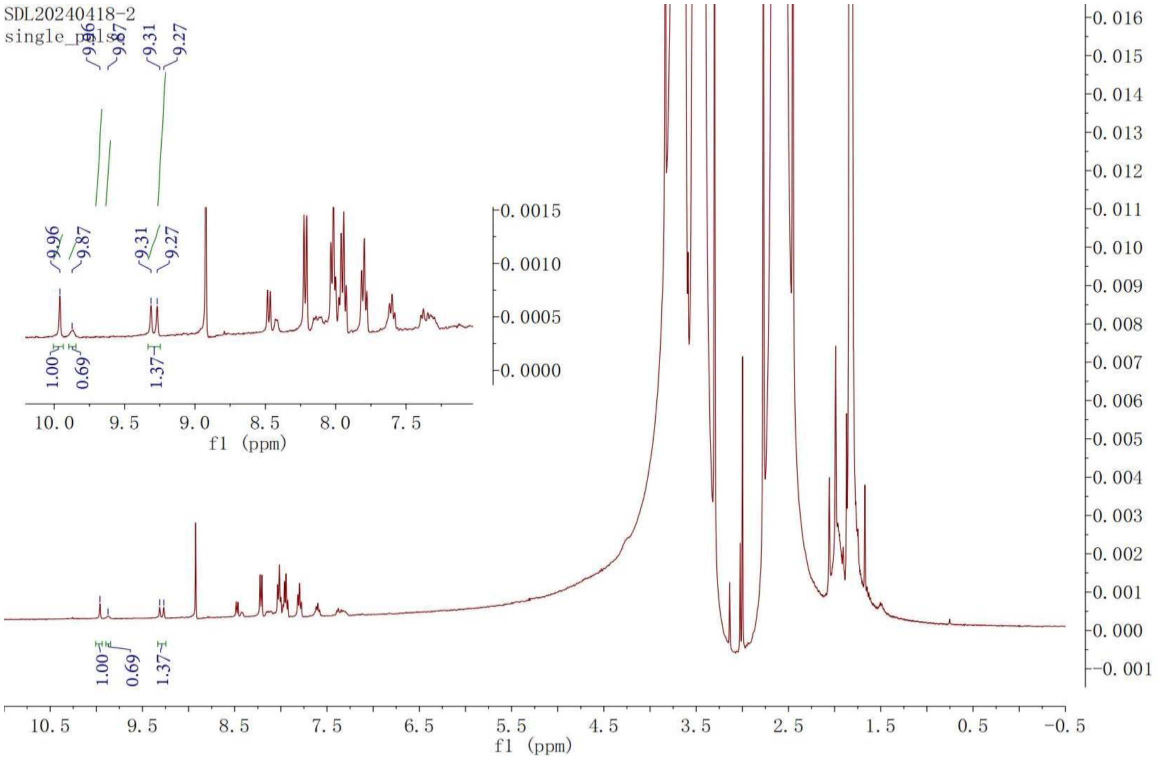

Full spectrum of Figure S5-a, ^{1}H-NMR (400 MHZ, DMA)

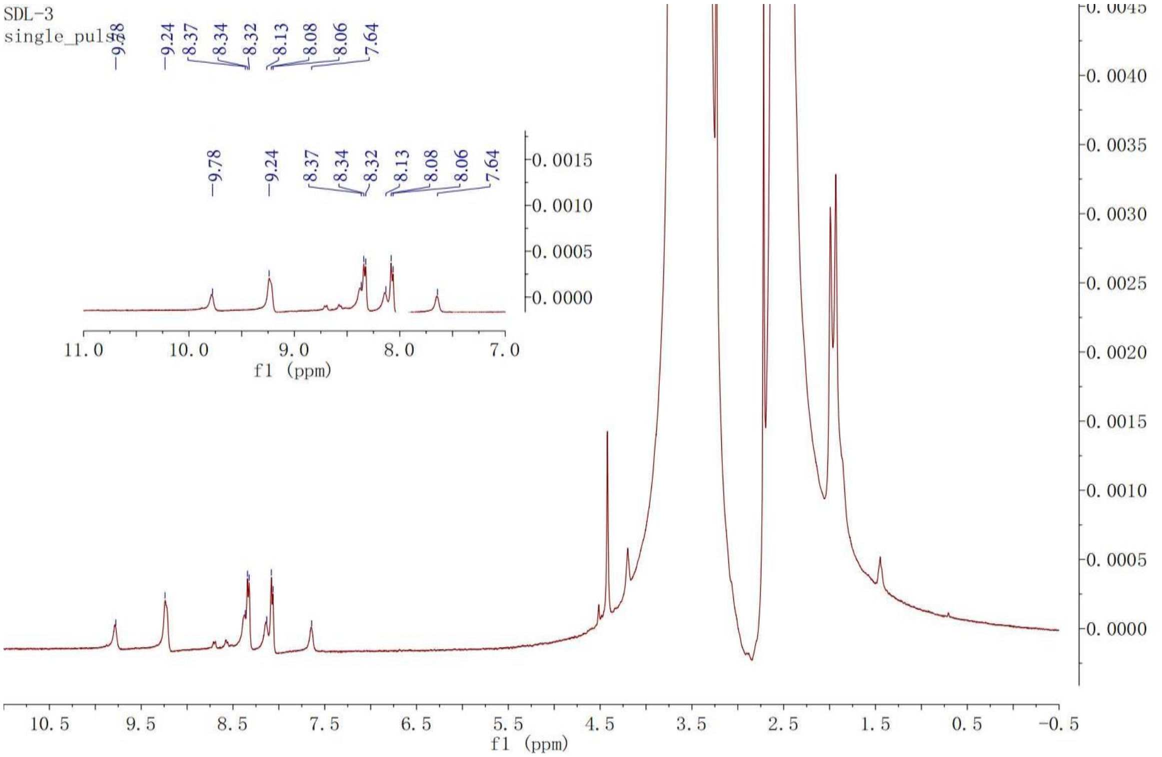

Full spectrum of Figure S5-b, ^{1}H-NMR (400 MHZ, DMA)

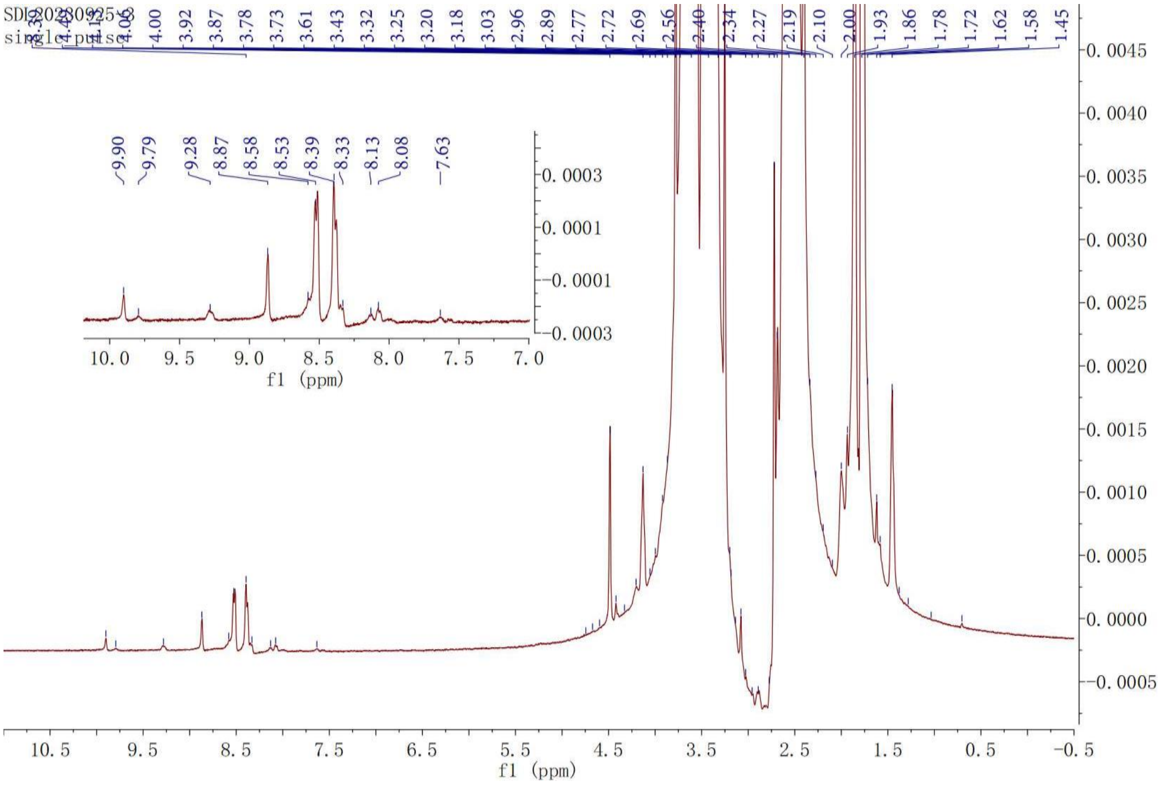

Full spectrum of Figure S5-c, ¹H-NMR (400 MHZ, DMA)

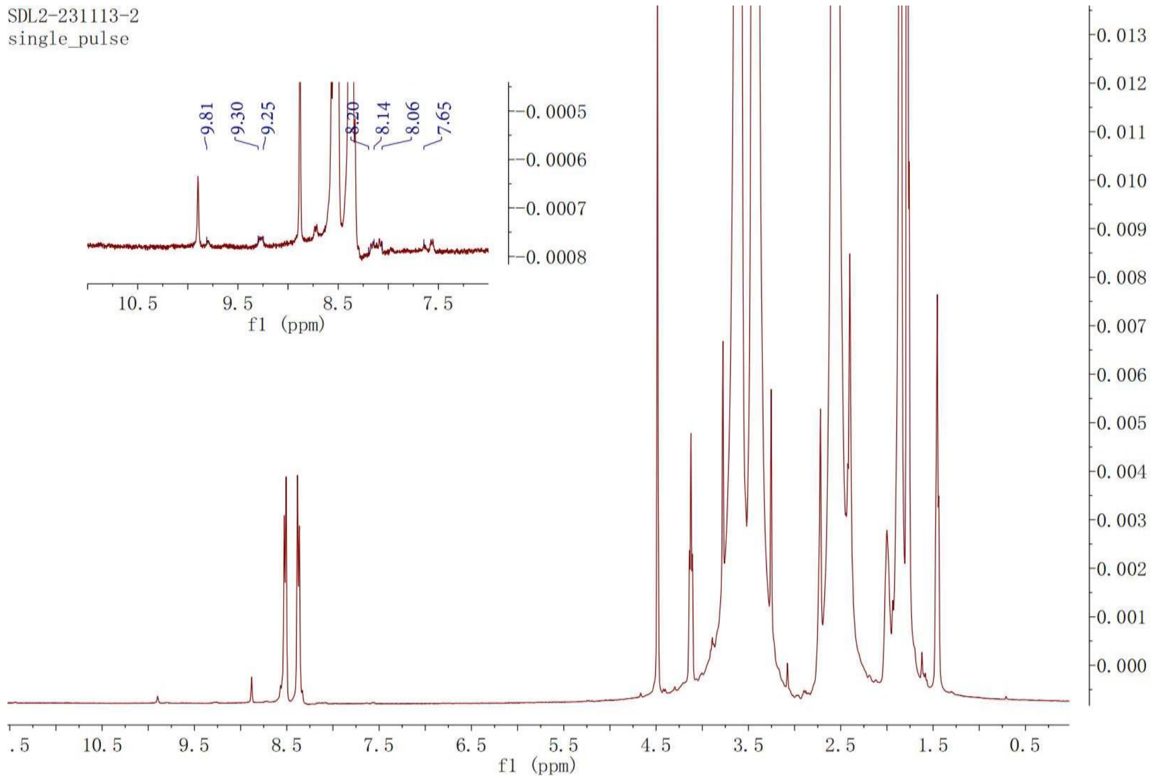

Full spectrum of Figure S6-a, ¹H-NMR (400 MHZ, DMA)

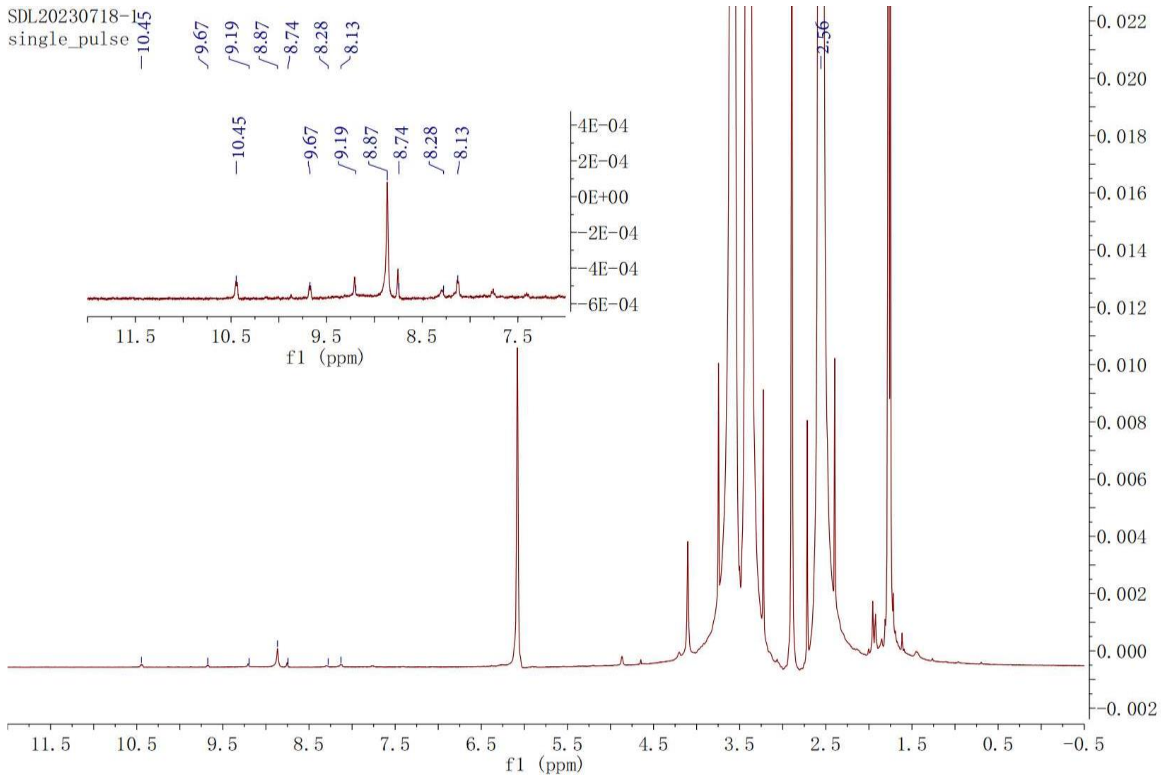

Full spectrum of Figure S6-b, ^{1}H-NMR (400 MHZ, DMA)

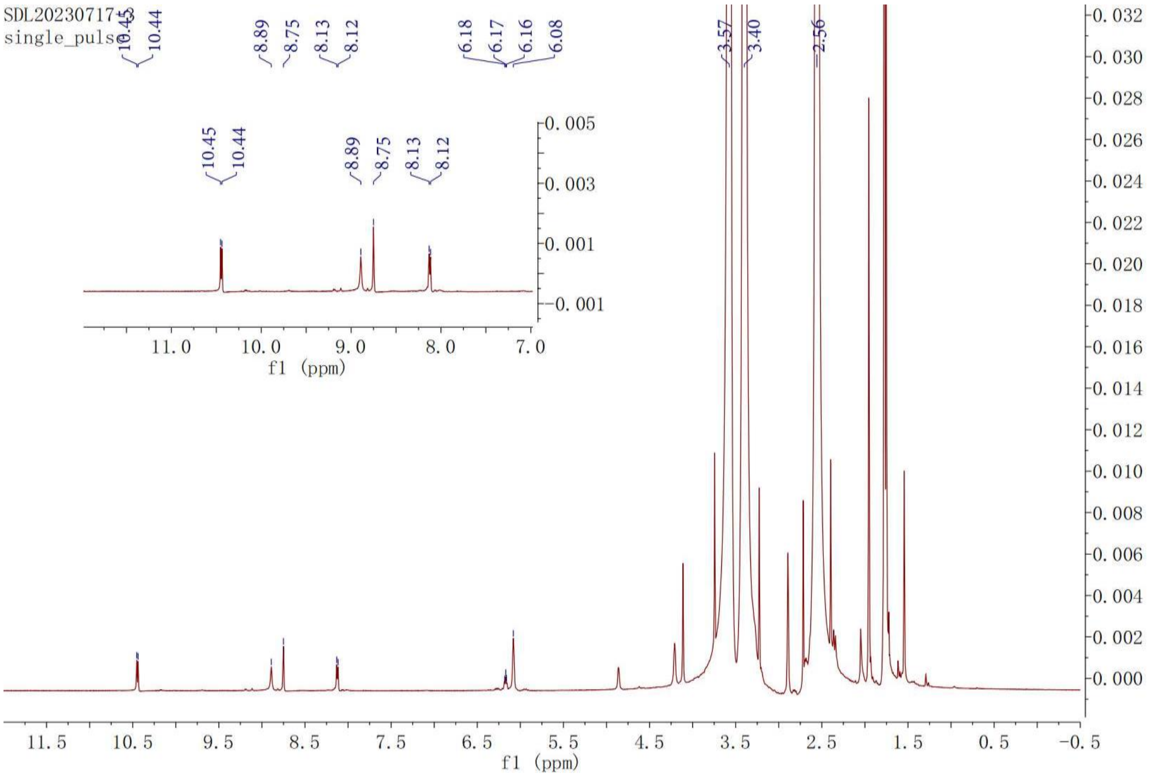

Full spectrum of Figure S6-c, ^{1}H-NMR (400 MHZ, DMA)

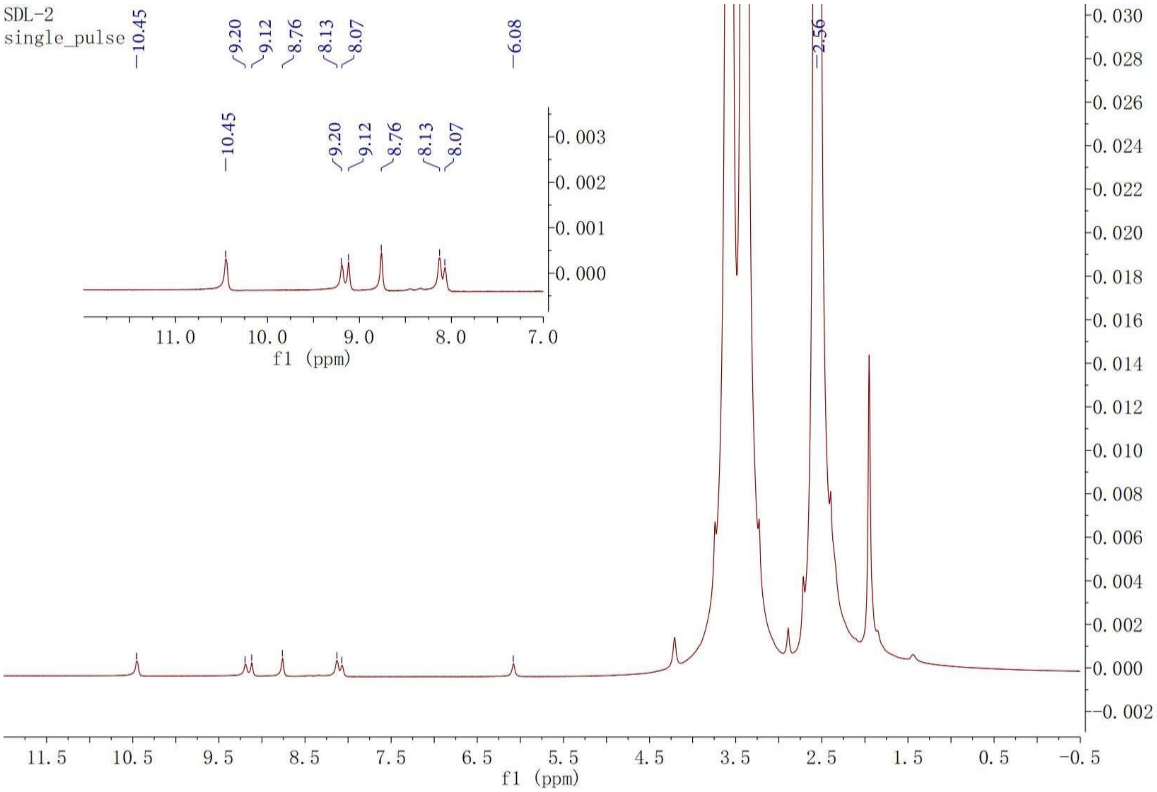

Full spectrum of Figure S6-d, ^{1}H-NMR (400 MHZ, DMA)

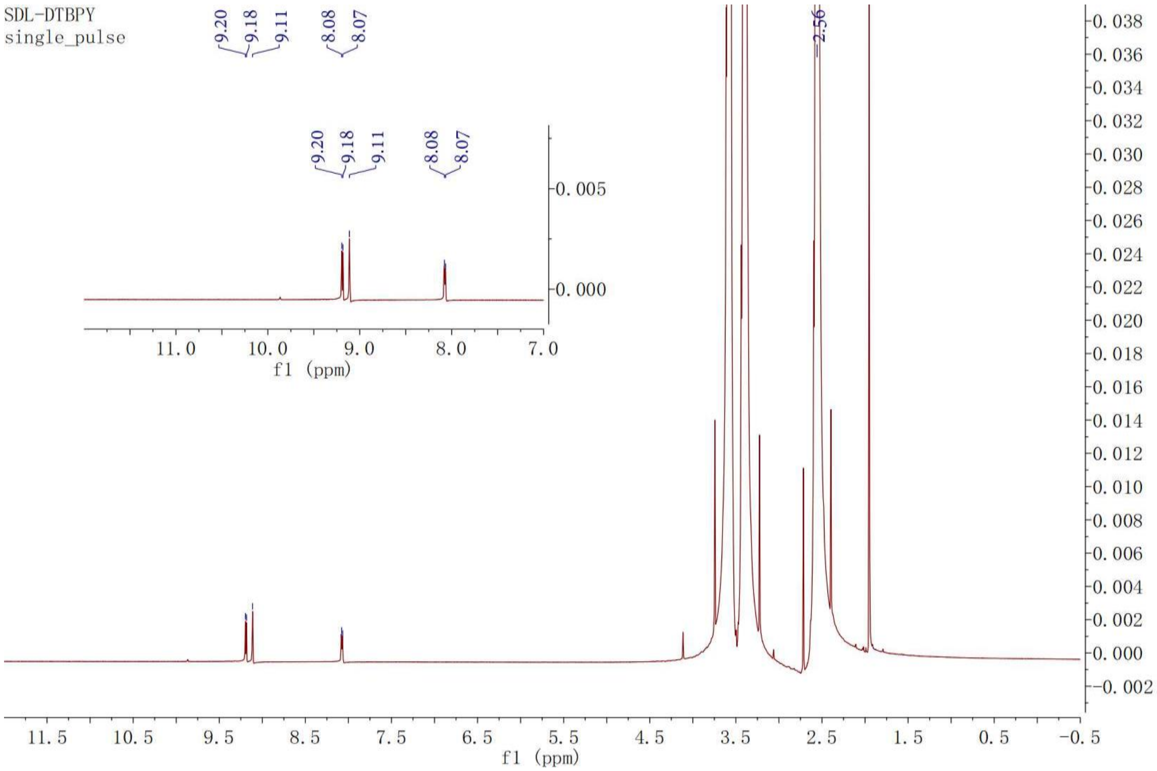

Full spectrum of Figure S8-a, ^{1}H-NMR (400 MHZ, DMA)

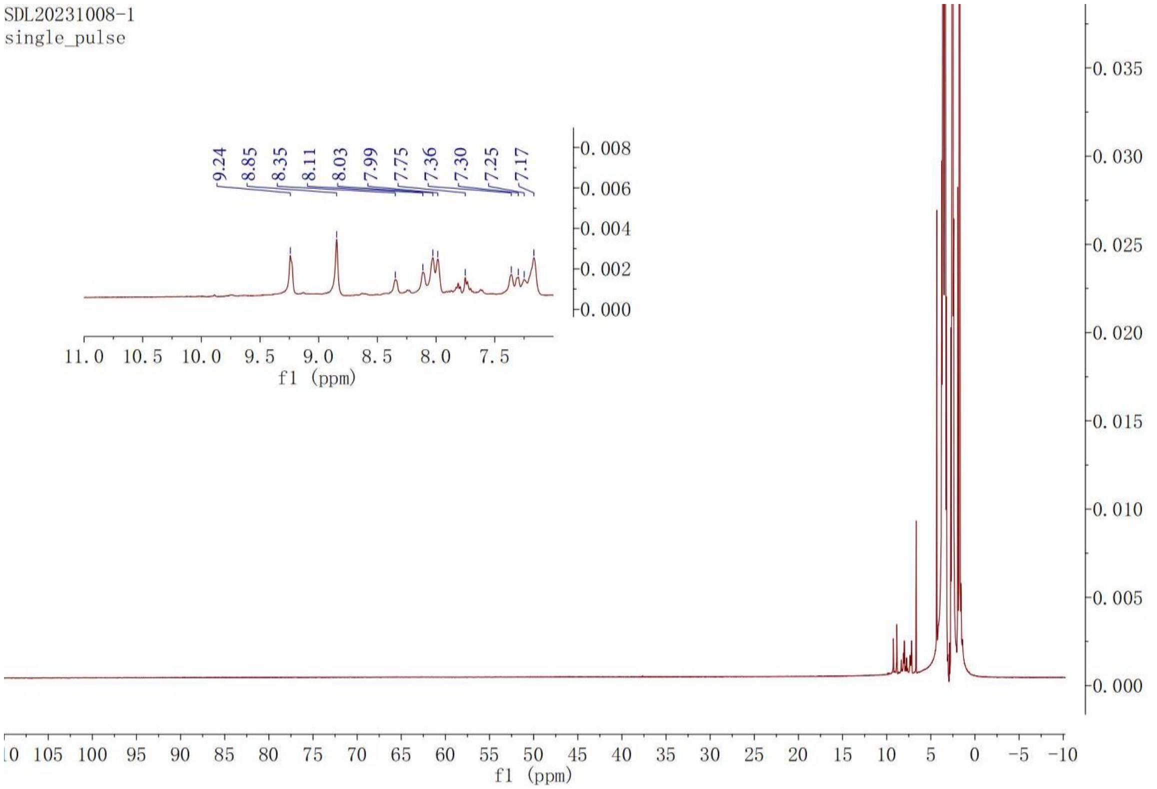

Full spectrum of Figure S8-b, ^{1}H-NMR (400 MHZ, DMA)

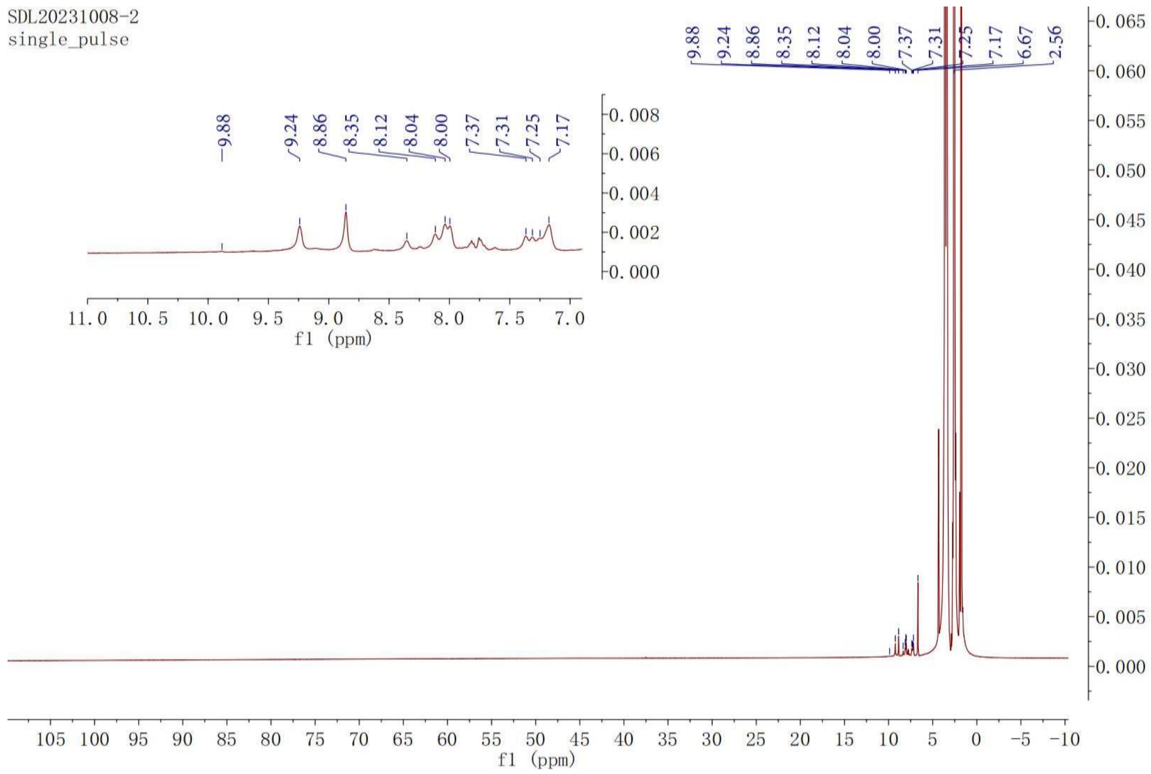

Full spectrum of Figure S8-c, ^{1}H-NMR (400 MHZ, DMA)

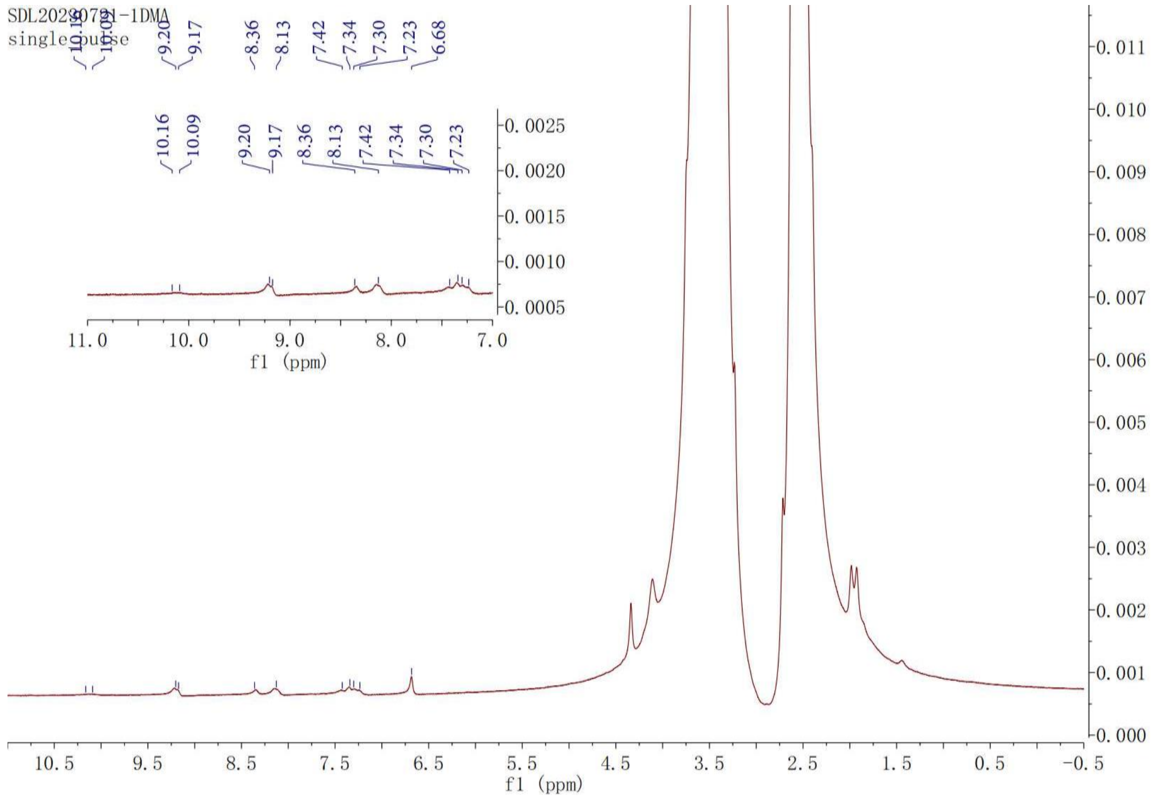

Supplement: Supplementary file 1 — Supporting Information [file ADVS-11-2404301-s001.pdf]
